# Deconstruction and Reassembly of Renewable Polymers and Biocolloids into Next Generation Structured Materials

**DOI:** 10.1021/acs.chemrev.0c01333

**Published:** 2021-08-20

**Authors:** Blaise L. Tardy, Bruno D. Mattos, Caio G. Otoni, Marco Beaumont, Johanna Majoinen, Tero Kämäräinen, Orlando J. Rojas

**Affiliations:** † Department of Bioproducts and Biosystems, School of Chemical Engineering, Aalto University, P.O. Box 16300, FI-00076 Aalto, Finland; ‡ Department of Physical Chemistry, Institute of Chemistry, University of Campinas, P.O. Box 6154, Campinas, São Paulo 13083-970, Brazil; § Department of Materials Engineering, Federal University of São Carlos, Rod. Washington Luís, km 235, São Carlos, São Paulo 13565-905, Brazil; ⊥ School of Chemistry and Physics, Queensland University of Technology, 2 George Street, Brisbane, Queensland 4001, Australia; ¶ Department of Chemistry, Institute of Chemistry of Renewable Resources, University of Natural Resources and Life Sciences, Vienna, A-3430 Tulln, Austria; # Bioproducts Institute, Department of Chemical and Biological Engineering, Department of Chemistry and Department of Wood Science, University of British Columbia, 2360 East Mall, Vancouver, British Columbia V6T 1Z4, Canada

## Abstract

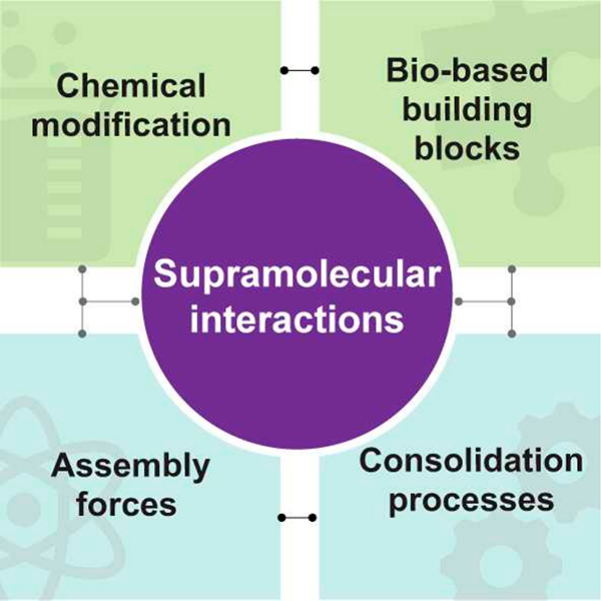

This review considers the most recent developments in supramolecular and supraparticle structures obtained from natural, renewable biopolymers as well as their disassembly and reassembly into engineered materials. We introduce the main interactions that control bottom-up synthesis and top-down design at different length scales, highlighting the promise of natural biopolymers and associated building blocks. The latter have become main actors in the recent surge of the scientific and patent literature related to the subject. Such developments make prominent use of multicomponent and hierarchical polymeric assemblies and structures that contain polysaccharides (cellulose, chitin, and others), polyphenols (lignins, tannins), and proteins (soy, whey, silk, and other proteins). We offer a comprehensive discussion about the interactions that exist in their native architectures (including multicomponent and composite forms), the chemical modification of polysaccharides and their deconstruction into high axial aspect nanofibers and nanorods. We reflect on the availability and suitability of the latter types of building blocks to enable superstructures and colloidal associations. As far as processing, we describe the most relevant transitions, from the solution to the gel state and the routes that can be used to arrive to consolidated materials with prescribed properties. We highlight the implementation of supramolecular and superstructures in different technological fields that exploit the synergies exhibited by renewable polymers and biocolloids integrated in structured materials.

## 1 Introduction

From the materials point of view, living organisms comprise organic/inorganic complex composites that fulfill multiple functions. They are built from elementary components into hierarchical structures. This review centers on such materials and their deconstruction to obtain building blocks that ultimately are reassembled or engineered into materials. Water and associated interactions forces are critical in related transformations. Together with the nanotechnologies of renewable materials, these subjects have witnessed an exponential growth during the past decade. Ultimately, our interest is to supply solutions to materials needs. For this purpose, it has been proposed that efforts should aim at substituting products from fossil carbon and consider a shift, under the concept of the circular bioeconomy. Specifically, we suggest the opportunity to use the assets found in natural organizations to achieve given functions and properties. Natural polymeric building blocks have evolved to adapt to environmental demands and stresses. They can be harnessed, using the power of supramolecular and supraparticle chemistry, to sustainably develop materials and bioproducts resourced from the land and oceans, mainly plant feedstocks, and residual streams from the agricultural and food chains. This is of course an extremely vast topic and this account only scratches the surface. We have evaluated the literature published in the recent years and have organized this in an attempt to make every subject self-contained. Several of the figures in the next sections were drafted from scattered information and aggregated in such a way to deliver a compact message together with the discussion provided in the different sections, which we hope the reader finds useful.

### 1.1 Building Blocks from Biomass: Enabling the Future Bioeconomy

The global economy is adopting a circular model to maintain the current standard of living while protecting the environment.^1^ This demands a material paradigm shift that benefits from the use of regenerative biological systems, which are also considered under the concept of the bioeconomy, ultimately leading to the circular bioeconomy.^2^ Together with recycling, and upcycling, the use of renewable resources and clean energy bring major benefits as far as carbon sequestration, for example, via the continuous and efficient use of the sustainably managed forest.^3,4^ Related operations reduce soil degradation and maintain productivity while recompositing of the organic matter in the soil leads to natural conversion into humic acids. Meanwhile, annual plants, typical of agricultural operations, are most significant to the future bioeconomy since they grow considerably faster and involve more frequent harvests.^5^ The utilization of associated side streams has a major socioeconomic appeal given the increase in the land footprint of the respective supply chain and the disposal costs, if not consumed. Sourcing from agricultural side-streams also opens the possibility for strong economic growth and supports agriculture-centered economies. Lastly, potential sources of material building blocks also include byproducts from fisheries and livestock industries^6,7^ as well as those found in downstream biotechnological processes.^8,9^

Natural biopolymers can be modified to introduce new properties or to adjust their interactions with other components and, especially with water, which is critical considering their behavior in suspension or in solution and their transformation into materials.^10^ In such processes, dynamic bonds are formed, which define the mechanical properties of the ensuing materials and their superstructures.

The shift from fossil carbon to biobased sources adds a challenge given that the respective building blocks and end-products are remarkably different. Although substitution strategies are easily identified in few specific cases, such as the replacement of phenol in adhesive resins with lignin, the adoption of biobased polymers is still largely unexplored. In related efforts, one can gain inspiration from natural assemblies, which involve hierarchical structures that are strong and tough. All in all, understanding the deconstruction, modification, and manipulation of biobased building blocks is critical to enable a fully circular bioeconomy.

### 1.2 Renewable Biopolymers

**Figure 1:**
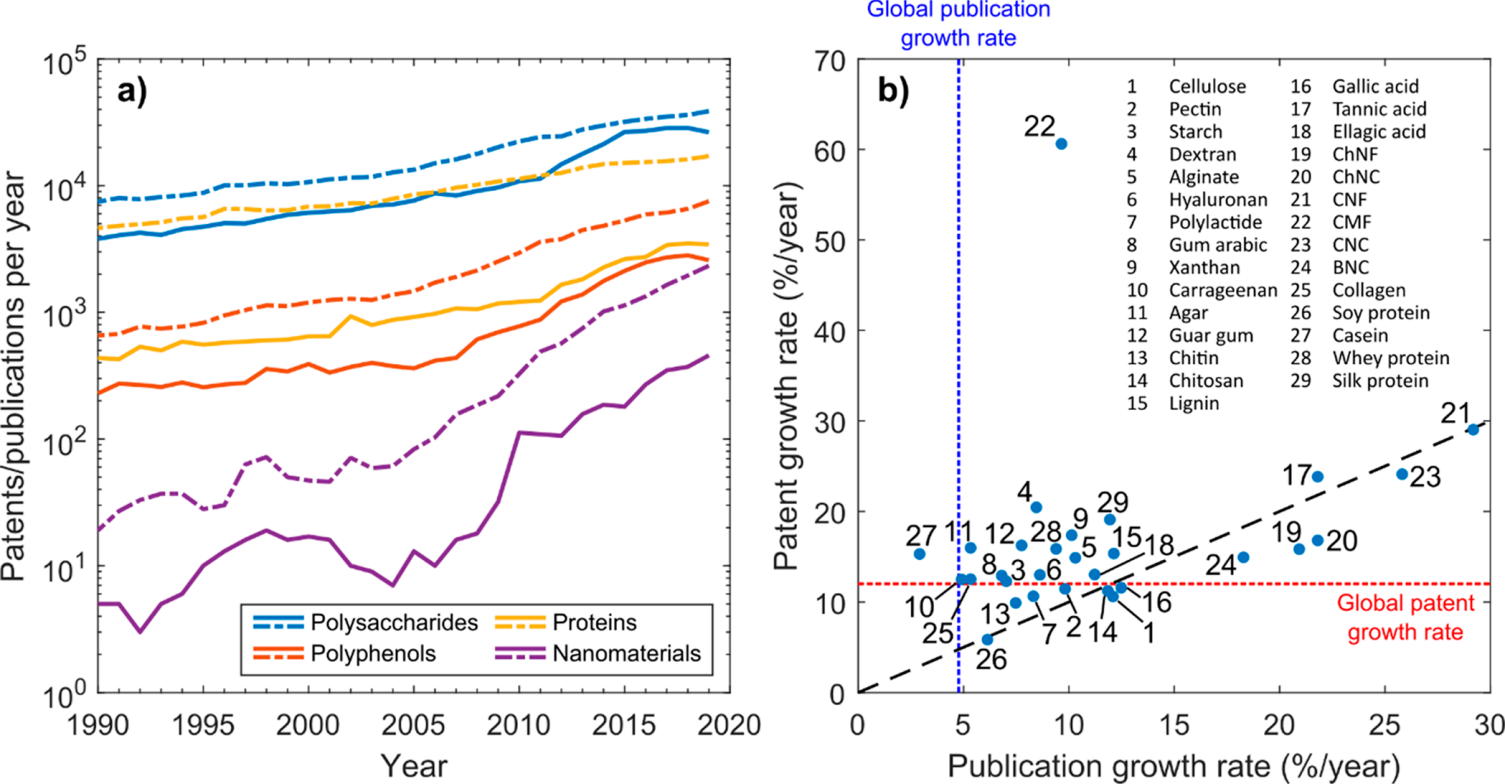
(a) Overview of the number of publications (dashed lines) and patents (continuous lines) per year (period between 1990 and 2019) related to polysaccharides (cellulose, pectin, gum arabic, xanthan gum, guar gum, starch, dextran, alginate, hyaluronan, carrageenan, agar, chitin, chitosan), polyphenols (lignin, gallic acid, tannic acid, ellagic acid), proteins (silk protein, collagen/gelatin, soy protein, casein, whey protein), and renewable nanomaterials (CMF, CNF, CNC, BNC, ChNF, ChNC). (b) Growth rate of patents and publications compared to the approximated global rates, used as reference. A fit to the trend is added following the equation *A*(1*+ B*/100)^year^, with parameters *A* and *B* used to estimate the annual growth rate (*B*) of the number of patents and publications for each material. Note the current interest in cellulose nanomaterials (21, 23), nanochitins (19, 20), tannic acid (17), and bacterial nanocellulose (24). Cellulose microfibers (CMF, 22) have been the subject of interest by industry, as judged by the comparably larger patent growth rate. See Supporting Information for details.

The main biopolymer architectures found in nature include polysaccharides, proteins, and polyphenols. Polysaccharides and polyphenols are mainly resourced from plants while proteins are principally byproducts of animal farming. Plant-based biorefineries, mainly cellulosic processing units, are highly optimized to achieve high yields with low environmental costs. In contrast, other byproducts such as gelatin, chitin, and chitosan have significantly higher costs of extraction. However, as is the case for plant-derived polymers, they are available at industrial scales and will have an important role in the future bioeconomy. Biotechnological production of silk precursors is underway, offering great promise in the synthesis of high-performance proteinaceous materials. These and other biopolymers are considered in this review, summarized in Figure 1. Therein, the term “polysaccharides” is used to refer to cellulose, chitin, chitosan, hemicelluloses (including guars, alginate, pectin), and starch. Meanwhile, “polyphenols” include tannins and lignins. “Proteins” are mainly represented by silk, amyloid fibrils, soy protein byproducts, gelatin (and collagen filaments), and those derived from whey (Figure 1a). As can be seen, the majority of research focuses on plant-based biopolymers due to the fact that they are readily available for materials preparation and are associated with the historical use for manufacturing. However, current research clearly indicates that the “plant-based” foundation can be extrapolated to a wide range of other bioresources, for example, for their use in the formation of sustainable materials. Furthermore, although at smaller scales, material fabrication from protein fibers, such as silk and their biotechnological derivatives is under considerable examination.^11−16^ The interest placed by the research communities in relation to the major biopolymers can be quantified by the annual growth of patents and publications, which surpass the growth in the respective global research outputs, Figure 1b. Renewable nanomaterials (nanocelluloses and nanochitins) have captured the attention (Figure 1a,b) of academia and industry, as evidenced by the exponential-like growth rates in the number of publication and patents, Figure 1b. These materials also have individually among the smallest absolute number of patents and publications within the group (not shown).

### 1.3 Scope of This Review

**Figure 2:**
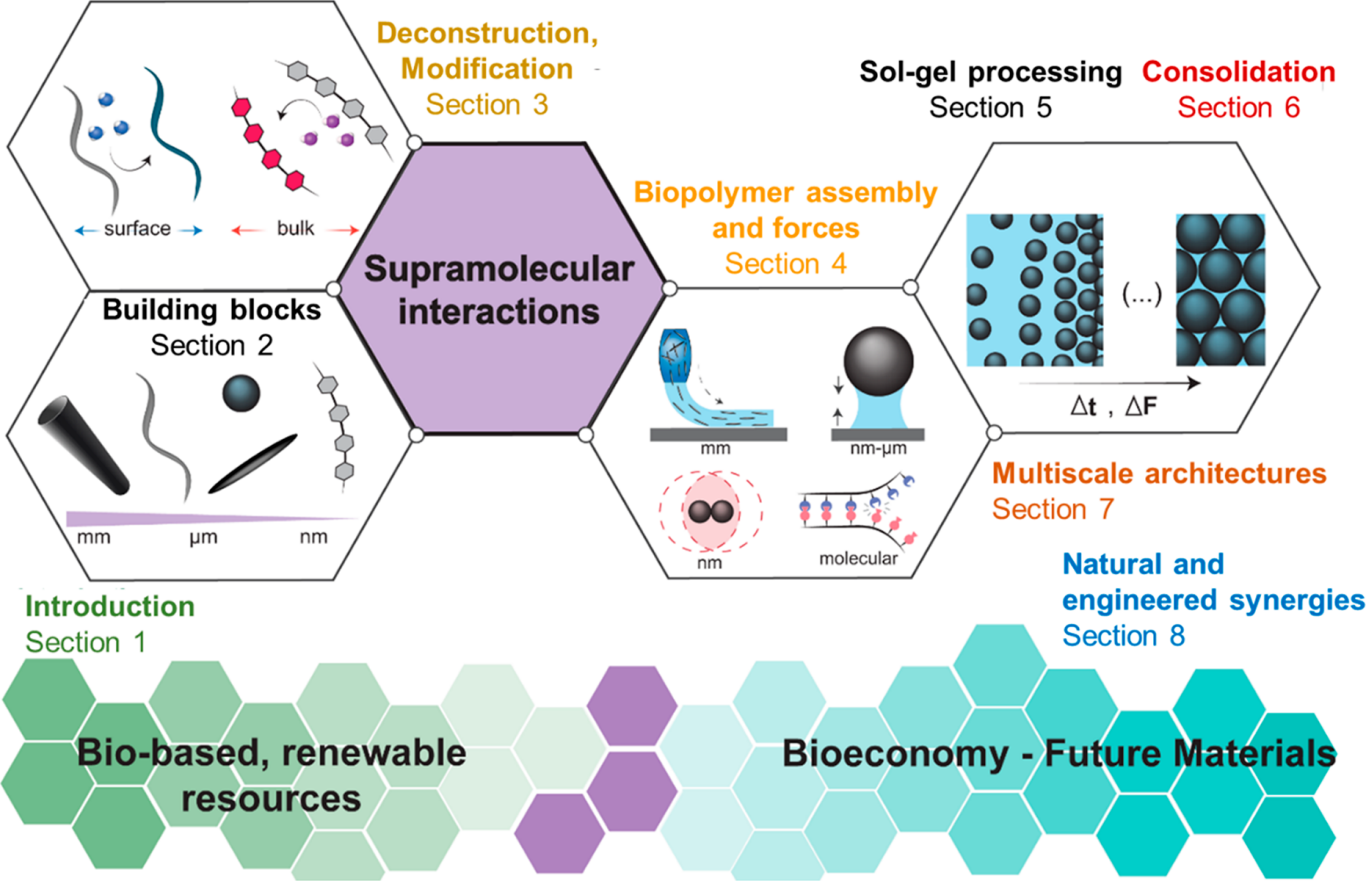
Schematic illustration of the organization of this review, including a brief introduction (Section 1) and the relationship between the building blocks and their associated size scale (Section 2) and chemistry (Section 3) as well as the assembly forces (Section 4) involved in their consolidation (Sections 5 and 6) when processed from solution or suspension into gels and solids, enabling breakthroughs in materials relevant to the bioeconomy. Supramolecular and supraparticle interactions are associated with all these aspects, playing pivotal roles in engineering the next-generation materials from biomass (Sections 7 and 8).

This review addresses comprehensively the topic of natural biopolymers and their assemblies as sources of sustainable materials for the bioeconomy (Figure 2, Section 1). We highlight building blocks with low environmental footprint, with a performance that matches or competes favorably compared to the synthetic counterparts as well as those that are readily available at industrial scales (Figure 2, Section 2). Several recent reviews on the topic of “biopolymers” are available, mainly focused on applications, such as packaging, and covering a subset of biopolymers used in a material synthesis and associated physicochemical phenomena.^17−26^ Herein, we address the assembly of biopolymers that are sustainably sourced, aiming at material fabrication and highlighting each of the individual steps, from deconstruction to modification, processing and scaling-up (Figure 2, Sections 3). The review is framed in the context of the physical forces acting across these processes (Figure 2, Section 4) and the resulting supramolecular and particle interactions that result in material consolidation (Figure 2, Sections 5 and 6). We discuss the fundamental aspects of molecular interactions, key in self-assembly of biobased colloids, and materials at varied length scales. The deconstruction (e.g., isolation of the polymer or colloid) is highlighted, bearing in mind the recent efforts to transfer cohesion from macromolecules to bulk materials. Importantly, we illustrate the potential of building blocks and their chemistry, processing, impact of assembly conditions and obtained superstructures (Figure 2, Section 6 and 7). We close the review by discussing multiscale materials and the synergies in multicomponent systems (Figure 2, Section 8) and the progress achieved over the past decade, hinting to the prospects of high-performance sustainable biobased materials. The principles put forward herein can be applied across manufacturing streams including roll-to-roll fabrication, additive manufacturing (including 3D-printing), molding, compositing, and microfiber/microfilament synthesis, among others.

## 2 Supramolecular Chemistry of Renewable Biopolymers

In biosynthesized systems, the structure–property relationships are tied to specific functions, associated with the organisms’ lifecycle, which is optimized by evolutionary processes. Consequentially, a variety of functional groups and polymeric architectures are utilized by nature in response to environmental challenges. Biopolymers are quite diverse compared to synthetic counterparts. The latter generally have a linear carbon-centered backbone (e.g., polyolefins and polyaromatics such as polyethylene and polyterephthalates); by contrast, polysaccharides and other oxygen-enriched molecular building blocks form the backbone of the repeating units of renewable biopolymers. In this section, we introduce the main biopolymers, short-listed in Section 1.2, and highlight their chemical structure and functionality at the molecular scale. Their functions and supramolecular interactions are discussed in relation to their role in biological matrices and in engineering new materials. We discuss biopolymer interactions in the living state or in the plant and consider topological aspects in the multiscaled architectures typical of biomass.

### 2.1 Structure and Functional Groups in Renewable Biopolymers

**Figure 3:**
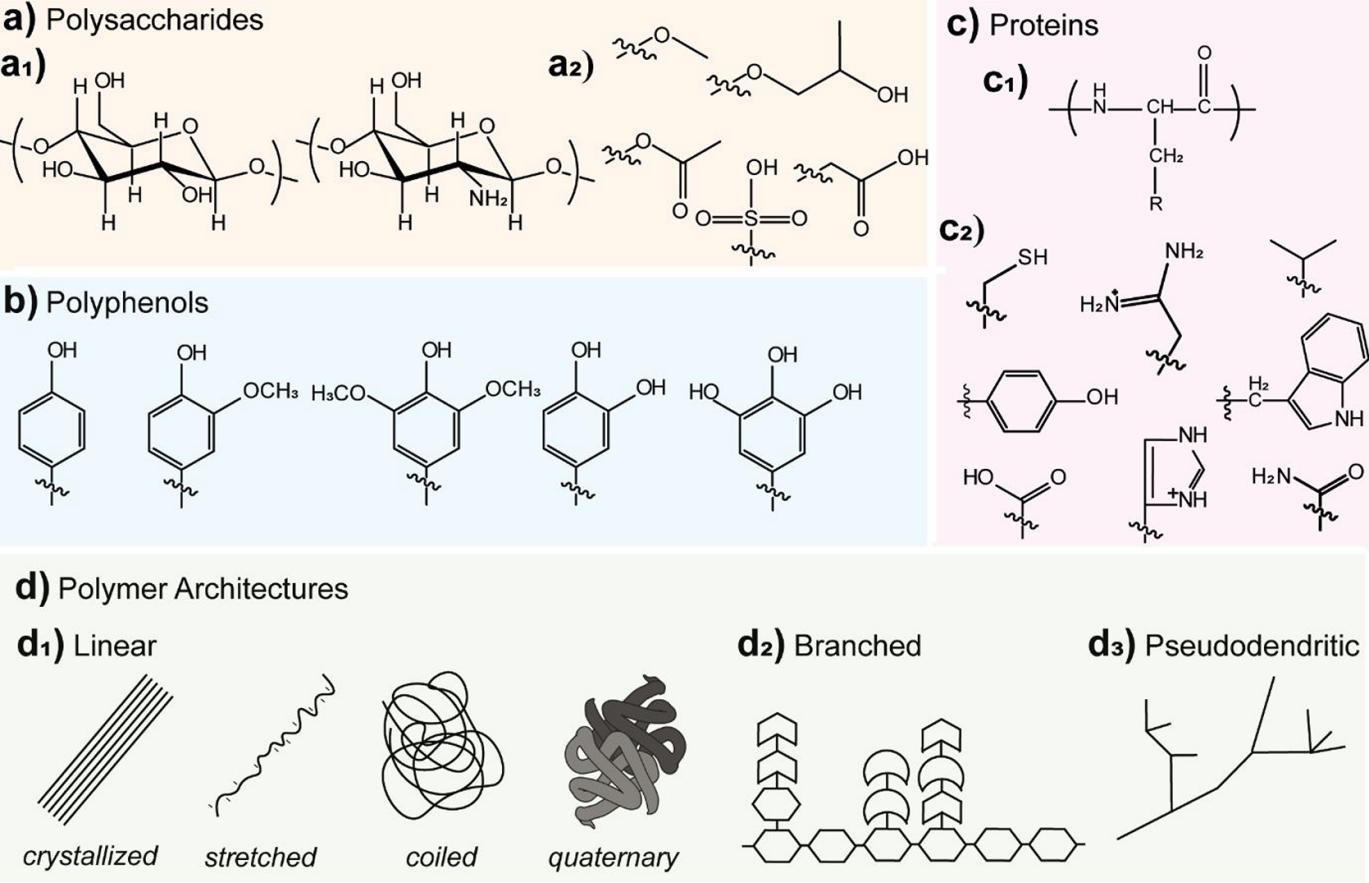
(a–c) Illustration of the monomeric units and functional groups typical of biopolymers. (a1) Cellulose and chitosan monomer units and (a2) commo chemical modifications of the primary and secondary hydroxyl groups. These include sulfated cellulose nanocrystals, methylcellulose, hydroxypropyl cellulose, cellulose acetate, and carboxymethylcellulose. (b) Aromatic groups present in most phenolic structures such as lignins and tannins. Typically, condensed structures are found in tannins. (c1) Monomer unit of polypeptides, the primary constituent of proteins. (c2) Examples of functional groups presented by proteins (note that, in addition, glycosylated proteins are commonly encountered). This set is representative for the supramolecular and covalently interacting functional groups. (d) Polymeric architectures typically found in biopolymers. (d1) Conformation observed for linear polymers such as cellulose, chitosan, and proteins. (d2) Branched architectures obtained from a linear backbone as typically found in denatured glycosylated proteins, hemicelluloses, or gums. (d3) Pseudo dendritic architectures observed for tannins and lignins.

The increasing knowledge about biosynthetic processes and the development of new extraction routes result in a vast variety of accessible biopolymeric structures,^8,24^ which can be sourced as linear macromolecules or highly branched dendrimer structures, with polymerization degrees ranging from nearly oligomeric, below 1 kDa, to the MDa scale. Typically, the types of biopolymers used in man-made materials represent a small subset of those available in nature. This is a consequence of their native interactions, which require chemo-mechanical energy to be broken during isolation of these biopolymers. In parallel, with the renewed interest toward biopolymeric structures, a deeper understanding of their relationship with the properties of derived material is sought after. Therein, we review the supramolecular and supraparticle interactions involving biobased oligomers, biomacromolecules, and biocolloids. The noncovalent interactions and their binding constants vary significantly and depend on the respective functional groups (Figure 3a–c). Furthermore, the topology of the polymeric networks and branching dramatically affects their interactions (Figure 3d). This section introduces the range of functional groups and polymeric architectures readily available in materials science, as obtained from biomass. Their functionalities, expected interactions, and structure–property relationships are discussed. We also present the higher order interactions observed in mesoscale polymer assemblies (tens of nm) and in the microscales, for example, typical of biobased biopolymeric constructs, biocolloids. We introduce their “idealized” structures as a result of biosynthesis and after extraction from residual streams. A deeper understanding of their fundamental properties and structures is expected to enable a better correlation and prediction of the properties of the materials they form.

#### 2.1.1 Polysaccharides

About 0.1–1 Gt of cellulose is photosynthesized every year.^27,28^ Pulp production, the main source of commercial cellulose, reached 330 Mt in 2000 and is witnessing a steady increase for packaging and lightweight grades.^27^ The most common commercial routes for extracting cellulose, sustainably, consist principally of wood and waste streams from agricultural residues, including fast-growing plant species such as annual plants and those resulting from aquaculture (e.g., algae). However, these cellulose sources are usually combined with hemicelluloses or lignins. Although presenting a reduced sustainability due to high water consumption, cotton, and streams of high cellulose purity obtained from bacterial biosynthesis, have high purity, molecular weight (MW) and crystallinity. Microbially produced cellulose assemblies, that is, bacterial nanocellulose (BNC), have the advantage of being synthesized at the air–water interface, enabling unique cellulose macrostructures.^29−31^ Less exploited sources of cellulose include animals, principally ascidians such as tunicate. The MW of the latter cellulose source ranges from a few kDa to MDa, with a polymerization degree, *n*, from <100 to about 50 000.^27^ The associated distribution of the extracted cellulose is generally polydisperse. Although it is the simplest polysaccharide architecture, with a linear chain without backbone branching, cellulose self-assembly leads to a wide variety of architectures. Because of its high self-affinity, cellulose is poorly soluble in most solvents, including water,^32^ and its crystalline polymorphs are hydrophilic.^33^ The low solubility is because the intermolecular hydrogen bonding of cellulose with itself is stronger than that between cellulose and water.^34^ As a result, modifications of cellulose by methylation or acetylation (which intuitively should yield a less hydrophilic material) affects these cohesive interactions as well as water solubility.^35^ The various dimensions of extracted cellulosic building blocks, from nanocrystals to fibers, significantly impact the interactions with surrounding polymers and molecules.^36,37^ The interactions within the crystallites, that is, a solid, involve hydrogen bonding, van der Waals (vdW), hydrophobic and Coulombic forces. The interactions at the surface of cellulose constructs appear to depend on the polymorph type, and principally behaves as a typical hydrated oxide layer, with a “frozen” layer of water molecules bound by interfacial hydrogen bonding.^38^ Nanocelluloses, mainly cellulose nanocrystals (CNCs) and cellulose nanofibers (CNFs), are structured assemblies derived from elementary fibrils of plants, industrially produced at ∼13.9 kt annually.^39^ Interestingly, during defibrillation, intermediate steps lead to higher crystallinity.^40^ Regarding cellulose derivatives, the vast majority are produced from dissolving grade pulps, due to the requirement of low impurity content. In 2003, dissolving-grade pulp was produced at an annual rate of 3.65 Mt,^27,41^ and increased to 7.5 Mt in 2015,^42^ which was associated with an increase in production of cellulose derivatives and man-made cellulose fibers, such as Viscose and Lyocell.

The polymer of *N*-acetylglucosamine linked by β-1,4-acetal linkages, that is, chitin, is synthesized in a wide range of organisms, such as insects, mushrooms, and crustaceans. Upon extraction, deacetylation (>45%) leads to the formation of the soluble form, that is, chitosan.^43−47^ Estimates point to 0.01 to 0.1 Mt being produced in the biosphere annually,^25^ while around 2 to 5 kt of chitosan and glucosamine are extracted from a small subset of the available waste products.^48^ The molecular mass of chitin range between 1 and 2.5 MDa and 0.1 to 0.5 MDa in chitosan.^49,50^ The N-acetyl groups strongly interact with proteins, leading to a substantial effect on the material properties of the mixtures. As observed in the squid beak, protein-chitin interfaces are used as a tool to control water interactions^51^ in a manner reminiscent of lignin-cellulose-hemicelluloses complex. Importantly, due to the presence of the acetyl group, chitin participates in a hydrogen bonded network while its surface is considerably less polar than cellulose. In addition to hydrogen bonding and van der Waals (vdW) forces, chitin undergo electrostatic and π-cation interactions. As such, it is the only positively charged polysaccharide that undergo electrostatic interactions with negatively charged surfaces and molecules. Chitin p*K*_a_ is ∼6.2,^52^ below which amine groups are protonated (positively charged).

Although obtained from side streams or at smaller quantities, there exists a larger range of polysaccharides besides chitin and cellulose. Most of these polysaccharides present a significantly higher water solubility than chitin or cellulose. For instance, in wood, hemicelluloses represent 15 to 25% of the dry mass, while it can reach values above 40% for seasonal plants.^53^ As obtained, hemicelluloses have a polymerization degree typically ranging from 50 to 200, with a M_W_ < 90 kDa, substantially smaller than cellulose.^53,54^ Hemicelluloses are highly branched heteropolymers of pentose, hexose, uronic acids, and acetylated sugars, with xylan being the most frequently occurring structure.^55^ Mannans and xyloglucans are the two other types also commonly found in a partially acetylated form. Xylan has a backbone exclusively composed of xylose, with partial acetylation and bares specific interactions with phenolic residues such as ferulate esters, which result from cross-linking, for instance with lignin residues. They also possess a negative charge from the carboxylic acid groups in glucuronic backbone moieties. The backbone of mannans is composed essentially of mannose. Because of their high diversity in topology and functional groups, hemicelluloses have a high potential to form ordered multivalent interactions with various biopolymers.^56,57^ However, thus far, the nature of the supramolecular interactions remains limited and is tied principally to subsets such as lignin-carbohydrate complexes (LCCs).^58^ Current efforts exploit the heterogeneity in structure and interaction of hemicelluloses to self-assemble materials, for instance to form nanoparticles.^59^ A particularly interesting aspect of hemicelluloses is that the topology of their branching is expected to follow regular sequences associated with a range of biosynthetic factors, leading to multiblock architectures as observed in their native structure.^60^ Exudate gums and highly water-soluble gums are another class of highly branched and chemically heterogeneous polysaccharides. The three main classes of gum exudates are gum arabic, gum tragacanth, and gum karaya.^61^ Gum arabic, the principal gum obtained at Mt quantities, is highly branched and water-soluble, with a MW ranging from 0.3 to 1.6 MDa.^61^ Approximately 10% of gum arabic is composed of arabinogalactan–protein complexes, which interact substantially with various polymer and interfaces. Gum karaya has a MW as high as 16 MDa and features 40% uronic acid and 8% acetyl residues.^61^ This renders the biopolymer both negatively charged and amphiphilic. There exists a wide variety of gums beyond exudate gums obtained from numerous sources.^62^ In recent years, dextran, carrageenan, konjac, pectin, agar, hyaluronates, and alginates have been described in numerous applications.^24^ They are presented with a wide range of topologies, chemical functionalities, and hydrodynamic conformations. Both pectins and, more commonly, alginate are well-known for their high affinity with calcium and other divalent cations in the so-called “egg-box” configuration that leads to cross-linked structures, a topic that is discussed in Section 5.3.2.^63−65^

Although nonfood biomass is preferred in sustainable materials science,^66^ starch presents some interesting properties. Starch blends used in the packaging industries are produced at up to ∼200 kt per year.^67^ Starch is a mixture of linear amylose and branched amylopectin, with both having glucose as the backbone (α(1 → 4) linkages). Amylopectin possesses a MW of up to 100 MDa, although it is quite compact due to its branched nature and semicrystalline arrangement. Amylose, with a MW between 0.1 and 1 MDa, is well-known for its ability to form inclusion complexes due to the chiral twists imparted by its chain conformation, giving rise to an amphiphilic 3D structure in water.^68^ This is also reflected in the polymeric interactions of cyclodextrins obtained by cyclization of starch, where water displacement competes with the complexation of molecules in their cavities.^69^

#### 2.1.2 Polyphenols

Besides oligomeric and hydrophobic aromatics such as rosins, terpenes and natural dyes, the main polymeric type of aromatics found in plant biomass are polyphenols, which mainly include lignins and tannins. Lignins are available as byproducts of wood processing in pulp mills and is one of the biopolymers with the highest potential for valorization. Although their biodegradability is relatively low,^70^ they pose no environmental concerns when reintroduced in the ecosphere.^71^ Principally, lignosulfonates (produce in the range of 1.8 Mt per year),^72^ kraft lignins (estimated at 55 Mt per year), and soda lignins (estimated at 6 Mt per year) can be obtained from such processes but, with the introduction of biorefineries, larger volumes of this biomacromolecule will be made available.^73^ Most industrial lignins are burnt for energy and chemical recovery and only 2% of all lignins and only 100 000 tons of Kraft lignins are valorized per year.

In terms of supramolecular interactions, the quadrupole of the aromatic groups of polyphenols and the various substitutions enable a highly heterogeneous polarity distribution, which make polyphenols to act as adhesives to a wide variety of surfaces, by the effect of highly nonspecific, universal interactions. Overall, natural polyphenols can undergo hydrogen bonding, π–π, π–cations, electrostatic, and hydrophobic as well as van der Waals (vdW) interactions. For instance, these interactions enable catechols and gallols to adhere to virtually any surface,^74−76^ and most natural polyphenols bind strongly to proteins, leading to rapid aggregation and disruption of proteinaceous crystalline domains.^77,78^ Lignins produce exfoliation of graphite into graphene nanosheets through π–π interactions,^79^ while π–cation interactions enable strong binding to metallic hydroxides, with a strength that is up to 3-fold higher than with nonmetallic hydroxides such as silica.^80^

Both industrial and native lignin structures are considered as cross-linked polymers of p-hydroxyphenyl (H), guaiacyl (G), and syringyl (S) primary units.^81^ Lignins contains carboxylate groups, giving them a negative charge above pH 3.5, although the isoelectric point is generally above pH 9 due to the high number of phenolic groups (p*K*_a_ 8–10).^82^ The MW of industrial lignins is between <1 kDa to 6 kDa, but larger molecules can be extracted, for instance in the form of lignosulfonates. The number of interunit linkages and functionalities are quite diverse, depending on the lignin type, Kraft, soda, organosolv (MW = 0.5–3 kDa), steam explosion, fractionated, enzymatic hydrolysis and lignosulfonates (MW = 5–400 kDa).^83−86^ Lignins are amphiphilic and possess generally a low aqueous solubility below pH 10. “Alkali” lignins are generally referred as the sodium salt of lignins and are highly water-soluble. Lignins are known for their interactions with metal ions with which they efficiently form coordination complexes.^87^ Lastly, their affinity with carbohydrates, abundantly present in biological structures, is preserved at least to some extent after extraction.^88^

Tannins, generally obtained from bark and outer morphological components of trees and shrubs, have a considerably higher amount of hydroxyl groups than lignin, leading to a higher reactivity, broader pH-responsiveness, higher water solubility, and increased affinity with metals via coordination bonds.^89^ Tannins possess catechinic acid-like and galloyl functional groups, which form strong coordination bonds that are hydrolyzable in mild acidic conditions.^90^ Hydrolyzable tannins are a mixture of pyrogallol, ellagic acid and esters of sugars with gallic and digallic acids,^91^ with MW generally below 3 kDa. These later species are higher priced tannins and are limited in their worldwide production, at least compared with condensed tannins, which are extracted in larger amounts, up to 0.2 Mt per year.^89^ Condensed tannins have considerably lower water solubility, particularly in mildly acidic conditions, and their MW is generally below 20 kDa.^92^ Owing to their heterogeneous electron density and functional group distribution, tannins have the most versatile affinity for supramolecular interactions compared with other biobased polymeric and oligomeric compounds.^93,94^

#### 2.1.3 Proteins

As is the case with starch, the role of protein as food source, significantly impact their potential for technological uses. For the development of sustainable materials, the main proteins are those extracted as byproducts or side streams in agricultural processes. They include soy proteins, which are readily available from the soybean oil industry that produces upward of 100 Mt soybean products per year, 30% of which can be isolated as soy protein isolates (SPI).^95,96^ Soy proteins are mainly globular and consist of 90% SPI. Another protein of technological interest is whey protein, which can be extracted as concentrate from wastewaters of the dairy industry.^97^ A low-cost and readily available protein of interest is collagen and its associated hydrolysate, that is, gelatin, which can be thermo-processed (see Section 5.3.3). The annual production of gelatin as side-streams from animal processing is estimated at around 0.4 Mt,^98,99^ for main uses in the food industry.^100,101^ In some cases, gelatin is underutilized due to logistical issues, as is for instance the case of fish gelatin.^102^ Silks and the constructs of silk fibroin are additional structural proteins that endow mechanical strength in architectures rich in β-sheets structures.^103^ Silk fibroin represents the main proteinaceous building block, with the potential to reach outstanding mechanical properties. Therefore, it is of high interest as a sustainable building block and understanding its use will unlock the optimal implementation of other proteins as well. An important endeavor to survey with regard to sustainable production of high-performance proteins are biotechnological, downstream, processes. While their yield is generally low and the operating costs are relatively high, biotechnologically engineered proteins are bound to play an important role in the future of sustainable materials, as hinted by recent indications.^104^

Proteins, being linear polyamides of mainly 20 amino acids, have adaptable chemical structures, as well as a folded tertiary and complex quaternary structures, as in the assemblies of silk fibers. The interactions of proteins can be very precisely controlled in such cases to yield very high specificity, leading to constructs of high mechanical strength and toughness.^105−107^ Because of the high diversity of functional groups, almost ubiquitous to proteins, proteins adsorb on most interfaces.^108,109^ However, in the bulk, proteins interact very specifically with other proteins, or with given secondary (macro)molecules carrying functional groups that can be oriented in three dimensions.^110,111^ Most proteins are amphiphilic, with an outer charge proportional to their isoelectric points, which depend mainly on the numbers of histidine, lysine, aspartic acid, glutamic acid, and tyrosine residues. They interact with most biopolymeric interfaces, although with varying affinities. For instance, when evaluating electrostatic interactions, proteins can bear a range of isoelectric points depending on their compositions. In addition, they may have highly hydrated shells due to glycan substitution.^112^ Interestingly, compared with other biopolymers, biomass-derived proteins possess a relatively higher structural homogeneity, as their polydispersity in size and in morphology is relatively low.

A range of interactions is available, which is intimately linked with the natural structures they are obtained from. Significant progress in biomimicry can be anticipated as our understanding of natural polymers and their structures is improved and the extraction processes are optimized to obtain structures that are closer to the natural, native composites.

### 2.2 Native Biointeractions

In natural biosynthesized materials, the biopolymers described previously are intertwined hierarchically by a combination of noncovalent and covalent interactions^113,114^ that increase in complexity, going from the molecular to the macroscale. Attention must be given to the inherent inter- and intrapolymeric interactions and those between the assemblies, so to adjust the chemical and mechanical treatments to be used for isolation from given biomass sources. Such efforts can be instrumental in reengineering the building blocks toward bioinspired architectures. In this section, we discuss the most relevant chemical interactions and the relationship between molecular, supramolecular, and morphological topology within higher organisms (e.g., plants and animals). This includes matching interactions between biopolymers, for instance lignin and hemicellulose, proteins, and inorganics, etc. We demonstrate how biomass deconstruction is closely related to the hierarchical structure of the isolated biopolymer.

#### 2.2.1 Multicomponent Interactions

**Figure 4:**
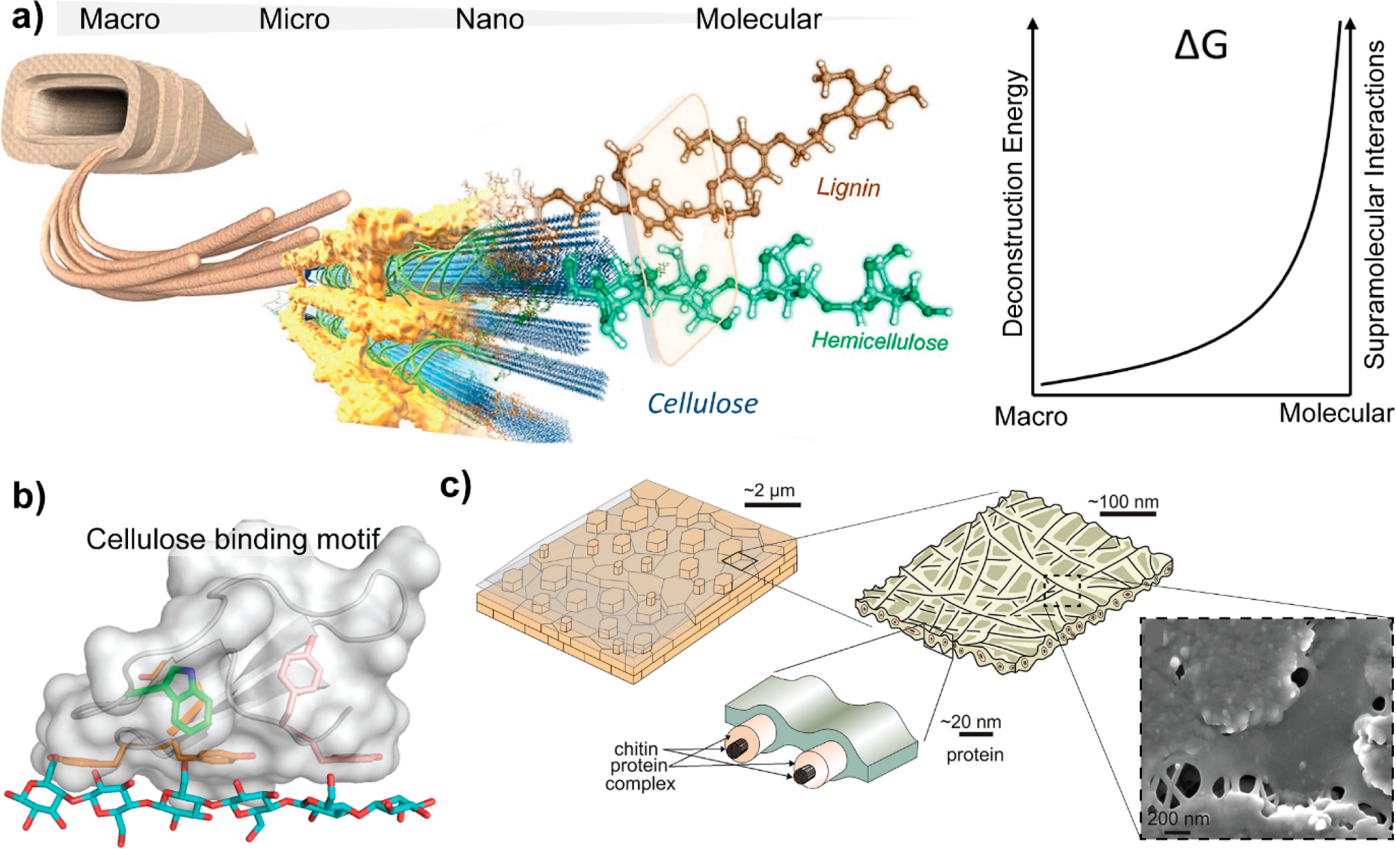
Illustration of some heterogeneous interactions and their effect in mechanical defibrillation. (a-Left) Cellulose, hemicellulose, and lignin organization within plant cell walls. Adapted with permission from ref (138). Copyright 2018 The Authors under Creative Commons CC BY license, https://www.nature.com/articles/s41598-018-24328-9#rightslink. (a-Right) Energy of deconstruction of biological architectures into nanoscale elements, highlighting optimal energy expenditure at intermediate scales. (b) Cellulose binding motif of endoglucanase (EGL1). Reprinted with permission from ref (139). Copyright 2017 The Authors. (c-left) Multiscale interaction in seashells between calcite, proteins, and chitin as revealed by scanning electron microscopy. (c-right) Chitin fibrils bundles within the protein matrix between inorganic plates.^132^ Adapted with permission from ref (132). Copyright 2015 The Authors.

For any monolithic or nanocomposite system, deconstruction in the macromolecular or nanoscale requires less energy and chemical reagents compared to bottom-up synthesis (Figure 4a).^114,115^ The extraction of biopolymers and their constructs from biomass feedstocks involves principally deconstruction by disruption of the weaker bonds; generally resulting from heterogeneous interactions. For instance, the stronger and more crystalline cellulose nanofibrils are the last to be separated from wood under high-energy treatments.^116,117^ Multiscaled hierarchical heterogeneous associations result in highly tortuous, multiscaled fracture dissipation mechanisms in biological materials, making them both tough and strong. For each scale, an incrementally higher energy is required to break bonds. When further deconstruction is required, higher energy input must be provided, for instance in the case of mechanical defibrillation used to obtain nanocelluloses. Finally, to dissolve cellulose, very specific solvents are required. Within each scale, the stronger interactions result in a higher energy cost for deconstruction (Figure 4b). For each biomass source, specific interactions exist between different classes of biopolymers as well as between biopolymers and other components. Generally, the interaction strength scales upward with the decreasing characteristic size, whereby the strongest building blocks are observed at the nanoscale (Figure 4c). One of the most well-known set of heterogeneous interactions are those between cellulose, lignin, and hemicelluloses (Figure 4a). The combination of these biopolymers results in a broad range of strength, toughness, and water interactions associated with all plants.^56,57,118^ The exact nature of these interactions remains largely unknown. One of the biggest hurdles in related efforts is that the process of isolation of lignins and hemicelluloses significantly affect their structures compared to the native state and because information about the respective organization in the biological matrix is lost in the process. However, it is expected that each of these components can interact favorably with each other. Hemicelluloses and cellulose, for instance, interact so strongly^60^ that chemical or mechanical defibrillation do not entirely overcome, such forces, thus leading to hemicelluloses binding elementary fibrils from wood into fibrillar aggregates.^119−121^ In the same vein, gums (Section 2.1) have very similar properties and functional groups as hemicelluloses and their interactions with cellulose will highly depend on the specific side groups present on the branched polysaccharides as well as their topology.^122^ Furthermore, the strength of interactions in biological systems depends highly on the presence of ions or water as they affect the surface potential and cross-linking ability.^123^

The main classes of supramolecular complexes involve proteins. Across biostructures, from plants to higher animals, their inherent interactions are enabled by the broad range of functional groups available in amino acids combined with binding motifs induced by the quaternary structures of proteins. Regarding the latter, the unique transition from secondary α-helices to β-sheet is one of the key mechanisms involved in silk’s crystalline structure.^16^ This type of homogeneous protein–protein interactions are also associated with the growth of amyloid fibrils as discussed in the following sections.

Proteins can undergo both specific binding^124^ and, less specific, aggregation.^78,125^ A typical example of protein heterogeneous aggregation is that resulting from the interactions between proteins and tannins,^126^ as shown in the early stages of the tanning process. Another important protein complex is that with minerals, exemplified by strong and tough materials such as bones or mineralized sea animals.^127,128^ In the case of bones, essentially collagen and phosphoproteins interact noncovalently with minerals.^129^ For marine shells such as those of crustaceans or nacre, the soft matrix of chitin and protein is bound to the mineral layer with specific protein-binding layers (Figure 4c).^130−132^ The molecular-level interactions between proteins and hard mineral components are the most important contribution to the mechanical toughness in bionanocomposites.^127,133^ In catalytic proteins, that is, enzymes, interactions with biological surfaces are most relevant for their specificity via supramolecular binding. The most common case relevant to biobased materials is found in mushroom, where cellulose-binding motifs are associated with cellulases, enabling the coupling of the mushroom’s metabolism to that of plants.^134,135^

In the case of chitin-containing species such as crustaceans, the interactions between individual components are extremely strong. This is also the case of hemicellulose and cellulose where mechanical treatment is not able overcome their interactions, eventually demanding rather harsh chemical treatments to yield more pure cellulosic nanofibers.^121,136^ Importantly, for all biomass types, the interactions with water and ions significantly influence the strength of the bonds and therefore, for effective deconstruction, the removal of cross-linking ions is as important as maintaining partial moisture. This is due to the association of amorphous domains, for instance associated with hemicelluloses, where water competes with the interactions between the macromolecules.^10,137^ Overall, heterogeneous interactions affect the material properties of bionanocomposites in their natural state as well as the recalcitrance for biopolymer extraction. This highlights the importance of gaining knowledge on the interactions that exist in biomass and the effect of solvents, particularly water.

#### 2.2.2 Self-Interactions

**Figure 5:**
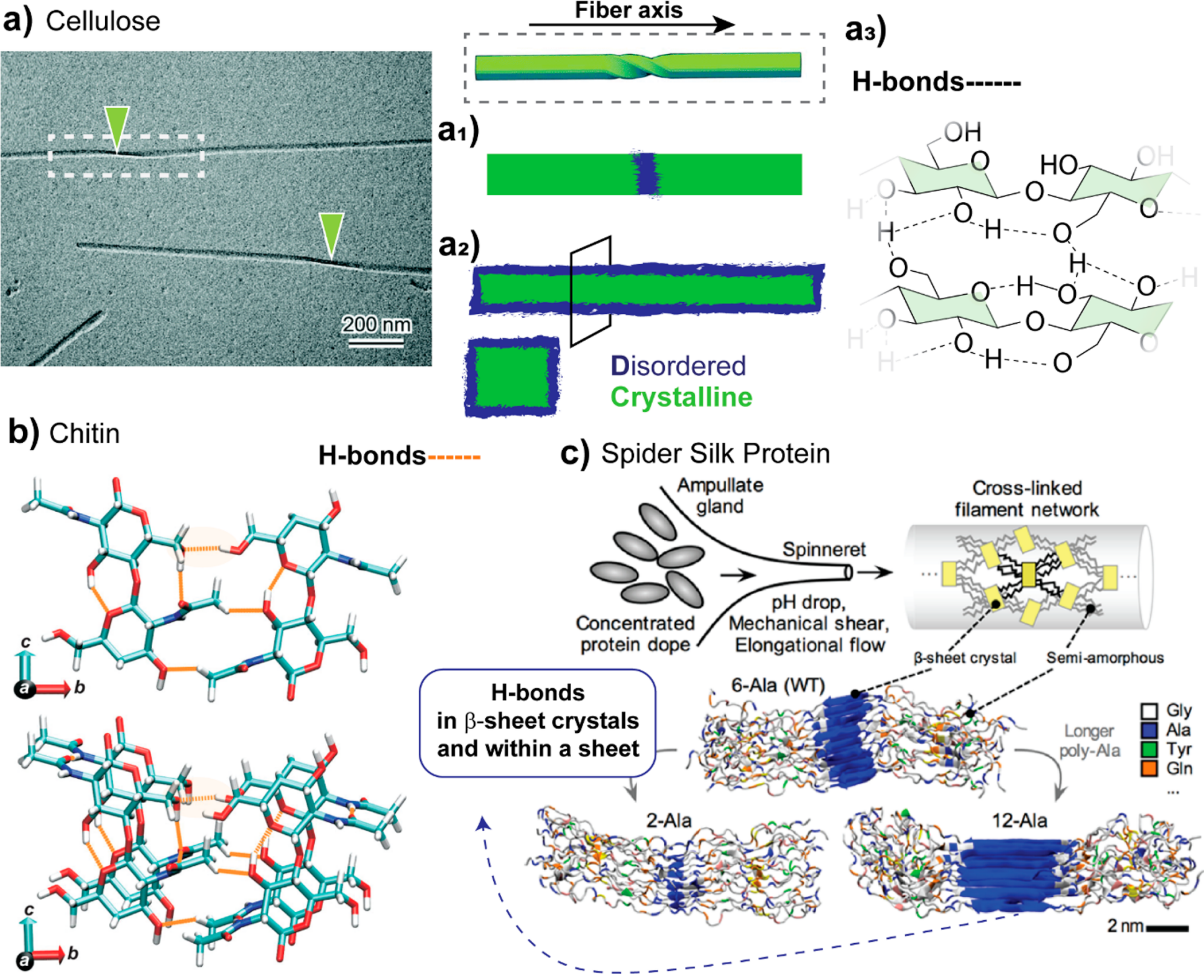
Self-interactions of abundant natural biopolymers such as (a) cellulose,^153^ (b) chitin,^154,155^ and (c) spider silk proteins.^156^ (a) Cellulose colloids have been shown to possess defect regions along the main axis, which are proved to be amorphous. Reprinted with permission from ref (145). Copyright 2019 The Royal Society of Chemistry. (a1, a2) Two most accepted models for the arrangement of crystalline (ordered) and amorphous (disordered) domains in cellulose colloids. (a3) Configuration and distribution of hydrogen bonding within the cellulose macromolecular structure. Hierarchically structured fiber analogues (in all shown examples) are the result of strong/weak hydrogen bonding to form crystallites (strong hydrogen bonds) and noncrystalline regions (weak hydrogen bonds). Extensive hydrogen bonding is evident between multiple OH groups present in cellulose/chitin macromolecular backbone, and between polypeptides rich in the alanine (Ala) amino acid of silk proteins. (b) Chitin molecular model is reprinted with permission from ref (149). Copyright 2012 John Wiley and Sons. (c) For silk protein fibers, the chemical/mechanical processing in the ampullate gland is highly relevant to the structural units of crystalline and semiamorphous features of the fibers. Adapted with permission from ref (156). Copyright 2012 John Wiley and Sons.

Native homogeneous biopolymer interactions spring from the molecular level, in elementary fibrillar/crystalline entities, up to macroscale fiber networks. Hydrogen bonding is ubiquitous in biomass-sourced polymers such as cellulose,^140^ chitin,^141^ and glycine-alanine/polyalanine-rich silk proteins.^103^ As an example, higher-order semicrystalline structures from cellulose possess ordered or more-ordered and disordered or less-ordered substructures that are stabilized by hydrogen bonds and hydrophobic vdW interactions, forming the elementary nanofibrils (Figure 5a). Disordered regions, often referred to as amorphous domains, organize the fibers into bundles and fibrillar structures oriented along the fiber’s axis. As stated earlier, biopolymer superior mechanical performance stems from specific interactions, which start at the molecular level and work their way to the nanoscale, in high-order hierarchical constructs finally forming the bulk materials. The structure of biopolymers is changed upon extraction and their initial cohesive interactions need to be rebuilt depending on the severity of the process.

The distribution of ordered and disordered regions is principally analogous to semicrystalline polymers with disordered regions distributed between ordered, crystalline regions along the nanofibrils (Figure 5a1).^142^ In contrast to the common view of the occurrence of alternating ordered and disordered structures along nanofibrils, a model of cellulose was suggested recently from solid-state NMR and suggesting that the disordered regions of cellulose are located at the surface of the nanofibril, surrounding the ordered, crystalline cellulose (Figure 5a2).^143^ Statistical analyses of the kink angle (“defects” or sharp bends in between straight CNF segments) distribution along individualized CNF from wood also seem to indicate that the defects are not disordered or amorphous.^144^ In addition, X-ray diffraction analysis of these “defect” regions in case of tunicate CNFs and CNCs have been shown to be ordered, crystalline.^145,146^

Ordered nanoconfined building blocks,^113^ nanocrystals, are the result of multivalent hydrogen bonding that maximizes material strength, stiffness, and toughness of silk^103^ and crystalline cellulose^147^ to name a few. Cellulose forms a network of inter/intramolecular interactions between individual polymer chains at different scales, and the dependence between interaction strength and size of the building blocks is well demonstrated from the mechanical properties measured in cellulose films made from either micron- or nanosized fibrils.^148^

On a molecular level, the highly regular and linear homopolymer chain of β(1 → 4)-linked d-glucose rings^149^ gives chain rigidity to cellulose as the glycosidic linkages on both sides of the ring are equatorially directed. In addition to hydrogen bonding, vdW forces have been described as main contributing force developing cohesion of cellulose chains.^150^ These vdW forces originate from hydrogen and carbon atoms positioning perpendicular to the ring plane and give the cellulose macromolecule also hydrophobic properties, which play an important role in the interaction of cellulose at interfaces (see Section 4.4). Native cellulose I occurs in two slightly different subpolymorphisms, Iα and Iβ, in which cellulose chains are packed together in a parallel manner, forming unique hydrogen bonded networks structures. The ratio of these suballomorphs relates to the origin of cellulose. Wood-based cellulose is rich in the Iβ-form and a large contribution of the Iα form is found, for example, in bacterial cellulose (BC) and algae.^151,152^

Cellulose I undergoes transition into the thermodynamically more stable polymorph, regenerated cellulose, that is, cellulose II, via mercerization in caustic alkaline solution^157^ or dissolution in a cellulose solvent and subsequent regeneration with an antisolvent. Cellulose II crystalline structure is less commonly found, present in nature in marine algae and bacteria,^158^ and is stable because of extensive intersheet hydrogen bonding. Cellulose III and IV polymorphs result from different cellulose processing. Ammonia or amine solution treatment turns native cellulose I and cellulose II into crystalline cellulose III_I_ and III_II_ forms, respectively, through a complex structure. Hot thermal treatment with stretching forms cellulose IV_I_ and IV_II_ structures from cellulose I or II, respectively.^159^

X-ray diffraction (XRD) is commonly used to determine the crystal type and crystallinity of different cellulose polymorphs.^160^ Crystallinity values should be only compared for a single polymorph or crystal structure, due to their inherent differences.

The value of crystallinity index varies significantly depending on the measuring technics, and it depends mainly on the crystallite size and the disorder in the sample.^161^ Varying crystal forms of cellulose (I–IV) exist in nanocelluloses. Different treatments used in wood pulp production, to prepare CNCs or CNFs, alter their crystal (cellulose I and II).^162,163^ Altered properties can be obtained from CNFs with a cellulose II crystalline structure, as demonstrated by the lower elastic modulus (8.6 GPa) compared to that of cellulose I (11.8 GPa). A higher toughness and fracture strain is noted as well (13.6% for cellulose II compared to 7.5% for cellulose I).^164^

The skeletal structures of marine species containing chitin demonstrate exceptionally high mechanical performance.^43^ Chitin exists in three crystalline forms, α, β and γ, which differ in the orientation and packing of the chitin chains. In crustaceans and insects, chitin is most commonly in the α-form (Figure 5b). In mollusks, chitin exists in the β-crystalline form. α-chitin with antiparallel chain packing has a higher degree of hydrogen bonding that makes the structure more thermodynamically stable. The γ-form of chitin is a variant of the α-form, with a mixture of β-forms.^165^ Inter- and intramolecular hydrogen bonding between chitin chains stabilize the α-form; interestingly, it differs from cellulose where additionally vdW attractions are required to maintain the crystalline structure.^166^

Studies on lignin interactions have aimed at understanding the lignification process of the cell wall and for efficient delignification for commercial purposes.^167^ Research efforts have been dedicated in nanoparticle preparation with controlled lignin particle size and morphology.^168,169^ Technical lignins are polydisperse and carry weekly acidic groups and disassociate in alkaline media.^84^ Lignin aggregation is dependent on the nature of lignin sample and triggered by the formation of nuclei, which may be present from the beginning or formed due to changes in the solution conditions. Experimentally observed phase behavior of colloidal Kraft lignin can be described reasonably well by a theoretical approach derived from the DLVO theory.^170^ Nonhomogeneous charge distribution as well as the molecular stiffness of the aromatic groups in lignins suggest π–π stacking of lignin molecules, which plays an important role in their self-assembly into clusters and particles. It has been shown that such stacking can be favored when the particles are formed in a solvent exchange process, in the absence of alkali salts. In such cases, better particle stability has been noted compared to that observed for particles formed, for example, by precipitation.^171^

Proteinaceous spider silk fiber possesses high tensile strength of 1–2 GPa and 50–60% strain at failure.^113^ Nanoconfined crystal formation and highly ordered fibril structures built from strong hydrogen bonding are the reason for the outstanding mechanical properties (Figure 5c). The optimal silk β sheet crystal size that yield these properties is 2–5 nm.^103,172^ Although the strength of hydrogen bonding is lower than covalent bonds or ionic interactions,^173^ hydrogen bond clustering and its ability to break and reform due to the cooperative nature are highly effective, as shown by the properties of spider silk fibers. Overall, the interactions between biopolymers discussed in this chapter are crucial for material research. These principles are already harnessed in the assembly and consolidation of structures that will play prominent roles in the future design of sustainable, high-performance materials.

## 3 Deconstruction and Modification of Biopolymers and Biocolloids

In contrast to conventional synthetic polymers, natural biopolymers are mainly extracted from biomass via top-down approaches. The focus of this section is on the isolation of hierarchically structured biopolymers from biomass (Section 3.1), their deconstruction into biocolloids and chemical modification to ease the deconstruction and tailoring the final properties of the colloids. Most efforts have so far focused on the isolation of cellulose fibers from tree-based resources, that is, wood pulp fibers.^27^ As this concept is accompanied by the most mature practical and theoretical knowledge, we will use wood biomass to explain the main processes and challenges in biomass conversion into biopolymers and high-performance colloids. Challenges related to isolation, deconstruction and modification of plant-fibers are mostly associated with their multiscale hierarchical structure and the strong matrix interactions at different scales, and this is very much related to other important bioresources, for example, crustacean shells, insect cuticle and animal tissues.^113^

### 3.1 Isolation of Biopolymers from Hierarchically Structured Biomatrices

**Figure 6:**
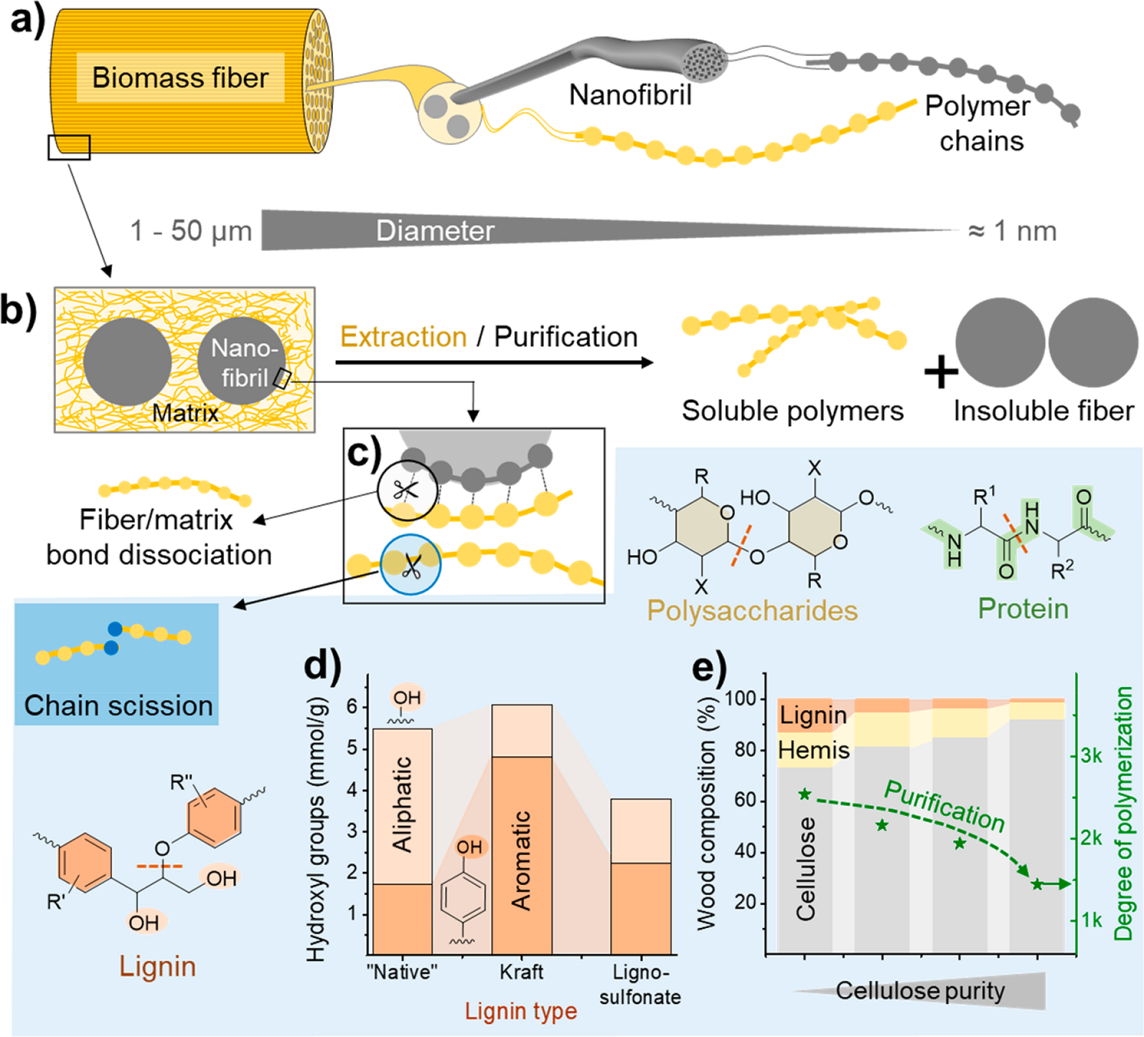
Effect of multiscale hierarchy on the extraction process and the properties of isolated biopolymers. (a) General structure of biopolymers, such as cellulose, chitin, and fibrous proteins based on assembled nanofibrils. (b) Nanofibrils are embedded into a matrix of disordered biopolymers and other components. The biopolymer of interest can be either isolated by removal of the matrix (purification) to yield a solid biopolymer fiber or by selective solubilization from the fiber matrix (extraction). (c) These processes are based on the disruption of the self- and intercomponent interactions with the matrix and accompanied by chain scission (polymer degradation) caused by hydrolytic processes. The most common chain scission reactions are indicated for selected biopolymers. During purification of plant fibers, that is, typically by pulping, (d) the native structure of noncellulosic biopolymers is altered, as shown in the case of lignin. (e) Recalcitrance of plant fibers requires harsh pulping conditions, especially to obtain high-purity cellulose, causing polymer degradation and reduction of cellulose molecular weight. Panels d and e were drawn from data published by Korntner et al.^203^ and Fang et al.,^203,235^ respectively.

Biopolymers are isolated by (1) extraction through solubilization of the respective biopolymer and its subsequent precipitation, or (2) purification of the biomass fiber to isolate a solid, generally, fibrous biopolymer through solubilization of its surrounding matrix polymers (Figure 6b). In these processes, the multiscale hierarchy and matrix interactions in the biomass determine their recalcitrance and is also a reason for the high mechanical strength of fibrous biopolymers^23^ including cellulose,^174^ chitin,^175^ collagen,^176^ keratin,^177,178^ and fibroin.^179,180^ Cellulose, chitin,^181^ and fibrous proteins, such as silk fibroin^182^ and fibrous collagen in leather,^183^ are traditionally isolated by solubilization of the surrounding matrix components (Route 2). In contrast, other polysaccharides, such as xylan,^184^ pectin,^185^ alginate,^186^ and hyaluronic acid,^187^ as well as proteins, including soy bean proteins,^188^ collagen,^189,190^ and gelatin,^191,192^ are mainly isolated through solubilization of these biopolymers from their matrices (Route 1). Figure 6a illustrates schematically the structure of fibrous biopolymers, exemplified with cellulose and based on the elementary nanofibrils. These fibrils are tightly bound in a matrix,^193^ forming bundles^194^ that scale-up to micron-sized fibers. In this structure (Figure 6b) and due to their disordered state, the matrix polymers feature higher chemical accessibility than the elementary nanofibrils^195^ or higher reactivity due to their functional groups, as is the case of aromatic hydroxyls of lignin.^196^ At the largest scale, in the obtention of pulp fibers, pulping is the first purification process, in which the matrix around cellulose is removed and polysaccharide-lignin associations are cleaved.^197^ The most common pulping process, Kraft pulping,^27^ is a chemical process that uses aqueous solutions of sodium hydroxide and sodium sulfide.^198^ Apart from chemical pulping, also mechanical pulping is utilized, although less frequently.^27^ In chemical pulping and other isolation processes, solubilization of the matrix polymers is mainly based on the dissociation of matrix bonds and the increase of polymer solubility upon degradation (Figure 6c) or chemical modification.^27^ Therefore, the chemical structure of these rather amorphous polymers is strongly altered during the pulping process. Industrial lignins, such as Kraft lignin and lignosulfonates from Kraft and sulfite pulping processes, respectively, contrast with milled wood lignin, which is considered to be closer to the native lignin structure.^199^ Kraft lignin has a very different structure due to bond scission, condensation, and other side reactions occurring during pulping, wherein the generated hydrogen sulfide ions in alkaline medium fragment and modify the intrinsic lignin structure, increasing its solubility in the cooking medium.^200,201^ The isolated lignin structure is highly condensed and contains a significantly higher amount of aromatic hydroxyl groups than its native analogue, and a low amount of residual β-O-4 bonds (Figure 6d). Similarly, the chemical structure of lignosulfonates differs significantly due to side-reactions during the pulping process and incorporates sulfonate groups.^202,203^ More details on typical and known lignin chemical reactions during pulping can be found in recent reviews.^201^ Because of the strong biomatrix interactions, the isolation of high-purity cellulose is usually accompanied by harsh pulping conditions, reducing significantly the MW of the cellulose and causing chain scission (Figure 6e). Cellulose fibers, of 90% purity or higher, are referred to as dissolving-grade pulp, the starting material for the production of soluble cellulose ethers or esters,^27^ but also used as precursor for functional biocolloids.^163,204,205^

The cellulose composition can be tuned to reach a desired composition.^27^ Selective removal of matrix biopolymers, such as lignin or hemicellulose, has been utilized to increase porosity in nano- and microscale, which can be exploited to control the wood properties and to engineer functional, high-performance materials.^206^ Moreover residual amounts of hemicellulose and lignin are important in paper products to obtain adequate mechanical properties,^207^ and to determine the surface properties of extracted biocolloids.^208,209^ It has been shown that never-dried hemicellulose-rich cellulose, that is, holocellulose, obtained from treatment with NaClO_2_^210^ or peracetic acid,^211,212^ can be deconstructed into CNF with higher energy-efficiency compared to celluloses with low hemicellulose content. Although most cellulose materials are still prepared from wood, preparation from nonwoody biomass is becoming popular as it represents a sustainable and low-cost alternative. Prominent examples are, for example, straw or sugar cane bagasse, featuring a less recalcitrant structure than wood. They are mostly processed by soda pulping, which is based on NaOH as active ingredient.^213^ As both Kraft and soda processes are use alkaline conditions, the resulting fractions of hemicelluloses and lignins are similar in structure, albeit sulfur-free in the case of soda pulping.^214^ Apart from these well-known processes, new strategies are being developed to enable isolation of lignins, hemicelluloses, and other polysaccharides in efforts to preserve their structure and to valorize the biomolecule in suitable applications.^215−217^ Such developments align with the scope of efficient biorefineries that aim to replace oil-based feedstocks in chemicals, solvents, material and bioproduct development.^218^ These biorefinery consider new pulping chemicals, such as imidazolium-based ionic liquids (ILs), solid maleic acid and new green chemistries to fractionate the biomass while allowing chemical functionalization.^216,219,220^ Thereby, the isolation of a colloidal fraction is achieved to produce high-performance materials. In addition, biochemical platforms, such as enzyme-based biorefineries are being developed to supply bulk chemicals and materials from lignocelluloses; as well as nanocelluloses.^221,222^ Recently, hydrothermal treatments have been proposed to valorize carbohydrate fractions, placing emphasis on the extraction of valuable lignin fractions.^217,223,224^

Similar to cellulose fibers, chitin is hierarchically structured and based on as nanofibrils assembled in a helicoid arrangement in the exoskeleton of animals.^113^ Chitin is also a main components of fungi cell walls,^225,226^ with significant differences noted for the chemical structure of chitin from fungi and animals. In fungi, chitin is chemically linked to glucans and is the primary component of the cell walls and, in most cases, is embedded in a hemicellulose matrix,^227^ which contrasts with chitins in crustaceans that are combined with proteins and inorganic carbonates. It has been demonstrated that a major part of the protein fraction is strongly bound to chitin, in the form of covalent chitin-protein links, resulting from interactions with aspartic acid and histidine amino acids, which pose a challenge for the extraction of pure chitin.^228^ In general, plant- and animal-based chitin are both extracted by sequential acidic and alkaline treatments to remove protein, polysaccharides, and inorganic components.^226,229^ The acidic extraction is especially relevant in the case of crustacean-based chitin to remove the inorganic carbonates, but it is not an obligatory step in the extraction of chitin from fungi.^181^ Because of the strong interactions of chitin with proteins, similarly to the heterogeneous interactions of hemicelluloses and cellulose in plants, it is very challenging to remove the protein fraction without leaving some residual protein bound to the chitin. Because of the partial hydrolysis of the chitin acetyl groups at the fiber surface and the residual glucan content in fungi-derived chitin, the amine content becomes an important factor influencing chitin properties.^230,231^

Both silk fibroin and collagen are examples for hierarchically structured protein fibers. Silk fibroin is generally extracted from silk cocoons in a heterogeneous purification process through removal of sericin and other impurities using caustic washing, that is, degumming.^182^ The extraction of collagen fiber from animal tissue is mainly associated with leather-making processes.^183^ The hierarchical structures of collagen and silk fibroin fibers have been utilized as template to obtain functional nanostructured materials^232−234^ but contrast to plant-based fibers since they are hardly used as direct precursor of nanofibrous building blocks.

Overall, it is important to consider the severity of biomass extraction and the extent of structural alterations in the biopolymers, which significantly affect their chemical and physical properties. Since the biopolymer chemistry plays a dominant role as well in the deconstruction of hierarchically structured biomass into biocolloids, a general understanding of these concepts is required.

### 3.2 Chemical Modification of Biopolymers and Deconstruction into Biocolloids

#### 3.2.1 Biopolymer Reactivity and Chemical, Structure, and Mechanical Property Relationships

**Figure 7:**
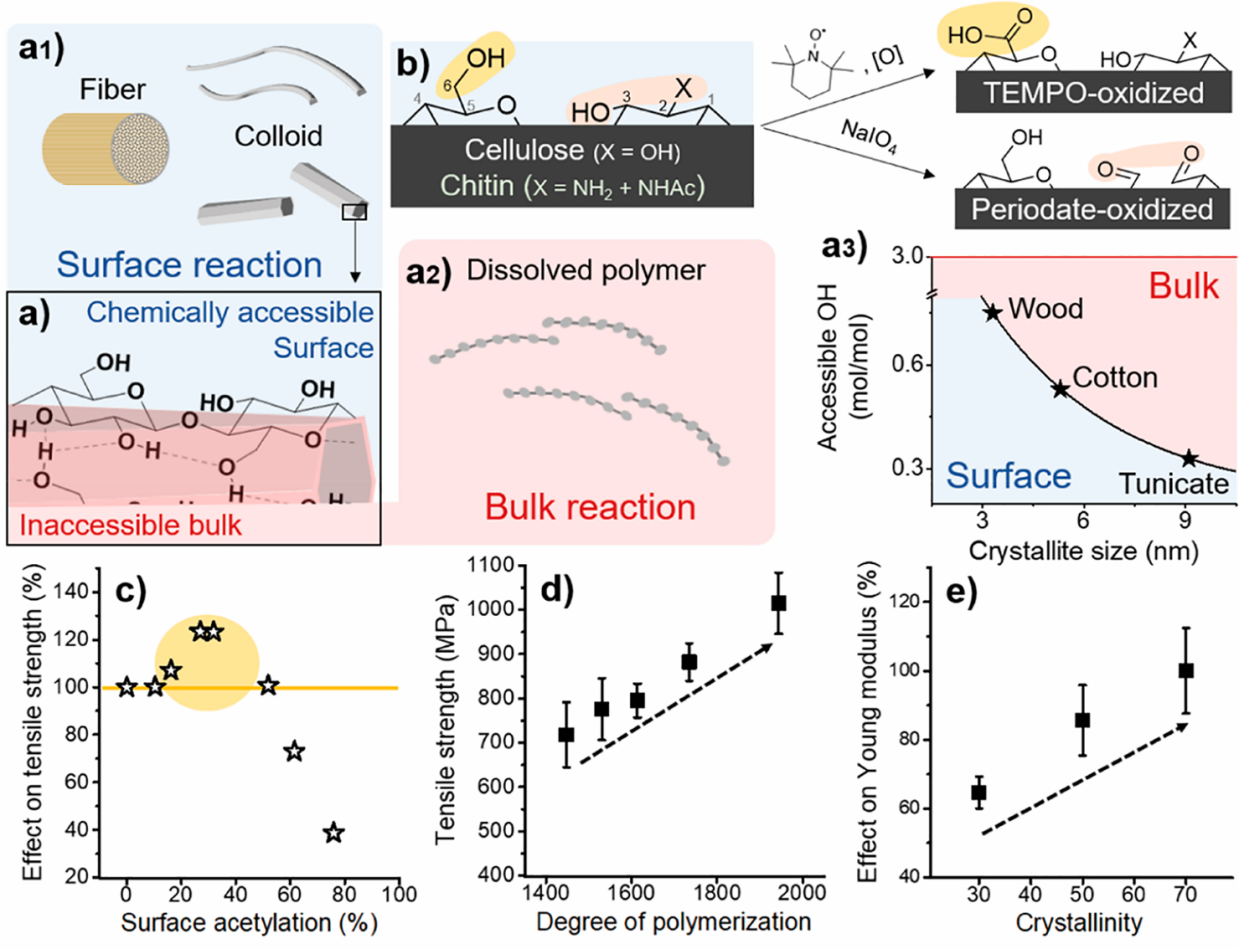
For the modification of hierarchically structured biopolymers, it is important to differentiate between heterogeneous surface reactions of fibers and colloids (a1), and homogeneous bulk reactions of dissolved biopolymers (a2). In surface reactions only the chemically accessible regions (blue shades) are modified while preserving the inaccessible and crystalline domains (red shades) (a). Full surface modification is defined as complete modification of alternating primary C6-OH and secondary C2,C3-OHs at the surface, that is, the number of chemically accessible hydroxyls. (a3) This number depends on the crystallite size of cellulose, limiting the number of functional groups that can be introduced under heterogeneous conditions. (b) Regioselective surface modification is often associated with TEMPO- and periodate-oxidation and can be performed on cellulose, chitin and other polysaccharides in water as reaction medium, yielding C6-carboxylated or C2,C3-dialdehyde derivatives. (c) Control of the extent of chemical modification is essential, as moderate surface-modification by acetylation increases the tensile strength of cellulose fibers, whereas it drops for higher acetylation degrees. Modification of biopolymer fibers should be mild and avoid the reduction of the molecular weight (d) or crystallinity (e) of the products, as both properties influence significantly the mechanical performance of processed materials. Plots a3, c, d, and e were drawn from literature data by Okita et al.,^236^ Aiken et al.,^248^ Zhiqiang et al.,^235^ and Ottesen et al.,^251^ respectively.

Hierarchically structured fibers are composed of elementary nanofibrils, which feature a chemically accessible surface regions and an inaccessible crystalline core domain, as shown schematically in Figure 7a. One must hence distinguish between heterogeneous surface reactions (Figure 7a1), which are confined to the chemically accessible regions of fibers or colloids, and homogeneous bulk reactions (Figure 7a2). The latter usually result in soluble biopolymers, but also water-insoluble fibers can be solubilized in special solvents such as ILs (see Section 3.3). The amount of accessible hydroxyl groups in cellulose varies by species and is evidently dependent on the crystallite size and the elementary fibril dimension (Figure 7a3).^40,236^ In case of cellulose, three different surface hydroxyl groups are available, the primary C6-OH as well as the secondary C2-OH and C3-OH (Figure 7b). In contrast, chitin features only C6-OH and C3-OH groups and an acetylated amine group, C2-NH_2_. During purification and processing of chitin, partial deacetylation occurs,^237^ but this usually is not affecting the structural properties of chitin. More severe, hydrolytic conditions yield water-soluble chitosan, for example, if the degree of deacetylation is above ∼50%.^238^

Controlled chemical modification of polysaccharides is commonly achieved by regioselective oxidation (Figure 7b). The most utilized is the selective oxidation of C6-OH of cellulose^239^ or chitin,^240^ commonly mediated by the 2,2,6,6-tetramethylpiperidine-1-oxyl (TEMPO) radical in combination with chlorine oxidizing agents. Alternatively, similar reactivity is achieved by oxidation with ammonium persulfate.^241^ Selective oxidation of C2- and C3-OH (or C2-NH_2_ and C3-OH in the case of chitosan or partly hydrolyzed chitin) and cleavage of C2–C3 carbon bond can be achieved by reaction with sodium periodate, that is, periodate oxidation.^242,243^ Noteworthy, this reaction proceeds only with vicinal diols or amino alcohols (in case of hydrolyzed chitin), peracetylated chitin or 3,6-anhydro-l-galactopyranose units in other polysaccharides are not modified. The aldehyde groups are highly reactive and can be versatilely postmodified, to introduce, for example, electrostatic charges that facilitate deconstruction into fibrils of smaller dimensions, as discussed in Section 3.2.2.2.

Although three hydroxyl groups are in theory chemically accessible at the surface of cellulose fibrils, it has been shown that the C3-OH is in fact hardly accessible due to steric effects and intrachain hydrogen bonding.^174,244,245^ Meaning that modification of C3-OH requires harsher conditions or special treatment, which will eventually affect the crystallinity or MW of cellulose. Consequently, the DS threshold for surface modifications (Figure 7a3), such as acetylation, is actually lower as only C6-OH and C2-OH are chemically accessible under heterogeneous conditions. This is also supported by the literature that discusses an upper esterification limit of approximately 66% in surface esterification.^174,246,247^

The relationship of chemical structure and mechanical properties has been investigated early on, for example, already in 1942 by Aiken and Bletzinger.^248^ They performed mild or low acetylation of cotton and woody cellulose, in the range of surface reactions, without affecting their molar mass (Figure 7c). The resultant tensile strength of cellulose could be significantly increased, by 20–30%, at an optimal surface acetylation, in the range of 30–40%. This is a surprising finding as one would expect a reduction in mechanical strength since less hydroxyl groups are available for hydrogen bonding upon acetylation. This observation is rationalized by the fact that the fibers were fibrillated after acetylation, while the introduction of acetyl groups increased the fibrillation tendency and the effective number of surface hydroxyl groups available in the final fibrillated fiber. If no fibrillation was performed, the mechanical properties were as expected, only hardly affected; similar observations were made for partially acetylated CNF.^249^ Nevertheless, to maintain the intrinsic properties of cellulose, it is important to limit surface modification to rather low levels (below 50%), exceeding this degree of substitution would drastically limit the mechanical properties, as illustrated in the case of acetylation shown in Figure 7c. Considering the higher reactivity of the C6-OH,^250^ one can assume in case of reported optimal degree of surface acetylation (between 30 and 40%), that most acetylation occurs in fact at the primary hydroxyl groups. Consequently, chemical approaches toward regioselective C6-OH modification are of high interest to produce functional materials with high mechanical strength. The crystalline domains will be affected, and polymer degradation will occur above the limit of surface acetylation, thereby reducing the degree of polymerization and crystallinity. As shown in Figure 7c and d, these properties are important with regards to the strength, decreasing crystallinity or MW will ultimately limit the mechanical performance of cellulosic materials.^235,251^ Other properties are also negatively influenced at high degree of substitution, such as the thermal-expansion coefficient and the transparency of nanocellulose, which can be significantly improved by mild surface acetylation but worsen at a higher degree of esterification.^249^

TEMPO- and periodate oxidation have been introduced as regioselective surface reactions, but it is important to consider that the oxidative conditions used usually cause a decrease in MW of the oxidized cellulose.^252,253^ Nevertheless, the TEMPO-mediated oxidation is commonly selective to the surface^236^ and very popular to individualize cellulose fibers into high performance TEMPO-oxidized CNF (TO–CNF).^239^ The periodate oxidation, on the other hand, is principally considered as not highly selective to accessible surfaces and propagates into crystalline domains.^252,254^ This is still under debate since recent approaches report reactions that can be controlled to attack preferentially the amorphous regions.^255^ Taking these facts into account, oxidation of cellulose needs to be controlled to limit its influence on the physical properties, which is in contrast to a recently developed wet esterification method using acyl imidazole that enable highly regioselective esterification of the C6-OH of cellulose while preserving the cellulose MW and crystallinity.^174,205,256,340^

Given the fact that the deconstruction process is affected by surface reactions, understanding their mechanisms and the effect of modification on biopolymer MW and crystallinity is important in the deconstruction of biomass into biocolloids. Moreover, oxidation based on TEMPO and periodate play an important role in the chemical behavior of the biocolloids.

#### 3.2.2 Deconstruction into High Aspect Ratio Nanofibers

Nanocellulose is the most popular class of biocolloids, including (a) high-aspect ratio nanofibers, that is, CNF^116,257^ and BC,^258,259^ (b) lower-aspect ratio colloids, such as rodlike^245,260^ and spherical CNC,^261^ and (c) soft cellulose nanospheres.^262−265^ Although biocolloids based on chitin (ChNF, and ChNC) have been known for more than two decades,^266^ they have been the subject of interest only recently.^47,113,267^ This section, focuses on these biopolymeric nanoparticles produced by top-down approaches, namely, CNF, CNC, ChNF, and ChNC.

##### 3.2.2.1 Production of Native Nanofibers

**Figure 8:**
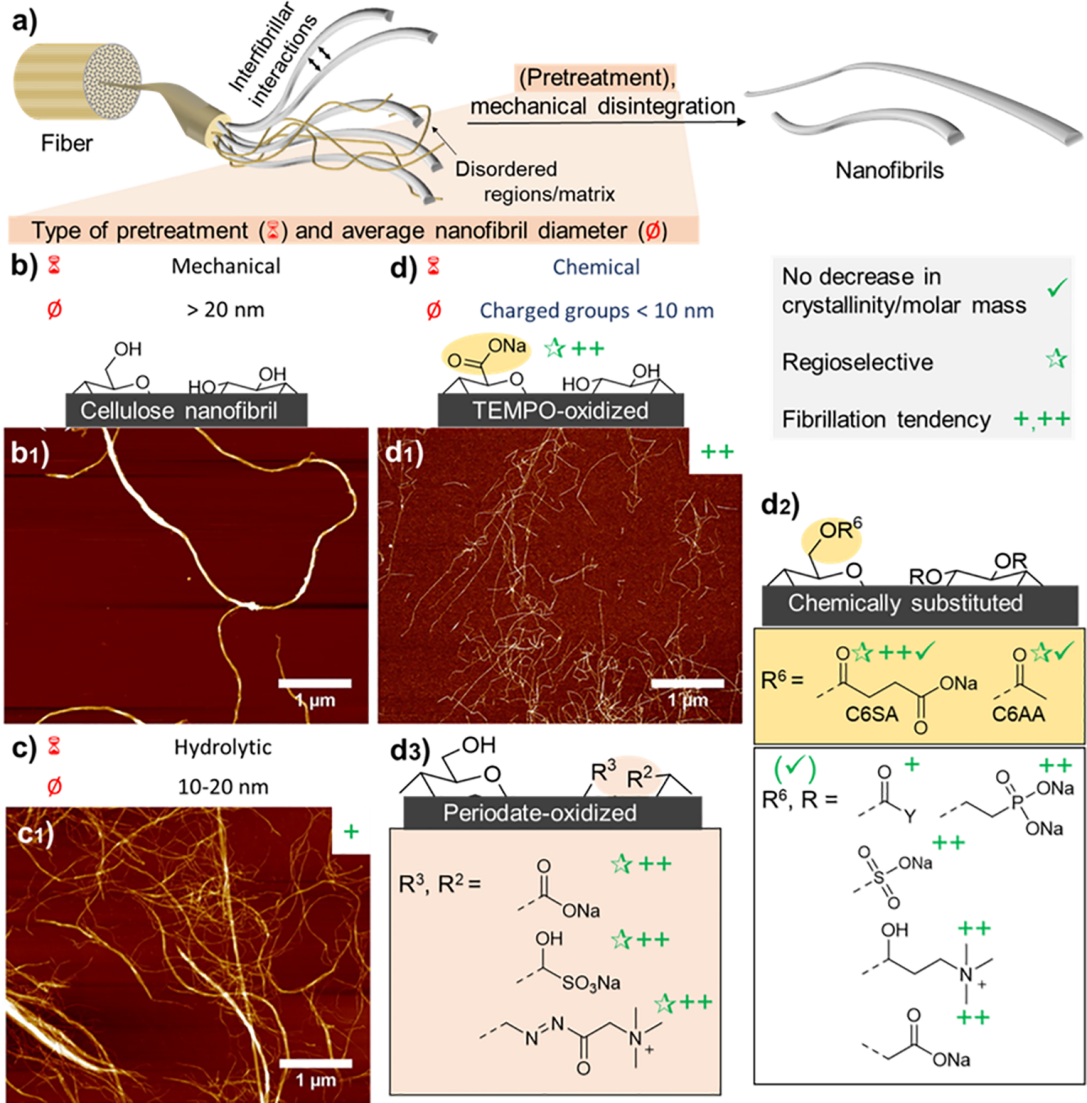
(a) Schematic structure of multiscale structures of polysaccharide bundled as nanofibrils in larger fibers. Surface modification of the fibers occurs on their chemically accessible surface or the surrounding disordered polymer matrix. Mechanical disintegration of the fiber yields individualized nanofibers or bundles. The fibrillation tendency of the fiber can be increased by weakening the interfibrillar interactions through (b) mechanical, (c) physical, or (d) chemical pretreatments. (d2, d3) Surface structure of chemically pretreated cellulose nanofibers is shown and compared, and the pretreatments are classified by (1) their effect on the molecular weight and crystallinity, (2) their regioselectivity and (3) the resulting increase in fibrillation tendency. AFM images are shown for CNF obtained by (b1) mechanical, (c1) hydrolytic mechanical, and (d1) chemo-mechanical treatments. AFM images (b1, c1 and d1) are reprinted with permission from ref (341). Copyright 2014 American Chemical Society.

The size of CNF ranges from that of elementary fibrils to aggregates or clusters with a cross-sectional diameter of up to 100 nm and a length from one to several micrometers;^268,269^ the obtained size depends on the pretreatment used. Similarly, the energy to produce one ton of dry CNF is process-dependent and can range from 2200 to 21900 kWh/t.^270^ One can distinguish between direct mechanical deconstruction, and pretreatments that increase the fibrillation tendency of cellulose by mechanical, hydrolytic, or chemical means (see Figure 8 for an overview). A mechanical disintegration step is in all cases required by (a) high-pressure homogenization,^271−273^ (b) microfluidization,^270,274,275^ (c) microgrinding,^271,276,277^ (d) high-shear blending,^210,212,278^ (e) high-intensity ultrasonication,^279,280^ (f) ball milling,^211,281^ or (g) high-consistency mixing in a kneader^282,283^ or extruder.^284,285^ These mechanical operations incur in the highest energy expenditure and are associated with the highest processing cost for large-scale implementation. Several pretreatments were established to ease the deconstruction or fibrillation into fibrous colloids, which can be distinguished into two main classes based on cellulose’s chemical structure (Figure 8), mainly mechanical or hydrolytic treatments, which preserve the native chemical structure of cellulose and chemical pretreatment (e.g., TEMPO-mediated oxidation), which either introduce repulsive charge or weaken interfibrillar interactions (see Section 3.2.2.2).

The most straightforward method of the former class is a low-energy mechanical pretreatment by, for example, milling, blending, refining, or beating the pulp fibers. These treatments increase the dispersibility of the fibers in water, delaminate their fiber cell wall (internal and external fibrillation) and reduce the size of the final building block (Figure 8b).^270,286,287^ Conventional high-performance CNF is prepared from never-dried pulp.^205^ This is because strong cohesive interactions, such as those that take place upon drying of cellulose (“hornification”), are partly irreversible.^288,289^ Although the fibrillation tendency of once dried pulps is significantly reduced, it can be increased by swelling treatment with, for example, deep-eutectic salts,^290^ ILs,^281^ salt hydrates,^163^ aqueous soda,^291^ or oil-in-water (o/w) emulsions.^292,293^

The fibrillation of cellulose fibers can be further eased through hydrolytic means by using enzymes or acids. Enzymatic pretreatment eases the defibrillation and reduces the nanofiber diameter in comparison to mechanically produced CNF (Figure 8). Usually, enzyme treatments will cause an undesired reduction of MW and fiber length, but this can be circumvented by using defined enzyme mixtures and an optimized process.^287^ Recently, it was shown that enzyme treatments can be conducted at high solid content in a kneader, which enables an energy-efficient access to high-consistency CNF.^283^ In contrast, acidic pretreatments are less favorable as they reduce fiber length and the treated pulps generally undergo lower fibrillation than enzymatically pretreated ones.^294,295^ The fibrillation tendency is decreased upon water removal, but also the cellulose fiber composition plays an important role and can even reduce the influence of drying. Recent efforts have shown that hemicellulose-rich celluloses can be disintegrated into CNF with a higher energy efficiency; this type of CNF is also water-redispersible upon drying.^208,212^ The presence of residual lignin in the fibers does not ease the fiber deconstruction, but lignin-rich CNFs are less hydrophilic than native CNF without requiring chemical modification.^209,296,297^

Compared with CNF, chitin nanofibers can be obtained under lower energy input; this stem mainly from the presence of free amine groups on the surface of chitin. These groups are charged at acidic conditions (p*K*_a_ = 6.2),^52^ introducing cationic charges, which eases the fibrillation into ChNF via electrostatic repulsion.^225^ Moreover, after removal of the matrix components (e.g., inorganics, proteins and other polysaccharides) chitin fibers are already in the nanoscale,^181,298^ which is in contrast to the micron-scaled cellulosic fibers. Further high-intensity fibrillation of protonated chitin yields ChNF with a uniform fibril diameter of 10–20 nm and a degree of acetylation of ∼95%.^298−300^ The electrostatic interactions of chitins can be controlled by varying the degree of acetylation,^301^ and deacetylation under harsh alkaline conditions yields ChNF with smaller width of ∼10 nm.^302,303^ Interestingly, this harsh deacetylation does not affect the bulk properties of chitin, preserving the α-chitin crystallinity.^298,302^ The width of ChNF is in general more uniform than native CNF and can be controlled by the extend of mechanical fibrillation, from a width of 50 nm to smaller sizes and higher aspect ratios.^303,304^ It is important to take into account that compared to native chitin, surface deacetylated chitin is more prone to disintegration into low aspect-ratio colloids.^225,305^ This is explained by the high electrostatic charge of deacetylated ChNF (ζ potentials of up to +105 mV),^304^ which facilitate the disintegration into ChNC. Similar observation have been made for highly charged carboxylated CNF.^306^ The native functionality of the ChNF with partially acetylated amino group renders them less hydrophilic than CNF; explaining their extraordinary performance in the stabilization of foams and emulsions.^304,307,308^

Although protein nanofibers are predominantly obtained by controlled assembly of dissolved proteins,^309,310^ recent efforts have considered the intrinsic hierarchical structure of silk fibroin fibers. These processes are principally related to similar concepts as those introduced in cellulose fiber deconstruction. Initially, nanofibers from spider dragline silk and silkworm fibroin have been isolated by high intensity ultrasonication.^311^ These nanofibers feature a rather heterogeneous diameter of 20–200 nm and appeared as aggregated matter, not dispersible in solution. More recently, a liquid exfoliation method has been established combining partial dissolution and mechanical treatment to isolate silk nanofibers of uniform dimensions with diameters of approximately 20 nm and lengths of up to 300–500 nm.^312^ In addition, methods involving high-pressure homogenizers are currently used to further up-scale the production of silk nanofibers.^313^

##### 3.2.2.2 Production of Nanofibers with Altered Surface Chemistry

Chemical pretreatments in the nanofiber production are mostly based on the introduction of charged functional groups through modification of chemically accessible surface hydroxyl groups.^314^ Among them, the most common TEMPO-mediated oxidation is able to achieve complete surface oxidation (Figure 8d).^236,239^ The introduced carboxyl groups (p*K*_a_ = 3–4)^315,316^ can be deprotonated to introduce repulsive negative charges on cellulose, weakening interfibrillar interactions and strongly enhancing fibrillation. TEMPO-oxidized cellulose can be dispersed by mild disintegration in a high-speed blender to obtain coarse TO–CNFs.^317^ High-energy treatments with high-pressure homogenizer or microfluidizer are required for the individualization into fine TO–CNFs.^239^ TO–CNFs are prepared and stored commonly at <2 wt %, which limits their application due to the high-water content. Recently it was shown that this bottleneck can be overcome by fibrillation of TO–CNFs at high consistency (10 wt %) in an extruder.^285^ C6-carboxylated CNF can be also obtained via oxidation with ammonium persulfate, similar to TEMPO-mediated oxidation; this treatment also partly degrades cellulose reducing its degree of polymerization (DP).^318^

During periodate oxidation, the functional groups, in the form of aldehydes at C2 and C3 positions,^252^ are in equilibrium with hemiacetals and -aldals, which are formed with water and neighboring cellulose hydroxyl groups, respectively.^254,319^ Postmodification of the aldehyde groups can be performed chemo-selectively and gives access to CNF with diverse functionalities (Figure 8d3). Chemical post-treatment results in the introduction of sulfonate,^320^ quaternary ammonium,^321^ or carboxylate^322,323^ groups, as a function of the specific reaction mixture. Some of these modified fibers are known to have better fibrillation tendency than TEMPO-oxidized cellulose.^323^

Both TEMPO-mediated and periodate oxidation are attractive avenues for surface modification of colloids. Their regioselective chemistry will ultimately be important to reliably predict the supraparticle interactions between colloids in the assembled materials, explaining the interest in improving the efficiency and sustainability of these oxidations. TEMPO-mediated oxidation is usually conducted in the presence of chlorine oxidizing agents, but such hazardous chemicals can be avoided by employing electrochemical or enzymatic approaches.^239^ Periodate oxidation has been recently optimized at high consistency to increase greatly the overall resource efficiency and sustainability of the process.^254^ Following this line, different approaches have been developed to recycle reacted sodium periodate.^324,325^ All of these efforts are important key steps to reduce the risk, the cost and the efficiency of nanocellulose production by oxidative means in an industrial scale.

Apart from the above-mentioned modifications, most chemical treatments are conducted in water-free conditions or water-poor reaction media, as water is often regarded as a limiting factor due to its competing nucleophilicity with the cellulose hydroxyl groups and unwanted side reactions.^174,205^ In this context, it is important to take into account the hygroscopicity of cellulose,^326^ that makes it challenging to completely remove water, since in ambient conditions the nanofibrils are intrinsically covered with a hydration layer.^327^ Reactions, which can be conducted in aqueous conditions include etherification, with the most frequent chemical reactions being conducted with (a) chloroacetic acid or its sodium salt,^328^ that is, carboxymethylation, and (b) glycidyl trimethylammonium chloride to introduce cationic quaternary ammonium groups.^329^ However, treatments with nonaqueous solvents can be also conducted in a sustainable manner, if the solvent and the used chemicals can be efficiently recycled and reused.^330,331^

Recently, it was shown that deep-eutectic solvents based on sulfamic acid and urea can be used as reaction medium and reactant at the same time. Treatment of dry cellulose fibers with this solvent modified the accessible hydroxyl groups with sulfates and gave access to highly charged sulfated CNF, with a uniform width of approximately 4 nm.^332^ Esterification of cellulose with ammonium phosphate in the presence of urea phosphorylated yielded cellulose fibers with high charge. It has been shown that the crystallinity was not influenced by this treatment up to a phosphorus content of approximately 1.2 mmol/g, which is in the range of TO–CNFs. These phosphorylated fibers could readily be disintegrated into individual CNF with an uniform width of 3–4 nm.^333^ Both phosphorylated and sulfonated CNF have shown high potential in removing heavy metals for water purification.^334,335^ In contrast to the introduced wet esterification approach, conventional esterification is conducted either under exclusion of water starting from predried cellulose^246^ or solvent-exchanged cellulose.^336^ Alternatively, water can be removed by addition of high-boiling point solvents, such as ILs, and subsequent water removal under reduced pressure.^331^ It is important to consider that traditional protocols, for example, using toluene, acetic anhydride, and acetic acid, are not selective to the chemically accessible surface of cellulose and will proceed also into crystalline regions.^337^ But this can be as well used as an advantage: partial dissolution of cellulose through excessive acetylation has been reported to improve tremendously the fibrillation tendency and the energy required to obtain well-individualized acetylated CNF.^336,338^ Reactive ball milling in aprotic polar organic solvents, such as dimethyl sulfoxide (DMSO) and *N*,*N*-dimethylformamide (DMF), in the presence of anhydrides or acid chlorides enables energy-efficient production of hydrophobic CNF esters with variable ester length.^246,247,339^ These reactions are more selective to the accessible surface hydroxyls than acid-catalyzed esterification protocols based on acetic anhydride. Wood-based cellulose was modified by reactive ball milling up to a degree of substitution (DS) of ∼0.5; which represents full surface modification of C6- and C2-OH.

Recent advances in cellulose chemistry have focused on the utilization of acyl imidazole enabling esterification of cellulose in wet conditions while preserving the native MW and crystallinity of cellulose.^174,205^ It was shown that the regioselectivity of this esterification can be ultimately dictated by the amount of water in the reaction. Thereby, it became possible to conduct esterification in a highly regioselective manner providing access for to C6-succinate CNF (C6SA-CNF)^340^ and C6-acetate CNF (C6AA-CNF).^256^ This can be regarded as new avenue to CNF bearing a defined and regioselective surface modification without reducing their MW nor crystallinity.

As illustrated in Figure 8d2 and d3, functional CNF with ionic (carboxylate, sulfonate, phosphate, trimethylammonium) or hydrophobic functionalities (ester groups with various alkyl chain length) can be directly produced from cellulose fibers, which gives a broad range of surface properties for material application. Further functionalization avenues to tailor more specifically the surface chemistry of nanocellulose will be introduced in Section 3.2.4.

#### 3.2.3 Deconstruction into Colloids of Low Aspect Ratio

**Figure 9:**
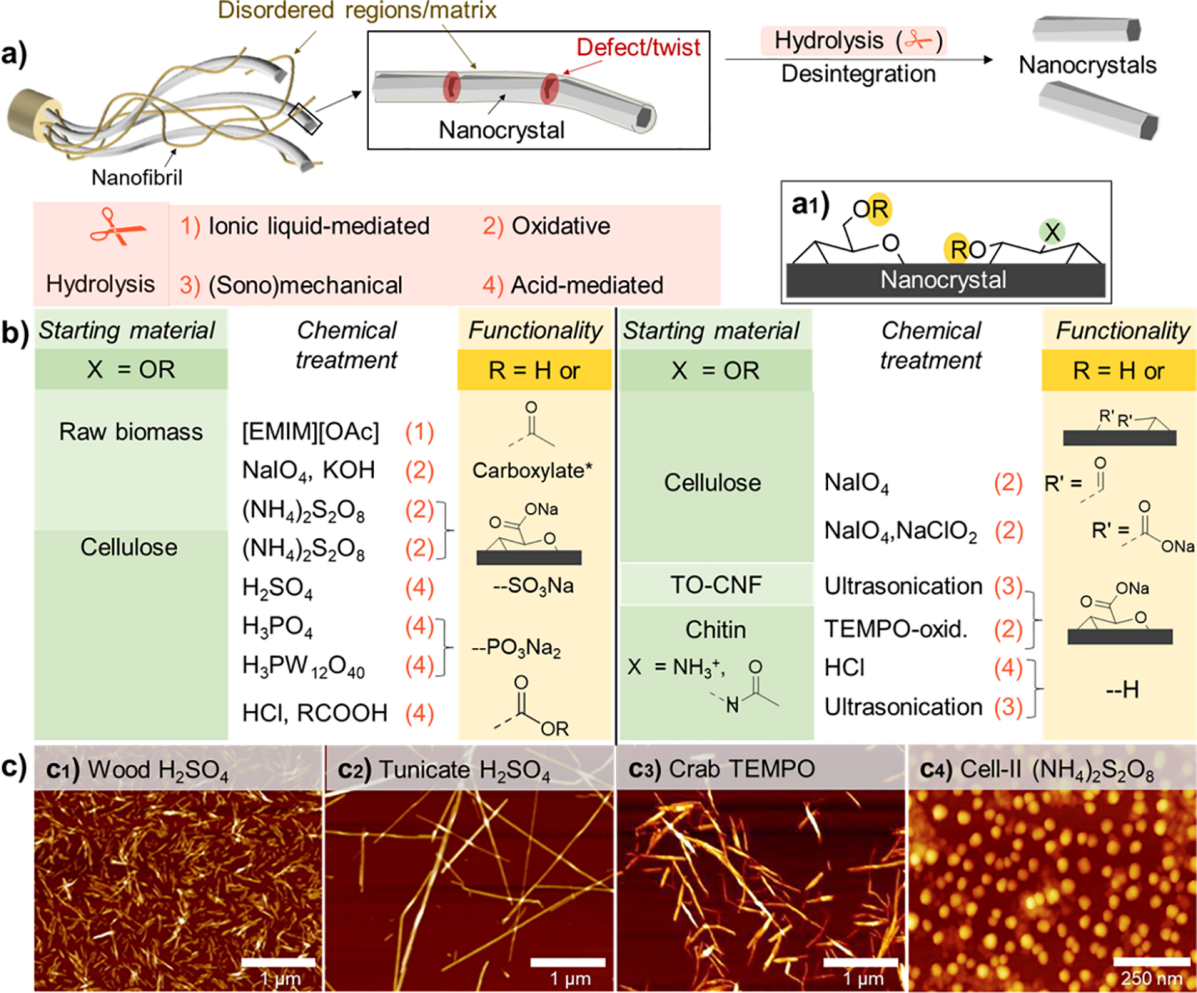
(a) Schematic extraction of colloids from biomass through hydrolysis and disintegration into individual nanocrystals. The disintegration can be mediated by hydrolytic treatment with ionic liquids, oxidizing agents, acids, or high intensity ultrasonication of charged nanofibers. (b) Conventional one-step procedures to individualize nanocrystals are listed for cellulose and chitin, including their respective surface chemistry. (c) The morphology and aspect ratio of the CNC are mainly dependent on the starting material: The shape can be tuned from spherical CNC (c4) to needle-like, high-aspect ratio CNC (b), starting from regenerated fibers and tunicate cellulose, respectively. *The chemical structure of carboxylated CNC from alkaline periodate treatment is not shown, as they originate complex aldehyde degradation reactions. (c1, c2) Adapted with permission from ref (341). Copyright 2014 American Chemical Society; (c3) Adapted with permission from ref (346). Copyright 2018 Elsevier B.V.; (c4) Adapted with permission from ref (261). Copyright 2014 The Royal Society of Chemistry.

The preparation of low aspect ratio colloids, such as nanocrystals, is based on breaking glycosidic bonds in the less ordered and defect regions of cellulose (Figure 9a). As these bonds are broken, the average degree of polymerization decreases until these regions have been fully hydrolyzed and the leveling off degree of polymerization is reached.^342,343^ This is achieved commonly by acid-mediated hydrolytic treatment with H_2_SO_4_ for cellulose^344^ and HCl for chitin,^266^ yielding in most cases liquid crystalline colloidal dispersions. The colloidal stability of these nanocrystals is based on repulsion forces of induced electrical double layers,^266,345^ due to the presence of sulfate half-esters in case of cellulose, introduced during the hydrolysis with H_2_SO_4_, or deacetylated and protonated amine groups in the case of chitin.

In general, the production of nanocrystals from biopolymers can be divided into acid-free systems such as those involving (1) IL, (2) oxidation, (3) (sono)mechanical treatment, and (4) acid-mediated disintegration with inorganic or organic acids (see Figure 9). CNCs are mostly prepared from cotton or purified cellulose from other sources, but can be also directly obtained from biomass, such as wood by IL-based or oxidative means. The ionic liquid, 1-ethyl-3-methylimidazolium acetate [EMIM][OAc], was used to treat extractive-free milled wood and enabled the direct extraction of partially acetylated CNCs, due to the capability of [EMIM][OAc] to swell the fibers and to remove most of the lignin fraction by dissolution.^219^ Moreover, the cohesive interactions of cellulose are weakened due to partial acetylation based on *in situ* formed *N*-acetylimidazole^347^ while the IL catalyzes hydrolytic processes to obtain individual CNC with a surface DS of 0.28 and crystallinity of 75% at 44% yield directly from wood. Similarly, ammonium persulfate, (NH_4_)_2_S_2_O_8_, has been utilized to directly individualize CNC bearing carboxyl groups at C6 positions from a variety biomass precursors. (NH_4_)_2_S_2_O_8_ directly removes the disordered noncellulosic plant components through chemical reactions, mainly involving sulfate radicals and H_2_O_2_, which are generated from the persulfate in acidic conditions. Depending on the source material, CNCs are extracted in up to 80% yield.^241^ Recently, it has been shown that CNCs with carboxylate functionality can be obtained from raw biomass streams using a different oxidative treatment based on periodate in the presence of KOH. This alkaline periodate oxidation fragments noncellulosic biopolymers and can be utilized to isolate CNCs directly from raw biomass, without requiring time-consuming pretreatments. The sustainability and efficiency of this method was elevated by implementing the regeneration of periodate by ozone into the process. The carboxylate groups of the final CNCs (0.4–0.6 mmol/g) originate from β-alkoxy fragmentation and further oxidation in alkaline media of the formed aldehyde groups.^255^ Surface-modified, individual CNCs with charged groups are stable in water due to their double-layer electrostatic repulsion, whereas CNC modified with noncharged acetyls are stabilized by steric means. In case of IL treatments, it is important to ensure that the utilized IL does not dissolve cellulose, otherwise its crystal structure will be affected. This risk can be avoided by using IL/solvent mixtures with a suitable net basicity ensuring only swelling treatment without regenerating the cellulose structure into cellulose II, that is, regenerated cellulose.^348^ IL-derived crystals were prepared also with [BMIM][HSO_4_], this production is based on acidic hydrolysis similar to conventional sulfuric acid treatment.^329^ Periodate oxidation of cellulose with^349,350^ and without^351^ subsequent postoxidation using sodium chlorite gives access to sterically stabilized/hairy CNC with either carboxylate or periodate groups. In these processes, oxidation of the fiber permits partial peeling of cellulose chains at the surface while maintaining contact with the crystallites, yielding nanoparticles with a needle-shaped crystalline body grafted with dangling disordered cellulose chains (hairs) at the respective end-groups.^352^ Apart from oxidative treatment, CNC and ChNC can be also obtained by (sono)mechanical treatments starting from either highly charged TO–CNFs or surface-deacetylated ChNF, respectively, through high-intensity ultrasonication.^305,306^ This is based on further repulsive destabilization at the crystalline defects due to the introduction of high amount of charges in their preparation process. In addition, modification of cellulose with maleic anhydride in an organic solvent-free process coupled with subsequent ultrasonication yields CNC with anionic maleate groups.^353^

The most common approach for CNC extraction is by acidic hydrolysis with inorganic acids. Dependent on the type of acid and conditions, the hydrolysis is accompanied by an esterification reaction for introduction of repulsive charges or hydrophobic ester groups. Apart from the sulfate half-ester groups introduced by H_2_SO_4,_^354^ H_3_PO_4_ is also widely use, introducing phosphate groups on the CNC surface. These phosphorylated CNCs are colloidally stable in polar solvents and show enhanced thermal stability due to the phosphate groups.^355^

CNC can be also prepared by a combination of treatments, using two types of acids, usually HCl and an organic acid, thereby various organic ester moieties can be introduced on the CNC surface during the hydrolysis.^356−359^ Addition of formic, acetic, citric, or lactic acids, into the hydrolysis step in aqueous HCl, yields CNCs decorated with ester groups of the respective acids. In case of formic acid and acetic acid, the resulting CNC is more hydrophobic.^357,358^ Esterification with lactic acid has been shown to be very useful in adjusting the surface energy of CNC to achieve higher compatibility with poly(lactic acid).^358^ Also, negative carboxylate groups can be introduced on the crystal surface by this approach through a combinatory treatment of citric acid and HCl.^359^ The crystallite size of cellulose varies by species, as shown in Figure 7a3 and determines also the CNC dimensions. Therefore, the shape and aspect ratio of the final CNC can be controlled by choosing an appropriate cellulose precursor type. This is clearly shown in the comparison of CNCs obtained from wood and tunicate, Figure 9c1 and c2.

ChNCs are conventionally produced by using HCl; due to the presence of deacetylated amino groups, no additional charge needs to be introduced to achieve colloidal stability. Depending on the duration of the hydrolysis, the length, the width and the degree of acetylation of the obtained ChNC can be adjusted.^360^ ChNC has been prepared from chitin by TEMPO-mediated oxidation, with adjustable carboxylate content, up to 0.8 mmol/g, yielding negatively charged ChNC.^240,346^ Deacetylation of TEMPO-oxidized chitin or TEMPO-mediated oxidation of partially deacetylated chitin yields ChNC with both amine and carboxyl groups, which are amphoteric and zwitterionic.^361,362^ The obtained ChNC is stable in alkaline and acidic conditions and can interact due to their amphoteric character with both cationic and anionic dyes or other species.

Cryo-electron tomography shows that isolated CNCs feature twisted (right-handed) morphology,^363^ which is in contrast to ChNC, produced from never-dried chitin, which seem not to have a favored twisting direction.^360^

In contrast to chitin, HCl treatment of cellulose only hydrolyzes the less ordered regions, but does not yield colloidally stable CNC in water, since no stabilizing surface groups are present nor introduced.^364^ Recently, it was shown that also vapor-based and solid-state hydrolysis can be used to produce CNC. During treatment of cellulose fibers with HCl vapor, HCl is adsorbed on the fiber and dissociation of HCl (facilitated by the intrinsically present hydration layer on cellulose nanofibrils) leads to the hydrolysis of the cellulose.^327^ Simultaneously, crystallization of less ordered cellulose chains occurs, which explains the almost quantitative yield measured for this method. Since these hydrolyzed fibers are not charged, individualization into CNC is most conveniently achieved by a subsequent TEMPO-mediated oxidation,^365^ alternatively other chemical methods can be used to introduce charged surface groups.

In addition, solid-state treatment by ball milling with phosphotungstic acid and subsequent sonication yields phosphorylated CNC with increased thermal stability.^366^ As was the case of solid-state hydrolysis, the presence of the hydration layer at the cellulose surface plays an important role enabling the dissociation of the respective acids at the fiber surface. Organic acid treatments with solid oxalic acid in a ball mill process enabled extraction of CNC in a sustainable manner, since the solid acid could be efficiently recycled and reused.^367^ In the typical cases, the yield of CNC production is moderate, up to ∼50%, when starting with cellulose-rich materials, such as cotton.^368^ The exceptions, as discussed before, include extraction with HCl^327,364^ (mass yields of >93%) or the solid-state extraction with phosphotungstic acid (88% yield).^366^ A factor that reduces the overall yield of CNC production is the remaining nonhydrolyzed, micron-sized fiber fraction. This fraction can be also valorized, for example, for the production of CNF, increasing the overall nanocellulose yield.^369,370^

Biorefinery approaches in current development integrate the production of nanocrystals.^222^ Alternatively, CNC production can be integrated in IL-based biorefinery concept^216,219^ or following hydrothermal treatment to increase significantly the overall CNC yield together with other streams such as furfural.^224^

As shown in Figure 9c4, CNC can be isolated in spherical shapes by following oxidative treatment with ammonium persulfate or acidic hydrolysis of given cellulose precursors.^261,371^ Specifically, regenerated fibers of cellulose II crystal structure are used, which feature crystallites of lower aspect ratio.^264,372^ Recently, it was shown that very similar nanoparticles can be obtained from a semicrystalline cellulose II precursors; this type of spherical nanoparticles are clearly different compared to CNC, given the soft and partly amorphous shell structure.^263−265^ The spherical cellulose II nanoparticles feature a swelling behavior and a distinctive rheology, typical of soft colloids,^263^ in contrast with the network swelling of anisotropic nanocellulose assemblies.^10^ These soft biocolloids self-assemble into nanogels with high accessible surface and are attractive for their adsorption capacity as demonstrated by their application in high sensitivity immunoassays.^265^

#### 3.2.4 Chemical and Structural Toolbox for Post Modification

**Figure 10:**
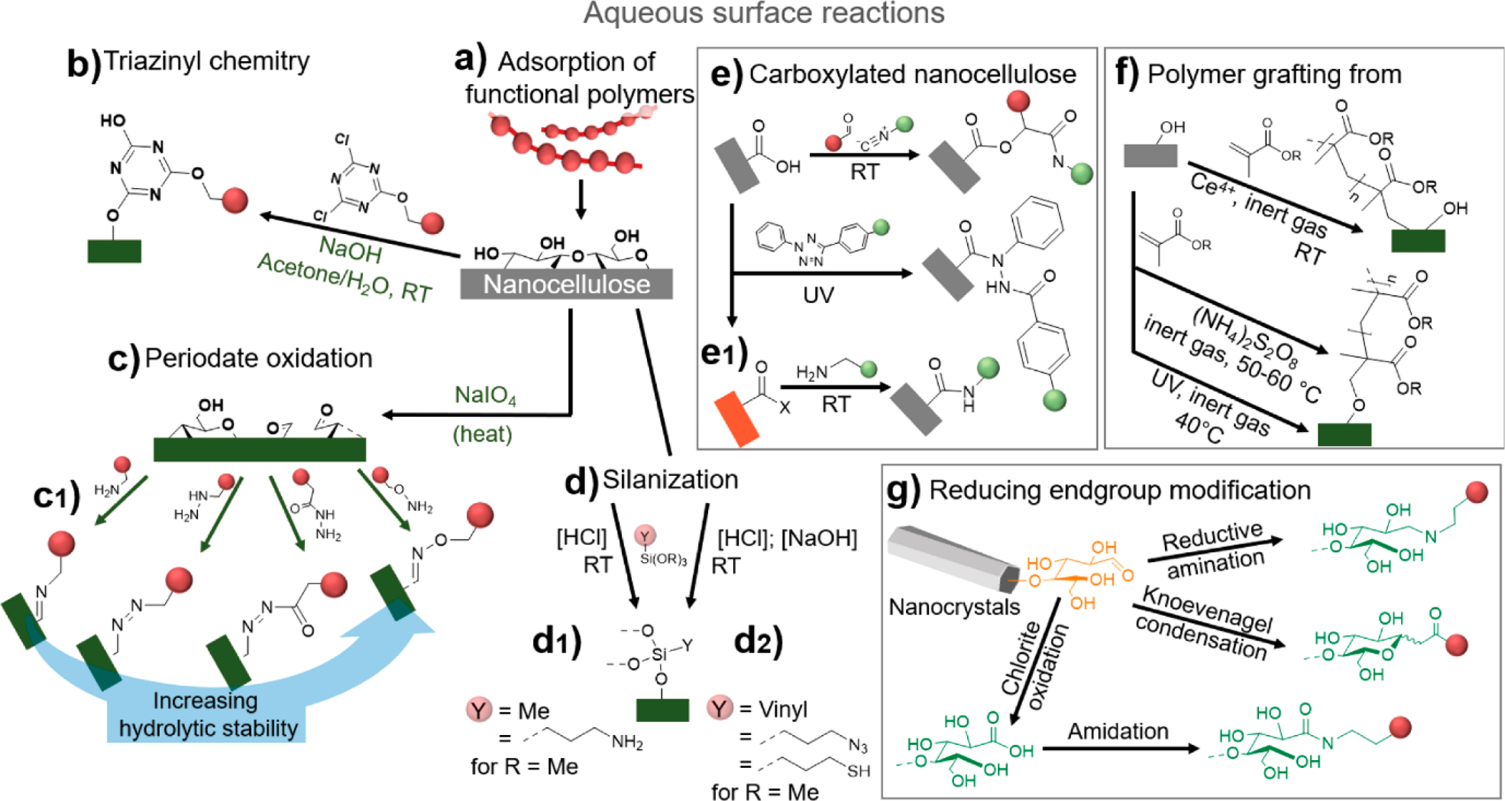
Overview of chemical reactions enabling surface functionalization of nanocelluloses in aqueous media and (a) surface modification via physical adsorption of functional polymers. The presented chemical strategies are versatile approaches to introduce functionalities (shown as red or green circles), encompassing an anchor group for postmodification, a covalently attached functional polymer chain or a functional group to tune the colloidal properties. (b) In this regard, the triazinyl reactants can be used to link functional polymers.^383^ (c) Aldehyde groups formed by periodate oxidation can be conveniently modified with (c1) amino nucleophiles in water and the kinetic stability of the final product can be tuned and increases from hydrolytically labile amines to hydrazines, hydrazones and finally oximes with moderate hydrolytic stability. (d) Silanization in water has been recently reported as a facile approach to functionalize the surface of nanocelluloses.^204,384−386^ (e) Carboxylated nanocellulose, such as TO–CNF, is mostly modified by amidation,^395^ (e1) which requires prior activation of the carboxyl group. Carboxyl moieties can be conveniently functionalized in one-step with stimuli-responsive polymers using the Passerini reaction^397^ or the photoinduced carboxyl-tetrazole ligation.^396^ (f) In addition, polymer chains can be introduced by grafting-from strategies; the polymerization of different methacrylates starts with the formation of a radical at the surface of cellulose by cerium-,^400^ persulfate-,^401^ or UV-initiation,^403^ yielding nanocelluloses functionalized with polymer brushes. (g) In the case of CNC, the reducing end-group plays an important role in the functionalization and can be used to introduce chemical anisotropy. End-group functionalization is based on aldehyde chemistry and reducing end groups can be modified, among others, by amidation coupled with prior chlorite oxidation,^417^ Knoevenagel condensation,^406^ or imine formation with amines and subsequent reduction (reductive amination).^407^

The moderate reactivity of hydroxyl groups of nanocelluloses and other related colloids hardly allows a selective and efficient modification, especially if reactions are conducted in water or aqueous media. Therefore, it is of major importance to address the issue of finding facile methods that incorporate anchoring groups for postmodification or to directly attach functional motifs or polymers. Aqueous modifications avoid hazardous and flammable organic solvents, increasing the safety of the reaction and the overall sustainability, which especially matters in the chemistry of renewables. Carrying out chemistry in aqueous conditions is even more important in case of CNF modification.^205^ CNFs are commonly handled as dilute aqueous dispersion and hence a considerable amount of energy and solvent are necessary to transfer/exchange CNFs from water to organic solvents, for example. Conventional drying of CNF and subsequent dispersion in organic solvents is not recommended due to strong cohesive interactions that occur upon drying, which eventually decreases the aspect ratio and dispersibility of CNF.^289^ This section summarizes a number of versatile chemical modifications possible in aqueous conditions that are selective to the surface of nanocelluloses. Apart from covalent modification, the surface functionalization via physical adsorption of functional polymers is straightforward possibility (Figure 10a). Functional carboxymethyl cellulose (CMC) can be successfully adsorbed onto CNF; this surface modification takes place even though both CMC and CNF are negatively charged. Indeed, the interaction are possible by the addition of small amounts of CaCl_2_ that shields repulsive double layer repulsion. Thereby, azido- or propargyl-functionalized CMC have been adsorbed onto CNF, to make them ready for click chemistry ligation.^373,374^ Click chemistry is a generic term for highly efficient and selective reactions that are generally orthogonal to most biopolymeric chemistries and can be utilized to modularly introduce a broad range of functionalities.^375^

Apart from CMC, also functional xyloglucans easily adsorb on the surface of nanocellulose, due to the native interactions of cellulose with hemicelluloses, and can be used to introduce short peptide motifs that improve the adhesion of cells.^376^ Finally, inspired by enzymes in nature, the strong affinity of cellulose-binding domains has been utilized for subsequent nanocellulose modification.^377,378^

Polymer adsorption can be driven by electrostatic interactions between TO–CNC and cationic block copolymers, to introduce stimuli-responsive gelation at a given temperature.^379^ Similarly, block polymers with bromine groups adsorb onto TO–CNF to enable polymer grafting starting from these groups, for example, via controlled surface-initiated polymerization in an aqueous system and to tailor the morphological properties of colloidal and aerogel systems.^380^ Specific interactions between amine and the CNF hydroxyl groups can be further utilized to *in situ* polymerize conductive polymers, such as aniline or pyrrole, via polymerization. Thereby multifunctional electroconductive hydrogels and X-band microwave absorbers based on CNF have been obtained.^381,382^ Overall, the adsorption of polymers on cellulose represent a simple opportunity to produce functional colloids.

Click chemistry enables the straightforward introduction of functional polymer chains or functional groups in a covalent manner but requires the prior introduction of an anchor group, this can be achieved by reaction with trazinyl derivatives (Figure 10b).^383^ This modification was conducted in acetone/water to graft onto the hydrophilic polymer chain. To introduce more hydrophobic groups, such as a propargyl moieties for click chemistry, the use of organic solvents is necessary due to solubility issues in water. Nevertheless, by choosing appropriate functional groups triazinyl chemistry can be a very powerful method to tune the interfacial properties of CNC, for example, to produce amphiphilic CNC, which are colloidally stable in both polar and nonpolar organic solvents.

Another efficient and simple route for surface functionalizing nanocelluloses is based on the principles of the aqueous silanization (Figure 10d). Conventional silanization is conducted in the absence of water, in organic solvents, but recently silanization via functional trimethoxy- or triethoxysilane was conducted in aqueous media either in a one-step approach in alkaline condition or using a two-step process following acidic hydrolysis and subsequent alkaline condensation. This versatile method enables introduction of azido, thiol, or vinyl groups in a very simple process.^204,384^ These functional groups could be covalently bound to the surface of cellulose in aqueous conditions,^384^ and the introduced groups can be postfunctionalized, for instance, via click chemistry.^204,384^ On the basis of this principle, the portfolio of silanes available has been extended by successful functionalization of CNF with trimethoxysilane and 3-aminopropyl trimethoxysilane. The former silane increased the hydrophobicity of CNF to increase, for example, the colloidal stability in organic solvents; the latter introduces reactive amino groups for postmodification.^385,386^

Periodate oxidation (Figure 10c), which was discussed previously, is highly attractive as a method for the surface modification nanocelluloses. It can be conducted in dilute and aqueous conditions,^387^ and the aldehyde groups can be further functionalized with different amino reactants. Imines formed by reaction with amino groups are rather labile in aqueous conditions and require post-treatment with reducing agents to form more stable amine linkages (Figure 10c1). Hydrazones formed by reaction of aldehydes with hydrazines or hydrazides are more stable in water and can be used for direct and efficient aldehyde functionalization, with no need for reductive treatments.^388,389^ Reactions of aldehydes with aminooxy reactants are very promising, due to the pronounced hydrolytic stability of the resulting oxime, which is higher than for the other groups.^390,391^ Aldehyde-based chemistry starting from periodate-oxidized nanocellulose are highly promising chemical reactions and are already used for different purposes, among others, to design self-healing hydrogels based on periodate-oxidized CNC and hydrazide-functionalized polymers.^392^ The principles of aldehyde reactivity can be also transferred to other biopolymers, such as nanochitin, offering a straightforward avenue to colloids with various functionalities.

Carboxylated colloids, such as those obtained via TEMPO-oxidation, are mainly modified by amidation (Figure 10e). However, amidation in aqueous medium requires the use of reagents to activate the carboxyl groups, such as carbodiimides, and has generally a poor atom economy (Figure 10e1).^393^ Still, it is a widely used method to produce multifunctional materials.^394,395^ Recently, new approaches have been introduced to enable direct functionalization of the carboxyl group in aqueous media, such as UV-induced tetrazole ligation to graft polymer chains onto TO–CNFs.^396^ A three-component Passerini reaction was established enabling the introduction of temperature-responsive polymer chains in water.^397^ These new methods further expand the repertoire of nanocellulose modifications to obtain functional colloids.

Chemical modification of the surface hydroxyl groups via cerium-induced radical grafting is one of the various methods to tune the wettability of nanocellulose and can be used to functionalize both CNF and CNC in water (Figure 10f). Cerium(IV) opens the pyranose ring of the cellulose monomer unit by oxidation of two adjacent hydroxyl groups (C2-OH and C3-OH). Thereby, an open-ring structure is formed featuring one aldehyde group at C3 and a radical at C2, which initiate polymerization with acrylates from the surface of nanocellulose.^398−400^ Alternatively, polymer grafting has been developed in aqueous media based on initiation with ammonium persulfate^401,402^ or direct UV-initiation.^403^ In the case of radical polymerization, the methods are generally conducted in oxygen-free, inert atmosphere to reduce the quantity of undesired quenching and scavenging reactions. Conducting chemical modification of CNF in organic solvent is a rather time-consuming approach. This is different for low aspect ratio biocolloids, such as CNC, which are commercially available in freeze-dried or spray-dried forms^162^ and can be redispersed efficiently. Hence, CNC surfaces can be tailored more efficiently in organic media than CNFs, for example, by introducing polymer brush architectures.^404,405^ The brush-modified CNC can be redispersed in given solvents and utilized in applications involving liquid crystals and to reinforce composites.

The reducing end-group of polysaccharides (Figure 10g) is in equilibrium between the hemiacetal ring and the open-chain aldehyde form.^406^ These end-groups can be functionalized by conventional aldehyde chemistry and are especially important for anisotropic CNC modification. A two-step process to introduce functionalities onto the CNC’s end group involves the generation of an imine bond through reaction with an amine and a subsequent reduction to obtain a hydrolytically stable product.^407^ An interesting alternative, is the direct modification with other amino reactants, which form hydrolytically more stable conjugates (Figure 10c1). The aldehyde group can be also converted with NaClO_2_ into a carboxyl acid and subsequently modified via amidation to introduce, among others, stimuli-responsive polymers.^408^ In a similar approach, an alkyl bromide initiator can be introduced to enable grafting of thermoresponsive polymers, yielding anisotropic CNC with thermally controllable liquid crystalline properties.^409^ In general, the spatio-selective end-group functionalization of CNC is of major interest to obtain colloids with anisotropic chemical structure that can be used to fine-tune its properties.^410,411^

In comparison to nanocellulose-based chemistry, the chemical modification of chitin is mostly based on the reaction of the chitin hydroxyl groups, for example, through esterification^412−414^ or etherification^415^ in nonaqueous media, under rather harsh conditions. In addition, the carboxyl groups of TEMPO-oxidized ChNC has been used to introduce functionalities by amidation.^394^ The natively present amino groups of nanochitin are suitable for postfunctionalization due to their nucleophilicity and reactivity. Likewise, amino side chains in proteins are one of the most important reactive groups used for functionalization.^416^ However, the risk of using amino groups in nanochitin for modification is the reduction of the colloidal stability, due to the use of the protonated amino groups. This could be overcome by adapting state-of-the-art protein chemistries, which enable a highly controlled and partial modification of the accessible amino groups, and are expected to further advance in future chitin chemistry.

### 3.3 Soluble Biopolymers

Most biopolymers are extracted through solubilization following a precipitation/regeneration step. This process alters the biopolymer structure due to chain rearrangement, but depending on the severity of the extraction process might also change the chemical structure (Section 3.1). Apart from this, solubilization is important in polymer chemistry of cellulose and chitin, as it enables homogeneous modification of the biopolymer, that is, bulk modification and hence the properties of these biopolymers can be more drastically altered and a higher number of functional groups can be introduced (Figure 7a3). Bulk modification can occur in reactions under heterogeneous conditions but proceeds to the ordered, crystalline regions, surpassing the limit of surface reactions. Typical examples are the synthesis of CMC in 2-propanol/water,^418^ the production of cellulose acetate,^419^ and periodate oxidation if used in excess.^252,254^

In this section, biopolymer dissolution and homogeneous chemical modification are discussed. Homogeneous modification contrasts with heterogeneous routes; in the latter, solvent media facilitates diffusion of reaction components, but the polymer remains suspended or dispersed and chemical modification is confined to its chemically accessible and less-ordered regions. Therein, it is possible to percolate the fiber network, as in CNF, while the structural properties (crystalline and less ordered regions) are preserved. Thus, the surface of CNF can be chemically altered, for example, to decrease their hydrophilicity with no major effect on the mechanical performance. Homogeneous modification is not limited to the surface, but reactants and the respective polymer are dissolved, enabling complete chemical accessibility. It is important to distinguish here between intrinsically water-soluble biopolymers and those that are insoluble in water, which require special solvent systems to achieve solubilization.

In general, the MW of the polymer and its chemical and chain architecture affect the solubility and therefore different dissolution procedures have emerged over the years. Depending on the initial chemical functionalities present in the biopolymer, solubilization followed by chemical modification completely alters the properties but can be done in a controlled manner. Also, as the degree of reactivity can vary for the different functional groups, depending on their position. Homogeneous solubilization gives access to a controllable total DS and the substitution pattern along the biopolymer chain; potentially even the substitution pattern of a single monomer in the chain, for example, in the case of proteins.^420^

**Figure 11:**
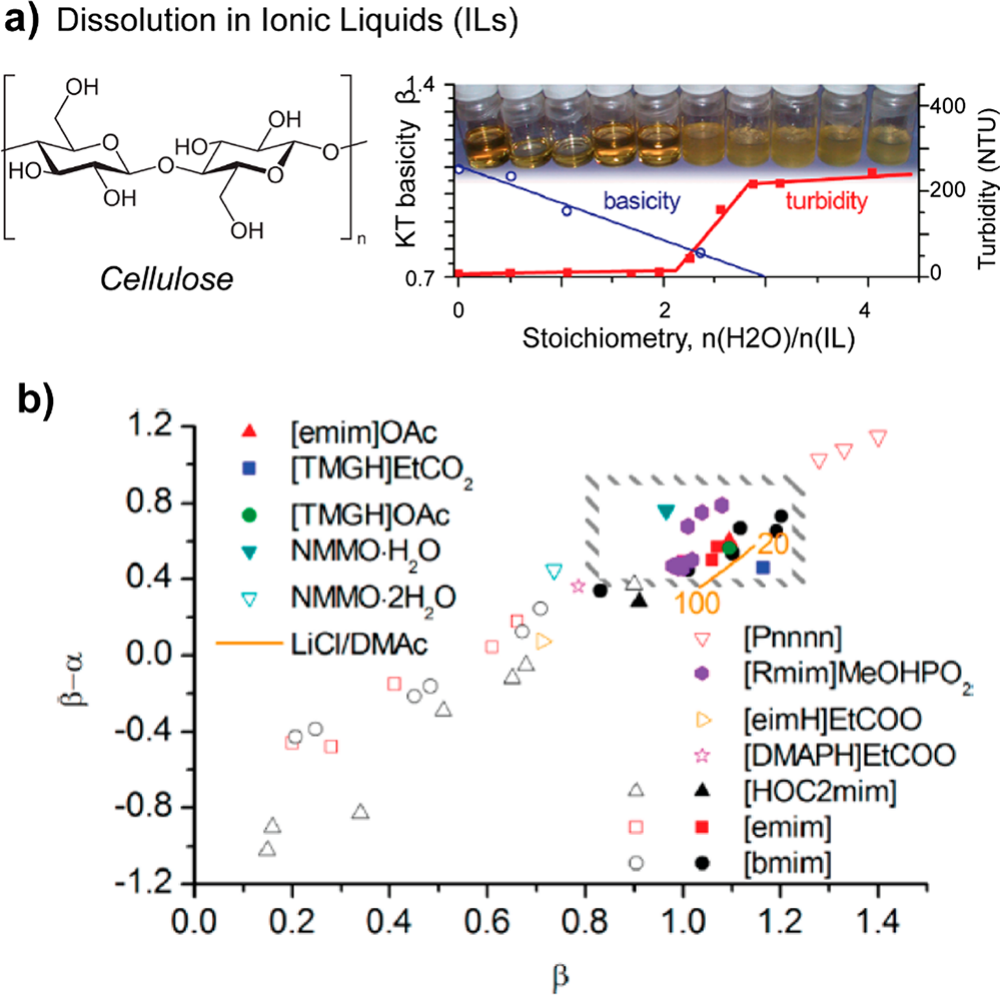
Cellulose dissolution in basic ionic liquids (ILs). (a) Solubility of [EMIM][OAc] can be tailored by the molar ratio of water and IL (n(H_2_O)/n(IL)) and monitored by the Kamlet–Taft parameter β, that is, hydrogen bond basicity. Regeneration occurs at a given threshold condition, as indicated by the increased turbidity (X = OH). (b) Cellulose is soluble in a dissolution window (gray box), as shown in the β–α (net basicity) versus β plot (full symbols are cellulose solvents, empty symbols nonsolvents), comparing ionic liquids with standard cellulose solvents based on NMMO/water and DMAc/LiCl. The solvency parameters in ionic liquids are varied by the amount of water and temperature, the line for DMAc/LiCl, represents its parameter in a temperature windows between 20 to 100 °C. Reprinted with permissions from ref (348). Copyright 2012 American Chemical Society.

Biopolymer homogeneous interactions, in biomass-derived polymeric materials, are key in hierarchical structures, and can be used to manipulate biopolymer solubilization for materials engineering. Cellulose, unlike most petroleum-based polymers, does not melt but decompose at elevated temperatures (>200 °C). To solubilize cellulose, it must be dissolved by special solvents or chemically modified. Cellulose dissolution happens through swelling, where extensive hydrogen bonded network forms between the three hydroxyl groups in the repeating glucose units and interact with the solvent molecules, disrupting the inter- and intrachain hydrogen bonding.^33^ Yet, the glucose rings of cellulose possess also hydrophobic sites for interactions (axial H atoms pointing perpendicular to the ring), which are also important to consider.^421^ Efficient cellulose dissolution has been facilitated by a range of ionic liquids (ILs) (Figure 11), including aqueous and nonaqueous systems and salt melts, allowing shaping of cellulose into fibers, films or 3D scaffolds.^422^ This avenue opens up as well paths toward nanostructured regenerated cellulose and tailored cellulose derivatives, which are not accessible via heterogeneous pathways.^372,423^

Diverse solvent systems are needed for chemical modification as varying reactions work under given solvents and for dissolution of derivatives, for example, for characterization. As chitin resembles cellulose’s semicrystalline structure, the same solvents that solubilize cellulose have been tested for chitin.^424^ For example, solvents/solvent systems include *N*,*N*-dimethylacetamide lithium chloride (DMAc-LiCl),^425^*N*-methylmorpholine-*N*-oxide/water (NMMO),^426^ and dimethyl sulfoxide/tetrabutylammonium fluoride (DMSO-TBAF)^427^ and are usually able to dissolve hemicelluloses as well.^428^

ILs have a low-vapor pressure and can be recycled upon dissolution of cellulose, for example, 1-butyl-3-methylimidazolium chloride [BMIM][Cl]^429^ and 1-allyl-3-methylimidazolium chloride [AMIM][Cl]),^430^ which are also able to dissolve chitin,^431^ and lignin.^432^ Cationic imidazolium-based ILs have good solubilization power, which is affected by the anionic counterpart. For example, for lignin solubilization, ILs containing large, noncoordinating anions [PF_4_]^−^ and [BF_6_]^−^ are unsuitable; yet ILs such as 1-methyl-3-methylimidazolium methylsulfate [MMIM][MeSO_4_] and [BMIM][MeSO_4_], with sulfate based anions, appear to be effective solvents for lignin.^432^ For swelling and dissolution investigations of cellulose, XRD results demonstrate how 1-ethyl-3-methylimidazolium acetate [EMIM][OAc] molecules penetrate within hydrogen-bonded sheets of cellulose I during dissolution and cause a slight expansion of the lattice, hence an intermediate structure is formed before complete dissolution.^433^ Dissolving cellulose in [EMIM][OAc] also acetylates the biopolymer as an unwanted side reaction; the presence of as little as 1 wt % lignin, based on cellulose mass, increases the initial rate of cellulose acetylation from 1.8 to 4.7%/h.^434^ The IL solvent parameters, hydrogen bond acidity (α), hydrogen bond basicity (β) and net basicity (β–α) (so-called Kamlet–Taft values) are used to explain the solubility of cellulose in ILs and similar cellulose solvents. Increasing the amount of water in [EMIM][OAc] decreases β as well as cellulose solubility, triggering cellulose regeneration (Figure 11a). A good correlation of cellulose solubility is achieved in a plot of (β–α) versus β, shown in Figure 11b, which allows the identification of a cellulose dissolution window, for a broad range of cellulose solvents, depending on temperature and water content.

Solvent reusability is highly valuable from the sustainable and upscaling point of view, for example, following distillation^330^ and phase separation.^435^ Reactive dissolution has been enabled through reaction with CO_2_ in switchable solvent systems.^436^ These have been recently demonstrated as cheap, easy to recycle ILs that act fast for cellulose solubilization under mild conditions.^437,438^

The functional groups of cellulose^439^ (OH), chitosan (OH, NH_2_), alginate (OH, COOH), dextran (OH), and hyaluronic acid (COOH, NH_2_COCH_3_) are attractive for chemical modification to tailor their structure and properties. Therein, conventional modification approaches such as esterification and etherification are used. Acylation activation reagents such as 1,1′-carbonyldiimidazole (CDI) have facilitated polysaccharide esterification with monofunctional carboxylic acids under mild conditions, based on the formation of reactive acyl imidazoles.^439^ Polysaccharide derivatives can be modified regioselectively with protection/deprotection chemistry to provide well-controlled structures and to understand the structure–property relationships of related derivatives.^440^ Common protective groups such as trityl^441−444^ and silyl^445^ have been introduced to protect regioselectively the hydroxyl group of cellulose (mostly the C6-OH group) and further modification of remaining functional groups followed by removal of the protecting group, provides a broad range of cellulose derivatives with a defined chemical structure.

Owing to its inherently high chemical reactivity, lignin chemical modification starts from its extraction. Lignin undergoes condensation reactions and repolymerization upon isolation (see Section 3.1).^446^ Lignin high reactivity originates from its subunits and the reactive aromatic and aliphatic hydroxyl groups present in its backbone. The possible chemical reactions of lignin have been recently summarized in a comprehensive review.^446^ Lignins from grasses and softwood feature a high amount of aromatic hydroxyl groups and possess hence a high potential for application in resins to replace phenols. Interestingly, it was shown that in case of softwood lignin only about 25% of the theoretically available hydroxyl groups are chemically accessible for reaction with formaldehyde, whereas hardwood lignin fractions, which are commonly less reactive, consumed formaldehyde close to the theoretical limit.^447^ The repolymerization during lignin isolation increases the number of C–C linkages; hence, the structure is further cross-linked and rendered more condensed, increasing the average MW of the extracted lignin. This causes a reduction in solubility and restricts the miscibility in a polymer matrix, which is also affected by the strong tendency of lignin to self-associate.^448^ Chemical modification has been demonstrated to enhance lignin isolation^449^ and can improve lignin compatibility in polymer matrices, increasing its potential to develop new biobased polymer blends.^450^

Dissolved lignin has an intrinsic tendency to assemble into spherical particles that minimize the surface area in contact with a nonsolvent phase. The preparation methods and applications for lignin nanoparticles have been reviewed.^451^ Lignin particle preparation can roughly be divided into dry (well-controlled drying of dilute lignin solutions)^452^ and wet (precipitation) methods (see Section 6.1.3.4).^169^ Spherical lignin nanoparticles are used as dispersants and in the formulation of coatings, adhesives, and composites due to their distinctive interfacial behavior (Section 6.1.3.4).

The chemical modification of proteins is rather particular due to their native assembly into either globular or fibrillar structures originating from their tertiary structure. As this specific assembly is based on folding patterns induced by interactions of the protein backbone and side chains, some functions are nonchemically accessible or “buried” in the native state of the proteins. These groups can be rendered accessible by denaturation with chaotropic agents such as guanidine, which can disrupt the hydrogen bonding network.^453^ Following this approach, the intrinsic functionality of the protein is lost since the structure is irreversibly altered. Other functional groups, which are not involved in cohesive interactions, might be still available and can be modified to introduce functional motifs, without affecting the intrinsic protein structure.^454^

The click chemistry concept introduced already in Section 3.2.4 is a well-established modification route, which has a high reaction efficiency and can be used to introduce diverse functionalities under mild and usually aqueous conditions.^455^ Related reactions are popular for postfunctionalization of biomass-based carbohydrates,^456^ proteins^457^ and lignin.^458^ It is important to consider that a preceding step is required to introduce a chemical anchor group onto the biopolymer structure, which can react via click chemistry. The most prominent groups are the azido, alkyne, thiol and alkene group. The azido group react with alkynes via azide–alkyne Huisgen cycloaddition,^459^ whereas the thiol groups are postmodified by thiol–ene^460^ reactions with alkenes.^461^ Other examples are selected Diels–Alder reactions, which have superior reactivity, thereby enabling bio-orthogonal reactions at very low concentration.^462^ Cu-catalyzed azide–alkyne cycloaddition is particularly interesting for the modification of the polysaccharide reducing end groups, enabling, for example, a pathway to fully biobased and biopolymer-synthetic polymer block copolymers, which can self-assemble into ordered nanostructures for application in pharmaceuticals and energy harvesting.^463−465^

## 4 Length Scales and the Physics of Biopolymer Assembly

**Figure 12:**
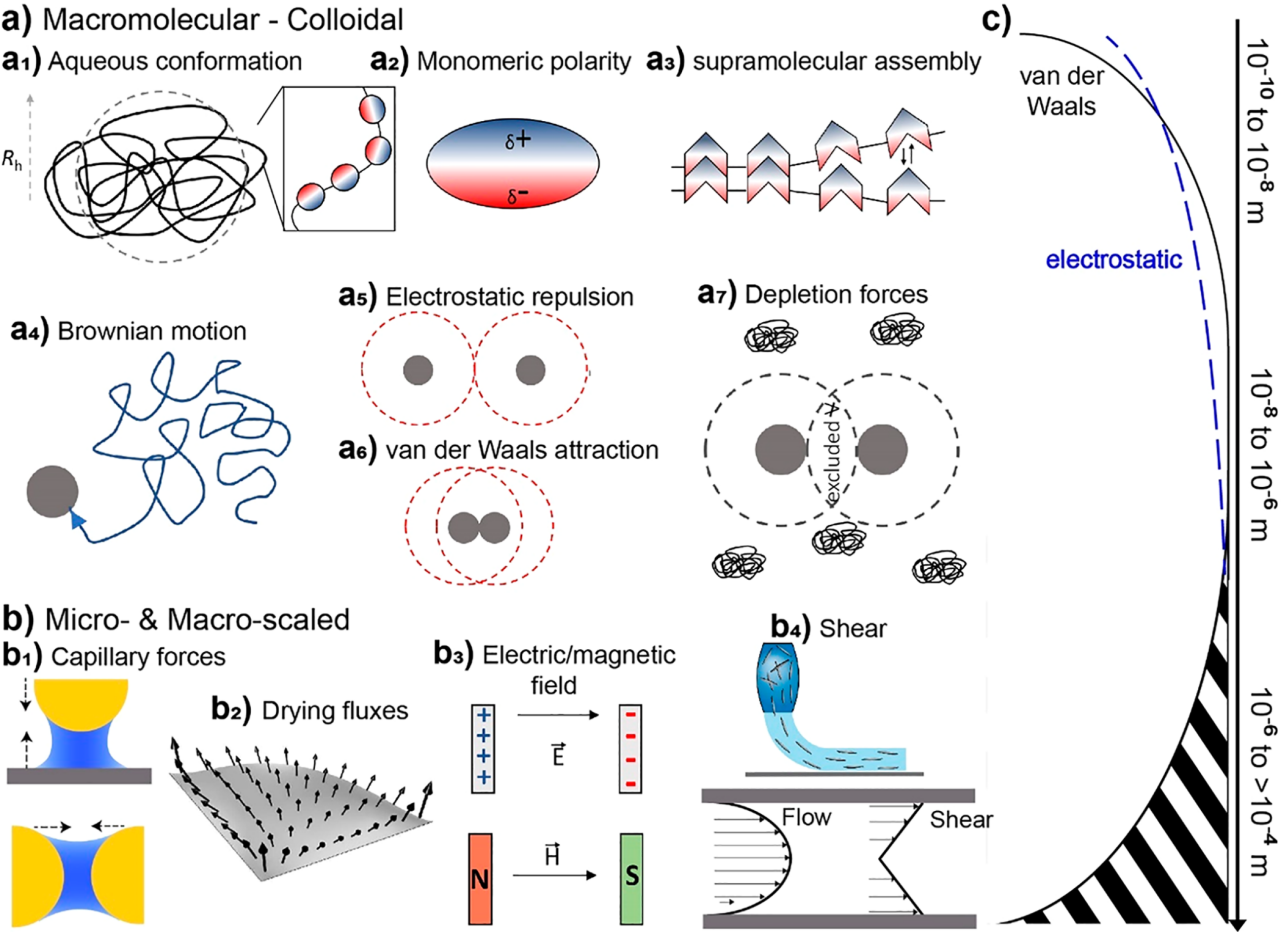
(a,b) Main forces occurring from the macromolecular- to the macroscopic scales. (a) Physical forces relevant to the macromolecular and colloidal scales, mainly (a1) solvent interactions and conformability of the polymer as a result of its rigidity, which can be visualized through the hydrated radius (*R*_h_) of the polymer. (a2) Illustration of a model monomeric building block and its dipoles, which play a crucial role in (a3) supramolecular self-assembly. (b) Physical forces relevant to the colloidal scale (herein in the presence of macromolecules) including (a4) thermal forces and associated Brownian motion, (a5,6) electrostatic repulsion, vdW attraction, and (a7) depletion forces. (b) Micro to macro-scaled forces. In (b1), capillary forces between a particle and a flat surface (top) as well as between particles (bottom). In (b2), inhomogeneity in the drying fluxes (black arrows) result in a higher evaporation toward the edge of a sessile drop; inducing convection and increasing the deposition of biopolymers and biocolloids in areas subjected to higher evaporation rates. (b3) Depiction of electric (top) and magnetic (bottom) fields. Typically, a biocolloid will have its (permanent or induced) dipole oriented along these fields. (b4) Shear forces are dominant in additives manufacturing and present complex flow fields and associated shear stresses. (c) Magnitude of electrostatic and vdW forces as a function of scale. Note (1) (macro)molecular to colloidal forces (a-to-b) are intertwined, wherein vdW, electrostatic repulsion, *etc*. are of molecular origin and are in the colloidal range, (2) the different power laws affecting electrostatic and vdW forces at short ranges and, (3) a transition toward macroscale forces to control the collective behavior of biopolymeric materials. Interfacial interactions as governed by described forces result in another important process parameter, as described in the following section.

The variety of functional groups in biopolymers, their interactions and hierarchical structures have a significant impact on their processability in solution or suspension as well as the properties of their assemblies. One of the key aspects associated with biopolymers is that they form cohesive materials upon water removal. The assembly is thereafter significantly affected by the dispersion state prior to the removal of water as well as the dewatering process itself. In this section we review the forces associated with the properties of biopolymers upon dispersion and water removal. When evaluating the behavior of biomacromolecules interacting with solvents, other biopolymers and interfaces are subject to a range of physical forces (Figure 12). We delineate four length scales relevant to biopolymers assembly (macromolecular, colloidal, micro and macroscopic) and relate them to the relevant forces to better understand relevant structure-process-property relationships.

### 4.1 Assembly at the Macromolecular Scales

The forces acting directly on dispersed oligomeric and highly polymerized biopolymers are generally associated with the relative mobility of the units, their electrostatic potential maps, and associated solvent- and self-interactions. Statistically, thermodynamic models give reasonable estimates of phase-separation and conformation as a function of solvent and secondary phase by introducing a mixing parameter (χ) that accounts for the energy of mixing.^466−468^ Some large scaled parameters can be extrapolated, for instance, the hydrodynamic radius (*R*_h_), which relates to the solvated conformation of given biopolymers (Figure 12a1).

The principal difference between synthetic polymers and renewable biopolymers is that water is almost never an ideal solvent for biopolymers. Moreover, the conformation of biopolymers in dissolved state or at interfaces cannot always be modeled as efficiently as that of a synthetic polymer in a given organic solvent. Additionally, a large subset of biopolymers is inherently highly branched with nonrepetitive motives, which prevents the prediction of their characteristics in solution.

In the case of biopolymers, individual properties of the monomer units and the topology of their branches can be used to predict the behavior of the biopolymer in solution and during assembly. For instance, this approach has resulted in fruitful simulation work to predict the properties of the crystal and amorphous domains of celluloses.^34^ As such, the polarity and pH-dependent ionizability of the functional groups as well as the building blocks forming the backbone give reasonable insights about the behavior of biopolymers in water (Figure 12a2). The *R*_h_ and radius of gyration, in the biopolymer dissolved state, reasonably describes the 3D conformation and their form factors.

Beyond structural considerations associated with backbone linkages and branching, the polarity of the accessible functional groups significantly affects vdW interactions. Combined with hydrogen bonding, vdW forces are critical in the formation of crystalline domains such as in nanocelluloses.^469^ These forces form part of the adhesion and cohesion effects observed in the assembly of such biocolloids, for instance, in engineered materials (Figure 12a3). The most relevant range of forces for interactions of biopolymers in solution is hydrogen bonding, given that most biopolymers undergo such interactions due to their nonionized functional groups involving hydrogen donors and acceptors. The presence of charged groups has a significant effect on long-range electrostatic interactions between biopolymers and biopolymers at interfaces. vdW forces and hydrogen bonding are substantially lower in magnitude and range than electrostatic forces. As such, the weaker but more dynamic nature of hydrogen bonding and vdW forces lead to more significant effects as far as the mechanical properties of biopolymeric assemblies are concerned, as they enable more interactions over the larger scale of the biopolymer assemblies, as is the case of cellulose–cellulose or cellulose–hemicellulose interactions.^470^ For biopolymers, as exemplified by cellulose-cellulose interactions, hydrophobic effects should be considered from the perspective of relative affinity with water, where a higher interaction energy may be obtained between two hydrophilic/polar biopolymers than the sum of their associated water interactions.

The thermodynamics of interaction associated with enthalpic and entropic contributions lead to the formation of high affinity bonds or conformational differences, respectively, and describe the system effectively. In the case of biomacromolecules, a combination of interactions is generally at play;^471^ therein, an exothermic process is usually associated with the release of adsorbed water into the bulk. In general, the main forces occurring at the macromolecular scale are vdW and π–π interactions inclusive of aromatic quadrupoles, hydrogen bonds, and electrostatic interactions. Additional information on interfacial interactions and adsorption of macromolecules can be found in Section 4.4.

### 4.2 Assembly at the Colloidal Scale

Colloidal-scale interactions are unified under an energy potential described, for instance, by the DLVO theory.^472^ Therein, vdW and electrostatic forces are summed and expressed as a function of the distance between colloidal surfaces (Figure 12d,a5,a6). The DLVO theory predicts the stability of biocolloids using geometrical (including roughness) and surface potential considerations.^473^ Nevertheless, in the nanometric scale, biopolymeric colloids such as CNCs or lignin nanoparticles, additional structural forces associated with colloidal self-assembly take place. These include depletion, also associated with lock and key structures, packing constraints, and convection (Figure 12a7),^474^ the latter of which scales to the macroscale (Figure 12d).

Depletion forces are associated with the presence of cosolutes, such as macromolecules or small particles, under concentration gradients leading to short-range interactions and chemical potential differences producing a net attractive force between the colloids. Depletion forces are relevant when the distance between colloids is proportional to the size of the cosolute. Also, for anisometric particles, self-depletion leads to liquid crystalline phase transition as determined by Onsager theory, where chirality, size, DLVO potential and anisotropy determine the various concentrations at which each phase occurs.^475^ Related phase transitions can be accurately predicted, for instance, for CNCs. Importantly, the range of colloidal forces described so far are impacted by the roughness scale. For instance, DLVO forces are strongly influenced by surface topographical/chemical inhomogeneities in spherical particles.^473^ Thermal motion, that is, Brownian movement (Figure 12a4), is considerably lower for colloids than for macromolecules, however it does play an important role when associated with fluxes arising from water removal (Figure 12b2). As such, drying fluxes lead to concentration gradients across the dispersion and to surface tension gradients across drying interfaces. These result in convection fluxes that significantly affect the packing and orientation of colloids in dispersions.^476,477^ Lastly, in the case of dispersions, shear-induced alignment takes place under the shear of a “doctor-blade” thin layer that forms (<100 nm) on a surface, leading to capillary flow alignment.^478^ At the macromolecular scale, the diversity in architectures and complex polarity profiles of the monomer units in biopolymers prevents accurate prediction of their dispersion behavior. This contrasts with the colloidal scale, where the current theoretical framework often allows accurate prediction of the dispersion behavior. The impact of colloidal-scale forces enables the prediction of long-range order in biocolloidal assembly. However, the association between the components and the strength of interactions still requires a better understanding, even if it has been a subject of several recent reports.^479−482^ More information on interfacial interactions and adsorption of colloids at interfaces, such as in Pickering and multiphase liquid systems is included in Section 4.4.

### 4.3 Assembly at the Micrometric and Macroscopic Scales

Forces include principally shear and capillary effects at the micrometric and macroscopic scales. With micrometric particles, drying-induced convection has a minimal effect; however, shear has a high impact on flow-induced particle packing. This is particularly the case of anisotropic particles, where shear-flow alignment is efficient in directing their assembly and reducing defects to maximize supramolecular and supraparticle interactions (Figure 12b4).^483−485^ As such, an important consideration is the elastic moduli of the particles including the transversal elastic modulus of rod-like or fiber-like particles and their response under applied forces. Furthermore, biopolymeric solution or gel under shear is in a balance between inertial and viscous effects, corresponding to the momentum and the respective counteracting forces, which play an essential role in structuring. While shear has been shown to enhance long-range order in biocolloid and biocolloids/biopolymers assemblies,^486,487^ the interplay between interfacial shear and internal flow fields is crucial in the transition from fully laminar to highly turbulent systems. The latter is defined by the Reynolds number, which relates the flow rate and the dynamic viscosity. Another important aspect related to shearing of anisometric biocolloids or large biopolymers is that most of them display shear-thinning behavior.

Electrical and magnetic fields are important “noncontact” stimuli that can affect the collective behavior of gels or biocolloids (Figure 12b3,b4).^488^ Their application, related to the electric and magnetic dipole-moment of the biopolymeric building blocks, depend on the dielectric properties of the particles. The magnetic dipole-moment determines the polarizability of a particle and shows the strongest response under permanent dipoles. Notably, a dipole can be induced as a result of anisometry in a building block.^489^ CNC presents a permanent dipole moment, while for instance, the filamentous bacteriophage (fd)-virus does not present a strong response to electric or magnetic fields.^490^

Finally, capillary forces are among the strongest forces, covering the nanometer to centimeter size range (Figure 12b1).^491,492^ They are described by the Laplace–Young equation, and increase with increasing wettability and confinement. Capillary forces are responsible for interparticle interactions during drying, overcoming the interfacial potential, between particles and with the substrate, leading to assembly into large scale materials.^491^ Shear flow and capillary forces are dominant in wet processing of both micrometric and macrometric particles. For instance, capillary forces are responsible for the strength of a partly dewetted paper during assembly,^493^ and they overcome the interfacial potential during evaporation, resulting in cellulose–cellulose interactions in the formed paper. A similar phenomenon is observed for cellulosic nano- and microfibers. These forces are responsible for the collapse of 3D networks, in precursors hydrogels or liquid foams, which damage their superstructure.

Importantly, going from the macromolecular to the macroscopic forces, those originating from the lower scales influence those at the larger scales. For instance, capillary forces depend on the DLVO interactions and macroscopic shear forces dependent on the same forces acting at the lower scales.

### 4.4 Biopolymer Assembly at Interfaces

Polypeptides, polynucleotides, and polysaccharides with macromolecular backbones fold via self-complementarity and assemble into structures that protect them (at least temporarily) from degradation. Folding of biopolymer structures (e.g., protein globule, DNA double helix, and cellulose fibril) is a vital function in nature as it aids heterogeneous interactions and aids in protecting against hydrolytic environments. Hence, the structure and folding of proteins play important roles, also in assemblies at interfaces. Taking polypeptides as an example, folding can take place on planar lipid surfaces in the form of protein crystallization^494^ or as amyloid fibril formation for many amyloidogenic peptides; this assembly is affected by the physicochemical nature of the surface.^495^ Protein aggregation at interfaces is often accompanied by changes in conformation, since proteins modify their folded structure in response to interfacial stresses (hydrophobicity, charge, mechanical stress). Uncontrolled protein aggregation through adsorption at vapor–liquid, liquid–liquid and liquid–solid interfaces challenges drug manufacturing and targeted functions.^496^

**Figure 13:**
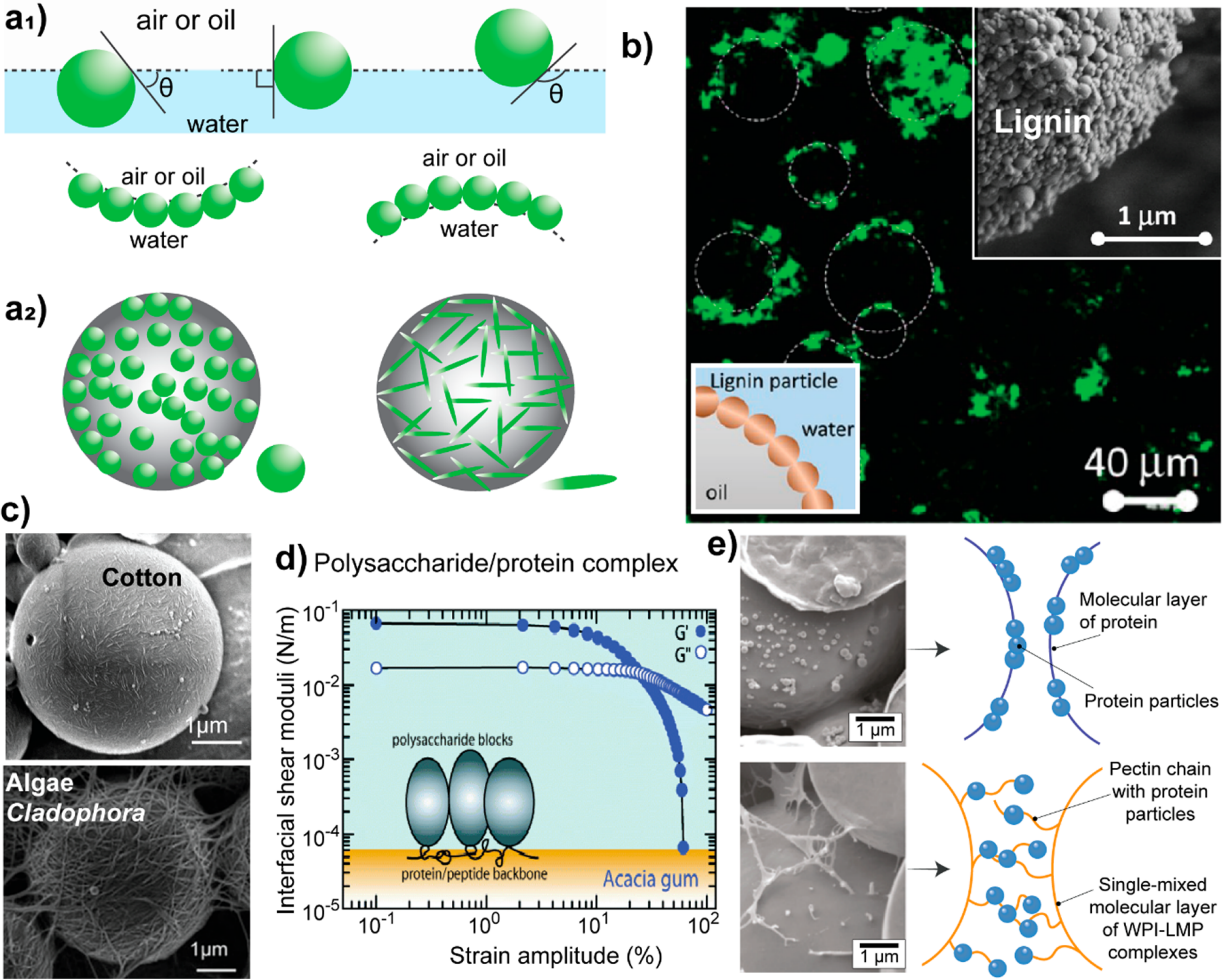
(a1) Particle wettability at the fluid–fluid interface. (a2) Effect of particle shape on the interfacial assembly as demonstrated with isotropic particles (spheres) and rod-shaped nanoparticles (CNC). (b) Autofluorescence image of lignin particle assembly at the oil–water interface in a Pickering emulsion (no fluorescence dye was applied), schematics (down left inset) and scanning electron microscopy (SEM) image (upper right inset). Adapted with permission from ref (452). Copyright 2016 American Chemical Society. (c) SEM images of cotton^498^ and green algae^499^ biocolloids at interfaces. Adapted with permission from ref (498) and ref (499). Copyright 2012 American Chemical Society and. Copyright 2013 The Royal Society of Chemistry, respectively. (d) Acacia gum shear elasticity at the o/w interface. Reprinted with permission from ref (500). Copyright 2007 American Chemical Society. (e) Cryo-SEM images (scale bars 1 μm) of protein particles and whey protein isolate/low methoxyl pectin complex (WPI-LMP) in HIPEs stabilization. Adapted with permission from ref (501). Copyright 2017 The Royal Society of Chemistry.

Colloidal forms of biopolymers and biocolloids stabilize multiphase systems such as emulsions and foams for food, pharmaceutical, cosmetic, and paint applications. In contrast to surfactants, used as typical emulsifiers and foaming agents that adsorb and desorb rather fast, colloids with the right size and wettability remain irreversibly adsorbed.^497^ Therefore, liquid droplets and air bubbles formed under interfacial interaction become kinetically stable. In these systems, particle wettability determines the particle contact angle adopted at the fluid–fluid interface (Figure 13a1).

The behavior of isotropic particles (spheres) at interfaces has been studied comprehensively, whereas that of high aspect ratio CNC and CNF materials are becoming subjects of increasing interest (Figure 13a2). The nature of the surface of nanocellulose is linked to its origin,^10^ for example, in the case of CNFs there are differences in the sulfate ester content, colloidal stability, crystallinity and morphology. These properties should be carefully considered for any use since the cellulose source and isolation conditions (see Section 3.2.3) impact significantly the interfacial properties and behavior. The percolation threshold of nanocellulose is already achieved at low volume fractions, which is beneficial in the stabilization of interfaces.^502^ Since nanocelluloses are available in different shapes, aspect ratios, surface chemistries, and crystallinities, they demonstrate tunable reinforcing behavior at the interface separating two phases.^502^

#### 4.4.1 Biopolymers and Colloids at Liquid/Liquid Interfaces

Emulsions are dispersions of at least two immiscible liquids, typically oil and water, in which one of the liquids forms the dispersed phase, that is, droplets, while the other forms the continuous phase. Surface-active emulsifiers lower the interfacial tension between the dispersed and continuous phases and reduce the droplet size. Particle-stabilized emulsions, or Pickering emulsions, first reported in early 1900s,^503^ are demonstrated for cellulose particles that are partially wetted by water and oil, stabilizing droplet with sizes much larger than the particle size (Figure 13a1).^504^ Hydrophilic particles are used to emulsify oil-in-water systems with the wettability proceeding from the nanoparticle’s contact angle (controlled by the hydrophobic/hydrophilic surface chemistry) at the interface. These emulsions are extremely stable as the energy required to desorb a particle is several thousand *k*_*B*_*T* whereas it is in the order of a few *k*_*B*_*T* for surfactants. Highly anisotropic, rod-like particles organize tangentially at the interface with possible long-range orientation of the rods, which is not possible for spheres; compared to spherical particles, a lower concentration of rod-like particles is used to stabilize the liquid/liquid interfaces.^505^ CNC charge density influences emulsifying properties, for example, CNCs with a surface charge density >0.03 e/nm^2^ are not able to efficiently stabilize as oil/water interface.^498^ Thus, the stabilization capability of CNCs is controlled by salt addition and pH.^506^ The aspect ratio of CNCs varies significantly based on their origin, for example, from 185 nm (cotton-derived) to 4 μm (algae-derived), which affects their networking properties and coverage in o/w emulsions. Higher aspect-ratio CNCs cover only approximately 40% of the droplet, but form interconnected networks, which contrasts the dense organization encountered with high droplet coverage CNCs of smaller aspect ratio (Figure 13c).

ChNFs^308^ (∼10 nm lateral size) as well as ChNCs^507^ can be used to formulate high internal phase Pickering o/w emulsions with an oil volume fraction of up to 88%. Chitin nanoparticles surpass any reported biobased nanoparticle, including nanocelluloses, for their ability to stabilize interfaces at ultralow concentrations (as low as 0.001 wt %) and could potentially fully replace surfactants in multiphase systems.^304^ As a comparison, much higher quantity of nanocrystals sourced from bacterial cellulose (0.2 wt %) is required to stabilize o/w Pickering emulsion.^508^

High internal phase emulsions (HIPEs, oil volume fraction = 0.82) were fabricated using colloidal complexes of preformed whey protein isolate (WPI) and low-methoxyl pectin. Emulsions were produced simply via homogenizing aqueous dispersions of WPI–pectin complexes with sunflower oil in the absence of any low molecular weight emulsifiers yet showed exceptional stability.^501^ The formation of HIPEs is strongly pH-dependent as the colloidal complexes form at specific pH (5.5) (Figure 13e).

Recently, an interesting pH-responsive emulsion system was obtained based on sulfonated lignin.^509^ Nanoparticles were formed from this sulfonated lignin under acidic conditions and formed ultrastable Pickering o/w emulsions by the effect of steric stabilization of oil droplets. At neutral conditions, the dissolved sulfonated lignin acted as a polymeric surfactant and formed emulsions of relatively low stability;^509^ whereas under alkaline conditions the strong hydrophilic interactions prevented emulsion stabilization. At pH 7, sulfonated lignin stabilizes emulsions by adsorption at the oil/water interface and preventing droplet coalescence by electrostatic and steric repulsion.^19^ It has been noted that the size of the macromolecular lignin dispersant (larger molecular weight) has a strong effect on lignin dispersion effect, in which larger molecules are more efficient given their role in steric stabilization.^451^ Wood hemicelluloses also form and stabilize oil-in-water emulsions. Galactoglucomannans from spruce and glucuronoxylans from birch provide multifunctional protection against physical breakdown and lipid oxidation in emulsions.^510^ Interestingly, the combination of lignin with very small amounts of carbohydrates cause the formation of complexes, which affects the overall surface activity of the “lignins” and promotes emulsion stability.^19^ Phenolic residues (coextracted with hemicelluloses using the pressurized hot water process) can deliver and anchor hemicelluloses at the emulsion interface and enhance emulsion stability.^511^

Apart from lignin, an improved polysaccharide emulsifying capability, for example, in case of acacia gum, occurs due to the presence small fraction of surface-active proteins (Figure 13d).^512^ This gum (collected from acacia trees), is one of the most prevalent industrial gums and a hybrid polyelectrolyte containing both protein and polysaccharide subunits (arabinogalactan protein/peptide complex), used mainly as a viscosity enhancer. Acacia gum demonstrates substantial shear elasticity at the o/w interface.^500^ The interfaces covered with the plant gum flow like a rigid, solid-like material with large storage moduli and a linear viscoelastic regime limited to small shear deformations, above which apparent yielding behavior is observed.

#### 4.4.2 Biopolymers and Colloids at the Gas/Liquid and Solid/Liquid Interfaces

Nanocellulose-based foams and aerogels are attractive for a wide range of applications (e.g., biomedical scaffolds, thermal insulation and energy generation/storage) since they combine ultralow density, tunable porous architectures and outstanding mechanical properties.^513^ A nanocellulose-based foam is produced as multiphase porous material (porosity >50%) in which gas (e.g., air) is dispersed in a liquid, solid or (hydro)gel. The diameter of the bubbles (or the pore size, if shrinkage is negligible) is usually larger than 50 nm. The definition of an aerogel differs from foams in that a mesoporous solid material (i.e., pore size in the range 2–50 nm) of high porosity (>90%) is obtained by removing the fluid inside the solvogel precursor while preserving the network structure.^514^ Nanocellulose processing starts with the preparation of a wet foam followed by removal of the solvent by evaporation (xerogel), freeze-drying (cryogel), and supercritical drying preceded by solvent exchange (aerogel). Foams can be also directly obtained from hydrogels or dispersions by, for example, freezing the solvent or from wet form in which gas bubbles are introduced by, for example, vigorous stirring or shaking.

Both CNFs^515,516^ and CNCs^517^ have been studied for their amphiphilicity; however, in unmodified/pristine form, they show no apparent surface activity. Naturally, as nanocelluloses can be modified with polymers^517^ and surfactants^518^ (e.g., octylamine or decylamine)^516^ through noncovalent means or chemically, the surface activity can be induced by tailoring the chemical surface structure. The high aspect ratio of CNFs favors charge-induced stable gel formation of the particle-filled, air–water interfaces which contributes to foam stability. Moreover, the addition of salt increases the adsorption of negatively charged CNFs at the air/liquid interface thereby increasing foam stability, as modified particles prevent flocculation and still induce a repulsive disjoining pressure.^516^ This arises from the screened electrostatic repulsion upon salt addition, which allows a more efficient particle packing at the interface. ChNCs^307^ and CNCs^519^ do not support foaming alone but stable foams can be prepared using Pickering stabilization with surfactants and polymers. The porous structure needs to be preserved during solvent removal for the preparation of nanocellulose-based porous solids with different drying techniques (supercritical drying and freeze-drying) to prevent the deformation or collapse of the porous network.^513^

Lignin has amphiphilic character and the surface functional groups of lignin nanoparticles are related to the assembly strategy. For instance, during lignin nanoparticle formation in aerosol systems, the hydrophobic groups are oriented toward the particle core at the air/liquid interface;^452^ this is in contrast to precipitation strategies in polar solvents.^171^

Beyond liquid/liquid and gas/liquid interfaces, biopolymer assembly at the solid/water interface is relevant to the formation of coatings as well as composites where the solid surfaces consist of nano- to macroscale solid particles. The long-range order of the materials formed at the interface can be enhanced by controlling the capillary forces that develope during drying^491^ or by using specific deposition techniques such as the Langmuir–Blodgett technique, where the air–water interface is first used for the formation of thin films that are then transferred onto a solid support.^478,520^ As the materials consolidate, mechanical parameters become key to control the formation of fractures in coatings as well as the strength and toughness of composites.^479,491^

## 5 Sol–Gel Behavior and Processing

As most biopolymers are hydrophilic and obtained as aqueous solutions or suspensions, their behavior in solution and associated solution-to-gel (sol–gel) transitions define their processability and the preliminary structures formed prior to consolidation. Regarding the latter, the topology of the hydrated, gelled network is largely affected by the dimensions of the building blocks, solvation and their ability to bond with each other in competition with the solvent. Gelation at a given scale is generally identified by rheological measurements, when the storage modulus (*G′*) overcomes the loss modulus (*G′′*) in oscillatory tests. However, these characteristics are not always easily identifiable, for instance as a result of the non-Newtonian response of a given hydrogel. For instance, biopolymers can show strong shear-thinning or shear-thickening responses. As we put forward below, the relationship between their MW, polarity and gel properties is not as straightforward as can be inferred from conventional polymer physics, which is readily applied to many synthetic systems. However, nanofibrous and spherical biocolloids behave more consistently despite their high polydispersity. In this section, we introduce the physics relevant to macromolecular and colloidal solutions and demonstrate discrepancies relative to biopolymeric systems. We then discuss general trends, where engineerability is grounded in theory and associated with fundamental process-related interactions.

Importantly, in the case of dissolved biopolymers, a relatively low volume fraction leads to the formation of poorly processable gels. At high volume fractions they can be processed as biocolloids. For instance, dissolved cellulose can be processed at a maximum of 18 wt %, while CNCs can be processed at least until 60 wt %.^485,521^ As a result, a variety of combinations and associated 3D topologies can be obtained through controlling sol–gel transitions using solely biopolymers. The properties of the biopolymeric materials and the sol–gel processing steps are quintessential since the structures obtained in the gelled state determine those in the consolidated form.

### 5.1 Gelation Associated with Overlapping and Crowding

For fibers, particles, or dissolved polymers, gelation may occur strictly based on solution crowding. The number of entanglements or contact points increases with concentration and the size of the building blocks, which typically leads to the system’s ability to act as a gel at time scales smaller than the lifetime of the entanglements. As a general rule in good solvents, such systems transition from a dilute to semidilute regime at the overlap concentration (c*), where the total (dilute) pervaded volume of the solute equals the solution volume.^522,523^ The polymer entanglement begins typically at an elevated concentration thereafter (*c*_e_ > c*). Similar transitions exist for rod-like particles in response to suspension crowding (explained further below).^524,525^

**Figure 14:**
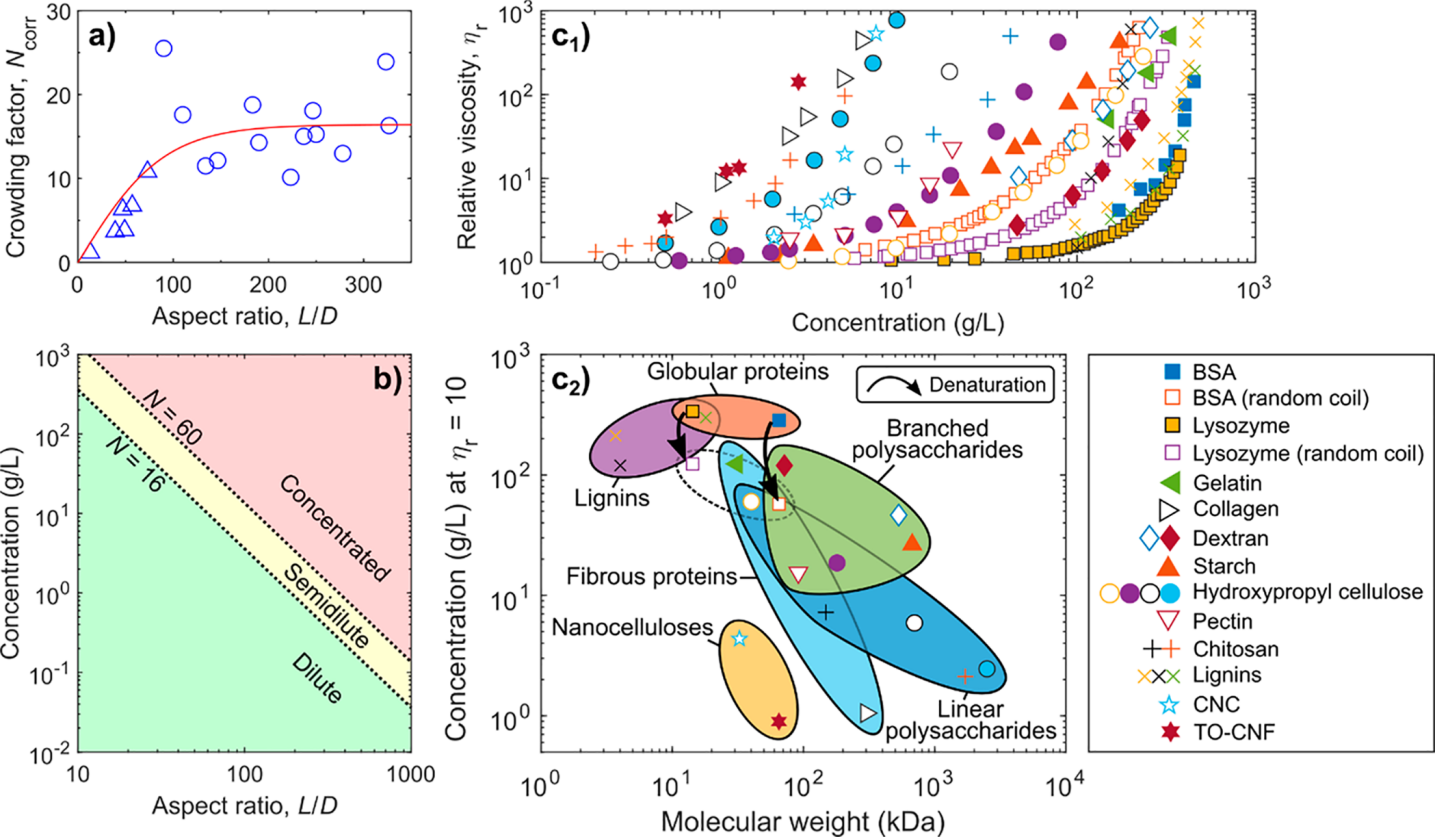
(a) Literature values for the connectivity threshold of CNC^544,545,553,554^ and CNF^545−548,551^ suspensions plotted using the corrected crowding factor, *N*_corr_, with a fit of the form *N*_corr_ = *A*/(1 + *e*^–*B*(*L*/*D*)^) – *A*/2; *A* = 32.85 and *B* = 0.02234 (*R*^2^ = 0.5394). (b) Suspension concentration and particle aspect ratio (*L*/*D*) regions corresponding to the crowding factor (*N*) transition values for the dilute (*N* < 16, density ρ = 1.5 g/cm^3^), semidilute (16 < *N* < 60), and concentrated (*N* > 60) regions.^524^ (c1) Zero/low shear rate relative viscosity (η_r_) of biopolymer solutions^529−531,555−566^ and colloidal^553,567^ suspensions at or near room temperature as a function of concentration. (c2) Concentrations corresponding to η_r_ = 10 are shown as a function of molecular weight determined from (C1) using log-scale linear interpolation. The molecular weight of cellulose constituting CNCs was estimated from particle length, while for TO–CNF the estimation was based on Shinoda et al. (2012).^568^

Hence, the viscosity of biopolymer solutions greatly vary depending on their conformation, molecular weight, topology, and solids content, as well as ionic strength and solvent quality (Figure 14b,c). The more compact conformation of branched polymers,^528^ such as dextran or hemicelluloses, result in the onset of entanglement and solution viscosity at higher concentrations compared to linear polysaccharides.^529^ Similarly, the highly compact secondary and tertiary structure of globular proteins, such as lysozyme and bovine serum albumin (BSA), affords processing at very high concentrations. For example, BSA is soluble in water up to around 585 g/L,^530^ yet once denatured, by breaking their intrachain disulfide bonds and bringing them into a random coil conformation, they develop a similarly high viscosity compared to that of fibrous proteins and linear/branched biopolymers (Figure 14c2).^531^ Less severe, reversible conformational changes and dynamic cluster formation occur at high solids content with some proteins that underpin their viscosity and functionality in the highly crowded macromolecular environments of biological systems that they operate in.^532−537^

In comparison, the rheological behavior of rod-like particles in suspension is influenced by their rotational motion which results in a viscosity that depends on the particles’ length-to-width aspect ratio (*L*/*D*) and distribution, in addition to volume fraction.^538,539^ In dilute conditions, the free rotational motion of a rigid rod-like particle takes up a volume of the size of a sphere with diameter *L*. The average number of particles in this suspension volume is given by the crowding factor (*N*). In the simplest case of monodisperse particle size, the crowding factor is given by *N* = 2/3ϕ(*L*/*D*)^2^ at the volume fraction ϕ.^524^ A polydisperse particle size distribution leads to underestimation of *N* that can be remedied by using the log-normal corrected form, *N*_corr_ = *N*(1 + CV^2^)^4^, where CV is the coefficient of variation of the length distribution (used to calculate the data presented in Figure 14a).^541^ The influence of surface charge and ionic strength on the effective dimensions of the particles has to be considered as well.^538,542^

Rheological measurements with CNC^543−545^ and CNF^545−549^ suspensions indicate that the transition from dilute to semidilute condition occurs at the connectivity threshold or gel crowding factor, roughly at *N*_*corr*_ = 16, especially when *L*/*D* > 100 (Figure 14a),^525^ which corresponds to the point when the storage and loss modulus become equal.^546,551^ The flocculation tendency is decreased with a smaller aspect ratio and higher surface charge, due to electrostatic repulsion.^546^ At the rigidity threshold, the system transitions from semidilute to concentrated whereby each particle is surrounded by neighboring particles, effectively arresting the suspension and forming a rigid, percolated structure. This takes place at roughly *N* = 60, which corresponds to three interparticle contacts at sufficiently high aspect ratios.^524,525,552^ Due to their high aspect ratio, nanocelluloses and nanochitins effective gel at low solids content (compare with Figure 14c).

### 5.2 Liquid Crystalline Phase Transitions

Liquid crystalline phase transitions are associated with the anisometry of the building blocks. In the case of biopolymers, this includes oblong stretched coils of dissolved polymers, and rod-like rigid as well as nanofibrous building blocks. Principally, nematic and chiral-nematic liquid crystals can be achieved from dispersions of biocolloids or biopolymers, which transfer to the materials they form. These long-range orders correspond to unidirectional alignment and to alignment within loose planes, with each pseudoplane being rotated partially into a helicoidal arrangement along a principal director, respectively. As described by the Onsager theory, and in further corrections in other studies for a range of anisometric colloids, lyotropic liquid crystalline phase transitions are bound to occur in dispersions of anisometric biocolloids, above a given volume fraction.^475,569,570^ This phenomenon occurs prior to kinetic arrest or gelation and depends on geometry, and conformability as well as surface charge.^569,571,572^ The impact of these factors has been highlighted comparing the assembly of biocolloidal nanofibers, mainly tobacco-virus fragments, engineered amyloid fibrils, and nanocelluloses.^572,573^ For CNCs of similar dimension, a lower surface charge leads to a decreased volume fractions for the onset of liquid crystalline phase transitions.^571^ For instance, decreasing the charge density of CNC by 3-fold reduces the concentration for the onset of anisotropic phase transitions, by 23-folds (from ca. 3.4% to 0.15%). Interestingly, the range of concentrations at which liquid crystalline phases are observed was not significantly influenced, 0.2–1.0% and 3.4–4.4%, respectively. Higher aspect ratios lead to a lower concentration required for phase separation and a proportionally lower gelation concentration. Using CNC as a reference, doubling the length and anisometry, doubled the concentration at which liquid crystals formed. Furthermore, the smaller the building blocks, the higher the concentration for phase separation and the larger the window between anisotropic phase separation and gelation.^572^ Typically, lower concentrations for proteinaceous liquid crystals (<0.5%) are observed for the onset of liquid crystallinity compared to nanocellulose-based transitions as a result of larger anisometric dimensions and higher charges. The surface tension of the interface between anisotropic/isotropic phases of biopolymers or biocolloids is rather small (<0.1 mN m^–1^),^574−577^ with higher values observed for biocolloids. Liquid crystalline spherulites,^594^ that is, tactoids, transform first into nematic phase and may rearrange and coalesce into chiral-nematically ordered tactoids, if the building blocks bare chirality within the relevant scale. Tactoids then merge into a continuous domain into the anisotropic phase by external stimuli or by equilibration of the suspension at a concentration below gelation, leading to uniform domains.^578−580^

Dissolved biopolymers such as peptides,^581^ nucleic acids,^582,583^ or dissolved cellulose and its derivatives were studied in the context of lyotropic liquid crystals,^584^ that is, concentration-dependent phase transitions.^585^ The vast majority of studies are dedicated to cellulose derivatives. In contrast with biocolloids, some of the liquid crystals from dissolved cellulose showed thermotropism associated with their decreased solubility at higher temperature.^586,587^ For cellulose derivatives, liquid crystal phase transitions were shown to be tied to the rigidity and the associated persistence length of the polymer. Substitution can decrease the rigidity of the polymer if the substituent does not present a high steric bulkiness.^585^ MW and DS play a significant role in the development of the liquid crystalline order and, uniquely among biopolymeric systems, its handedness or chirality.^588,589^ The most studied liquid crystalline cellulose derivative is hydroxypropyl cellulose (HPC). HPC with DP of ∼500 shows an onset of anisotropic fraction formation starting from a polymer concentration of 20 wt %.^590^

**Figure 15:**
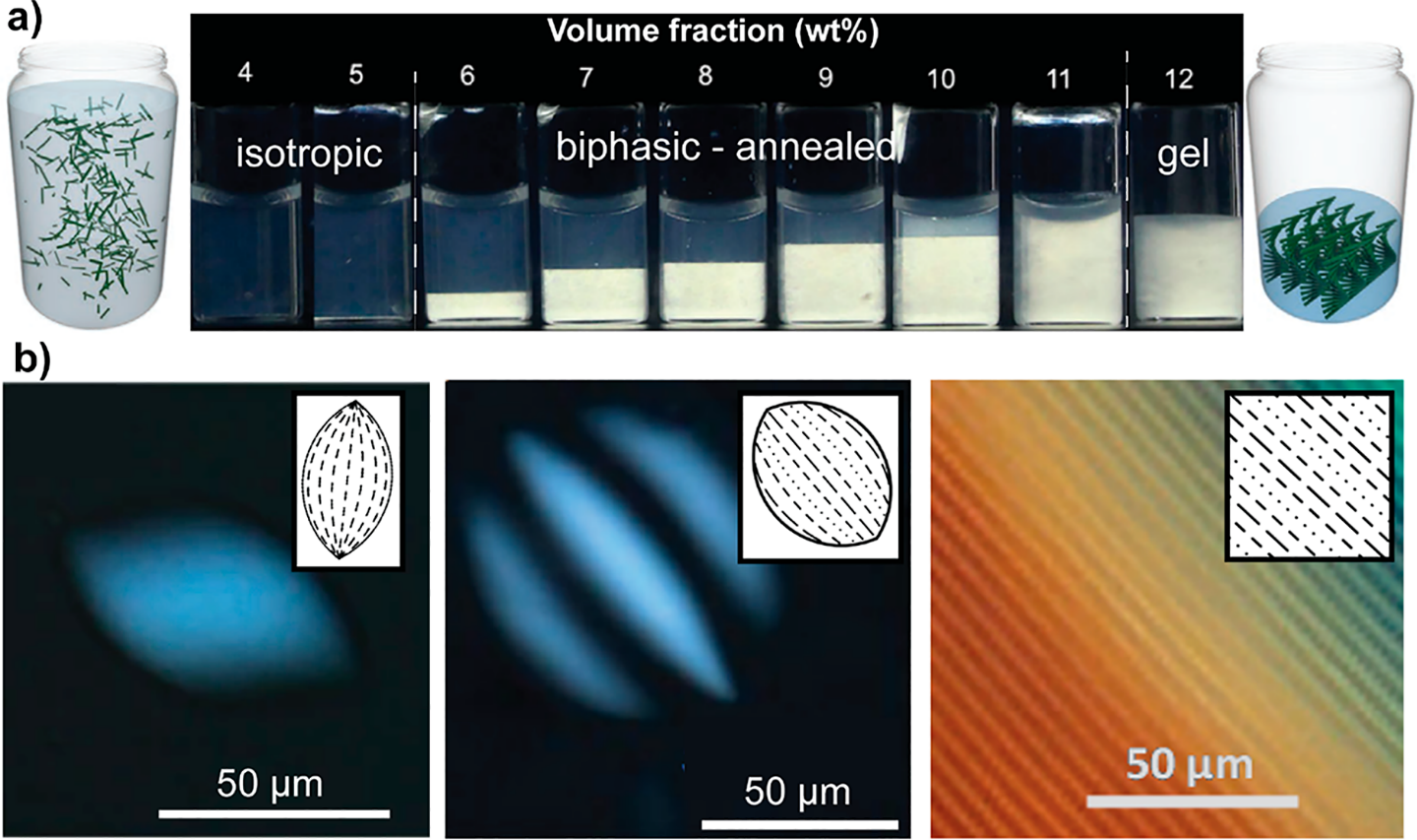
(a) Lyotropic liquid crystalline transitions of biopolymeric colloids, such as CNC, as a function of concentration. The left- and right-hand side schematics illustrate the disordered and annealed liquid crystals, respectively. Adapted with permission from refs (596 and 603). Copyright 2018 The Authors and Copyright 2020 John Wiley and Sons. (b) From left to right, nematic and chiral nematic tactoids, and an annealed chiral-nematic phase as observed between cross-polarizers by optical microscopy. The biocolloids are amyloid fibers and CNCs (right panel). The left and center panel in (b) are adapted with permission from ref (570). Copyright 2018 The Authors. Right panel in (b) is adapted with permission from ref (604). Copyright 2014 American Chemical Society. Note: Further permissions related to the material utilized in (b) (far right) (pubs.acs.org/doi/10.1021/la501741r) should be directed to the American Chemical Society.

The liquid crystalline behavior has been extensively studied for protein biocolloid such as tobacco virus^591^ and amyloids^570^ as well as polysaccharide nanoparticles, nanochitins,^592^ and nanocelluloses.^572^ In the case of protein assemblies, the principal constructs studied are tobacco virus and man-made amyloids obtained, for instance, from lactoglobulin or lysozyme, with amyloid fibers being heavily studied in recent years. They offer a wide range of prospects due to engineerability of the protein building blocks and the various liquid crystalline structures available (Figure 15b).^570,593^ Despite being reported only recently, they bear the most varied cholesteric phases shown thus far for biobased building blocks and hint at a bright future to engineer material properties through superstructures.^513,537^ Interestingly, prior to these recent advances, proteinaceous liquid crystals were exclusively reported for their nematically oriented phases, while polysaccharide-based liquid crystals were principally observed to undergo chiral-nematic phases. The large-scale production of CNCs, paralleling the emerging use of nanochitins, enable a deeper understanding of biobased liquid crystals suspension, gelation and consolidation behavior.^592,595^ The assembly into chiral-nematic order takes place prior to gelation (Figure 15a). In the dispersed state there is no strong evidence of an intermediate nematic arrangement, prior to the formation of chiral-nematic domains; however, shearing the gelled dispersion does result in nematic arrangement of CNCs, even if gelation and further consolidation without shearing conserves the chiral-nematic order.^485,596^ In the case of biobased colloids, the high polydispersity is an impactful factor:^597^ longer CNCs form anisotropic domains at lower concentrations with a larger pitch.^572^ Thereafter, the various phases of the system enable separation by size, both of the original mesogens and of added nanorods (e.g., rod-like gold particles).^596,598^ The phase transitions have been studied and highlight the formation of a two-dimensional (2D) packing of the rods, within the chiral nematic phase, followed by 3D packing.^599,600^ Furthermore, the liquid crystalline architecture can be modified in the dispersed-state by external factors such as magnetic and electric fields, or by the addition of (macro)molecular additives.^601−603^

To date, combined with other self-assembly efforts, liquid crystalline processing is the only approach to obtain advanced architectures tailored at the nanoscale. Therefore, for all biomimetic endeavors encompassing multiscale hierarchical architectures, control over the liquid crystalline properties of dispersions plays a significant role.

### 5.3 Polymeric and Colloidal Gelation

Loosely speaking, a gel can be taken as an intermediate state in the consolidation of biopolymers where the rheology and gelation kinetics play a critical role in the process and in defining the properties of the final system. A fundamental understanding of the mechanisms of gelation of biopolymers and biocolloids is addressed in this section. Recognizing the importance of organogels and oleogels, most of the present discussion relates to hydrogels given that water is the most prevalent media for biocolloids. Polymeric and colloidal gels present singularities, for example, reversibility, responsiveness, etc., that drive efforts in the exploration of supramolecular/supracolloidal interactions, for instance, to enable and control gelation. The most important physical (i.e., noncovalent) means of cross-linking biopolymer and biocolloid gels are discussed below.

#### 5.3.1 Electrostatically Driven Gelation

##### 5.3.1.1 Gelling Induced by Matching Electrostatic Interactions

Sol–gel transitions involve intermolecular or interparticle association and are notably affected by the presence of ionic or ionizable groups. While on one hand repulsive interactions are promoted within same-charge entities, due to electrical double layer overlap, on the other hand, these groups may enable complexation among molecules or particles bearing opposite electrostatic charges. Both systems are prone to gelling, albeit through different mechanisms, as developed in this section. In the case of polyelectrolytes, intramolecular electrostatic repulsion drives conformation toward more extended chains when compared with the more conventional coiled conformation,^605^ which may undergo a sol–gel transition either via associative or segregative means.

**Figure 16:**
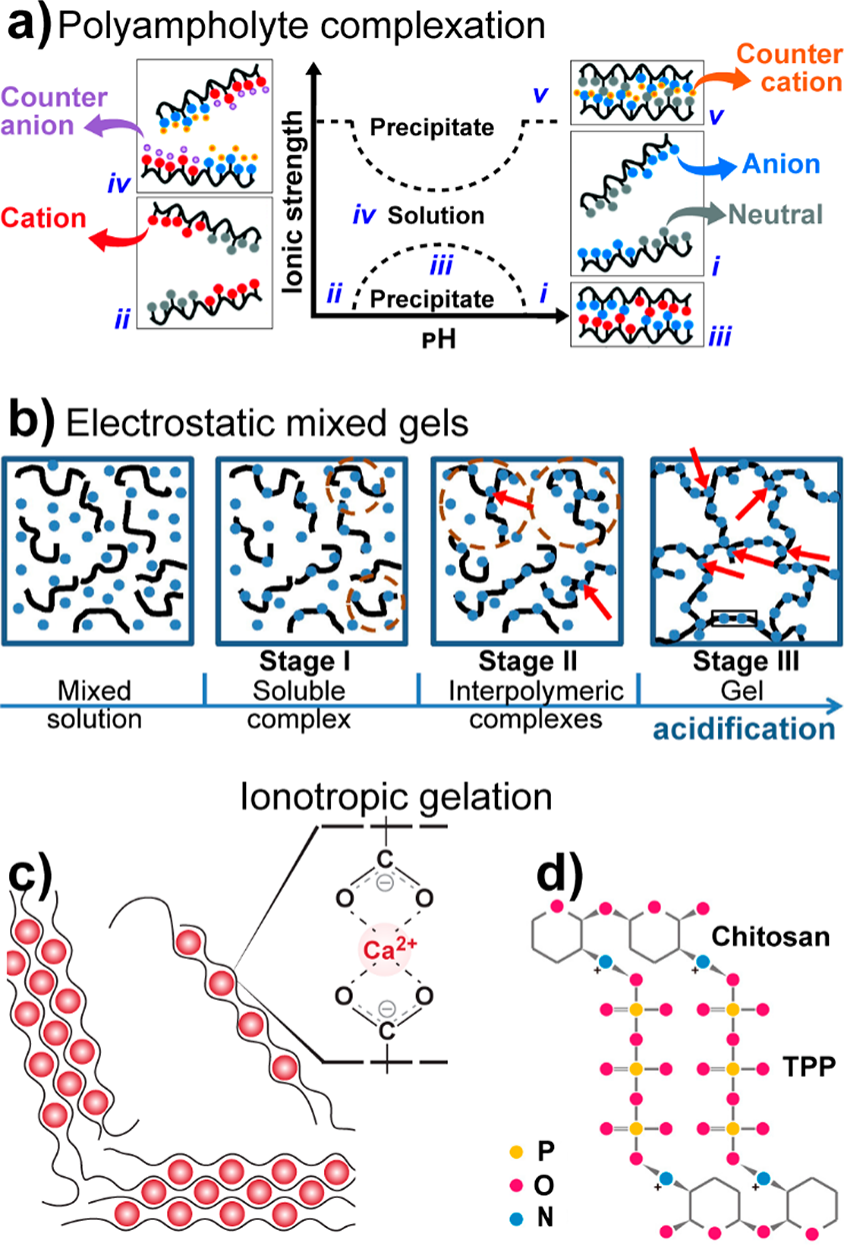
Electrostatic means of gelling biopolymers and biocolloids. (a) Phase diagram of a *quasi*-equimolar cationic/anionic weak polyampholyte in solution at varying pH and ionic strength.^610^ Adapted with permission from ref (609). Copyright 2019 The Royal Society of Chemistry. (b) Proposed three-stage mechanism of electrostatic mixed gel formation kinetics upon acidification under quiescent conditions, with blue dots and black chains representing a protein and an anionic polysaccharide, respectively. Red arrows highlight junction zones/electrostatic cross-linking points (wherein more than one polysaccharide chain may share the same protein domain), and the rectangle indicates an aggregation zone. Adapted with permission from refs (605 and 613). Copyright 2013 The Royal Society of Chemistry and Copyright 2016 Elsevier B.V. (c) Proposed scheme of a typical “egg-box”-like structure formed when an anionic macromolecule, such as alginate, percolates and gels in the presence of divalent cations like Ca^2+^, which serve as intermolecular ionic bridges, or electrostatic cross-links (see inset). (d) Schematic illustration of intermolecular bridging of chitosan through the electrostatic interaction among its protonated primary amine groups (blue circles) and the anionic ends (pink circles) of tripolyphosphate (TPP). Adapted with permission from ref (614). Copyright 2020 The Authors.

Electrostatic associative gels are formed via complex coacervation when the complexing species are thermodynamically compatible with the resulting ordered domains termed coacervates. Even if extensively attributed to the Coulombic attraction between matching charged species, coacervation is further driven by another enthalpic contribution arising from local water perturbations, as well as by entropy via counterions that are released upon binding into a complex.^606^ The free energy of complex formation is primarily enthalpy driven for weak polyelectrolytes (i.e., polyions bearing pH- and ionic strength-dependent degrees of ionization) and entropically driven for strong polyelectrolytes (i.e., completely ionized in solution).^607^ Intuitively, this type of gelation is more common in mixed gels, wherein more than one ionic species, commonly a polycation/polyanion pair, meet in solution or suspension and form gels in conditions under which these would not be expected to gel individually. For instance, at low solid contents and in the absence of thermal denaturation. This concept applies to mixtures of a range of natural biopolymers involving charged proteins (e.g., bovine serum albumin, caseinate, gelatin, lysozyme, and β-lactoglobulin) and polysaccharides (e.g., alginate, chitosan, gellan, pectin, xanthan, and κ-carrageenan).^605,608^ Exceptions to mixed gels include a specific class of zwitterionic polymers that carry both anionic and cationic groups, namely polyampholytes and polybetaines (depending on the arrangement of the charged groups along the chain), which may be prone to self-coacervation depending on the chemical structure and the environment.^609^Figure 16a presents the typical behavior of a weak polyampholyte in solution:^610^ at low ionic strength, high (*i*) or low (*ii*) pH prevents complexation due to electrostatic repulsion among the negatively and positively charged groups, respectively, while the other groups are neutralized (deprotonated and protonated, respectively); at intermediate pH (*iii*), close to the isoelectric point, a precipitate is formed owing to the electrostatic coupling of the oppositely charged groups, while mild salt addition (*iv*) favors solubilization due to the screening of the Coulombic attractive forces, with repulsion prevailing (salting in); further addition of salt diminishes the repulsive forces and leads to a new precipitation at sufficiently high ionic strength (*v*), where non-Coulombic attractive forces dominate (salting out). Enzymes and other proteins such as gelatin are examples of naturally occurring polyampholytes. Self-coacervation is also present in proteins involved in mussel’s underwater adhesiveness^611^ as well as in modular squid beak proteins.^612^

As opposed to solid–liquid two-phase systems, where the solid phase is a dehydrated precipitate, formation of coacervates results from the associative phase separation into two liquid phases in thermodynamic equilibrium: a biopolymer-rich phase (coacervate) and a biopolymer-poor analogue (supernatant), representing a hydrated gel-like complex.^609^ The water holding capacity of electrostatic mixed gels is outstanding, potentially reaching several hundred grams of water per gram of dry biopolymer. Swelling depends on the pore size and therefore capillarity, which is higher for smaller pores according to the Young–Laplace theory. Pore size, as well as other characteristics of electrostatic gels, can be tuned for a given polyelectrolyte pair by varying the solids content, polycation/polyanion ratio, charge density and ratio, temperature, ionic strength and pH.^605,608^ For a pair of oppositely charged entities an optimum charge balance exists (affected by virtually all of these factors) between attractive and repulsive interactions. In turn, these can lead to the most mechanically robust gels; considering that too strong associative interactions might result in spontaneous syneresis, that is, contraction of the gel. Importantly, a given system may phase separate either associatively into physical composite gels, as in the coacervate/supernatant system described above, or segregatively, with each phase being enriched in only one of the biopolymers. The latter includes the aqueous two-phase systems (ATPS) and is often true for like-charge polyions (e.g., an anionic deprotonated polysaccharide and a protein at a pH greater than its isoelectric point).^615^

Mixed/coupled gels are advantageous from several perspectives, one being the low solid content required for gelation. This is enabled by phase separation and hence solid content increases in the biopolymer-rich phase and causing gelation. Proteins and polysaccharides often act synergistically in this regard. Whey protein, for instance, has been demonstrated to gel at 8 wt % upon the addition of 1% pectin at pH 6, a condition at which the same solid content of protein alone would not gel.^616^ One may claim that simply increasing the solid content is a straightforward means of gelling biopolymer solutions or suspensions, but actually concentrating the system is not a universal solution to gel single-component systems. This is true in the cases of xanthan gum (XG) and λ-carrageenan, two nongelling polysaccharides, whose combination with proteins denotes a prerequisite for gelation.^605^ Relying upon one of these combinations (β-lactoglobulin/XG), a generic mechanism has been proposed for the evolution of protein/polysaccharide mixed gels upon acidification at quiescent conditions (Figure 16b). Addition of acids is indeed the most common trigger of gelation in these systems because most natural polysaccharides bear negative ionic charge. Addition of bases are to be used when involving a cationic polysaccharide, such as chitosan, where complexation occurs at a pH higher than the isoelectric point of the protein. Electrostatic complexation will be more likely to take place at a pH value that is intermediate to the p*K*_a_ and isoelectric point of complexing polysaccharide and protein, respectively. As the pH approaches the isoelectric point of β-lactoglobulin upon acidification of a mixed solution with XG, soluble complexes (Stage I, Figure 16b) are formed by the interaction between the positively charged patches of the protein and the deprotonated carboxyl groups of the polysaccharide. Additional acidification promotes further complexation toward reducing net charge as well as aggregation, through junction zones of soluble complexes into interpolymer complexes (Stage II, Figure 16b). Under quiescent conditions, these complexes may percolate and undergo a sol–gel transition, leading to a gelled network with electrostatic cross-links (see arrows in Stage III, Figure 16b).

##### 5.3.1.2 Gelling Induced by Specific Ion-Binding Affinity

As developed elsewhere in this contribution, electrically charged biocolloids are stabilized in suspension by the repulsive forces arising from, for example, sulfate half-esters of sulfuric acid-hydrolyzed CNCs, carboxyls in TEMPO-oxidized nanocelluloses, or primary amino groups in partially deacetylated nanochitins. Gelation of fully soluble polyelectrolytes may be prevented by Coulombic repulsion arising from like-charge groups. In this sense, shifting pH or adding salt allows a more intimate intermolecular or interparticle association through varying principles (e.g., hydrophobic interaction, hydrogen bonding, coordination or physical entanglement), denoting a common strategy to gel ionic biopolymers and biocolloids. Because the gelling principle in these cases is not purely electrostatic in nature, they are addressed herein.^617^ A particular case of electrolyte-induced gelation results from the ability of electrically charged biopolymers and biocolloids to percolate with multivalent counterions acting as ionic cross-linking points within a gelled meshwork. The gelation mechanism driven by this specific ion-binding affinity of polymers is commonly referred to as ionotropic gelation and results from the selectivity of charged groups in the biopolymers/biocolloids to complex with metal ions. When deprotonated, the carboxyl groups in TEMPO-oxidized cellulose and alginic acid, for instance, show strong affinity to transition metal cations.^618,619^

While ionotropic gelation may share the same roots with the aforementioned monovalent salt-induced gelation, via ionic screening, multivalent ions have been further demonstrated to serve as intermolecular ionic bridges in gels made up of soluble biopolymers or suspended biocolloids bearing opposite charge compared to the ion. This behavior deviates from the well-founded polyelectrolyte theories, with the most established examples being the gelation of alginate and pectin in the presence of divalent calcium ions: Ca^2+^ ions interact selectively with the α-l-guluronic acid and β-d-mannuronic acid residues of alginate and with the d-galacturonic acid residues of pectin, creating “egg-box”-like structures (Figure 16c).^608^ This specific ion-binding mechanism leads to an ordered structure with high periodicity, resembling a coordination complex, though electrostatic or double-layer interactions may also be at play.^620^ While readers are referred to Section 5.3.2 for further details on metal coordination, the alginate’s egg-box case is discussed in this section as it is not a classical coordination chemistry. Ca^2+^ ions have been demonstrated to induce the gelation of carrageenan, with changes in the secondary structure from random coil to single helix and its further gelation through supercoiled and coiled-coil helix secondary and tertiary structures, respectively.^608^ Furthermore, Ca^2+^ and other multivalent cations have been extensively investigated for the interparticle bridging of sulfuric acid-hydrolyzed and TEMPO-oxidized nanocelluloses.^621−624^ While the structure and properties of TO–CNF gels have been shown to depend on the carboxylate-to-cation binding energy, which in turn depends on valence,^624^ the selective bridging mechanism is still in debate. Some groups demonstrated that cation valence and radius either have little or no effect on gel strength, while divalent cations induce gelation at lower polymer concentrations compared to their monovalent counterparts.^622^ In fact, the critical aggregation concentration is expected to be lower for higher counterion valence, in accordance with the Schulze-Hardy theory, which has been validated for sulfated CNCs.^472^ Finally, in an analogous approach, though with opposite charge, chitosan has been gelled electrostatically via the interaction of its protonated primary amine groups with COO^–^ groups of sodium citrate and P_3_O_10_^5–^ groups of sodium tripolyphosphate,^617^ leading not only to gels, but also to nanoparticles with tunable size and stability (Figure 16d).^625,626^ Since chitosan is a weak polyelectrolyte, its degree of ionic cross-linking can also be modulated by simply changing pH.^617^

##### 5.3.1.3 Non-Electrostatic Association Induced by Physical Confinement and Charge Reduction

Hydrogen bonding is by far the most important non-Coulombic mechanism of gelation, as intra- and interchain hydrogen bonds are ubiquitous in biopolymers and biocolloids.^148^ This particular dipole–dipole interaction is largely responsible for several of the physical and chemical features of biorenewables in the solid state. This includes, to higher or lesser extents, recalcitrance, hydrophilicity, cohesion, infusibility and semicrystallinity. Hydrogen bonding is also of utmost importance for solubilization as the hydrogen bonding network must be overcome by the solvent in biomass and for the (re)establishment of the intermolecular or interparticle hydrogen bonding network in dispersed solutions. Unlike the long-range Coulombic forces addressed previously, hydrogen bonds involve shorter distances, a condition that is not always met in solution or suspension, at least in a dilute state. Any effort to reduce the distance between soluble chains or suspended particles is therefore important for gelation.

Solid content is a central parameter in hydrogen bonded gel networks, which can be lower than 1% for agar after its hydrogen bonding-driven aggregation and network formation.^605^ The gelling of aqueous biocolloid suspensions may be trigged by different stimuli, one of which being high energy input.^617^ This has been demonstrated in the ultrasound-assisted gelling of aqueous ChNC suspensions above the percolation threshold,^627^ in a process relying on the energy transfer from the ultrasound source to the particles through cavitation. This phenomenon provides energy in the same order of magnitude as hydrogen bonds (<100 kJ mol^–1^).^627^ Such sonochemical approach, which has been demonstrated to trigger nanostructural reorganization in bacterial cellulose,^628^ gelled nanochitin through rearrangements of the hydrogen bonding network, enabling the formation of a 3D-percolated network of rigid whiskers made cohesive by the hydrogen bonds themselves.

Solids in solution or suspension can be forcibly bundled together by increasing the solid content until they percolate or entangle into a gelled network. This can be achieved by the high capillary stresses faced during convective drying, or by, for example, freezing, centrifuging or solvent exchange. Freezing has been extensively used to induce gelation, as biopolymeric solids are excluded from the freezing front and end up being physically confined in the intercrystalline domains. The ice crystals are then removed by a suitable method, such as thawing or freeze-drying. Such an approach applies not only for water (hydrogels), but also for polar organic solvents, including DMF and DMSO (organogels).^629^ In principle, it also applies for nonpolar organic solvents, but most biopolymers are hydrophilic and show limited dispersibility in these systems. Freeze–thawing has been used to gel numerous polysaccharides^608,630^ via hydrogen bonds introduced in junction zones. The properties of the resulting gel, particularly the rheological behavior, can be modulated by the ice crystal nucleation and growth kinetics, as shown for CNC hydrogels.^629^

Unless covalently cross-linked by glutaraldehyde, the gelation of PD-ChNF suspensions was prevented by the longer-range electrostatic repulsion among the cationic particles when compared to hydrogen bonds, which were only allowed once the particles were forced against each other by a freezing/thawing cycle.^303^ Likewise, numerous charged biopolymers and biocolloids are not allowed to gel as this process is prevented by Coulombic repulsion arising from like charges. Instead of physically buckling chains or particles together, simply eliminating the factors that prevent them to come close to each other denotes another important means of triggering gelation. The electric repulsion barrier between like-charge entities may be relieved and their surface potential made no longer repulsive by having electrolytes compress the electrical double layers (EDL), thus favoring hydrogen bonding or hydrophobic interactions.^608^ Whereas salt-induced gelation is valid for entities showing strong and weak polyelectrolytic nature, the latter (higher occurrence among biopolymers) is also susceptible to pH-induced gelation as their protonation/deprotonation state, and therefore charge density, is tailored by the proton activity. Indeed, a similar ChNF suspension that would not gel as indicated above was instantaneously converted into a freestanding hydrogel without any forced confinement by simply adjusting the originally acidic 0.5 wt % system to pH 10–11 with sodium hydroxide.^632^ On similar vein, the gelation of CNFs has been extensively reported upon acidification.^480^ Note that the pH-driven gelation in this case goes in the opposite direction of that used to activate ionic groups (electrostatic gelation).

**Figure 17:**
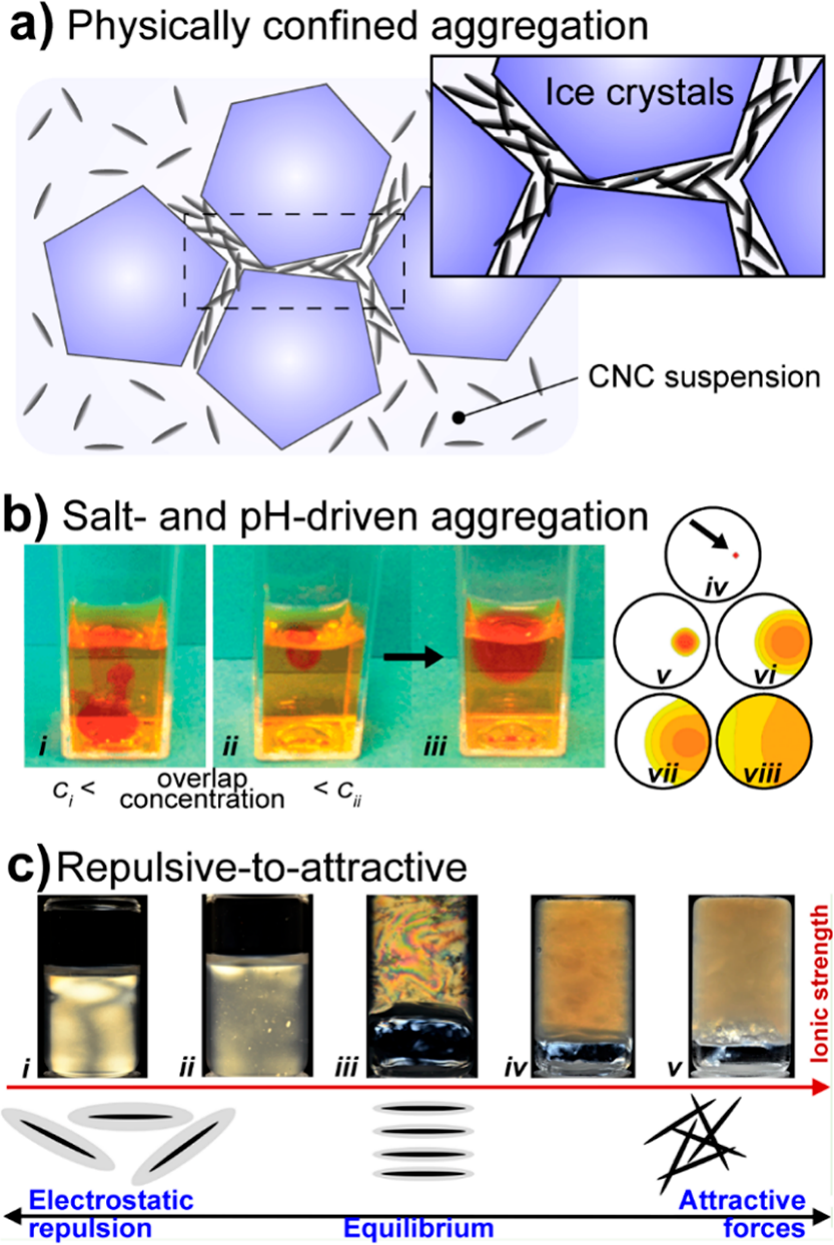
Gelation of biopolymers and biocolloids through hydrogen bonding. (a) Physical confinement of CNCs by exclusion from growing ice crystals from an aqueous dispersion, inducing aggregation (red circles) that is maintained upon thawing or freeze-drying. (b) Drops of 0.1 mM HCl acid solution just added into CNF suspensions below (i) and above (ii) the overlap concentration and containing methyl-orange as pH indicator (dark orange at pH < 4), clearly demonstrate, respectively, the unstable (*c*_*i*_ = 0.05 g L^–1^) and stable (*c*_*ii*_ = 1.5 g L^–1^) acid front spreading, the latter evolving gently during 5 min (iii) as the protons diffuse through the CNF suspension, inducing further gelation. A similar procedure was carried out with a drop of an NaCl solution (iv, injection point indicated by the arrow), which according to theoretical predictions gelled the surroundings as the ions diffused for 201 s (v), 34 min (vi), 56 min (vii), and 224 min (viii). The slower electrolyte-induced gelation kinetics reflects the lower diffusivity of sodium ions compared to protons. Adapted with permission from ref (631). Copyright 2013 The Royal Society of Chemistry. (c) Images of 4 wt % CNC suspensions between cross-polarizers added by 0 (i), 1 (ii), 2.5 (iii), 5 (iv), or 10 mM (v) of CaCl_2_ and schemes of the nanorods (black) and their respective electrical double layers (gray) proposing the mechanism for the evolution from a suspension stabilized by electrostatic repulsion to a laterally oriented nematic gel (note the birefringence in (iii) and an isotropic gel (v). Adapted with permission from ref (622). Copyright 2017 American Chemical Society.

The acid- and salt-driven gelation of carboxymethylated CNF dispersions were demonstrated to occur at higher rates with HCl due to the much higher diffusivity of protons from added HCl (Figure 17b).^631^ Fibril crowding must be ensured at solid contents above the overlap concentration in order to percolate, but in this case, crowding is not per se enough for gelling. More importantly, a low-repulsion regime must be achieved, for example, by charge screening for new hydrogen bonds to form. Electrostatic repulsion typically renders nanocellulose dispersions in water colloidally stable (Figure 17c,*i*). The case of CNFs is similar to CNCs, except that highly charged rods have “excluded volumes” that are larger than their actual volumes (Figure 17c,*i*) owing to the osmotic pressure that arises from the charge-induced EDL.^621,622^ Depending on the concentration and ionic strength, CNCs can self-assemble into a nematic phase. The ionic strength will directly affect the effective biocolloidal diameter, whose mismatch with the actual diameter also indicates that of the excluded and actual volumes and, therefore, the radius of the EDL (i.e., the Debye length [nm]).^622^ As salt is added, the Debye length is gradually reduced, leading first to the nematic association of the CNCs (Figure 17c,*iii*) and finally to gelation (Figure 17c,*v*) once attractive forces (e.g., hydrogen bonds, vdW interactions, depletion forces and hydrophobic interactions) dominate.^622^ Even if CNC gelation in the presence of calcium ions has been attributed to interparticle ionic bridging, evidence show that it is rather independent of cation valence and determined by the increased intermolecular attraction mainly due to hydrogen bonding between hydroxyls or carboxyls (in the case of carboxylated CNCs), which are in turn enabled by charge screening.^621,622^ Notably, adding 25 mM NaCl to the CNC system at 4 wt % shown in Figure 17c increased the dynamic moduli by two orders of magnitude. A comparable effect can also be matched by concentrating the suspension, but requires a high solid content of ∼10 wt %, confirming the efficiency of salt in inducing gelation at lower solid content.^622^ The cation valence is therefore important, besides the quantity of added salt, as the same gelling effect is observed for lower quantity of multivalent ions than their monovalent analogues.^622^ The critical aggregation concentration has been demonstrated to be smaller for higher counterion valence and, for ions with the same valency, for higher ionic radii.^621,633^

Finally, the repulsive barrier against gelation can be removed by the chemical conversion of the ionizable groups. For instance, sulfuric acid-hydrolyzed CNCs have been desulfated by heating an acid-form CNC aqueous suspension in the presence of glycerol (glycerolysis) followed by controlled water removal, yielding thixotropic CNC hydrogels that would be otherwise prevented by the charged sulfate ester groups.^634^ Different desulfation routes have been demonstrated (including solvolysis into pyridinium salts, hydrothermal treatment, mild HCl hydrolysis and NaOH hydrolysis)^635−637^ and could be used for the same purpose not only in CNCs, but also in other sulfated polysaccharides such as carrageenan and heparin.

#### 5.3.2 Gelation Induced by Metal-Coordination

A metal coordinated complex refers to a molecular structure consisting of a metal ion, called coordination center, surrounded by bound organic molecules, the ligands. Metal coordination enables precise spatial organization of molecular assemblies. Its versatility has motivated numerous research efforts related to metal organic frameworks (MOF) and metal phenolic networks (MPN), with natural phenolics such as tannins being the most explored. Although alginates form well-ordered and periodic networks with bivalent cations (especially Ca^2+^) called egg-box, they do not comprise a classical metal coordination assembly. The cross-linking of Ca^2+^ with the −COO^–^ groups present in the *G* units of alginates, or on TO–CNFs, is most widely characterized as an ion specific electrostatic interaction,^608^ and is therefore not addressed in this section (see Section 5.3.1.2).

The versatility of metal coordinated networks arises from the vast number of possible combinations between organic ligands and coordination centers.^638^ Pseudodendritic or polydentate ligands coordinated by multivalent metals are the most applied in materials development. They lead to a high coordination number, thus resulting in robust complexes and continuous networks by bridging the primary complexes across length scales. Gelation takes place when the interconnectivity between the primary complexed structures overcomes a percolation threshold that is imposed by the components of the system. Metallogels, that is, gels formed by metal coordination, aim mostly at specific functions rather than structural features for mechanical performance. However, multiple metal coordination sites, which may be considered weak interactions in isolation, can collectively create robust gels and self-standing materials upon solvent removal.

##### 5.3.2.1 Metal Phenolic Networks (MPNs)

**Figure 18:**
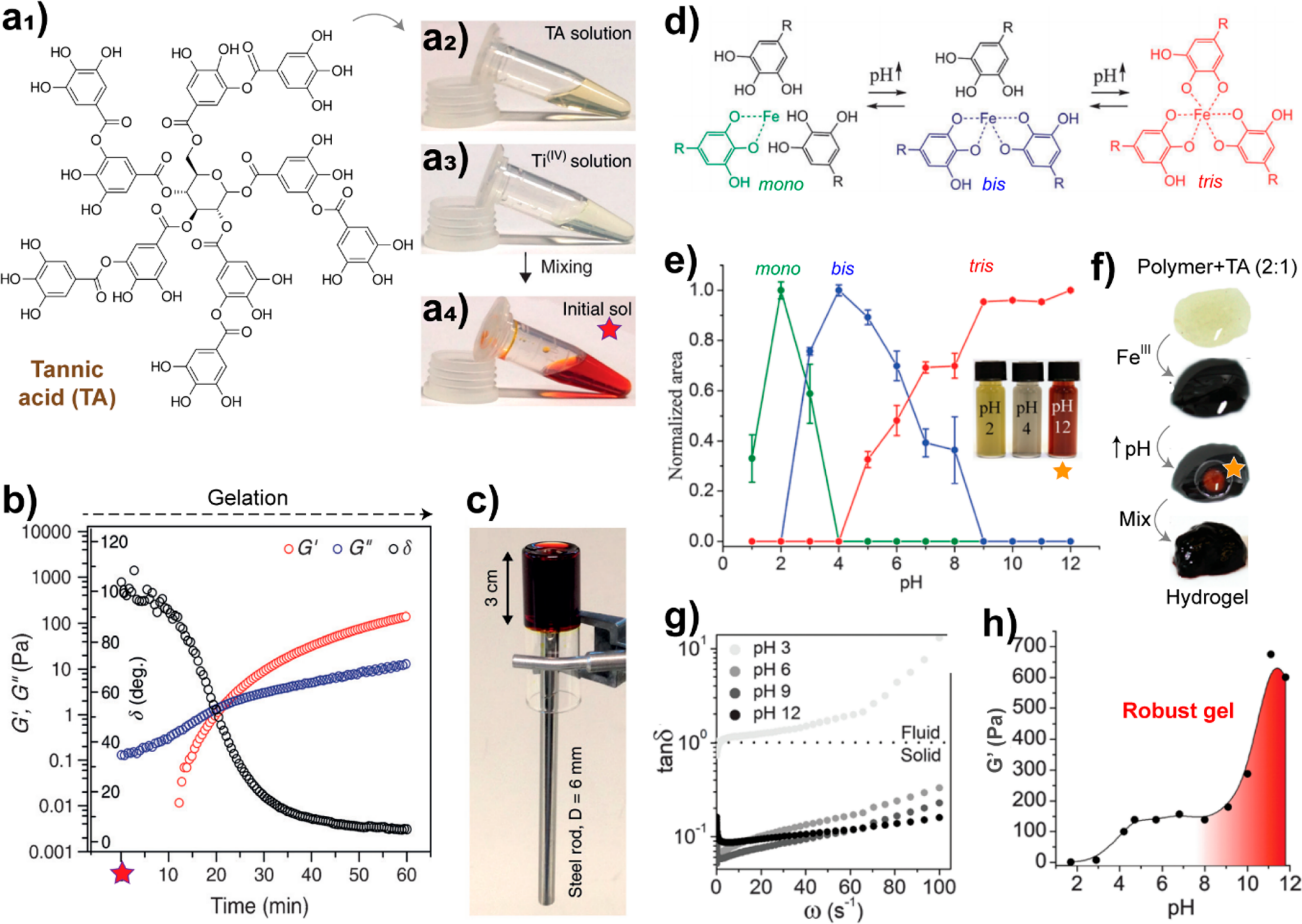
Gelation of renewable biomolecules following sol–gel transitions. (a1) Tannic acid (TA) molecule widely utilized in the formation of metallic coordination networks, which upon mixing with Ti^**4**+^ gels spontaneously under short time (a2–a4). (b) Gelation of TA-Ti^4+^ system takes place after only 15 min of contact, which is characterized by the increase in viscosity and *G*′ > *G*′′, making strong adhesive gels. (c) TA-Ti^4+^ metal phenolic gelled network displaying high adhesion and gel cohesion. (a–c) Adapted with permission from ref (641). Copyright 2016 John Wiley and Sons. d) TA can form mono, bis and tris complexes with multivalent cations, especially Fe, depending on the pH of the media (e). (f) Adjustments of pH are a central resource to induce gelation in TA containing systems, due to favoring of tris complexes at basic conditions. (g, h) Effect of the pH on the viscosity and overall rheology of the systems displaying strong gels being formed when tris complexes take place. (d–h) Adapted with permission from ref (647). Copyright 2014 The Royal Society of Chemistry.

Tannic acid (TA), a polyphenolic biomolecule derived from plants, has been widely used in the recent efforts on MPNs.^75,76,639,640^ The chemical structure of TA typically comprises ten galloyl moieties bound to a central glucose unit (Figure 18a), providing a relatively high density of hydroxyl groups in a smaller and more homogeneous molecule, when compared to lignin. TA solutions mixed with solutions of metal precursors form a “sol” phase, typically in acidic conditions (pH < 2.5), which upon pH adjustment spontaneously form metal-coordinated complexes that continue forming until a transition from “sol” to “gel” takes place. Titanium (Ti^4+^) and iron (Fe^3+^) are the most common metal ions used in the formation of metallogels as they can form *tris* complexes with the galloyl moieties of TA at neutral pH.

Multivalent titanium ions form strong gels in the presence of only TA. A spontaneous transition of a TA-Ti^4+^ (1:5 molar ratio) solution into a gelled phase (Figure 18a2–a4) takes place at room temperature in a time span of 1 to 30 min, depending on the solvent (Figure 18b,c).^641^ TA-Ti^4+^ gels formed either in organic solvents (e.g., *N*-methyl-2-pyrrolidone (NMP), DMF, and DMSO) or water, which expands their applications and possibilities for compositing with other nanomaterials using such gels as supports.^642,643^ There is an interplay between the TA-Ti^4+^ molar ratio and the total concentration of the components during the spontaneous gelation of this system: lower molar ratios (meaning high metal contents) and higher overall concentration of the components lead to faster gelation, reaching gelation times as low as 1 min. Both storage (*G′*) and loss (*G′′*) moduli of the gels increased significantly with the increase of the TA content in the systems, with the TA-Ti^4+^ molar ratio always following proportionally. At 20 wt %, *G′* of the TA-Ti^4+^ organogels approached 50 kPa, whereas at 10 wt %, it lies around 300 Pa. Such systems are notably versatile as far as the properties of the gels and their composition. However, the authors pointed out that the spontaneous formation of such gels occurs only for metals from the group IV and that the mechanism is not fully understood. Zr^4+^ also formed strong gels spontaneously in the presence of TA, following the same principles described for TA-Ti^4+^ gels.^644^ It was inferred that the oxidation states and charge of Ti^4+^ plays a role in the solvent entrapment in the gelation, which would explain why Fe^3+^ cannot form gels in the presence of TA alone despite exhibiting very strong coordination interactions with it.^641^

In TA-Fe^3+^ enabled metallogels containing a third component, typically a polymer, the metal coordination between TA and Fe^3+^ cross-links the polymeric solution and creates a gelled system.^645−647^ Few examples in the literature have demonstrated the power of such a combination in creating gels with a variety of functions such as sensing^647^ and self-healing.^646^

TA can interact with polymeric chains via hydrogen or ionic bonds while cross-linking the whole matrix by metal-coordination with Fe^3+^. Many polymers have been used in TA ternary gels, such as poly(dimethyldiallylammonium chloride) (PPDA), poly(vinylpyrrolidone) (PVP), poly(styrenesulfonic acid) (PSS), and poly(ethylene glycol) (PEG).^645^ The balance between each of the mentioned interactions can be tuned by adjusting the mass ratios between the polymer/TA and TA/Fe^3+^. This leads to a fine-tuning of the cross-linking density, and results in tailored mechanical cohesion of the hydrogels. Typically, increasing the TA/polymer ratio causes a stiffening of the hydrogels; however, upon increasing addition of Fe^3+^ the hydrogel stiffens first but ultimately weakens. The authors found a TA:Fe^3+^ 3:5 molar ratio to be the optimum conditions to balance TA cross-linking (either by hydrogen bonding or ionic interactions) with the polymer and TA-Fe^3+^ metal coordination.^645^*G′* of a PEG/TA/Fe^3+^ hydrogel increased from 2.5 to 10 GPa when the TA:Fe^3+^ ratio went from 3:3 to 3:5, but *G′* decreased to 4 GPa when the ratio was 3:8.

The pH dependency on the formation of *mono*, *bis*, or *tris* complexes between TA and Fe^3+^ (Figure 18d) has been investigated for understanding the effect of the complexation degree on the properties of the obtained gels. In that case, the third component of the TA-Fe^3+^ gels was poly(allylamine) (PAA).^647^ It was demonstrated that degree of complexation is fully tethered to the pH of the solution, where *mono* complexes with Fe ions are formed in highly acid medium (pH < 2), *bis* ones in a mildly acid range (peaking at pH 4), while *tris* complexes start forming at pH 4–5 and are maximized at pH 8 (Figure 18e). There is a remarkable effect of the complexation degree on the *G′* of the PAA-TA-Fe^3+^ gels. *G′* increases significantly from *mono* to *tris* complexation type, going from values ∼20 Pa for *mono* complexes to over 300 Pa for *tris* complexes (Figure 18g,h). Many other efforts have been harnessing the pH dependency on the TA-Fe^3+^ complexation degree in the formation of not only gels, but a whole variety of systems constructed by MPNs.^640^

Efforts on using TA toward gelled systems were further expanded by anchoring the TA molecules onto CNCs.^648^ The rod-like cellulose nanoparticles were functionalized with tannic acid by physical adsorption at high pH, which was made possible because of the highly adhesive character of the TA galloyl and catechol moieties.^649^ Subsequently, the TA-coated CNCs were incorporated into a poly(acrylic acid) solution, and cross-linked with the addition of Al^3+^ ions by supramolecular metal coordination.^648^ Interestingly, although used as a minor component (ranging from 0.1 to 1.5 wt %), the TA-CNC components played a key role in the cohesion of the gelled system. Increasing the content of TA-CNC (with a constant TA-Al^3+^ molar ratio), the toughness of the hydrogels increased from ∼0.2 MJ m^–3^ at 0.1 wt % TA-CNC concentration to over 5 MJ m^–3^ at 0.6–0.8 wt %. After 0.8 wt % TA-CNC in the hydrogel, the toughness decreases while the ultimate tensile strength increases from 300 to ∼370 kPa.

The formation of MPNs from TA, and therefore the corresponding metallogels, are affected by external factors that can block (partially or fully) the coordination interactions between the galloyl and catechol moieties with the metal ions. The effect of the ionic strength was addressed by using 0–2 M NaCl in the medium for the formation of MPN from TA and Fe^3+^.^650^ Using thin films as a model system, the authors found that at high ionic strength (2 M NaCl) the chelating groups of the TA-Fe^3+^ complexes are shielded by sodium salts, thus interacting with other coordination centers more loosely. At lower ionic strength, the metal-TA complexes are assembled tighter, thus blocking the access to the coordination metal from other interacting groups. Although not explored in the referred work, this ionic shielding could be a tool for manipulating the cohesion of hydrogels as well as their gelation.

More recently, TA has been integrated with other biomolecules, such as proteins,^93^ enzymes,^651^ and cyclodextrin,^652^ thus expanding greatly the MPN toolbox for materials development. Although sol–gel transitions driven by coordination interactions are nearly ubiquitous in such systems, they have not been addressed specifically yet. However, one can foresee developments of high-performance gelled systems built from TA and other biopolymers for biomedical applications because of their highly adhesive character to virtually any surface^76^ and its strong interactions with proteins,^94^ especially for *in vivo* gelling purposes such as injectable hydrogels. This is a timely topic given the recent prospective efforts to map the biocompatibility and immunogenicity of MPN systems.^653^

##### 5.3.2.2 Metal Organic Frameworks (MOFs)

MOFs have not been as thoroughly investigated for biopolymer gelation purposes as MPNs. However, they have been combined with biopolymers, especially nanocelluloses, to prepare a wide range of functional materials, from volatile organic compound (VOC) sensors^654^ to supercapacitors,^655^ whose functions are tethered to the chosen ligands and coordination sites. A gelling phase, even if not the center of these studies, is often present as an intermediate state prior to consolidation of the precursor solution/suspension into a dried, robust material. For instance, zeolitic imidazolate framework (ZIF) MOFs were assembled on TO–CNFs (CelloZIF8) to prepare functional 3D print inks containing drugs for drug delivery by one-pot reaction pathway at room temperature. CelloZIF8 displays higher viscosity than TO–CNFs, which is mainly attributed to the MOF structures bridging nanofibers via coordination with Zn^2+^ ions and increasing the density of their interactions. The values of *G′* and *G′′* increased both with the increase of ZIF-8 loading in the system, from values below 1 kPa to values above 100 kPa, thereby the rheological properties could be tuned for optimal printability.^656^

**Figure 19:**
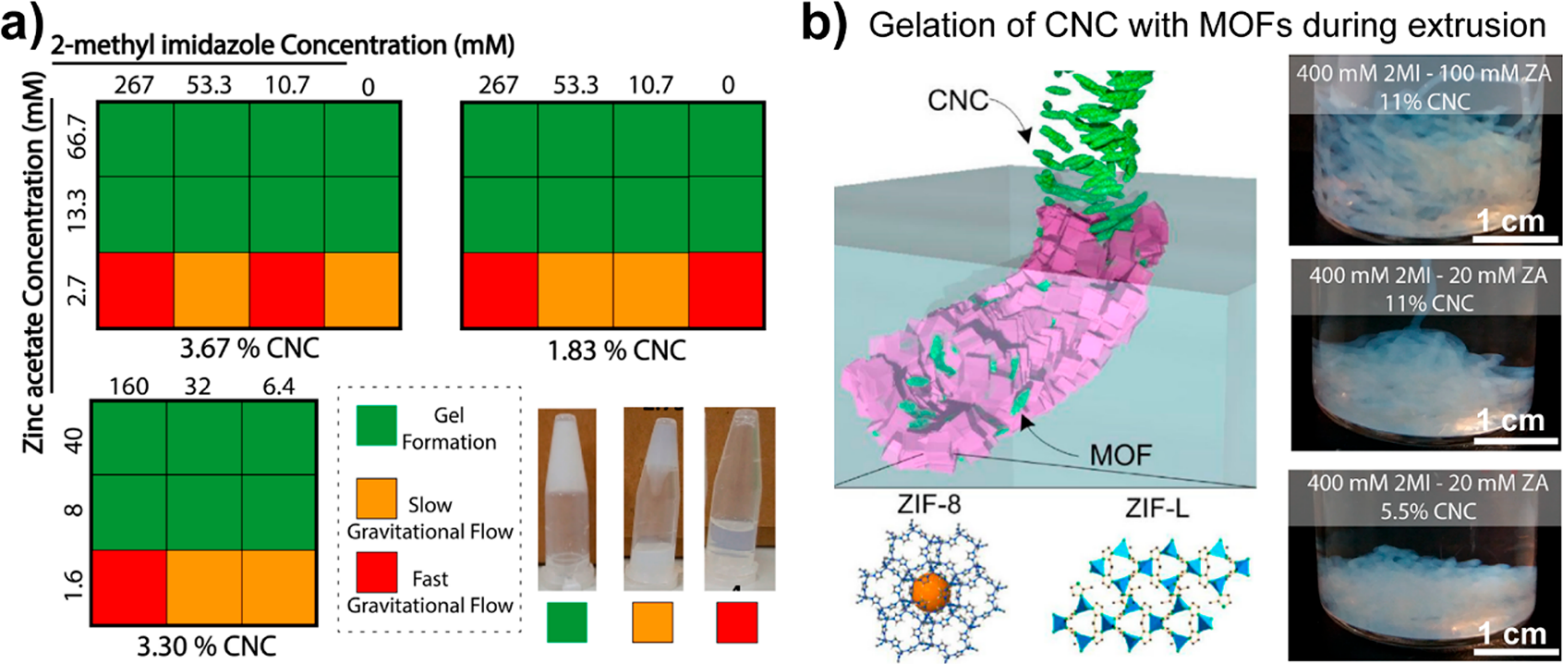
MOF-assisted gelation of CNCs. (a) Phase diagram to discriminate the sol–gel transition of the hybrid MOF (ZIF-8)/CNC system as a function of the concentration of each MOF precursor (ligand and metal) at given CNC fractions. (b) Spontaneous gelation assists the extrusion of MOF/CNC hybrid systems in a continuous manner, having the cohesion of the wet construct tethered to the conditions (i.e., concentration) of the precursors. Adapted with permission from ref (657). Copyright 2019 American Chemical Society.

CNCs have been demonstrated to enable a continuous nucleation of ZIF-8 (and ZIF-L) MOFs from the surface of CNCs, thus allowing the formation of composite hydrogels that can further be consolidated into porous assemblies that carry active ingredients, either *in situ* or post formation.^657^ In this effort, the authors investigated the formulation aspects on the gelation of the systems. Generally, gels form more spontaneously at higher Zn^2+^ concentration, regardless of the concentration of the ligand (2-methyl imidazole) or the solid support (CNCs). Remarkably, gelation is achieved at very low mass fractions (ca. 0.6 wt %, Figure 19a), if the formulation is well devised. To obtain a gelled system, cohesive enough for extrusion and resilience in liquid media, the concentration of the formulation components is slightly increased proportionally to 400 mM of the organic ligand, 20–100 mM of the metal ion and up to 11 wt % of CNC concentration in the suspension (Figure 19b).^657^

#### 5.3.3 Thermally Induced Gelation

Temperature shifts, either cooling or heating, induce gelation by physical cross-linking, driven by molecular interactions, in a wide variety of biopolymers. This takes place principally in aqueous systems, as temperature strongly affects the potential for intra- and interpolymeric hydrogen bonding. Thermal gelation is also tethered to the upper (UCST) and lower (LCST) critical solution temperature, highly relevant for biopolymeric solutions and mixtures in terms of processing, but also for thermoresponsive materials. For instance, agarose and carrageenan gels are obtained from cooling their hot solutions, while some cellulose derivatives and proteins can form gels by heating their cold solutions. Temperature shift leads to conformational changes, for example, polymer coiling/uncoiling, that allow the molecules to interact via hydrogen bonding, electrostatic, hydrophobic or vdW interactions above or below a temperature threshold.

**Figure 20:**
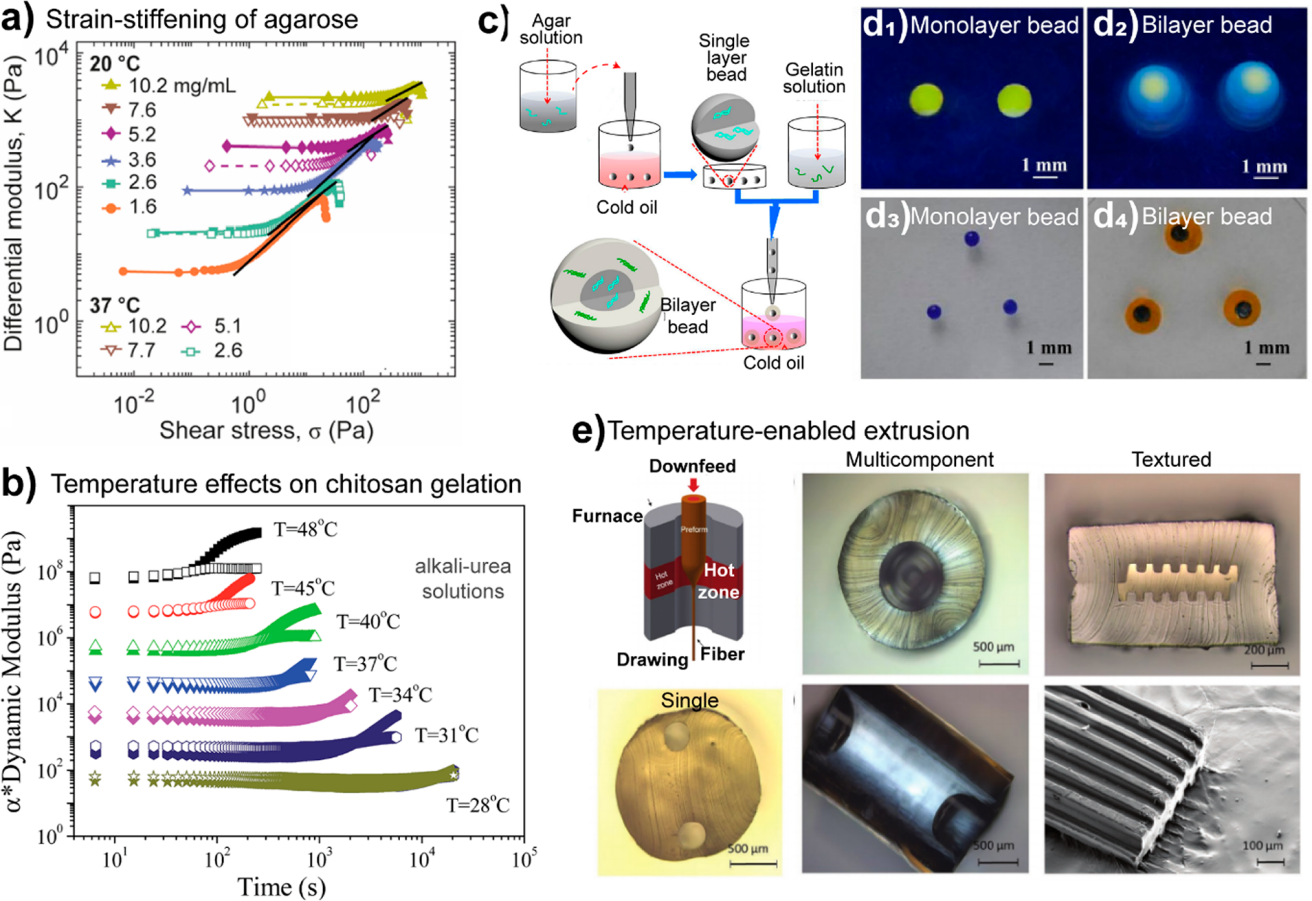
Thermal gelation of biopolymeric precursors. (a) Effect of the temperature and concentration on the strain-stiffening behavior of agarose solutions. Reprinted with permission from ref (658). Copyright 2019 American Chemical Society. (b) Effect of temperature on the viscoelastic properties of chitosan in aqueous alkali urea solutions. Reprinted with permission from ref (659). Copyright 2014 The Royal Society of Chemistry. (c) Thermal gelation of agar and gelation upon cooling have been harnessed to prepare bilayer beads that can contain particles entrapped inside the first (agar) or the second layer (gelatin) (d1–d4). Adapted with permission from ref (667). Copyright 2017 Elsevier B.V. (e) Thermal gelation and melting of gelatin have also been utilized in the preparation of multicomponent, textured filament via simple, but modulated, thermal injection processes. Adapted with permission from ref (101). Copyright 2019 John Wiley and Sons. Note: Further permissions related to the material utilized in (a) (pubs.acs.org/doi/10.1021/acsmacrolett.9b00258) should be directed to the American Chemical Society.

The architecture of the gelled network is tethered mostly to the biopolymer nature rather than the pathway taken for gelation. For instance, the hierarchical structuring of *k*-carrageenan from single coiled-coil into quaternary structures, or in the case of the triple-helix structure characteristic of gelatin gels.^608^ It is common that thermally conditioning biopolymeric gels below or above the temperature for sol–gel transition does not lead to significant changes in their viscoelastic properties. Strain-stiffening agarose gelled networks does not change their network morphology or viscoelastic properties when conditioned at 20 or 37 °C (Figure 20a). Gelation, however, was remarkably affected by the agarose concentration, with critical stress for strain-stiffening scaling at power three with respect to the agarose concentration.^658^ Agarose solutions gel due to the formation of helical fibrillar bundles upon cooling their hot aqueous solution, with strain-stiffening character coming from the semiflexible nature of the agarose fibrils as well as their geometrical connectivity being below the central-force isostatic critical connectivity.^658^ The latter corresponds to the presence of rigid meta-structures which favor a high load transfer to the gel upon shearing.

Temperature-induced gelation of biopolymers can display reversible character with an associated hysteresis on the phase transition temperature. For instance, chitosan dissolved in cold aqueous alkali-urea solution gels independently of the chitosan concentration at ∼41 °C, transitioning back to solution state at ∼5 °C. Hysteresis was observed in cooling–heating cycles, shifting both gelling from 41 to 37 °C and liquifying from 8 to 3.5 °C. Gelation time was also remarkably affected by temperature. Whereas the chitosan solution required ∼3 h to gel at 28 °C, it only needed ∼1 min to form a strong gelled phase at 48 °C (Figure 20b).^659^ Chemical modification of chitosan to display isopropyl side-chains has been shown to yield a thermoresponsive precursor capable of forming gels, driven by hydrophobic interactions, at a temperature starting from 25 to 47 °C directly from neutral aqueous solutions.^660^ Chitosan solutions gelling at ∼37 °C, and in neutral pH conditions, are especially attractive for biomedical applications given its biocompatibility. Another way to induce temperature driven sol–gel transitions in chitosan-centered systems relied on the grafting of PEG methyl ether and poly(ε-caprolactone) copolymers to the chitosan chains, which enable a tunable LCST.^661^

Most proteins are denatured at high temperatures (above 60 °C commonly, and above 85 °C for proteins from extremophiles). Proteins undergo gelation either at cooling or heating conditions, depending mostly on their molecular arrangement, for example, if globular or fibrillar,^608^ which makes them versatile building blocks for a variety of multiphase systems. The effect of a partial replacement of cod proteins (CD) by soy protein isolates (SPI) (β-conglycinin and glycinin) on the sol–gel transition and rheological behavior of the given gels was investigated. Gelation took place by cooling (to 4 °C) a hot protein solution (at 100 °C). Within a fixed CD:SPI ratio of 1:3, the increase of the glycinin fraction in the SPI total content led to higher surface hydrophobicity, which resulted in the formation of larger protein aggregates. During heating, the proteins unfold thus exposing hydrophobic and sulfhydryl groups that interact strongly to form aggregates and consequently a strong gel. Glycinin content also modified remarkably the *G′* of the CD/SPI protein mixture, from 800 to 1400 Pa when 25% of the SPI contribution came from the glycinin fraction. Surprisingly, preheating of protein solution had only a mild effect on the final viscosity of the systems.^662^

Temperature can act synergistically with other gelling factors, for example, salts, to induce or to manipulate the gelation of biopolymer solutions. Structuring of carrageenan can be strongly influenced depending on its sulfonated degree (κ- or ι-) and the interacting ion. Upon cooling a hot solution (at 90 °C), κ-carrageenan (one sulfate group per disaccharide) self-assemble in the presence of potassium salts from random coil to single helix, followed by intrachain supercoiling and macroscopic anisotropic domains that form a quaternary network. Potassium cations had a greater effect in the early structuring (from primary to secondary structures) of the κ-carrageenan than sodium cations, indicating an enhanced binding affinity of K^+^ to κ-carrageenan. Loosely intertwined single helices were found in ι-carrageenan in the presence of either Na^+^ or K^+^ ions, which provided an elastic mesh network with several interactive junctions. By understanding the self-assembly of biopolymers, similarly to what is known for proteins, one can prepare gels with tunable elasticity, of up to ∼1000 Pa.^663^

Nowadays, the larger body of research on temperature-centered biopolymer gelation lies, however, more frequently on the preparation of functional and structural materials rather than on the understanding of their gelation mechanisms. Functional edible fibers,^101^ injectable hydrogels,^661^ flexible films,^664^ capsules for cargo deliver^665^ and condition-responsive gels^666^ are few examples of the utilization of thermal centered sol–gel transitions toward the development of structured materials or systems.

Controlled gelation of agar-gelatin mixtures, both gelling upon cooling their hot-conditioned solutions, has led to the low-energy formation of double layered capsules that can carry a variety of cargoes (Figure 20c).^667^ Such mixture is especially attractive as multipurpose carriers as it contains relatively different environments across the layers of the material—one polysaccharidic and another proteinaceous—to which biodegradation and bioabsorption can take place selectively depending on the surrounding aqueous environment. Placing different cargoes within a specific carrier environment opens new possible applications, for instance, for gastrointestinal delivery of active molecules. Such capsules are formed in a two-step procedure. First an agar solution (5 wt %) at 95 °C was added dropwise into a soybean oil reservoir at 4 °C (cold oil), where it promptly gelled. Then the agar beads were mixed with a gelatin solution (10 wt %) at 40 °C, which was not enough for reversing the agar from gel to solution. The latter mixture was again added to a cold soybean oil bath to form the bilayer beads. Particles or molecules that can be suspended or dissolved in such conditions can be entrapped in the gelatin or agar gels, as it was demonstrated by loading rhodamine B (fluorescent at 365 nm light) (Figure 20d1–d3), and a combination of rhodamine B in the inner agar layer, and carbon dots in the gelatin outer shell (Figure 20d2–d4).^667^

Capsules with tunable mechanical performance, from soft to hard, could be fabricated by using κ-carrageenan and locust bean gum (LBG) gels.^665^ Therein LBG was added to the κ-carrageenan matrix to overcome its brittleness and instabilities upon storage. Strong gels were formed by heating LBG/κ-carrageenan dilute solutions (1.5 wt %) containing potassium citrate (0.2 wt %) at 90 °C for 1 h. The solution was then cooled to obtain the gels. An important observation is that the presence of LBG did not interfere with the formation of double helical 3D continuous network of carrageenan, displaying a sol–gel transition at roughly the same temperature (ca. 40 °C) regardless the LBG content. Such system, and its varied formulations, could be used to prepare hard capsules and flexible films, mostly based on the LBG content. For instance, a 1:2 LBG:κ-carrageenan ratio resulted in brittle materials under ∼2 kgf, whereas a 3:1 ratio led to softer materials with brittleness value below 1 kgf. The presence of K^+^ ions, which interact ionically with the carrageenan molecules and cross-links them, remarkably affected the LBG: κ-carrageenan hardness and water retention capacity. Hardness increased constantly up to 0.2 wt % of K^+^, and then plateaued due to the saturation of the cross-linking sites. Water retention capacity decreased by 10% at such K^+^ concentration.^665^ Additionally to LBG, other nonstructural biopolymers (i.e., saps), such as guar and tara gums, have been used to similar purposes.^664^ Gums and soy proteins have also been combined to increase the sol–gel transition by up to 16 °C, which could help to preserve nonthermal stable components coexisting in the system.^668^

Gelatin is a remarkable precursor for development of materials as it structurally changes into a triple helix gelled state from a random coil configuration upon cooling its hot aqueous solutions. Thermal drawing of gelled gelatin precursors can be realized due to the reversibility of the triple helix into a more mobile disorganized structure. It was shown that by controlling the gelatin to water ratio, and by adding plasticizers (e.g., glycerol), very soft or brittle materials can be obtained, with water content having an exponential relationship with the critical complex viscosity of the gelatin-glycerol precursors.^101^ Gelatin-glycerol 1:1 (mass fraction) displayed phase transition at 55 °C, and it could gradually increase up to 65 °C with the reduction of the gelatin-glycerol ratio to 1:0.5. Using such systems, edible filaments in a variety of morphologies and compositions were drawn (Figure 20e), finding applications as functional foods.^101^ Remarkably, the cross-sectional geometry of the complex precursor dope could be used to predict the multimaterial composition and location of the drawn filaments.

Microgelation of agar and Curdlan, to thereafter be applied in Pickering-emulsions, could be achieved by either bottom-up or top-down approaches. For a bottom-up strategy, agar was dispersed at dilute conditions (ca. 1 wt %) in water under stirring (550 rpm) and heated up to 95 °C, kept for a short time, and let to cool down to 25 °C, still under stirring. A similar pathway was taken for Curdlan, with the addition of a step involving a high-speed blender prior to the heating step. For the top-down method, macrogels were first prepared following the procedures described above in the absence of constant stirring, broken into small pieces, diluted, and then subjected to homogenization by using a high-speed blender. Depending on the processing conditions, such as homogenization severity, the size of the microgels could be controlled from 5 to 15 μm. In their microgelled, colloidal form, both agar and Curdlan could stabilize Pickering emulsions containing soybean oil, with controllable creaming properties dependent on the processing conditions. The mild top-down approach led to higher creaming index (20%) for agar, whereas the bottom-up approach led to 40% of creaming index in the case of Curdlan.^669^

In conclusion, with these studies, which are supported by decades of research, it is demonstrated that several physical phenomena can be exploited to manipulate the gelation properties of biopolymers. These gels can be suitable precursors to introduce properties in their consolidated materials that are otherwise absent, as well as to enhance specific features, both leading to the formation of high-performance sustainable materials.

## 6 Consolidation Phenomena in Material Development

Consolidation, where bulk materials are obtained from gelling or gelled biopolymeric precursors, are affected both by the architecture of the precursors, solid content, the building block dimensions, surface chemistry, and more importantly, by the process and the parameters used to form the final material. In this section, we first discuss the process parameters as exemplified by the most common assembly processes reported in the literature. We put forward the range of materials that can be obtained and critically discuss their architectures, mechanical properties and functionalities. Thereafter, the effect of the biopolymeric building block’s surface chemistry and dimensions, as related to their extraction process are critically evaluated. We also compare the efficiency of the processes used for consolidations to form strong and tough materials. Overall, this section presents the necessary information for choosing a suitable process used as a starting point to design novel materials from biopolymers and new designs for future processes.

**Figure 21:**
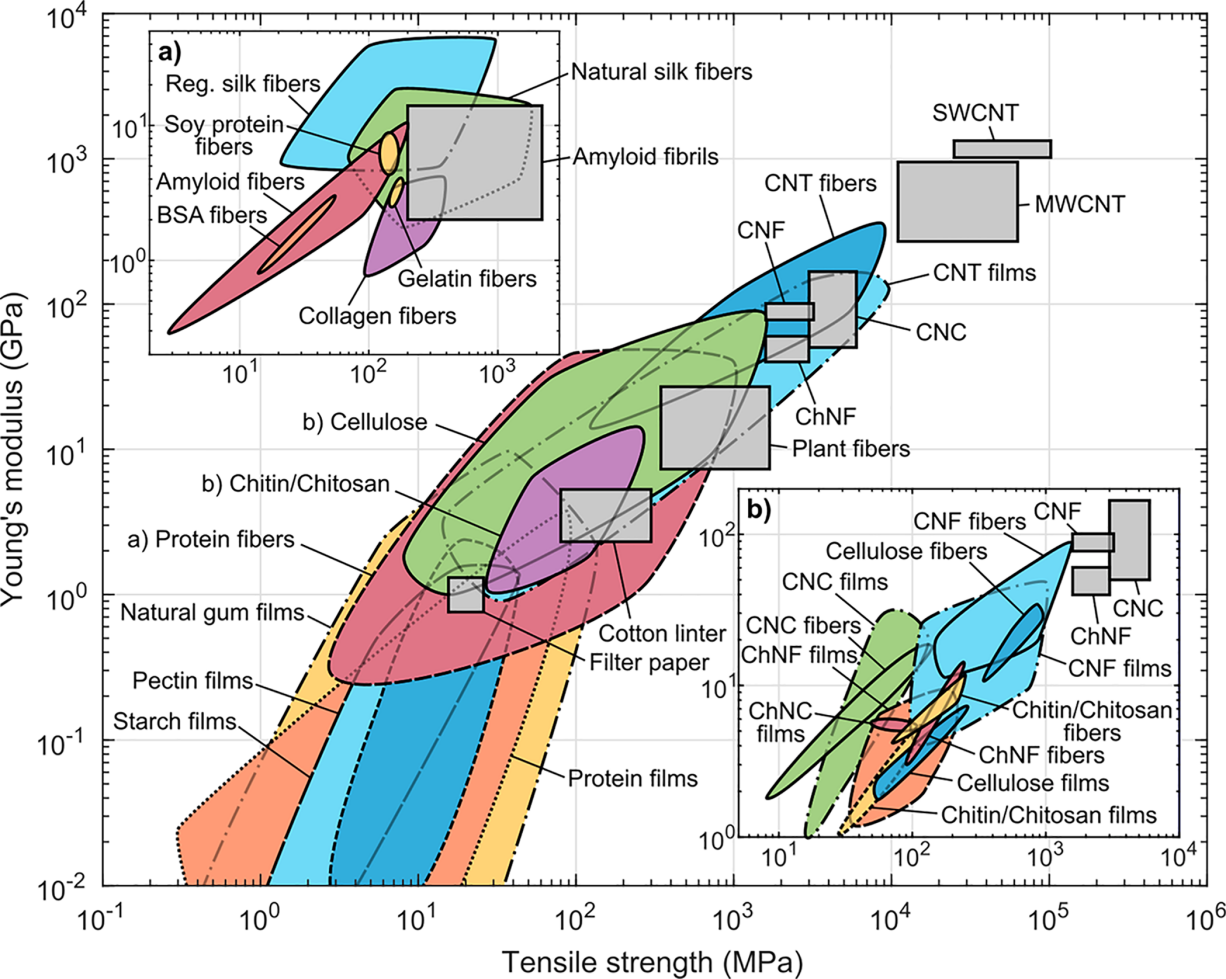
Ashby plot showing literature values of Young’s modulus as a function of ultimate tensile strength with emphasis on the progress during 2010–2020. The insets detail the contents of protein fibers (top left) and nanocellulose/nanochitin fibers/films (bottom right). See Supporting Information for details and references used in the development of this figure.

We delineate five assembly processes, namely spinning, casting, regeneration, self-assembly, and compositing. Compositing and self-assembly can result in materials by any combinations of spinning, casting or regeneration, while regeneration is most typically performed in a spinneret. However, as can be seen in Figure 21, the process has a significant impact in the strength of the materials formed. Across those various processes, the impact of size of the building block and that of the surface charge of biocolloids are finally discussed and put in perspective.

Process-wise, filament or fiber formation leads to significantly higher strength and stiffness compared to film formation as a result of long-range order formation during extrusion as well as tension induced during consolidation. This is the case of protein (silk), cellulose and carbon nanotube (CNT) fibers, where improvements in mechanical properties of about three, one and half an order of magnitude are observed, respectively, compared to films. Interestingly, when comparing the properties of the building blocks to that of their constructs, cellulose is considerably closer than carbon nanotube materials. Although for silk, chitin, cellulose, and CNT-based fibers, an improvement in mechanical properties is in line with the strength of the corresponding building block. For CNT, one and a half order of magnitude is observed between the best nanocarbon constructs and that of the individual building blocks while for nanocellulose the best performers have ∼7-fold weaker tensile strengths compared to the theoretical maximum of the building block. Interestingly, chitin constructs are still far from the building blocks’ properties, although this may be attributed to the infancy of the field. As we further our understanding in the relation between constructs and building blocks, as associated with defects, and more importantly, optimization of interactions, one can expect understanding of the best possible constructs to be better recognized. Many of these aspects are explained in more detail in the following subsections, where Figure 21 can be used as a reference point.

### 6.1 Gel-to-Solid: Processing Toolbox

As described previously, sol–gel transitions enable a variety of biopolymeric hydrogel architectures. Hydrogels are generally precursors to bulk biopolymeric materials, with tunable porosity, and therefore density. More importantly, the gel to solids approach significantly affects the multiscaled architecture of the final, consolidated, bulk biopolymeric material. In the final processing steps, additional interactions can develop as a function of the physical forces that are in play, as described in Section 4. These last steps are of the greatest impact on the materials properties. Therefore, it is important to consider the theoretical framework developed thus far to engineer biopolymeric materials and to facilitate predictable outcomes as a function of the scale of the involved forces and the dynamics of consolidation. Since the networks formed during the process consolidation are not easily accessible, the material properties and their structures in the final stage are used as “black-boxes” to trace back the dynamics of interactions. This section summarizes associated findings, both in terms of end-properties that are process-dependent as well as how such properties can be leveraged in the most appropriate use or application.

#### 6.1.1 Spinning and Extrusion

We discuss the consolidation of solutions and suspensions into solid materials through extrusion processes assisted by confined flow through spinnerets or dies. These assembly techniques are addressed separately as they feature unique characteristics. We highlight the large hydrodynamic stress in shear and extensional mode as well as their effects on orientation. Spinning- or extrusion-based methods include die extrusion, micro/nanofluidics, additive manufacturing (see Section 7.1.7 for further details) and electrospinning, among others and can be potentially coupled to an additional elongational step to improve alignment. This section addresses the methods used to produce morphologies ranging from elongated filaments to isotropic particles.

##### 6.1.1.1 Single Filaments and Low-Complexity Geometries

**Figure 22:**
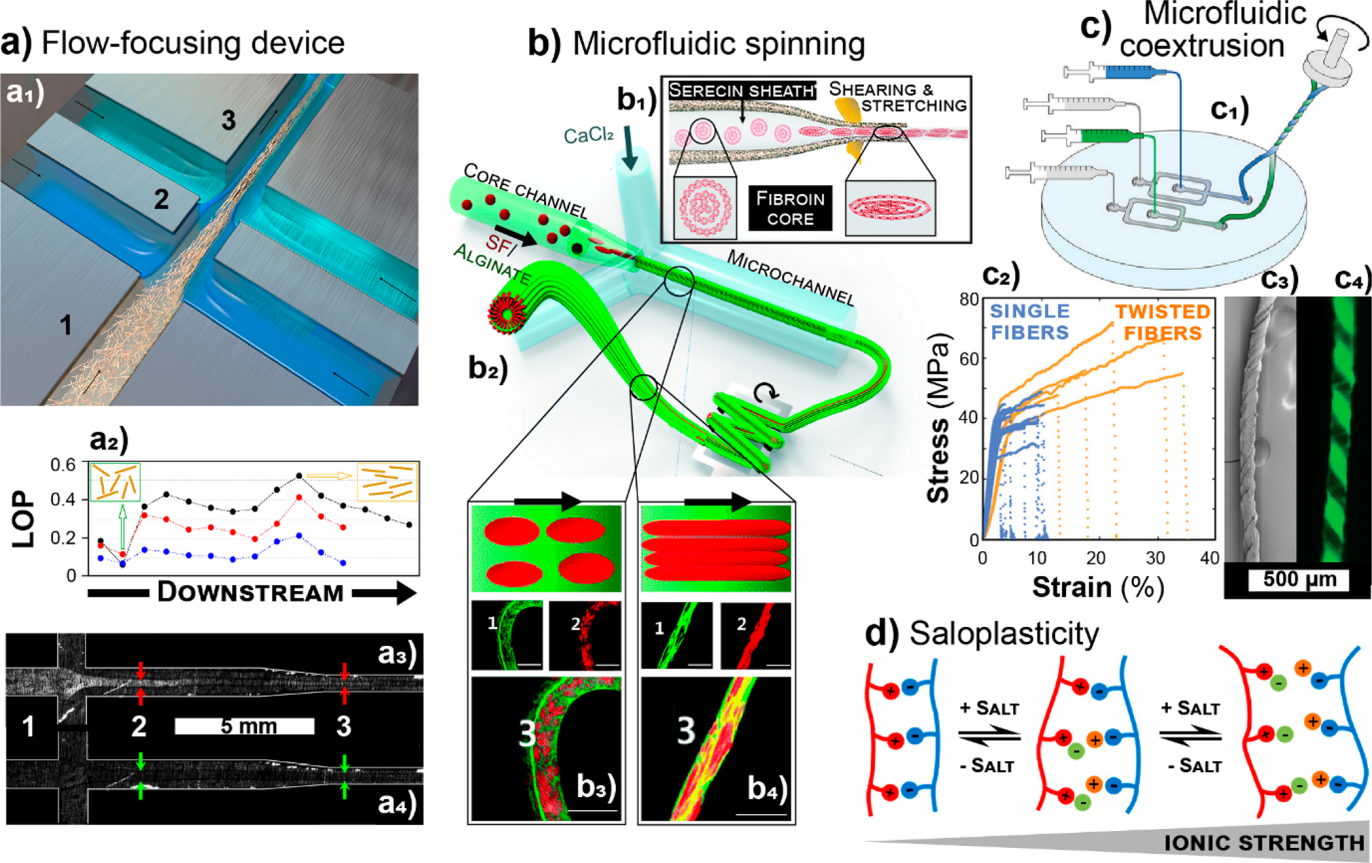
Confined flow-assisted assembly of biopolymers and biocolloids. (a) Double flow-focusing apparatus to assemble microfibers from cellulose and straight or curved nanofibrils of whey protein isolate (WPI). Suspensions/solutions are injected in a core flow (a_1_, position 1), followed by sequential injections of sheath flows of water and acid (cellulose) or buffer (WPI), driving the local order parameter, LOP (a_2_; 0: isotropic; 1: perfect alignment), from poorly aligned to different extents of nanofibril orientation (black: cellulose; red: straight WPI. Note the birefringence between cross polarizers in a_3_; blue: curved WPI, see a_4_). Adapted with permission from refs (480 and 670). Copyright 2017 National Academy of Sciences and Copyright 2018 American Chemical Society. (b) Scheme of the spinning of silk fibers by *Bombyx mori* (b_1_), where micelle-like fibroin globuli are elongated and oriented by shearing and stretching at the silkworm duct and gland. The resulting fibroin-rich core/sericin-rich sheath fiber is mimicked via microfluidics (b_2_) by ionically cross-linking an alginate sheath and machine-direction aligning the fibroin domains within the dope in a postextrusion elongational step. CLSM images show alginate (green) and fibroin (red) domains before (b_3_) and after (b_4_) stretching (scale bars: 100 μm). The black arrows in d_3_ and d_4_ indicate the fiber axis. Adapted with permission from ref (671). Copyright 2016 The Royal Society of Chemistry. (c) Dual microfluidic spinning (c_1_) of amyloid-like β-lactoglobulin nanofibril/alginate solutions (green and blue, the latter comprising thioflavin T (ThT) as a fluorophore), which are coextruded, ionically cross-linked by sheath PEG/CaCl_2_ flows, and twisted into yarn-like composite microfibers, *vide* SEM (c_3_) and CLSM (c_4_, only one extrudate dope was dyed) images. Twisted fibers perform better mechanically than single fibers (c_2_). Adapted with permission from ref (672). Copyright 2019 John Wiley and Sons. (d) Reversible evolution from intrinsic (left) to extrinsic (right) charge compensation among oppositely charged polyelectrolytes upon the introduction of salt ions (saloplasticity). Adapted with permission from ref (606). Copyright 2016 American Chemical Society.

Biopolymer solutions and biocolloid suspensions are often shaped into bulky and simple geometries at the macroscale with well-defined nanoscale structures. The preferential orientation improves the axial resistance to mechanical stress. This relationship is almost always true. Straight/long and curved/short (ca. 50 times lower persistence length than the former) amyloid-like nanofibrils were produced using, respectively, low (<4%) and high (>6%) concentrations of whey protein isolate (WPI).^670^ A core flow was connected to a double flow-focusing setup (Figure 22a_1_) wherein a WPI nanofibril dispersion was enveloped first by a sheath flow of water for fibril alignment and then by a gelling agent to quench the aligned architecture at the isoelectric pH of β-lactoglobulin. The local order parameters (i.e., degree of orientation along the flow direction) increased at the flow constrictions, followed by some Brownian motion and relaxation that favored isotropy (Figure 22a_2_). While straight fibrils (Figure 22a_3_) were more aligned than those in the curved counterparts (Figure 22a_4_), the entangled network of the latter was essential for the cohesiveness and mechanical properties of the resulting materials, that is, the strongest protein filaments benefited from the interplay between interchain alignment and entanglement.

Most cellulose composites are 3–15-times weaker than CNF,^480^ meaning that not only the biological role but also the engineered assembly has an important role in translating the mechanical properties from the nanoscale building blocks to the macroscale materials. Recently, a flow-focusing apparatus (Figure 22a_1_) was used to assemble microfibers benefiting from the nanoscale mechanics of aligned CNFs followed by quenching into a metastable colloidal glass. Originally, TO–CNFs were randomly oriented in suspension with a high freedom of rotation under Brownian diffusion and electrostatic repulsion. An increased order parameter was measured (Figure 22a_2_), and the alignment, induced by hydrodynamics was retained to a higher extent due to acidification and protonation of the surface carboxyl groups, thus strengthening attractive vdW forces and reducing Brownian motion. In this case, alignment was remarkably beneficial to transfer the mechanical stress from the macroscale fibers to the nanoscale building blocks.

Screw extrusion is a classical processing method for polymers in the molten state into filaments or other geometries, for example, sheets, pipes and pellets. More complex geometries may be achieved similarly, but through injection molding instead of die extrusion. Nevertheless, melting/softening semicrystalline/amorphous natural polymers is not always feasible as they might undergo thermal degradation at temperatures lower than those required to suppress the any intermolecular interactions in the network, which in turn prevents macromolecular flow. Starch is a natural polymer commonly used in melt-processing, even if its native granules are infusible, that is, pyrolysis takes place prior to melting.^673^ Extrusion of thermoplastic starch is enabled by weakening the interchain hydrogen bonds when plasticized with low-MW, yet nonvolatile molecules (e.g., glycerol and sorbitol). This plasticization approach also applies to other biopolymers, such as chitosan.^674^ In fact, biopolymers have been melt-processed after chemical modification, via esterification of the cellulose backbone that impairs the intermolecular hydrogen bonding and provides chains with enough mobility for processing as a thermoplastic, even in the absence of an added plasticizer.^675^

Biopolymers and biocolloids can be extruded as high-consistency solutions or suspensions. CNFs, for instance, have been slot-die extruded at a high solid content from an aqueous slurry into wet sheets that were then hot-pressed and dried.^676^ Sheets with ultimate tensile strength of ∼110 MPa and Young’s modulus above 9 GPa were produced in a continuous fashion (>1 kg/h dry mass). The introduction of processing aids was necessary, since pure CNF underwent severe dewatering, which in turn increased the solid content and led to die clogging. Among 15 water-soluble polymers that were investigated, CMC stood out as the most suitable agent to retain water. Schlenoff et al., introduced the concept of compacted polyelectrolyte complexes, saloplastics, in an analogy to thermoplastics, where interchain flow is enabled by salt loading, like heat does in the case of thermal processing.^677^ By introducing low-MW electrolytes (e.g., NaCl), electrical charge compensation was demonstrated to gradually shift from fully intrinsic, wherein all charged groups in the polycation and polyanion are paired, preventing flow, to fully extrinsic, in which the polyelectrolyte chains are isolated and the charges are compensated by salt ions (Figure 22d).^606^ In the process of increasing doping level, polyelectrolyte complexes undergo a glassy-to-rubbery-to-soluble transition that allows die extrusion.^678,679^ Saloplasticity has been mostly demonstrated using synthetic polyelectrolytes, but was extended to chitosan/sodium alginate complexes,^680^ therefore denoting a feasible strategy to enable die extrusion of other biopolymers bearing ionic or ionizable groups.

A special variation of the static die approach is when channels that confine the flow are in the nano/microscale. Nano/microfluidics allows precise control over multiphasic flow dynamics and fabrication of constructs with a range of morphological features, from monodisperse particles to elongated fibers, without the need for high energy input. Interestingly, different biomimicry microfluidic-related approaches have been proposed for mirroring silkworms, which extrude protein dopes through a narrow duct and spinneret, which in turn impose shear and elongational forces into stretched and oriented fibers that are then enriched with core-fibroin/sheath-sericin. These biomimicry efforts, pioneered by Kinahan et al.,^681^ have been growing. Notably, photolithographed polydimethylsiloxane (PDMS) microfluidic channels were designed to mimic silkworm glands and spinning ducts by extruding fibroin solutions that were then concentrated by microdialysis through a regenerated cellulose membrane, which allows water scavenging from the donor dope to a solution of hygroscopic PEG acting as water acceptor.^682^ A slightly different approach used a core flow of aqueous alginate solution and a sheath flow of a low-polarity solvent, for example, isopropyl alcohol. The sheath was responsible for dehydrating the core solution and inducing self-aggregation among the polar biopolymer chains via dipole–dipole attractions. Kelvin-Helmholtz instabilities at the dehydrating interface between the miscible solvents and generating fibers were used. Thereafter, in such processes, fibers with diameters within the nano and microscales, were tailored by the relative flow rate in the core–sheath fluids.^683^ Then silk-like core/sheath morphology was achieved through the flow-focus elongation of micelle-like fibroin globules sheathed by an ionically cross-linked alginate layer, further oriented by postextrusion using mechanical stretching (Figure 22b). Finally, a similar microfluidic apparatus was recently used, and related concepts were instrumental, to fabricate meter-long microfibers of amyloid-like β-lactoglobulin nanofibrils and alginate.^672^ The microfibers were cross-linked with divalent ions forming a coflown PEG/CaCl_2_ solution, followed by a postextrusion step intended to twist neighboring fibers into yarn-like composites (Figure 22c). The sheath flow rate itself allowed controlling both fiber diameter and mechanical performance, with increasingly faster thinning of fibers and increasing both tensile strength and Young’s modulus by inducing nanofibril orientation. Postprocessing by twisting into yarn-like fibers perpendicularly to the fiber axis also improved toughness (Figure 22c_2_).

**Figure 23:**
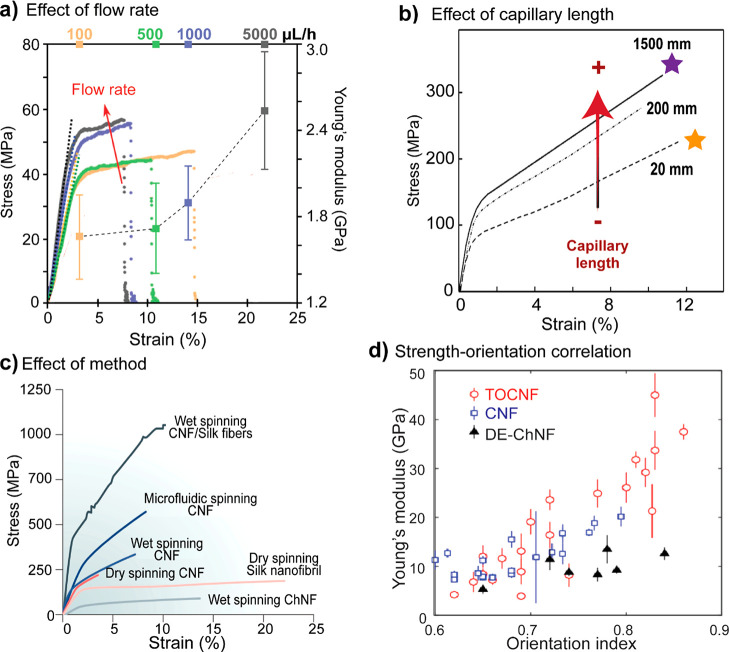
Processes and associated factors affecting the mechanical properties of spun materials. (a) Typical stress–strain curves (dots) of microfibers resulting from the shear-induced assembly of protein nanofibrils at different flow rates in a microfluidic spinning device, resulting in different orientation indices and Young’s moduli (squares). Adapted with permission from ref (672). Copyright 2019 John Wiley and Sons. (b) Representative stress–strain curves of CNF fibers spun through different extrusion capillary lengths. Adapted with permission from ref (481). Copyright 2020 The Authors. (c) Typical stress–strain curves of fibers spun from ChNF, CNF, and silk nanofibril suspensions by wet, dry, and microfluidic spinning. Adapted with permission from ref (113). Copyright 2018 The Authors. (d) Correlation between orientation index and Youngs’ modulus of filaments produced from nanocellulose and nanochitins. Plot in (d) was created using data from refs (481 and 684−686) for CNF, data from refs (480, 685, and 687−690) for TO–CNF, and data from refs (688 and 691) for DE–ChNF.

Processing factors affect remarkably the morphology and consequentially the mechanical performance of the as-spun fibers/filaments. The effect of the flow rate during spinning, capillary length (which is associated with the intensity of applied shear) and the process for spinning are summarized in Figure 23a–c. Aligning the biocolloids during spinning is most relevant as far as the mechanical performance of the materials (Figure 23d).

##### 6.1.1.2 Particles and Shape: Complex Geometries

**Figure 24:**
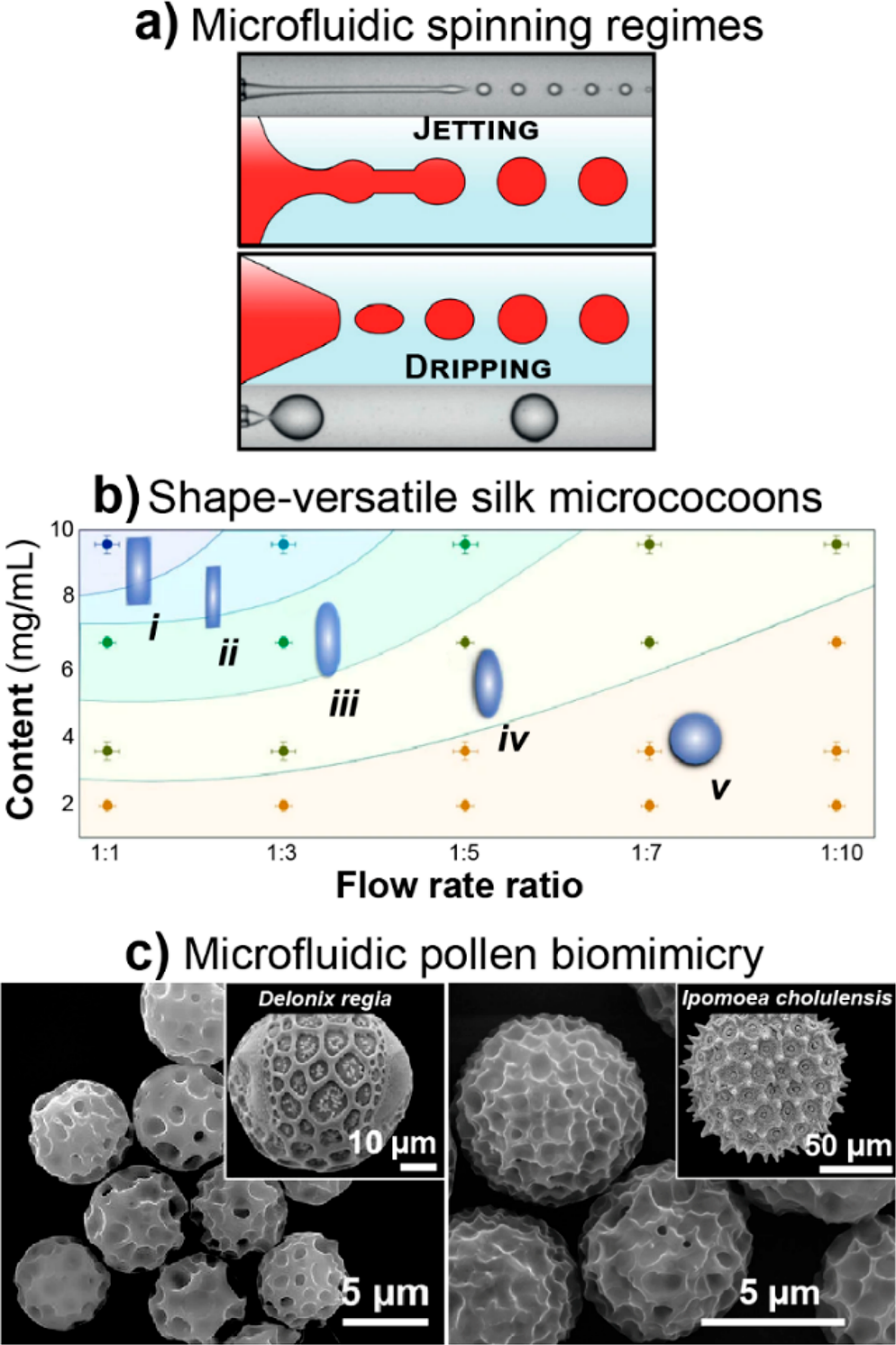
Confined flow-assisted assembly of biopolymer beads and particles. (a) Schematics (color) and optical microscopy images (grayscale) of microfluidic spinning in the jetting and dripping regimes. Adapted with permission from refs (692 and 697). Copyright 2007 The American Physical Society and Copyright 2019 John Wiley and Sons. (b) Relative flow rate (aqueous:oil phases) and fibroin content-dependent morphology of silk micrococoons: continuous, thick fiber (6–65 μm in diameter) (*i*); continuous, thin fiber (0.5–2.5 μm in diameter) (*ii*); short fiber (4–65 μm in length, 5–25 μm in diameter) (*iii*); 5–35-μm-wide, 4.5–65-μm-long cylinder (*iv*); and 6–80-μm-diameter sphere (*v*). Adapted with permission from ref (693). Copyright 2017 The Authors. (c) Representative SEM images of two of the several controllable patterns of liquid crystal elastomer microparticles inspired by natural pollen grains (insets) and produced by microfluidics. Adapted with permission from ref (696). Copyright 2020 National Academy of Sciences.

Besides elongated fibers, nano/microfluidics has been used in the fabrication of lower aspect ratio particles. Therein, fiber-to-particulate transition is achieved by adjusting the flow conditions and channel geometry, so the hydrodynamic regime is shifted from the jetting to the dripping modes, respectively (Figure 24a). The Rayleigh–Plateau instability gives rise to droplets even in the jetting mode. By adjusting the complex balance among inertial, viscous (e.g., mechanical shearing and drag), and interfacial (e.g., surface energy) forces, one can periodically breakup the flow of a dispersed phase within a continuous medium, leading to droplets that may be further consolidated into shape-isotropic particles.^692^ Such a transition was elegantly demonstrated by tailoring the fibroin concentration in a given dope as well as its microfluidic spinning rate ratio relative to an oil phase, fabricating continuous thick and thin fibers, short fibers as well as cylindrical and spherical micrococoons.^693^ At low solid contents and, therefore viscosities, surface energy dominates and favors spherical domains toward minimal interfacial area. On the contrary, shear forces govern the assembly of high-aspect ratio domains (Figure 24b). The diameter of uniform fibroin spheres were further controlled within the micron and submicron scales by varying the composition of the continuous phase, the fibroin concentration in the dispersed phase, the relative flow rates, and the MW of the protein.^694^ An even smaller size was reached when fabricating Ca^2+^-cross-linked alginate nanogels with tunable diameters (68–138 nm) and pore sizes (11–24 nm) via hydrodynamic flow-focusing fluidics.^695^ Likewise, numerous biopolymer-derived particles of varying dimensions and properties were fabricated via similar nano/microfluidic strategies, including agarose, albumin, alginate, cellulose, chitosan, collagen, gelatin, hyaluronic acid, pectin, silk and zein, as recently summarized elsewhere.^692^ It is worth mentioning that, beyond the aspect ratio, microfluidic systems are versatile and allow the fabrication of surface-patterned particles. This has been outstandingly demonstrated in a glass flow-focusing capillary device by the assembly of a range of pollen-inspired patterned liquid crystal elastomeric microparticles (Figure 24c).^696^

In addition to single filaments and particles, solids of several other geometries can be produced through spinning and extrusion. Briefly, the static die approach is limited to materials of low shape complexity, typically elongated fibers with a cross-sectional geometry resembling the die itself. An extension of this approach, herein called dynamic die, that is, when the die is movable, has been extensively exploited to fabricate biopolymer-based complex geometries, the most successful techniques being three-dimensional (3D) printing. The literature on 3D-printed biopolymer constructs is extensive and has been summarized recently and is further discussed in 7.1.7).^10,698,699^ Notably, 4D printing relates to properties, morphologies or composition of the printed object that change over time, often triggered by a suitable external stimulus, which opens up a wide spectrum of possibilities.^700,701^ Finally, biopolymer fibrous mats are typically produced via high-throughput spinning techniques, which all require that the biopolymers are processed in the liquid state. This may be achieved by heat, pressure, dissolution or combinations thereof. The most widespread techniques is electrospinning, but other variations are numerous and have been comprehensively reviewed recently in the context of biopolymers.^702^

#### 6.1.2 Solution and Suspension Casting on Substrates

Here we primarily address aspects related to casting biopolymeric solutions or suspensions on flat, nonconfined substrates, by pouring and spin or dip coating. In addition to the materials fabrication perspective, the simplicity of a casting process, its low-energy demand, reproducibility and versatility is a powerful route to investigate factors affecting the assembly of primary biopolymeric elements into macroscale constructs.

**Figure 25:**
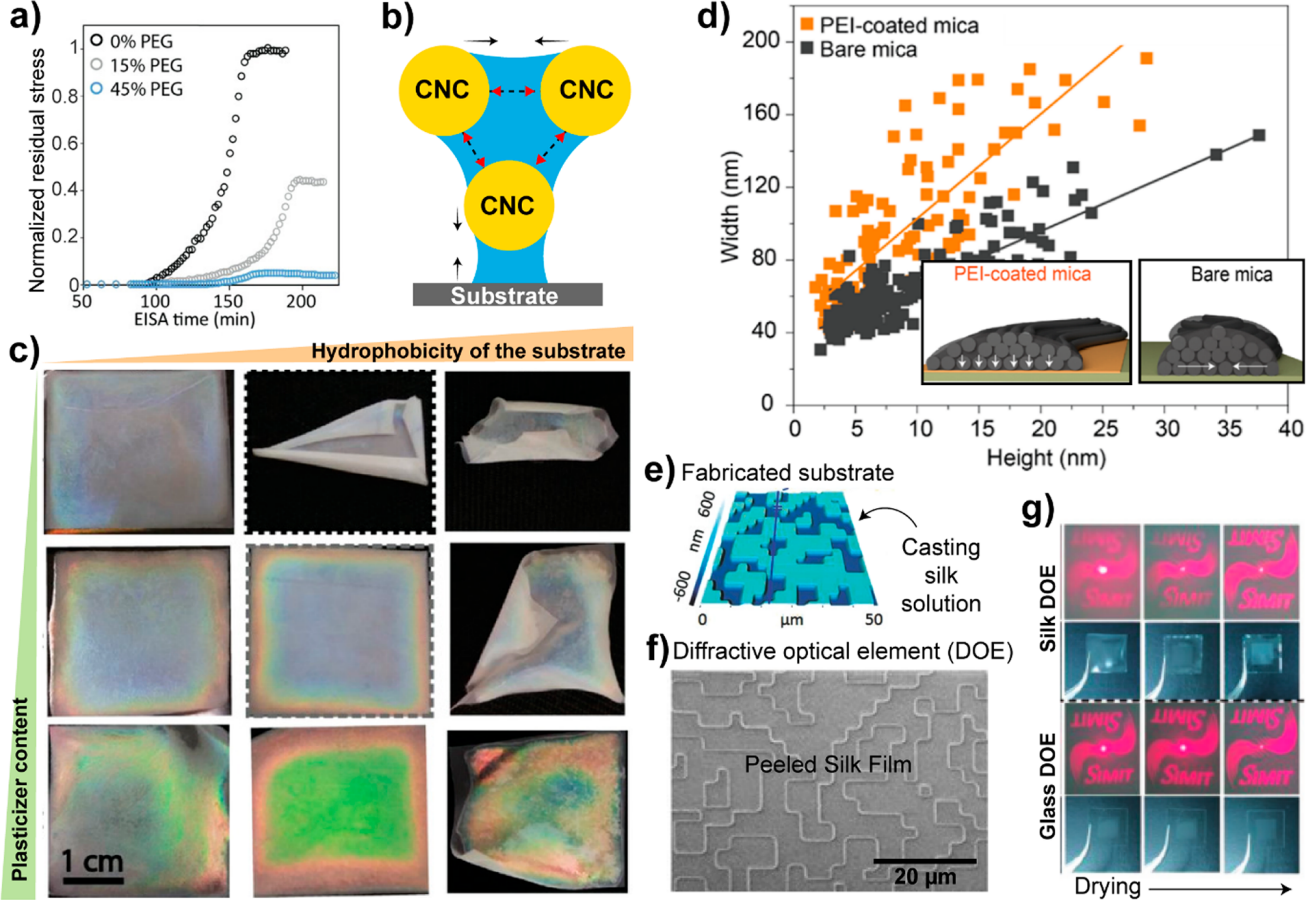
Substrate interactions within the consolidation of biopolymeric precursors using the casting technique. (a) Residual stresses arising from drying can deform significantly the substrate upon which the biopolymeric precursor has been cast, due to the (b) coupling of interactions between the building blocks and the substrate. (c) Properties of the resulting material as well as the quality of the casting procedure can be controlled by manipulating the colloidal interactions with plasticizers and tailoring the water contact angle of the substrate. (a–c) Adapted with permission from ref (491). Copyright 2019 John Wiley and Sons. (d) Biopolymer–substrate interaction may be strong enough to deform the colloid, as is the case of mechanically fibrillated CNF. This was shown by measuring AFM profiles of CNF cast on PEI treated mica (strong interaction) and pure mica (weaker interaction). Adapted with permission from ref (119). Copyright 2019 American Chemical Society. (e) Engineered substrates were produced to infuse patterns on peeled silk films (f) in order to create diffractive optical element (DOE) devices (g).(e–g) Adapted with permission from ref (711). Copyright 2017 John Wiley and Sons. Note: Further permissions related to the material utilized in (d) (pubs.acs.org/doi/abs/10.1021/acs.biomac.9b00432) should be directed to the American Chemical Society.

Whereas interactions among the material’s components are virtually independent of the casting process, physical forces developing during consolidation (e.g., capillary, wetting or vacuum-induced dragging forces) are responsible for a wide variety of features in the assembled materials, such as their microstructure and cohesion. For instance, capillary forces developing during solvent removal can be high enough to induce phase separation in multiparticle systems where the components display unfavorable interfacial interactions or geometrical incompatibilities.^703^ In casting techniques, the interactions with the substrate cannot be neglected as they disturb the cohesive biopolymer-biopolymer interactions by coupling them with surface interactions at the solid–liquid interface (Figure 25a–c).^491^ When the balance of coupled interactions favors the biopolymer–substrate counterpart, residual stresses arise, deforming the coatings or films (Figure 25a).^491,704^ The effects of the drying stresses on the formation CNC films by casting has recently been demonstrated.^491^ The residual stresses could be relaxed by the addition of plasticizers, in this case PEG, which is known to weaken the CNC–CNC interactions (Figure 25b); however, their coupling with a hydrophilic substrate (e.g., glass), even in the presence of plasticizer, was strong enough to deform the substrate. On the other hand, no interaction took place between the CNCs and a superhydrophobic substrate, favoring the CNC–CNC interactions, thus resulting in a highly deformed film (Figure 25c). Therefore, a good balance between CNC–CNC and CNC-substrate interactions is key for the successful formation of films by casting (Figure 25b).

##### 6.1.2.1 Casting of Biopolymer Solutions

Several reports on the formation of films or coatings from dissolved biopolymers have been highlighted over the past decade. Biopolymers soluble in water or common organic solvents, such as alginates,^705^ chitosan,^706^ starch,^707^ lignin,^708,709^ soy,^708^ and silk^710,711^ proteins, and several others, have been converted into planar materials by promoting their consolidation from cast solutions. Supraparticle interactions develop at the later stages of consolidation, at higher solid fractions.

Alginates are one of the most reported biopolymers for the formation of materials following casting, additionally to several others consolidation routes. Their molecular structure allows cross-linking with divalent cations, for example, Ca^2+^, forming the well-known water-resistant egg-box structure. Inspired by algae (e.g., *Saccharina japonica*) that can bind water molecules even in high ionic strength, a recent study addressed the formation of superoleophobic coatings by casting alginate solutions and subsequently cross-linking with calcium ions.^705^ The substrate upon which the oleophobic coating was cast was first treated with polyethylenimine (PEI) to ensure electrostatic interactions with the alginate matrix. As the native seaweed, the coatings showed remarkable oil-repellency, with underwater contact angle of ∼160° for crude oil and sliding angle lower than 2°. The repellency arises from the ability of the Ca-alginate coating to hold water and keep its mechanical integrity, even under hydration/dehydration cycles and high NaCl content, which typically have water exclusion effects on hydrated materials.^705^

The adhesion of polyphenols can present limitations for the formation of materials that are peeled from a substrate if the wetting-associated interactions are not properly controlled. A series of lignin (either alkali or lignosulfonate), SPI and transglutaminase enzyme solutions were cast on polytetrafluoroethylene (PTFE) to prepare antioxidant films. Soy proteins are highly stretchable, while lignin polyaromatic structure offers antioxidant capacity. Transglutaminase was added to the mixture in order to catalyze cross-linking between the lignin and SPI, via activation of the amino (SPI) and hydroxyl (lignin) groups, and therefore increase the viscosity of the system; however, the addition of lignin acted as a lubricant and decreased the viscosity of the multicomponent system. The radical scavenging activity of the cast film improved at least 10-fold, when up to 10 wt % lignin was added. The tensile strength of the SPI films (ca. 4.5 MPa) increased constantly with either the addition (from 2 to 10 wt %) of alkali lignin or lignosulfonate, reaching ∼11 and 8 MPa, respectively. Elongation at break decreased drastically from 120 to ∼8% when adding 10 wt % of alkali lignin to the SPI matrix. These results indicate a strong self-interaction between the relatively pseudodendritic lignin molecules with the protein matrix.^708^

Casting on microfabricated templates has led to several interesting materials, for instance, for diffraction-centered sensor and optical devices.^710,711^ Designed, patterned silicon substrates (Figure 25e) were produced by using a software that simulates inverted light propagation from the plane in which the pattern should be created back to the plane where the diffractive optical element (DOE) is placed.^711^ To facilitate the fabrication, the templates were chemically modified to display a hydrophobic character. Then silk fibroin solutions were cast on the template to be later peeled, a technique that is called cast-and-peel soft lithography. The patterned DOE films from silk fibroin (Figure 25f) performed comparably with the glass counterpart (Figure 25g), with an advantage of being biocompatible, thus allowing applications in biomedicine. The hydration degree of the silk DOE films has an impact on its optical resolution, the signal-to-noise ratio moved from 4 when dried to ∼0 at 50% humidity, which correlates with the size of the patterned elements that swell when in contact with water (Figure 25g).^711^

Coatings and films from edible biopolymers, such as starch and gelatin, have been extensively investigated toward more sustainable, biodegradable food packaging and coatings.^707^ The latter efforts are similar to those preparing films from cast solutions, with a major difference that the coatings form by dipping the substrate, for example, a fruit, in the biopolymeric solution followed by drying, typically, at room temperature.^712−714^ The physical barrier, ∼700 μm thick, against oxygen and humidity created by the biopolymer monolithic coating has been shown to improve significantly the shelf life of food such as cucumbers and tomatoes without affecting their sensorial properties (e.g., taste and color). Chitosan has been also applied in the formulation of an edible, functional coating for food.^715^ The effects of additives (carotenoproteins and glycerol) on the mechanical properties and processability of chitosan films formed by casting were investigated. For instance, the addition of glycerol led to a remarkable increase in the toughness of the chitosan films due to an increase in the elongation from 30 to 140% while keeping similar tensile strength ∼20 MPa. More relevant to this review is that the cohesion of chitosan films could be manipulated by weakening the interactions with multivalent cations.^706^ Such versatility enables the utilization of chitosan coating for biological applications, for instance for injectable gels that dissolve or solidify in certain saline conditions.

Casting-centered consolidation of native dissolved cellulose involves harsh, more complex, solvent systems (e.g., DMAc/LiCl, NMMO) and dissolution conditions (e.g., below 0 °C), which have so far limited the broader use of dissolved cellulose in the formation of monolithic planar materials. However, coatings of cellulose model surfaces have helped significantly the understanding of interfacial interactions between cellulosic substrates and other molecules, such as proteins.^716^ Recently, dissolution of cellulose toward its conversion into materials has advanced by using ionic liquids that can be recovered and recycled.^717−719^ The technologies on the subject have advanced significantly leading to several patents, as is the case of the dissolution process based on [emim][OAc] and mild temperatures.^720^ Cellulosic films were formed from cast cellulose solution, at concentration ranging from 2 to 14 wt %, using [emim][OAc] as the solvent. The solutions were cast in a heated glass container, and then coagulated in water, ethanol and their mixtures. The resulting films were transparent and presented tensile strength up to 100 MPa and elongations of up to 30%.^718^

##### 6.1.2.2 Casting Biocolloidal Suspensions

When forming materials by casting, high cohesion of nanochitins and nanocelluloses arise from a synergy between multiple colloidal interactions, for example, hydrogen bonding in cellulose nanofibrils, combined with physical entanglement and the formation of nanonetworks. Spherical biopolymeric colloids, such as those derived from lignin^721^ and cellulose,^263^ form planar materials from casting; however, their cohesion does not compare to that developed from high-aspect ratio colloids. Consolidation of such precursors into materials can be assisted/manipulated by external forces, such as those arising from filtration under vacuum or pressure,^212^ as well as magnetic fields.^722^ Consolidation assisted by filtration is more commonly seen in nanofiber processing with the length of the latter being larger than the filter mesh size. However, nanofiber suspension containing dissolved additives can retain the molecules only if high interfacial interactions take place or if the molecules are deeply infused in the fibers’ structure.^723^ In addition to the parameters controlled during the consolidation upon casting, post treatments are usually applied to modify the mechanical properties of the cast films. In the case of neat nanocellulosic films, hot pressing leads to denser films with higher mechanical properties,^724^ up to 3-fold higher. This is a result of the single fibrils forming the nanonetwork being closer to each other thus allowing additional hydrogen bonding.

In the case of CNFs and ChNFs, the properties of the cast films are typically governed by their dimensions and chemical composition^136,212,725−727^ with little influence on the materials properties coming from their interactions with the supporting substrate. The dynamics of consolidation under casting, however, can lead to various morphologies from nanocellulose precursors. Cast nanocellulose suspensions can consolidate into either dense or porous materials depending on the solvent used for their suspensions. Drying from water led to densification of the nanocelluloses arising from a combination of the capillary pressure of the water evaporation and disruption of the hydrogen bonding, while in octane the interfibril hydrogen bonding was not affected.^725^ Such morphological aspects are discussed later in this review.

The reason for the densification of the CNF films upon drying from water is the combination of the capillary pressure of the evaporating water and the disruption of the hydrogen bonding network at the intersections of fibrils by water Well preserved β-ChNF from squid pens, with a degree of acetylation (DA) of 99.3% and MW of 843 500 Da, has been converted into films by casting dilute dispersions (0.05 wt %), followed by vacuum filtration, and hot pressing in a sheet former (Rapid Köthen). The formed films displayed the typical morphology of materials formed from cast fibrillar colloids dispersion, namely a lamellar network of nanofibers. The orientation of the building blocks during consolidation was improved by the surface charges on the nanofibers. ChNFs with positive net surface charges (ζ potential ca. + 35 mV) repelled each other, thus favoring alignment to maximize their interparticle distance under the electrostatic repulsive forces and the filtration drag forces. Highly charged ChNF could also induce alignment and tighter packing in cosuspended colloids during consolidation.^728^ The well-preserved nature of the nanofibers and their high alignment led to tensile strength of >300 MPa. It was noted that the variations among the specimens was high, which does not typically happen for cellulose-based films. The acetyl groups at the chitin polymeric chain most likely led to mismatches in the hydrogen bonding network, creating regions where hydrophobic groups (acetyl) approached hydrophilic ones (hydroxyl). Similar observations were made for films created upon casting of well-preserved α-ChNFs from insect cuticles.^727^ Both reports have demonstrated that chitin nanomaterials can compete as far as mechanical performance with cellulose nanomaterials, provided that the extractions processes are well controlled.

Photonic materials from CNCs^483,491,603,729^ and ChNCs^592^ can be prepared by casting. Typically, such materials are cast on a rigid substrate to be later peeled and used as a free-standing material. The interactions between the crystals with the substrate must be optimized, as discussed earlier in this section, in order to form materials with controlled multiscale hierarchy. ChNCs form helical structures from the drying of cast dispersions. Their microstructure can be manipulated by the properties, such as charge and dimensions, of the colloids which in turn is tethered to the extraction conditions. Although some ChNC films do not form structural colors, their microstructure can be used as templates for other materials,^730^ or for sensing in the infrared domain. Ionic strength and pH of the medium have a major effect on the consolidation of chitin materials, and the same applies for consolidation under casting. The degree of electrostatic repulsion between the chitin colloids is dependent on both the acid concentration and the overall ionic strength, which typically leads to more accentuated effects with more dilute chitin suspensions. Overall, higher NaCl and HCl concentration led to higher pitch values due to intensified repulsive forces between the chitin colloids.^592^

Dispersion of nanoparticle drying over substrates tend to accumulate matter at the pinned edges, where evaporation rates are proportionally faster, resulting in variations in chemical potential and the formation of Marangoni flow, leading to the well-known coffee ring effect.^731^ In the case of chiral-nematic CNC films with structural color tethered to the pitch size, the presence of coffee rings typically shows a different color than the center of the film, due to variations in the density of CNCs as well as the pitch. Although not optimal for photonic applications, such features arising from coffee rings have been explored in modern designs to replace hazardous synthetic pigments.^477^ A compelling example of the interplay between science and art was demonstrated, where fundamental knowledge on the CNC assembly, and its interaction with the casting substrate, could be used to conceptualize artistic materials.^477^ Briefly, CNC suspensions were cast on substrates with same area but decreasing inner corner angle, from 90 to 27.5°, tracking the formation of the coffee ring. Decreasing the corner angle resulted in wider (up to 5-fold) coffee rings, which was explained by faster evaporation rates leading to stronger capillary forces. In another effort, homogeneous colored films, with high control over the coffee ring effect, have been achieved by producing arrays of CNC microfilms from spatially defined, nanoliter sessile droplet printing, and employing slow evaporation during self-assembly.^732^

Functional coatings can be formed from spherical biopolymeric building blocks, such as lignin nanoparticles.^721^ Dilute suspensions of polydisperse lignin particles were cast on hydrophilic substrates in order to investigate the effects of the consolidation kinetics on the size stratification across the thickness of the coating. Evaporation kinetics correlates well with the film nanonetwork, a topic that is addressed further in this review. More importantly, lignin interparticle interactions are not strong enough to allow self-standing films. However, casting of cosuspensions to form robust supracolloidal composite materials has been demonstrated in recent efforts using cellulose nanofibrils as a universal binder for particles.^479^ Multicomponent materials exploit synergies from each component, such as the strength of chemically and biologically inert CNF networks and a variety of functionalities from nanoparticles such as magnetism^733^ and photocatalyst.^734,735^

The high flexibility and high-aspect ratio of CNFs enable a conformation-dependent response to capillary stresses upon drying as was recently demonstrated.^479^ When drying with spherical particles of given sizes, from 20 nm to 40 μm, CNFs assembled either into 3D continuous or 2D planar nanostructures. Whereas a disconnected network was observed for particles with dimensions similar to that of the fibril diameter, a nanopaper network formed for particles much bigger (1000-fold) than the fibrils’ diameter. A continuous, well-intermixed, 3D network between fibrils and particles formed when particles with diameter 5 to 30 times the fibrils’ diameter were compounded. In this casting process, the early gelation of CNF, which happens around 2.5 wt %, entrapped the spherical particles within the nanonetwork, thus preventing separation of the two components. Cohesion is primarily transferred from the single fibrils to the whole construct by secondary interactions. Interestingly, high cohesion is achieved even considering the repulsive character between CNFs and hydrophobic particles, which is due to a compensation of the weak particle-fibril interaction by innumerous and strong interfibril ones.^479^ In sum, casting techniques, assisted or not, are versatile routes to fabricate biopolymeric materials from virtually any building block. The materials properties can be to a certain extent manipulated by the casting conditions (e.g., substrate and consolidation rate), but they are mostly and more easily tailored by the properties of the building blocks used, for example, surface chemistry and dimensions, as well as their formulation with other components.

#### 6.1.3 Regeneration

Regeneration involves dissolution of a polymer in a solvent prior to coagulation, either triggered by the removal of the good solvent or by immersion into a poor solvent. Although this bottom-up approach implies that the natural crystalline structures are lost in the case of cellulose, there are also advantages. One of them is the possibility of shaping the regenerated biopolymer on demand, in morphologies such as beads, fibers, sponges, membranes and other bulk materials.^22^ One of the major drawbacks, however, is that the maximum solid content in solution that is processable is considerably lower than those of biocolloids as also discussed in relation to Section 5.1.

##### 6.1.3.1 Regenerated Silk Fibroin

**Figure 26:**
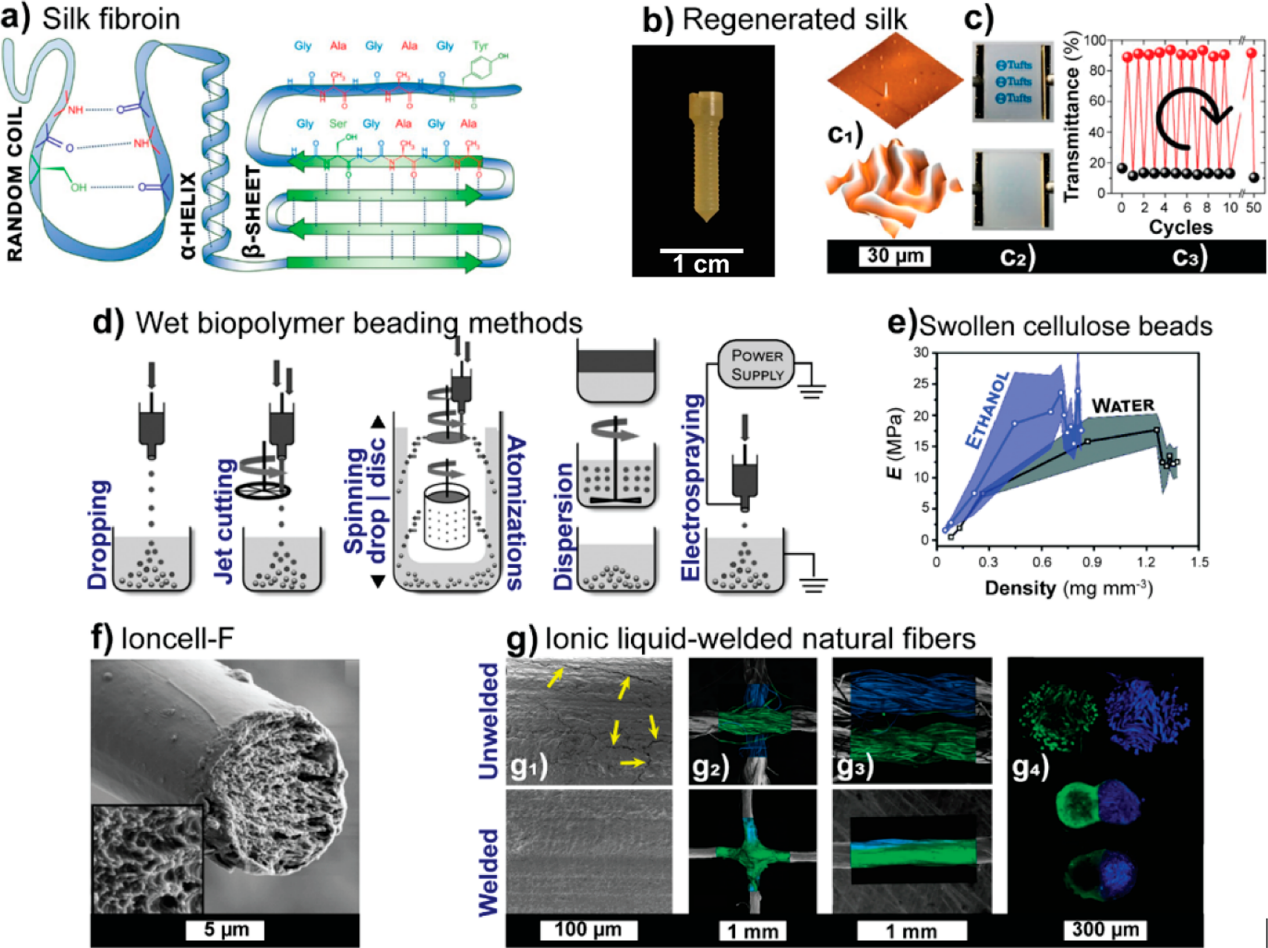
Materials from regenerated biopolymers. (a) Proposed structuring of regenerated silk fibroin, highlighting the secondary structures and hydrogen bonding among amino acid (glycine, serine, alanine, and tyrosine) residues. Adapted with permission from ref (737). Copyright 2019 National Academy of Sciences. (b) An object (screw) obtained from thermoplastically molded regenerated, amorphous silk pellets. Reprinted with permission from ref (736). Copyright 2020 The Authors under exclusive license to Springer Nature Limited. (c) AFM reconstructions (c_1_, height of ca. 150 nm) and actual pictures (c_2_) of transparent (top) and opaque (bottom) PDMS/regenerated silk fibroin bilayers in the unwrinkled (top) and wrinkled (bottom) states switched reversibly by (c_2_) electrical-to-thermal energy conversion or by (c_3_) multiple heating and cooling/water vapor exposure cycles. Adapted with permission from ref (737). Copyright 2019 National Academy of Sciences. (d) Regeneration-based wet protocols to produce biopolymer beads. Adapted with permission from ref (738). Copyright 2013 American Chemical Society. (e) Young’s moduli vs apparent density of water- and ethanol-swollen regenerated cellulose beads. Adapted with permission from ref (739). Copyright 2020 The Royal Society of Chemistry. (f) Representative SEM image of an Ioncell-F fiber (inset at 2.5× magnification). Reprinted with permission from ref (740). Copyright 2016 The Authors. (g) Unwelded (top) and IL-welded (bottom) natural fibers: g_1_) cross-sectional SEM images of delignified and pressed wood (voids in unwelded samples are indicated by yellow arrows); SEM and confocal fluorescent spectromicroscopy (CFM) images of orthogonally (g_2_) and parallel (g_3_) welded silk yarn; and cross-sectional CFM images of a side-by-side cotton yarn welded for 0 (top), 5 (middle), and 60 min (bottom). Adapted with permission from refs (741 and 742). Copyrights 2019 American Chemical Society. Note: Further permissions related to the material utilized in (g) (pubs.acs.org/doi/10.1021/acssuschemeng.8b05059) should be directed to the American Chemical Society.

Akin to cellulose, silk is a semicrystalline biopolymer spanning a network bound by strong interactions (Figure 26a), limiting melting and solubility to a high extent. The major protein in silk, fibroin, is commonly isolated from other cocoon components, mainly sericin, through a method known as degumming, followed by processing fibroin in solution into 1D, 2D, and 3D materials.^106^ Alternatively, a recent regeneration/pelletization protocol has enabled silkworm-derived fibroin to be processed as a thermoplastic, which would not be possible otherwise owing to the highly thermally stable β sheet crystals of native silk.^736^ In this method, amorphous silk pellets, ranging in diameter from 30 nm to 1 μm, were reconstituted from dissolved fibroin in the presence of concentrated LiBr. The all-aqueous regenerated, freeze-dried, and milled pellets were densified under heat and pressure into molded objects that were stronger than their solvent-cast counterparts (Figure 26b). The melt-state processing of regenerated amorphous silk resulted from bound water-assisted molecular rearrangement and self-assembly at high temperature and pressure.

In addition to parts, silk fibroin has been regenerated as a thin layer onto a soft PDMS substate.^737^ Reversible switching amid fibroin polymorphs (Figure 26a), in response to different stimuli, triggered reversible wrinkling from a smooth into a labyrinth-like topography (Figure 26c_1_). The approach of regenerating fibroin in a bilayer system of mismatched Young’s moduli creates in-plane compression strains at the nanoscale upon cooling and differential retraction. Exposures to water or methanol vapors affect the hydrogen bonding network and induce β sheet formation, while UV irradiation leads to photodegradation through weak C–N bonds, impairing crystallinity. These conformational rearrangements enable the reversible onset and erase wrinkling, rendering the material opaque and transparent in the wrinkled and unwrinkled states, respectively (Figure 26c_2_,c_3_). One of the applications envisaged for these systems is associated with smart windows.

##### 6.1.3.2 Biopolymer Beads

Once solubilized, regeneration of biopolymers into (*quasi*-)spherical particles ranging in diameter from a few μm to a few mm, have found application in stationary phase for size-exclusion chromatography, desalting, ion exchange, water remediation, protein immobilization, solid support for synthesis and drug delivery.^738^ Generally, after dissolution, biopolymer solutions are shaped by any suitable method into spheres, undergo a sol–gel transition, and are solidified into beads, by solvent removal or coagulation in a nonsolvent (e.g., water, ethanol, or acetone in the case of cellulose),^738^ with the possibility of encapsulating particles or compounds that are in the precipitating medium.^743^ Once solidified, the intermolecular interactions that are characteristic of native cellulose, that is, hydrogen bonds, vdW forces, and hydrophobic interactions,^744^ and that were hindered upon dissolution, are reestablished to hold the bead structure together.

The most widespread wet shaping methods of regenerated beads are outlined in Figure 26d, namely dropping, wherein bead volume and shape will depend primarily on the surface energy of the precursor solution and on the mechanical resistance offered when entering the coagulation bath (flattening), respectively; jet cutting, in which a high-throughput stream of the precursor solution is cut, for example, by a rotating knife, either in the air or dipped in the bath; atomization under a centrifugal field imposed by a rotating disc or a rotating meshed barrel, through which high yields are achieved by ejecting numerous drops geometrically controlled by spin rate and opening dimensions; dispersion, in which a high-energy-input device comminutes a biopolymer solution dispersed as droplets within the coagulation bath, where coalescence is typically prevented by added surface-active molecules, the mechanical and physicochemical forces dictating bead volume;^738^ and electrospraying, which relies upon an electrostatic potential between a nozzle, where the wet precursor is atomized, and the gelation bath, where spherical droplets are regenerated as beads bearing finely controlled shape and size.^745^

Electrospraying, specifically, is similar to electrospinning as a means of shaping biopolymer solutions/suspensions into elongated fibers, but in this case droplet breakup arises from the electro-capillarity in low-viscosity precursor solutions, favoring the spraying of *quasi*-spherical beads. Electrospraying has been exploited in bottom-up assembly of beads from a range of biopolymers, including alginate and collagen,^745^ chitin,^746^ cellulose,^747,748^ SPI^747^ and zein,^749^ to mention a few. Maintaining the viability of species, such as human cells,^745^ is among the major advantages of electrospraying. The interplay among the electrical (e.g., voltage, electrode spacing, electrical conductivity and flow rate), cohesive (e.g., biopolymer/solvent pair and surface energy), and viscous forces (e.g., biopolymer/solvent pair, solid content, MW, temperature and flow rate in shear thinning solutions) can be modulated to tailor the dimensional characteristics of the resulting beads in a straightforward fashion.^748^

Regardless of the beading protocol, the shaping step usually determines the volume of the resulting construct, whereas its inner structuring, including density, porosity, pore size and distribution, and specific surface area, are typically dictated by the coagulation step.^738^ Although often neglected, drying also plays a critical role in the evolution of the bead nano/microstructure, as recently shown for cellulose regenerated from a LiCl/DMAc solution, solvent exchanged into ethanol or water. SAXS and WAXS evidenced different drying regimes for beads from these solvents.^750^ Interestingly, the mechanical properties of regenerated cellulose beads were monitored by AFM indentation throughout drying, and the foremost roles of the main factors were identified: solid content, intermolecular interactions and heterogeneity of the bead microstructure.^739^ While the effect of cellulose concentration is intuitive, one might expect the Young’s modulus of ethanol-swollen beads to be lower than that of their water-swollen analogues, given that the latter holds a higher effective cellulose concentration, same mass, but lower volume. However, the opposite was found (Figure 26e). Neglecting microstructural differences prior to drying,^750^ the diverging Young’s moduli is a manifestation of the stronger interactions of cellulose with water (via hydrogen bonding) than ethanol combined with self-interactions (via attractive vdW interactions) in water, as well as of the lack of a tightly bound layer in ethanol-swollen beads, which in turn leads to greater molecular friction and interlocking extent when in ethanol.^739^ At increasing solid contents in the water-swollen beads, the effect of microstructure raise in importance due to the structural transition into an heterogeneous network comprising large (ca. 10 nm) cellulose II aggregates interconnected by less associated cellulose chains.^739,750^

##### 6.1.3.3 Biopolymer Filaments and Fibrous Hydrogels

Man-made fibers have been fabricated for more than a century via dissolution and regeneration of biomass. The traditional Viscose and Lyocell process have drawbacks as solvents for cellulose and some of these issues have been addressed by Ioncell-F.^740,751^ These processes produce fibers that still do not reach the expected mechanical performance of the building blocks, namely, CNFs, as explained by the changes in the crystal structure upon dissolution. The allomorph II of cellulose can be bottom-up assembled into high-performance regenerated materials, but chemical cross-linking or reinforcement fillers are sometimes required. Yet, the Young’s modulus of native cellulose I is about twice that of cellulose II; the former typically leading to more robust materials.^22^ Still, the aforementioned spinning processes are highly scalable and of commercial importance, *vide* Viscose, Lyocell and cuprammonium Rayon. Differently from such fibers, ILs is used in the Ioncell-F process. The solubility of cellulose in these organic molten salts is attributed to the favorable interactions between the high density of hydroxyls from cellulose and the anions from the IL. These ions serve as hydrogen bonding acceptors, disrupting the intramolecular network that holds the cellulose superstructure. In the Ioncell-F method, cellulose is dissolved in ILs at mild temperatures (typically lower than 100 °C) and with less energy input and depolymerization, leading to a viscous dope that is then extruded, stretched in air, and uniformly regenerated in a coagulation bath. The alignment of cellulose chains in the machine direction (Figure 26f), arising from high shear at the die and from drawing (winding rate higher than extrusion rate), quenches upon spinodal decomposition in the antisolvent, allowing the molecules to densely pack and crystallize and for short-range cohesive forces to dominate. This renders Ioncell-F fibers more birefringent, more oriented, tougher and stiffer than Viscose fibers and similar in properties to the Lyocell fibers.^740^

In biocolloidal precursors, regeneration often comes at the expense of the native structuring, posing a potential drawback of dissolution as far as mechanical performance. As option is that of welding through partial dissolution. This results in a more intimately packed network that improves the interactions among fibers while still benefiting from the innate hierarchical architecture and properties in the core. ILs, for instance, have been exploited lately to swell and to (partially or superficially) solubilize natural fibers, creating sheaths that feature enough mobility to interact more intimately with adjacent fibers.^752^ As far as cellulose fibers, while one may expect individual fibers to be weakened due to the conversion of highly crystalline cellulose I into less ordered cellulose II, the structural integrity of the fibrous network is likely to be improved through more extensive interactions, leading to reinforced materials. Indeed, ionic liquid-welded, hot-pressed CNF films were found to be surface patterned, more transparent, stiffer and tougher than the untreated film.^753^ A similar IL welding approach was used to strengthen all-cellulose composites, wherein untreated samples performed weakly due to voids (Figure 26g) that were removed once penetrated by the solvent, which in turn solubilized the outer surface of the fibers, melting cellulose I crystals and expanding the hydrogen bonding network among adjacent fibers.^742^ The welding extent is controlled primarily by the temperature (controls mostly swelling and dissolution rates), time (controls mainly penetration depth and net dissolution extent) and pressure plus the characteristics of the solvent itself, such as chemical nature and concentration.^752,754^

**Figure 27:**
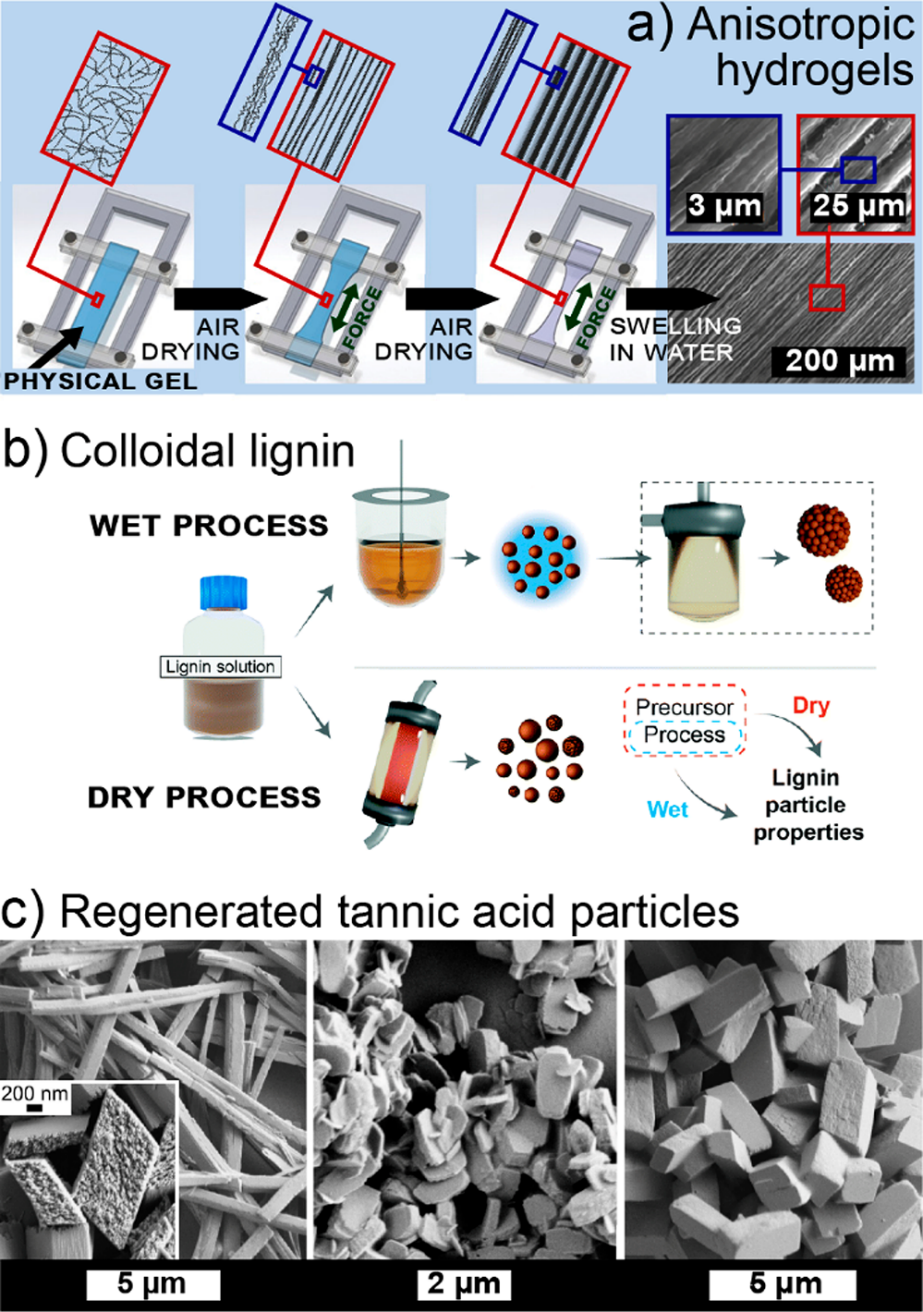
Materials and nanoparticles from the regeneration of biopolymers. (a) Scheme of the ‘drying in confined condition’ to produce highly aligned fibrous hydrogels mimicking natural tendons and ligaments. Adapted with permission from ref (755). Copyright 2018 John Wiley and Sons. (b) Method for fabricating colloidal lignin via precipitation of wet particles through solvent exchange, possibly followed by spray drying into superstructured clusters (wet process) or atomization into dry micro/nanosized particles (dry process). Adapted with permission from ref (451). Copyright 2020 The Royal Society of Chemistry. (c) Generic means of directing the assembly of tannic acid solutions into regenerated particles of varying aspect ratios, yields, and volumes by tuning solution pH, base conjugate acid p*K*_a_, and base countercation ionic radius, respectively. Adapted with permission from ref (756). Copyright 2019 American Chemical Society.

Besides spinning or extruding biopolymeric dopes and inducing alignment, for example, into yarns, anisotropy is achievable by restricting the macromolecular rearrangement when drying stresses are developed upon solvent removal. This is the case of cellulose or alginate physical gels that are air-dried under confinement in the axial direction.^755^ As shown in Figure 27a, an isotropic physical (hydro)gel specimen was kept with the ends clamped to a static holder and was allowed to dry, which led to volume contraction, shrinkage and macromolecular alignment along the axial direction. Further drying created thin nanofibrils that are then bundled into thicker fibers, which remained stable even in the reswollen gel, due to extensive hydrogen bonding. The high alignment and tensile stresses developed along the length direction led to structural materials mimicking natural tendons and ligaments.

##### 6.1.3.4 Phenolic (Nano)particles

Lignin and tannins are polyphenolic molecules that can be easily converted into colloids for the formation of hierarchically structured materials and systems. Several of the colloidal features, as far as morphology and chemistry, are tethered to the formation pathways taken and associated processing conditions.

Technical lignins are typically not soluble in water, but highly soluble in a variety of organic solvents and bases. The regeneration of lignin solutions into nano and microparticles has been accomplished mostly by aerosol flow or solvent exchange techniques (Figure 27b).^451^ In the aerosol flow method, a dilute lignin solution (up to 5 wt %) in the appropriate solvent is atomized into a heated laminar flow chamber under the flow of an inert gas (e.g., N_2_), in which the solvent is slowly removed to form spherical dried particles. The selection of the solvent depends on the lignin type, but usually includes high pH water, acetone or DMF, resulting in regeneration from room temperature up to 150 °C. Therein the size of the obtained spheres varies with the processing conditions (e.g., the intensity of the atomizer), but mostly with the concentration of the initial solution.^452^ The surface chemistry of the resulting particles is fully tethered to the initial precursor. For instance, biorefinery lignin from hydrothermally treated wood led to less negatively charged particles (ζ potential of −35 mV) when compared to Kraft lignin (ζ potential of −50 mV), which is due to its lower content of hydroxyl and carboxyl groups that contribute to the net charge around the particle under deprotonation.^217^

Manipulating the drying kinetics under the heated laminar flow can lead to the formation of drying stresses exceeding the critical buckling stress of the drying lignin droplet, thus leading eventually to a collapsed structure that can be rougher or completely wrinkled. The drying stage could be engineered by a proper selection of the solvent (ammonium hydroxide) and the use of a blowing agent (ammonium carbonate). This is a classic example of the same process (i.e., aerosol flow reactor) and precursor (Kraft lignin) leading to different outcomes depending only on the manipulation of few parameters.^473,757^

Solvent exchange (or shifting) is also a widely reported process for the regeneration of lignin macromolecules into colloids. Such process also harnesses the solubility of lignin for its regeneration. In a typical procedure lignin solution is poured into an antisolvent until the system reaches a solubility threshold that precipitates the particles. The lignin solution goes through a metastable phase very quickly, where the higher MW lignin macromolecules aggregate first, due to their reduced water solubility, followed by subsequently smaller entities. THF and acetone are typical solvents, whereas water is the most utilized antisolvent. The resulting particles are obtained in a colloidal suspension that can be further spray dried to form lignin supracolloidal structures.^758^ With the solvent exchange process, the initial precursor lead to similar lignin particles, as far as morphology and surface charges; however, the processing parameters heavily influence their properties and especially those related to particle dimensions and size distribution. The pH of the antisolvent has a negative correlation with the particle size, with sizes being controlled from 250 to 50 nm only by adjusting the pH from 3 to 7. The order of addition of the lignin solution into the water or water into lignin solution plays a key role if the addition happens slowly. Slow addition of water into solution leads to particles at least 4-fold larger than those produced from fast addition with the same concentration and precursor.^759^

Controlled depolymerization and reassembly of tannin acid, TA, has been shown to induce precise control over the morphology of the formed TA particles (Figure 27c).^756,760^ Controlling the oxidation kinetics of 2 wt % TA solutions by adjusting the pH from ∼8 to 11, using KOH, NaOH, LiOH, Na_3_PO_4_ or NH_4_OH and letting the reaction occur over few hours to up to 1 week. The countercation ionic radius correlated negatively with the particle volume, whereas the base strength and the initial pH of the solution correlate positively with the yield and aspect ratio of the particles. The latter leading to highly elongated particles.^756^

#### 6.1.4 Self-Assembly in Confined Spaces

Here we refer to confined self-assembly of biopolymers precursors consolidating within well-defined spatial boundaries, in the nano- and micrometric length scales. This assembly is reminiscent of that found during biosynthetic processes, where higher order structures are generated. Confinement in reduced length scales harnesses the effects arising from geometric constrictions,^761^ interfacial interactions,^762^ and consolidation directionality^763^ to systematically study the biopolymeric self-assembly found in nature^172^ and to further advance biomimetic materials and systems.^113,492^

**Figure 28:**
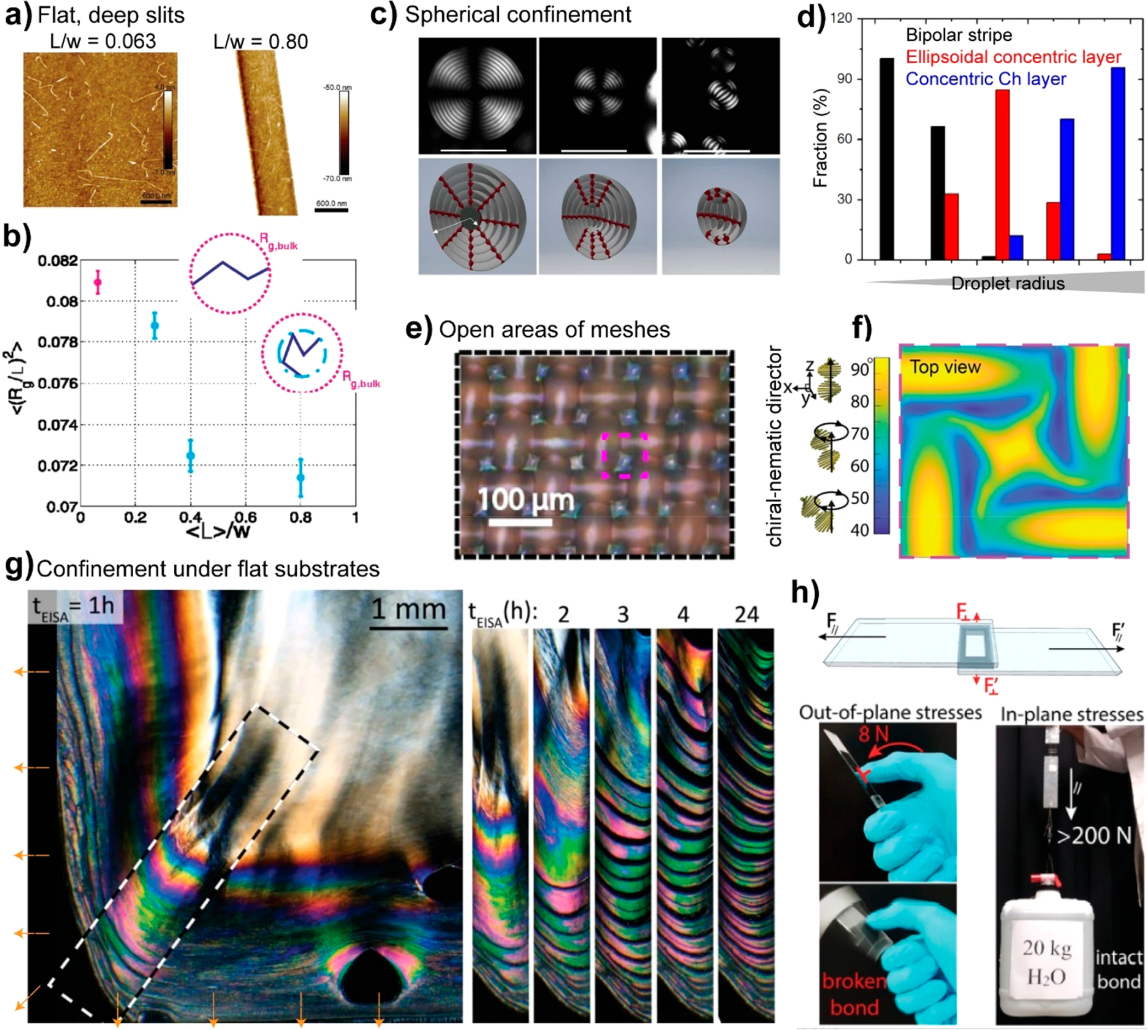
Behavior of biopolymeric building blocks when consolidated under confinement. (a) Alignment of long biocolloids upon casting a dilute solution on engraved substrates. The alignment index (b) is tethered to the ratio between the length of the colloid (L) and the width of the engraved valley (w), where long biocolloids tend to self-fold, due to their soft nature, depending on the geometrical constrictions imposed by the confinement. Adapted with permission from ref (761). Copyright 2019 John Wiley and Sons. (c) Observation of the CNC tactoid formation when confined in spherical geometries as a function of the sphere radii. (d) Formation of liquid crystalline phases and associated fractions depending on the size of the confinement. Adapted with permission from ref (765). Copyright 2016 The Authors. (e) Microtemplated assembly of CNCs within open areas of Nylon meshes allows a higher control over the (f) chiral nematic director across, and within the template and its confined areas. Adapted with permission from ref (491). Copyright 2019 John Wiley and Sons. (g) Assembly of CNC between planar surfaces aiming at highly anisotropic assembly, in which a (h) high adhesion arises from extensive alignment of CNC depending perpendicular to the drying front, leading to stratified superstructures. Adapted with permission from ref (492). Copyright 2020 John Wiley and Sons.

Assembly under confinement provides structural information on the possible topologies of constrained components.^113^ Asymmetrical particles are more extensively affected by confinement as to favor organizations of the element matching spatial features. The high-aspect ratio TO–CNFs tends to naturally change their morphological features to match the spatial conditions imposed by micrometric confinement,^761^ and it was shown that TO–CNFs displayed sharp bends (kinks) in between stiff segments, leading to self-folding, which can be used to change the function under confinement.^144^ TO–CNF suspensions were deposited on a flat substrate containing slits (height, *h* = 60 nm and width, *w* varying between 0.75 and 9.50 μm) (Figure 28a). The ratio between the contour length of the fibril (*L*) and the width of the slit (*w*) determined the behavior of the TO–CNF. With *L*/*w* tending to unit, the fibril orientation within the confinement increased significantly, with *L*/*w* = 0.4 (slit’s width almost 2 times larger than the contour length of the fibrils) being the threshold for a notable orientation degree. Overall, given their flexible character, TO–CNFs tend to conform more, that is, higher kink angles (ca. 10%) and higher density of kinks (ca. 25%), over higher *L*/*w* (Figure 28b).^761^

Confinement in spherical shapes has been used to investigate the formation of cholesteric arrangements of CNCs in suspension and their consolidation upon evaporation induced self-assembly (EISA). Under spherical confinement, the director of the cholesteric plane is tethered to a curved surface and the consolidation arises from the spherical shells and inward.^764−766^ The effects of the radius of curvature of the confined space on the cholesteric phase of CNC suspensions, as well as their lyotropic properties were demonstrated. It was observed that a phase separation of the initial suspension into an isotropic core and a cholesteric shell comprising concentric CNC layers rapidly took place, with the ratio between the two well-defined structures correlating to the radius of the droplet, that varied from 40 to 155 μm (Figure 28c). Droplets with radius (*R*) much higher than the pitch (P), exhibit cholesteric structures similar to the macroscopic, nonconfined CNC films. However, CNC cholesteric shell layers organized concentrically in droplets with *R* < 40 μm (Figure 28d). Ellipsoidal arrangements were observed, and such transitions are imposed by the radius of curvature to which the director is attached to, that is, if the curvature is too high, concentric mismatches (radial defects) must take place to allow a proper packing. At the smallest droplet size, a bipolar planar cholesteric structure was found.^765^ It was later shown that a spherical confinement induces a kinetic arrest of the EISA, where a shell forms and the sample cannot fully relax, leading to a buckling of the dried sphere of self-assembled CNCs.^764^

The consolidation of CNC suspension in acoustically levitated droplets, used as a means to quantitatively measure structural features of CNCs assembling, was studied.^600^ For this purpose, they used time-resolved small-angle X-ray scattering (SAXS),^600^ and measured the distance between CNC particles at different stages of the assembly. It was found that upon drying, the CNC-water interactions are overcome by the CNC–CNC supraparticle interactions. For instance, the CNC–CNC separation distance decreased from 50 to 5 nm as the concentration increased from 1 to 35 vol %. This implies that short-range interactions in CNC assemblies take place at later stages of consolidation, with significantly high solid fractions, higher than their gelation point. It was also observed that there is a compression of the pitch toward the inner regions of the microdroplet,^600^ that derives from the radial, concentric compression of the nematic CNC structures, corroborating previous studies discussed above.^764,765^

Confinement imposed by solid boundaries significantly affects the behavior of biopolymeric building blocks upon consolidation. Coupling the interactions between the biobased building blocks and the adjacent surfaces is expected to take place, hindering, changing, enabling, or favoring the formation of given microstructures. The self-assembly of CNC suspensions confined in the micrometric open areas of hydrophilic meshes (Nylon-66) was evaluated. CNC planar materials were built on the solid support to display a single consolidation front that was perpendicular to the mesh. However, when suspended within micrometric grids, the consolidation of the CNC suspension took place from both bottom and top air–water interface, leading to a vertical contraction of the gelled systems toward the center. A balance between hydrogen bonding in CNC–CNC and CNC–mesh, during consolidation, drove the assembly, structure, and formation. A deformed cholesteric phase was observed in the films, which suggested the stretching of the tactoids in the latter stages of film formation. Additives, surfactants [sodium dodecyl sulfate (SDS) and cetyltrimethylammonium bromide (CTAB)] and NaCl, prevented the chiral nematic order of the confined CNC films, mostly because they altered the CNC–CNC, CNC–mesh coupled interactions, for instance, leading to spreading of the suspension on the mesh (SDS) or formation of CNC aggregates (CTAB).^762^ Subsequently, it was showed that the assembly of CNCs in the confined areas of meshes led to higher ordered assemblies, resulting in a better control and tunability of their photomechanical properties (Figure 28e,f).^491^ Chiral-nematically ordered structures, confined in mesh opening areas were tiled into hierarchical structures via topographical templating. An underlying rigid substrate, with controlled wettability, was used for coupling of CNC–CNC and CNC–substrate interactions. In this type of confinement, CNCs interacted with the surrounding mesh filament (Nylon-66) and with the underlying substrate, thus consolidating mostly from the top air–water interface. The underlying substrates, with superhydrophobic character, WCA > 150°, resulted in a buckled and deformed material because of the absence of CNC interactions with the rigid substrate, to alleviate drying stresses. CNC interactions, aligned with drying residual stresses, were strong enough to deform soft templates, extensively pulling the mesh filament toward each other. Therefore, controlling the capillary stresses was key to anchor the orientation of the chiral-nematic director across the topography of the template.

Under spatial confinement, one can engineer the direction of solvent removal and manipulate capillary forces, hydrostatic pressure and transport phenomena (e.g., permeation), which consequently affect the collective behavior of the system. Such phenomena tether the spatial organization of the building blocks to the drying front, for instance, to align high aspect ratio colloids perpendicular to the drying direction. Confined assembly of CNCs in capillaries with rectangular microscaled aperture (height: 50 μm and width: 1 mm) has been performed to investigate orientation under directional consolidation by extensive water evaporation.^763^ Whereas the confined suspension evaporates at slow rate at the top meniscus, because of the presence of saturated vapor, the evaporation is faster at the bottom, causing asymmetry of drying and a fast formation of liquid crystal structure on the side with the faster evaporation rate. Orientation of the liquid crystal layers is caused mostly by capillary pull, with negligible influence of sedimentation. EISA was faster than typical dish-cast films, taking only ∼2–8 h to form materials with similar photonic properties.

Building block alignment is intensified under confinement, which can be harnessed to synthesize a variety of advanced materials, for example, for sensing and structural adhesion. Confined assembly induced nematic orientation in CNCs to develop piezoelectric properties,^767^ since otherwise the chiral nematic assembly would cancel out the polarization, leading to weak or no piezoelectric response.^768,769^ In this effort, the authors used a confinement cell built with superhydrophobic walls, with a single consolidation front, and ethanol as additive to manipulate the CNC–CNC and CNC–substrate interactions. The negligible CNC–substrate interactions allowed for CNCs to move freely and orient perpendicularly, because of the torque caused by shear forces with respect to the consolidation front. The addition of ethanol to the aqueous CNC suspensions reduced the repulsive electrostatic interaction between CNCs that otherwise is a main factor for their assembly into chiral-nematic arrangement. By increasing ethanol fraction from 0 to 90%, the ζ potential of CNC changed from −62.4 to −7.7 mV, which resulted in transparent solid films without the characteristic iridescence color of CNC chiral-nematic assemblies. Therefore, by confinement and simple manipulation of the interactions between the systems components, it was possible to reach highly aligned CNC-based films with piezoelectric performance, with a piezoelectric coefficient d_33_ of 19.3 ± 2.9 pm/V, comparable to the of poly(vinylidene difluoride) (PVDF) (20–30 pm/V).^767^

By adjusting CNC interactions with the surrounding surfaces it was possible to produce high performance superstructured bioadhesives that mimic those observed in nature (e.g., with microstructures mimicking gecko feet).^492^ These CNC systems, assembled under confinement, displayed a remarkable adhesiveness for hydrophilic substrates (Figure 28g). The confinement configuration allows four micrometric consolidation fronts where two thin flat surfaces overlap the larger surface. CNC suspensions consolidating between two flat, hydrophilic substrates induced alignment by shear stress, associated with the directional drying.^763,767^ Adhesion is a result of capillary forces overcoming water layers bound on the CNCs to generate multiple and coupled CNC–CNC–substrate secondary interactions. The confinement induces nematically ordered lamellae (Figure 28g) across multiple length scales that mimic the arrangements of setae of insects onto the gecko feet. In turn, this leads to a remarkable anisotropic adhesive strength (≈ 7 MPa in-plane and (≤0.08 MPa in the out-of-plane direction), with an areal density of CNC as low as 20 μg cm^–2^ (Figure 28h). CNC–substrate friction forces are key for such anisotropic structural adhesion, from the confined assembly of CNCs. This can be further tuned by simple changing the CNC density, which results in a gap between the lamellae, varying in size and, therefore, in adhesive performance. Other efforts have shown the use of lignin nanoparticles (LNP) consolidating under similar geometrical containments, producing a shear strength of ∼250 kPa.^770^

In conclusion, confined systems offer possibilities to understand the self-assembling mechanisms of a range of biopolymeric colloids, and to develop high performance functional materials. By using confined assembly, one can engineer interesting attributes, such as consolidation directionality, impose geometric constrictions to favor given arrangements, and to harness interfacial interactions for various purposes.

#### 6.1.5 Biopolymer Compositing and Blending

In this section, we use the term blend or composite to refer to materials prepared from primary components that are not necessarily optimized for given interactions. Biopolymers have been blended with several other polymeric matrices, biodegradable, nonbiodegradable, biobased or petroleum-based, for the preparation of composites targeting high-performance, cost-efficiency or environmental-friendliness.^771^ Within such composites or blends, biopolymers act as fillers or reinforcement agents, depending on a series of factors that are heavily influenced by processing and intercomponent interactions. The terms blends, therefore, broadly includes composites and multilayer materials. Generally, “filling” is used to reduce material costs and to improve sustainability, for example, by partially replacing a synthetic component with a biobased one. The use of biopolymers as reinforcing materials improves properties such as mechanical strength, allowing lighter but stronger materials. The degree of purity of a biopolymer impacts the end goal. For instance, for filling purposes, a less pure, less processed biomass may be preferred, while for reinforcement purposes, more refined and nanoscaled biocolloids might be better.

##### 6.1.5.1 Blending for Cost and Sustainability

In contrast to the assemblies discussed so far, the biopolymer–biopolymer interactions are usually detrimental for efficient blending, within the most applied polymeric matrices, that is, polypropylene (PP) and polyethylene (PE). Biopolymers widely used in blends and composites are typically hydrophilic and thus, incompatible with hydrophobic PP and PE. The interactions among the biopolymers is usually far stronger than their interfacial interactions with other blend components, leading to aggregation and phase separation. Chemical modifications, as already discussed, have been exploited to favor interfacial interactions between biopolymers and hydrophobic matrices. Such modifications typically end up decreasing the biopolymer self-interactions, for example, interfibril hydrogen bonding in the case of cellulosic building blocks.

Wood polymer composites (WPC) is the most conventional type of material explored in the context of biopolymeric blends. WPC comprises raw wood from sawmill or residues from other industrial processes, combined or infused with hydrophobic polymeric matrices. Research on the subject is quite mature, dating back to at least 80 years ago, with several products reaching the market over the past decades. Currently, research on the subject focuses mostly on technical aspects, such as optimization of processing parameters and formulation,^772,773^ public perception and market acceptance,^774^ end-cycle, recycling and environmental issues.^775^ Natural fiber composites (NFC), which are materials similar to WPC, have been also explored as a means to upcycle agriculture side-streams. Examples of fibers that have been used include those derived from banana, sisal, grape pomace, coconut, sugar cane bagasse, heart-of-peach palm, mate tea, and several others.^771,776−781^ Polyphenolic biopolymers such as tannin^782^ and lignin,^783−786^ which display some degree of amphiphilicity, have been utilized as compatibilizing agents and as thermal stabilizers in wood polymer composites. The mass fraction of biopolymer in such blends varies from 20 to 50%; however, given their relatively higher hydrophilicity, detrimental effects related to water absorption and swelling of the blend appear at high biopolymer fractions.

Technical lignins have been blended with a variety of synthetic polymers. For instance, commercial alkali lignin and lignin extracted from almond shell using organosolv processes were blended (loadings ranging from 0.5 to 20 wt %) with poly(lactic acid) (PLA) matrices using a extrusion process. Both lignins were acetylated in order to improve their interactions with PLA. Overall, with unmodified lignin, aggregation across the blend was observed, even at low solid fractions (5 wt %); however, with acetylated lignin, no aggregation took place. Clearly, the introduction of acetyl groups hinders lignin–lignin interactions, for example, hydrogen bonding, thus improving the dispersibility within the PLA matrix. Interestingly, blending with lignin improved the elongation of the PLA, from 2 to 6–8%.^787^ Lignins, either modified or as-extracted, were blended with polyolefins to endow antioxidant and thermal stabilization. Lignin hydrophobization was effective to achieve a good dispersion and led to oxidation induction time (OIT) over two times higher for a PP film containing 1 wt % of lignin (with 50% OH replaced by butyl group) when compared to neat PP. The lignin/PP blends also displayed aging resistance, over 500 h as measured by the density of carbonyl groups under accelerated aging, compared to neat PP (250 h).^788^

It was demonstrated that the lignin particle size has a great influence over the properties of the blend. Irregular lignin particles in aqueous suspensions, with primary sizes from 5–10 μm, were ball-milled (at ca. 80 rpm) and sprayed or oven-dried. Two particle size distributions were obtained and further used to formulate blends with PP (with lignin loading fixed at 10 wt %), one narrower with average at ∼2 μm and the other more polydisperse, ranging from 1 to 100 μm. The particles became, on average, bigger when blended with PP due to weak interactions with the polymer and strong self-interaction, thus creating aggregates that could be 10 times bigger, for instance 20 μm aggregates, compared to the size of the smallest particles (i.e., 2 μm). The smallest particles aggregated into bigger clusters, which led to poorer mechanical performance. Lignin-PP blends, featuring well-distributed particles across the PP matrix, performed similarly to the neat PP; in fact, an increased Young’s modulus, from ∼850 to 1050 MPa, was observed but with a reduced elongation, from 14 to 12%.^789^ Interestingly, lignin nano and microparticles can be produced in relatively large scale with fine control over size, surface chemistry and morphology.^451^ This will warrant significant advances in the utilization of lignin biocolloids in polymeric blends.

##### 6.1.5.2 Blending/Compositing for High Performance

**Figure 29:**
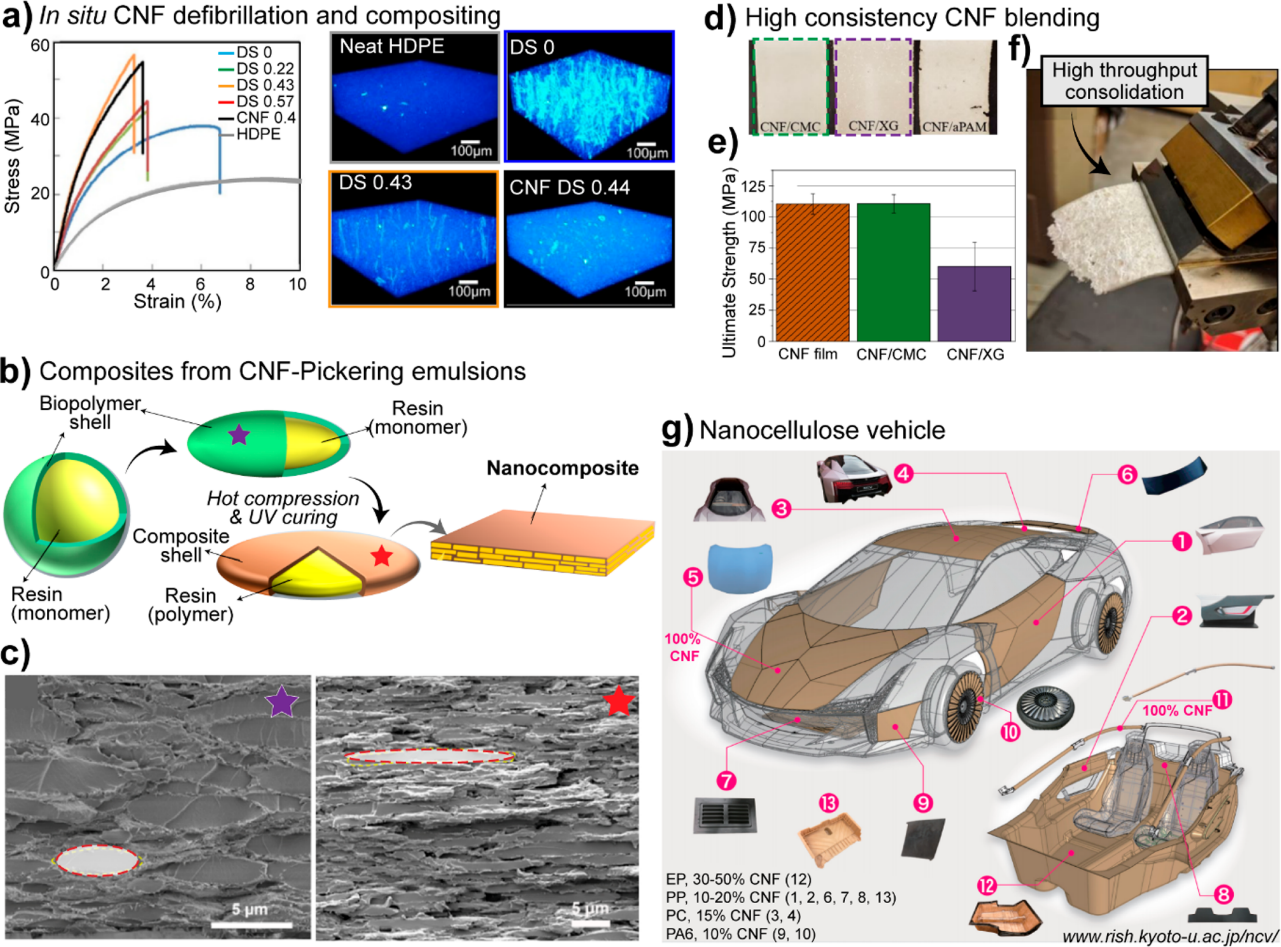
High performance biopolymeric blends. (a) High mechanical performance of HDPE-CNF blends is achieved by partial acetylation of the CNF *in situ*, which leads to better distribution of the nanofibers across the polymeric matrix during screw extrusion. Adapted with permission from ref (793). Copyright 2018 Elsevier B.V. (b) Nanocellulose in Pickering emulsions stabilize resins to prepare strong and tough composites. (c) The reinforcement character is tethered to the size of the nanocellulose and the size of the resin oil droplet created upon emulsification, upon compression by hot pressing and after filtration. Adapted with permission from ref (798). Copyright 2019 American Chemical Society. (d–f) High consistency CNF precursors can be extruded into films by the assistance of low-MW biopolymers (gums) and cellulose derivatives. The (e) resistance and (f) morphology of the final construct is affected by the composition of the blend. Adapted with permission from ref (676). Copyright 2020 American Chemical Society. (g) Developments in polymeric blending with nanocelluloses is displayed in the nanocellulose vehicle, with many of its components built from nanocellulosic materials or their blends (rish.kyoto-u.ac.ja/ncv/).

Blends comprising biobased nanoparticles, for example, CNFs, ChNFs, CNCs, and ChNCs, have been proposed for the fabrication of high-performance materials.^790^ There is an extensive literature on surface modification of nanocelluloses, for uses in reinforcement of, for example, hydrophobic matrices, targeting at an improved wettability of the polymeric matrix that enhances stress transfer mechanisms across the material.^791,792^ Efforts to utilize CNFs have attracted industrial attention and prototyping efforts have become popular. The ubiquitous presence of water in such colloidal dispersions limits direct blending with hydrophobic, thermoplastic polymers. Therefore, attempts have been directed in the area of defibrillation of dried pulps with a polymer *in situ* or to dry nanocelluloses in such a way to facilitate redispersion. For instance, a method to defibrillate dried pulp into CNF *in situ*, by direct kneading with high-density polyethylene (HDPE) using a twin-screw extruder, was developed in the so-called Kyoto Process (Figure 29a).^793^ The latter involves a chemical modification step using refiner-treated pulp with alkenyl succinic anhydride (ASA), a typical paper sizing agent, in *N*-methyl-2-pyrrolidone. Further control over the CNF–polymer interface was achieved by esterifying the CNF surface to display a series chemical structures.^794^ The modified pulps were mixed with MAPP, CaCO_3_ and HDPE powder in isopropanol, vigorously mixed, filtered to 50 wt % solids, and then dried under mixing. CaCO_3_ was added to preserve the chemical modification during melt compounding. Additional HDPE was introduced in the mixture for a final CNF content of 10 wt %. It was shown that the DS of the reaction with ASA played an important role in the *in situ* defibrillation of the pulp and the distribution of the resulting nanofibrils across the polymeric matrix (Figure 29a). ASA modification partially suppressed the multiple hydrogen bonding between elementary cellulose fibrils when subjected to drying, therefore allowing them to disassemble into single fibrils more freely. As expected, a better distribution and defibrillation led to higher mechanical properties, given that CNF aggregation would otherwise produce a poor performance. Tensile strength of the CNF/HDPE blends was ∼50 MPa, compared to 20 MPa of the neat HDPE (Figure 29a).^793^ Related efforts were carried by blending unmodified CNF with PP; however, to induce interfacial adhesion, MAPP was modified to produce a cationic polymer with primary amino groups (CAPP).^793^ CNF/PP blends were also successfully prepared by using spray-dried CNF^795^ as an attempt to overcome water-association during processing. Many attempts have been made to develop efficient ways to dry CNF and to understand their interactions with water,^10,796,797^ mostly because their potential for utilization in high-performance polymeric blends, typically involving hydrophobic components.

The ability of CNFs and CNCs to stabilize multiphase liquid systems (emulsions)^19^ has been recently harnessed to prepare transparent, mechanically strong blends that are capable of precisely manipulating light diffraction for photonic and optoelectronic applications.^798^ Sugar cane-derived nanocelluloses were used in Pickering emulsions with an acrylic resin (ABPE-10 monomer), that was further vacuum filtered to form a multiphase resin/nanocellulose mat on a PTFE filter membrane, which was dried for few hours at relatively low temperature (40 °C) (Figure 29b). The dried mats were then hot pressed (150 °C, 2 MPa, 5 min) between two flat surfaces and the morphology of the materials, before and after the compression, are shown in Figure 29c. A clear vertical flattening, and horizontal stretching, of the cured resin pockets encapsulated by nanocelluloses was observed, a structure that resembles that of nacre. The nanocellulose assembling at the oil/water interface played an important role to obtain a homogeneous distribution of the fibrillar reinforcement. Among all the blends, the combination of long and short nanocelluloses resulted in the highest tensile strength, ∼30 MPa. Nanocellulose networks were recognized as mechanically strong due to the multitude of interfibrillar hydrogen bonding, and physical entanglement, that transfers cohesion from the single fibrils to the macroscale constructs.^10,17,26^

CNFs has been recently blended with other biopolymers to continuously produce sheets under single-screw extrusion (Figure 29d–f). In such process, concentrated CNF slurries, at 25 wt %, were blended with small fractions (ca. CNF to additive of 10:1,) with CMC, XG, and anionic polyacrylamide (aPAM), among others. They were extruded to produce wet sheets that were hot-pressed to form robust biobased composites (tensile strength >100 MPa).^676^ The mechanical performance of such blends is still below that of TO–CNF films,^799^ but it is often higher than widely utilized synthetic polymers, such as PS and nylon.^800^

The potential of nanocelluloses in the synthesis of industrially relevant, high-performance materials, by blending with PP, has been showcased by universities, research institutions, companies and government. A concept vehicle, a car containing several components built from pure nanocellulose or blends with PP, polycarbonate (PC), polyamide and others is an example (see www.rish.kyoto-u.ac.jp/ncv/ and Figure 29g). The content of nanocellulose in the car elements varied from 10 to 100%, depending on the component, for example, engine parts, seats or hoods. The “Nanocellulose Vehicle”, as the project is called, showcases a realistic, high-performance and high-value technical application for nanocelluloses.

CNC has been widely utilized in blends.^801−805^ Its processing is rather similar than that utilized with CNF, but it requires less energy intake to promote their efficient dispersion across the polymeric matrix, given the CNC single-particle, nonentangled characteristics. For instance, spray-dried CNCs (150 nm long and 15 nm wide) were blended with poly(ethylene-*co*-vinyl alcohol) via melt mixing and melt-compounding. The same blend was produced following dissolution of the poly(ethylene-*co*-vinyl alcohol), which was used as coupling agent, in CNC-containing DMF, later dried under vacuum. Both batches were ground into powder and given fractions of PP were added to the mixture. The final blend was processed similarly as the masterbatches, but the consolidation was done by compression molding. The authors showed that despite the lack of compatibility between the CNCs and PP, the tensile Young’s modulus increased with the coupling agent content, reaching up to 50% increase (from 1000 to ca. 1600 MPa). Therein the CNC content was fixed at 5 wt %, a value below the percolation threshold at which the CNC interconnectivity is reached (to maximize the mechanical properties).^804^ The morphology of CNCs can be manipulated depending on how (if) it is dried (e.g., spray or freeze-dried), which has been demonstrated as a tool to tune the blend properties.^805^

Blends of hydrophobic polymers and nanochitins, both ChNFs and ChNCs, have also been attempted.^806−809^ Acrylic resin (AR) and TEMPO-oxidized ChNCs (TO-ChNCs) have been blended (AR/TO-ChNC) and further laminated with biaxially oriented PP (BOPP) aiming at high barrier properties for packaging applications. The initial blended suspension gelled when only 3 wt % of TO-ChNCs was used, which is likely a result of multiple hydrogen bonding taking place among the blend’s components, forming an interconnected and more rigid network. When blending with BOPP layers, at the macroscale, the presence of AR/TO-ChNC did not improve the barrier properties; however, layers created only with TO-ChNC resulted in a reduction of oxygen permeability, from 350 to <200 cm^3^μm/m^2^/day/kPa using a TO-ChNC layer of only 8 μm. This indicates that the interactions between TO-ChNCs are much stronger than those between AR and TO-ChNCs, and are able to form very tight, nonporous networks reducing the gas permeability.^807^ Like CNCs, ChNCs have been demonstrated to significantly affect the properties of PP in blends. With ChNCs, the properties reached a maximum depending on the nanochitin content, below 4 wt % to avoid strong chitin–chitin interactions.^808^

Chitins can be partially or completely deacetylated, the latter yielding chitosan. Several authors have investigated the effects of partial deacetylation on the formation of materials from chitin nanostructures.^810,811^ Controlling the hydrophobicity and surface charges of chitin colloids could be an effective pathway to tune their interfacial interactions with other polymers, for the formation of blends. On the other hand, one of the greatest advantages of chitosan is its water solubility, which allows efficient blending with a variety of water-soluble polymers, such as PVA. For instance, ternary blends of chitosan, PVA and PLA were prepared using O/W emulsions,^812^ with chitosan and PVA in the water phase and PLA dissolved in chloroform, the oil phase. The two solutions were emulsified, concentrated and used to cast blended films. The authors showed that blends containing more chitosan were stronger (ca. tensile strength of 30–40 MPa), but less extensible (<5% elongation) when compared to the other polymeric matrices (tensile strength <20 MPa, elongation >30%). Balanced hydrophilic interactions among the components, mainly chitosan-PVA, resulted in blends with tunable properties given the formation of homogeneous interfaces across the material.^812^ Similar materials were obtained by melt processing.^813^ Other biopolymers, such as starch, can be used in the same systems, without requiring significant modifications for processing.^814^

In conclusion, biopolymers can be blended with a variety of matrices including hydrophobic ones. The latter is challenging as biopolymers are typically hydrophilic and bind strongly to residual water. However, chemical modifications intended to manipulate interfacial interactions are accessible, and several outstanding contributions have been made along the past decade.

### 6.2 Cohesion Transfer and Assembly Strength

Within several natural organisms, such as trees, biopolymers display an extremely high affinity to each other, leading to highly robust materials that typically transfer cohesion from the molecular to the macroscale levels, via supramolecular interactions. Compared to synthetic counterparts, biopolymers are effective in transferring cohesion across length scales. CNFs and carbon nanotubes are great examples of cohesion up-scaling. While carbon nanotubes are remarkably strong nanoparticles, their assemblies may not form materials proportionally as strong, due to relatively weaker CNT–CNT interactions. On the other hand, cellulosic assemblies form strong systems even if compared with their primary building blocks, mirroring the more homogeneous interactions that occur at the molecular scale. Considering such remarkable ability to transfer cohesion, from the building blocks to their constructs, we discuss next the effect of the biopolymeric precursor size and surface chemistry in the formation of cohesive mono and multicomponent biopolymeric materials.

#### 6.2.1 Effect of the Building Block Size

High mechanical performance of primary building blocks, at the molecular or the nanoscale, is typically associated with their well-ordered, defect-free arrangements that are highly efficient in dissipating energy.^147^ Efficient transfer of cohesion across length scales can be only achieved through a controlled organization of such building blocks, for example, in well-defined multiscale architectures that minimize defects arising from higher order constructions.^480^ The materials’ architecture, and consequently the density and arrangement of interactions among the building blocks, is fundamentally tethered to the size of their elementary units. Herein we focus on the effects of the building block size, both at molecular (i.e., MW and DP) and colloidal scales, on the cohesion of biopolymeric constructs. We discuss mostly films and filaments, as their tight networks minimize the effect of pore on the transfer of cohesion arising from the building block size.

##### 6.2.1.1 Effect of the Molecular Weight of Building Blocks in Dissolved Systems

**Figure 30:**
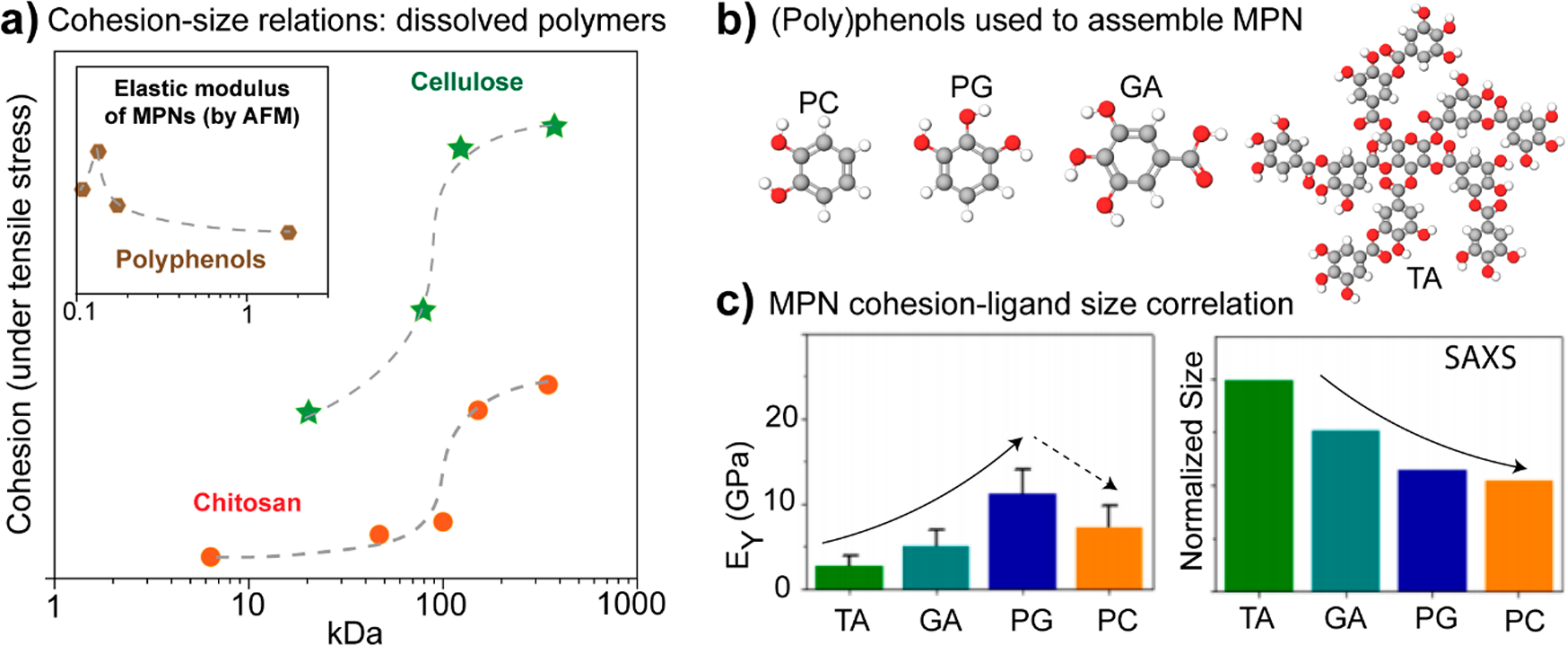
Overall effect of the building block size, while dissolved, on the cohesion of biopolymeric constructs. (a) Approximation, based on literature data discussed in this section, of the relation between size and cohesion for three biopolymers, namely, tannins, chitosan, and cellulose. (b) Phenolic compounds used to assemble metal phenolic networks, and (c) associated cohesion of the coordinated assembly. Adapted with permission from ref (638). Copyright 2019 American Chemical Society.

At a molecular length scale, there is an overall positive effect of the building block size, that is, MW and DP, on the cohesion of monocomponent cellulosic construct (Figure 30a). However, the cohesion of materials formed from dissolved polymers is highly connected to the pathway used for their regeneration, and therefore in some instances lower MW cellulose leads to stronger constructs.^521,815−818^ Additionally, at such length scales, the effects of polymeric crystal arrangement and associated crystalline indices become more relevant.^816,818^ The effect of MW of the cellulosic precursor on the cohesion of the regenerated fiber, when formed using identical processing steps, has been discussed.^817^ Cellulose pulps with MW from 125 to 363 kDa were dissolved in 1-butyl-3-methylimidazolium chloride (BMIMCl) and regenerated by dry-jet spinning. Among other processing conditions affecting the cohesion of the spun fibers (e.g., pH of the solution and temperature of the regeneration), the MW of the precursor showed a clear positive relationship with the tensile strength of the fiber. Fibers formed from pulp with MW = 125 kDa were 10% weaker (tensile strength ca. 1020 MPa) than the ones formed from pulps with MW = 363 kDa (tensile strength ca. 1150 MPa). Nonetheless, the regenerated fibers showed good mechanical performance, comparable to the highly aligned CNF-based multiscale fibers.^480^

Chitosan macromolecules with high MW and DP led to strong materials (Figure 30a).^819−823^ The process for consolidation, again, had a great influence on the cohesion of the final material; it is apparent that a universal scaling relation exist for the cohesion as a function of the polymer MW. In monolithic chitosan materials, that is, films, cohesion scales positively with MW. Whereas chitosan with MW ranging from 6.5 to 48 kDa resulted in materials with tensile strengths from 15 to 22 MPa,^824^ higher MW in the range of 150 to 350 kDa led to materials with tensile strengths of 55–62 MPa.^823^ Similar results have been reported in other studies.^822,825^ For instance, an almost linear cohesion scaling was found as a function of chitosan MW, spanning from 100 to 200 kDa for a tensile strength from 25 to 40 MPa.^822^ The positive scaling with MW arises from the intrachain interactions in polysaccharides, stronger and more stable than interchain and stacking interactions, due mostly to the longer H··acceptor/donor distances^166^ where nondirectional interactions tend to prevail.^826^

For alginates, the MW of the polymer plays an overall less determining role on the mechanical cohesion compared to the ratio of mannuronic (M) to guluronic (G) acid.^827,828^ Strong materials from alginates are usually assembled from complexation with multivalent cations, typically Ca^2+^, with the G blocks along the polymeric chain forming a cohesive egg-box structure. Although ionic complexes are favored with the G blocks, there is no clear correlation between G content and mechanical properties; instead, other factors such as the distribution and arrangement of the blocks may influence the mechanical cohesion of the molecular construct.^828^

Natural polyphenolics, such as lignin and tannin, add to the examples of versatile biopolymeric building blocks for materials.^88,735,783^ At the molecular length scale, one cannot trace a clear correlation between the size of these building blocks (i.e., MW) and the cohesion of their constructs, given the fact that most of the available lignins are heterogeneous as far as chemical structure and, particularly, as a function of the combined isolation process and biomass source. Although partially correlated, the density of certain groups or bonds such as phenolic OH or β-O-4, may easily overlap with the cohesive effects arising from the MW. For instance, when utilized in the preparation of lignin-based polyurethanes (PUs), Kraft lignin with high MW (43 000–66 000 g/mol) led to composite materials with higher tensile strength (43 MPa) compared to those of lower MW (1322–3790 g/mol), 35 MPa.^829^ In this latter case, the extremely high MW improved the materials stiffness because of the presence of nanosized lignin structures, anchoring the secondary polyol matrix (e.g., PEG), which was flexible. On the other hand, at smaller length scales (from 3600 to 600 g/mol), the size of the building block had a negative relationship with the cohesion of the lignin construct.^830^ Interestingly, at higher lignin fractions (at 40 wt %), the contribution of MW to the cohesion of the lignin was remarkable. The stiffness increased from 3.92 to over 140 MPa, when the MW of the lignin was reduced from 3600 to 600 g/mol, and the tensile strength from 4.4 to over 30 MPa. Such remarkable results were attributed to the higher density of hydroxyl groups that interact with isocyanate; a higher solubility and higher reactivity of the 600 g/mol lignin led to PU materials that were more homogeneous and with less defects.^830^

As discussed earlier (Section 5.3.2.1), tannin molecules form cohesive networks when coordinated with multivalent metal ions, especially Fe^3+^. The effect of the polyphenolic ligand size on the cohesion of the metal-phenolic networks has been studied.^638^ Well-defined molecular structures, that is, TA, gallic acid (GA), pyrogallol (PG) and pyrocatechol (PC) (Figure 30b), were used to assess the mechanical cohesion of MPN complexes by AFM force measurements. Overall, the smaller phenolic molecules led to higher MPN mechanical performance (Figure 30c). Whereas in TA-based MPN assembling was mostly driven by metal coordination, smaller building blocks (ligands) such as gallic acid (GA) may induce other interactions, for example, π–π, that may contribute to the cohesion of the formed MPN. With smaller ligands, one can expect more uniform and long-range order coordinated structure, owing to their molecular geometry that enhances orientation and promote tighter packing.^638^

The effect of the building block size remarkably affects the cohesion of protein assemblies. Silk proteins are classical examples of high-performing building blocks, recently used to synthesize materials with very precise control over their assembling structures and therefore over their properties.^104,113,831^ The cohesion of spider silk arises from its molecular organization, antiparallel β sheets and crystal sizes (2–4 nm), that are naturally optimized to induce strong hydrogen bonding and dissipative stick–slip deformation. Studies have demonstrated that for crystals with sizes over a critical length (>4 nm), the mechanism of energy dissipation changes from a favorable shear mode to an unfavorable bending mode. Therefore, in order to maximize the cohesion in artificial silk constructs, one should consider the synthesis of building blocks below such threshold.^113^

##### 6.2.1.2 Effect of the Biocolloid Nano- and Microscale Dimensions

**Figure 31:**
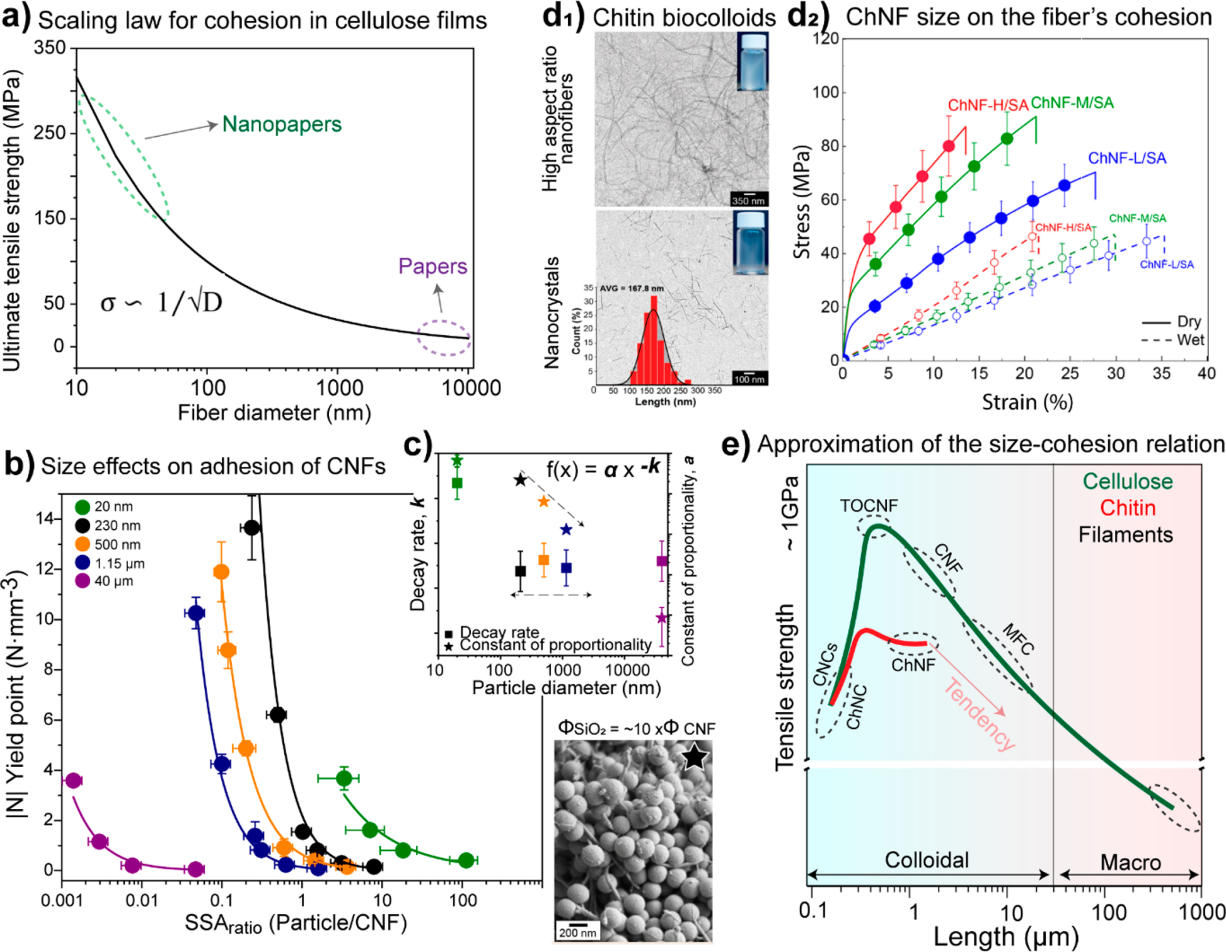
Cohesion transfer in biopolymeric assemblies as a function of the size of the building blocks. (a) Scaling law that determines the tensile strength of cellulose films and papers based on the diameter of the primary building block. Adapted with permission from ref (148). Copyright 2015 The authors. (b) Adhesion capacity of cellulose nanofibrils is linked to the size of the “bound” elements, where (c) cohesion arises universally across a wide range of particle sizes–optimized when the particle size is near 10-fold the diameter of the CNF (SEM inset). Adapted with permission from ref (479). Copyright 2020 The Authors. (d1) Chitin nanofibers are obtained in different lengths, depending on the processing conditions, and used to assemble filaments (via complexation with alginate upon dry spinning). (d2) Cohesion of the microfilament is related to the size of the chitin biocolloids. Adapted with permission from ref (838). Copyright 2019 American Chemical Society. (e) Qualitative diagram showing, as an approximation, the size-cohesion relationship for microfibers or filaments formed from cellulose or chitin nanofibrils of given aspect ratio.

The cohesion of nanocellulosic macro-sized materials scales according to the size of their elementary building blocks, following σ ≈ 1/ √ D where σ is the ultimate tensile strength, and *D* the diameter of the fiber/nanofiber (Figure 31a).^148^ This scaling law describes the mechanical cohesion of cellulosic assemblies (i.e., paper and nanopaper) formed from fibers with mean diameter, from 11 nm to 27 μm. The effect of the building block size is remarkable, with the ultimate tensile strength increasing more than 40 times from ∼7 MPa for regular paper compared to the ∼280 MPa observed in nanopapers. The density and nature of the interfiber interactions play key roles in such performance, as underpinned by the stick–slip motion under tension resulting from the dynamics of interactions between fibers. By decreasing the building block size, the density of such interactions increases exponentially, leading to much higher fracture energy, as required to break the material. Additionally, cellulosic materials display anomalous behavior as far as the toughness-strength relationship, which typically present a negative correlation. In cellulosic constructs, toughness and strength are positively correlated with scaling down the building block size, both remarkably increasing at the nanoscale. Toughness of the materials increases nearly 130 times, from 0.13 to 11.7 MJ/m^3^, when the fibers diameter is reduced from 27 μm to 11 nm.^148^

Within the nanoscale, the size-strength relationship is also affected by the processing conditions; for instance, when shear-induced alignment takes place. Cellulosic macrofibers can mirror the mechanical performance of nanocelluloses by controlling their multiscale assembly into an aligned, near defect-free architectures.^480^ Macrofibers (diameter of ca. 10 μm) with tensile strength over 1.1 GPa (ca. 1.6 GPa if cross-linked with 1,2,3,4-butanetetracarboxylic acid, BTCA) and Young’s modulus of ∼70 GPa were obtained. The length of the nanocelluloses had a remarkable effect on their cohesion, longer fibrils favored the alignment, leading to an overall tighter packing and better cohesion transfer from the single elements to the macrostructure. Fibrils 590 nm long resulted in assemblies with 1.1 GPa tensile strength; those of 390 nm in length led to macrofibers that were remarkably weaker (ca. 700 MPa),^480^ yet still relatively strong compared to other nanocellulose assemblies.^17^

In cellulosic constructs, there is a clear gain as far as mechanical performance when reducing the size of the building blocks, from the micro to the nanoscale.^136,148,832,833^ The higher crystallinity of the smallest elements would hint at possible gains by reducing the size of the building blocks. However, in practice, within the nanoscale, the higher aspect ratio found in longer fibrils favor higher mechanical performance. For instance, CNCs (ca. 5 nm in width and 150 nm in length) form materials that are weaker than the ones formed from relatively larger colloids, such as CNFs. The tensile strength of CNC films typically falls below 100 MPa.^17,834^ Stronger materials are formed if functionalized,^835^ composited,^836^ or aligned.^837^ The main difference between the strength of CNC and CNF assemblies relates to the ability of CNFs to create entangled architectures, which is not possible with CNCs given their less flexible nature.

The flexibility of CNFs was shown to play a fundamental role in enabling high cohesion in multicomponent constructs.^88,479,735^ CNF’s high cohesion is transferred to their composites by a series of self-and interfacial cohesive interactions. In the case of particulate composites, the size of the primary building blocks changes the interactive CNF-particle interface, thus leading to the formation of a variety of topologies that favor either CNF-particle or CNF-CNF interactions, depending on the dimensional relationships (Figure 31b,c). Whereas particles with diameter near that of CNF tend to form disconnected, weak networks, where cohesion arises mostly from interparticle interactions, with microscale particles (over 10 μm) CNFs form sheets that bring cohesion to the system via multiple interfibril interactions. When the particles are near 10–20 times the diameter of the CNF, the resulting network is intermixed with well-balanced CNF–CNF and CNF–particle interactions. For instance, cohesion was demonstrated to scale with the dimension of the assembled interface, following a power law with constant decay rate, *k* = −1.4, for hydrophilic particles ranging from 230 nm to 40 μm in diameter^479^ (Figure 31c). The effect of fibril concentration on the cohesion scaling varied depending on the CNF–particle interface, increasing more sharply, from low to high concentrations, for hydrophobic particles and more linearly for particles with a strong affinity with CNFs.^479^

Cohesion scales for chitin biocolloids similarly to what has been observed for cellulosic counterparts. There is a clear gain in mechanical performance arising from a decrease in the dimensions of ChNF, from the micro (or submicron) to the nanoscale. This has been recently demonstrated by comparing the tensile strength of films made from α-chitin (from insect cuticles) and assembled from partially defibrillated (bundles) or single ChNF.^727^ Partial deacetylation led to ChNF bundles, with a log-normal diameter distribution (median at 19 ± 18 nm) but with values as high as 100 nm with several micrometers in length. Complete protonation led to single ChNF, shorter in length (submicron range), but with higher aspect ratio associated with their smaller diameter of 6 ± 2 nm. In contrast, materials formed from the smaller fibrils resulted in ∼200–250 MPa of tensile strength; their bigger counterparts formed materials relatively weaker, with a tensile strength of ∼100 MPa. Larger fibrils led to overall higher defect density in films, due to their more extensive aggregation, leading to a decrease in the fibril–fibril friction, slippage and reduced fibrillar entanglement, all of which would otherwise enhance the energy dissipation mechanisms in fibrillar matrices. Beyond size, two main factors affected the cohesion of the nanochitin films and associated scaling, as a function of building block size. The larger fibrils contain small fractions of protein that decreased the chitin–chitin interactions. Additionally, intrinsically, the chitin backbone is potentially more rigid and stronger, than the proteinaceous matrix. Recently, the aspect ratio of chitin nanofibrils was shown to greatly influence the mechanical performance of chitin constructs produced by dry spinning^838^ (Figure 31d). Nanochitins with aspect ratio of 15, 25 and over 60 (lateral sizes of ca. 11 to 40 nm and lengths from ca. 150 nm to up to 4 μm), were used to assemble composite fibers by interfacial complexation with alginates via ionic interactions. Overall, chitin colloids with higher aspect ratio led to stiffer, and more brittle macrofibers (diameter ca. 10–40 μm). Additionally, the (ionically) complexed biopolymeric construct displayed higher wet strength (up to 40 MPa), which is not usually observed for materials built from unmodified polysaccharides. Therein, the lower aspect ratio nanochitin, which were also smaller, favored higher interfacial interactions with alginate, improving the toughness of the formed microfibers by enhancement of the stress transfer mechanism, across the interfaces. However, they showed a lower degree of alignment in the microfiber, which resulted in lower ultimate tensile strength. The opposite was observed for the nanochitins of highest aspect ratio, which were larger in both lateral and length dimensions. This corroborates the reported observation for cellulosic colloids,^480^ indicating a universal law for polysaccharide-based colloids. On the basis of the literature data concerning the mechanical performance of materials built from high aspect ratio chitin and cellulose biocolloids, a general size-cohesion relationship can be drawn, as introduced in Figure 31e.

Polyphenolic nanoparticles have been highlighted as better options for the preparation of strong materials when compared to powders that are irregular in shape.^88,839^ The latter is the typical morphology following extraction from biomass using conventional processes. Recently, colloidal lignin particles (LP), diameter of ∼100 nm, were used to synthesize CNF–lignin films with tensile strength over 160 MPa, Young’s modulus of 5.5 GPa and 15.5 MJ m^–3^ in toughness.^88^ Films with the same lignin fraction (10 wt %), but using irregular micron-sized powder, presented considerably lower mechanical strength (tensile strength of 115 MPa, Young’s modulus of 4.2 GPa and 12.6 MJ/m^3^ in toughness). As discussed earlier, at this length scale, cohesion arises from optimized interparticle interactions, which in this case concerns hydrogen bonding and vdW interactions. Irregularly shaped and larger particles lead to a higher defect density across the materials and hinder the components from efficient interaction at the interfaces, thus reducing the efficiency of energy transfer under mechanical solicitation. A similar trend has been observed for lignin bioadhesives, where well-defined colloidal lignin (shear strength >1 MPa) performed better than the irregular counterparts (shear strength <0.1 MPa).^839^

#### 6.2.2 Effect of Surface Chemistry

**Figure 32:**
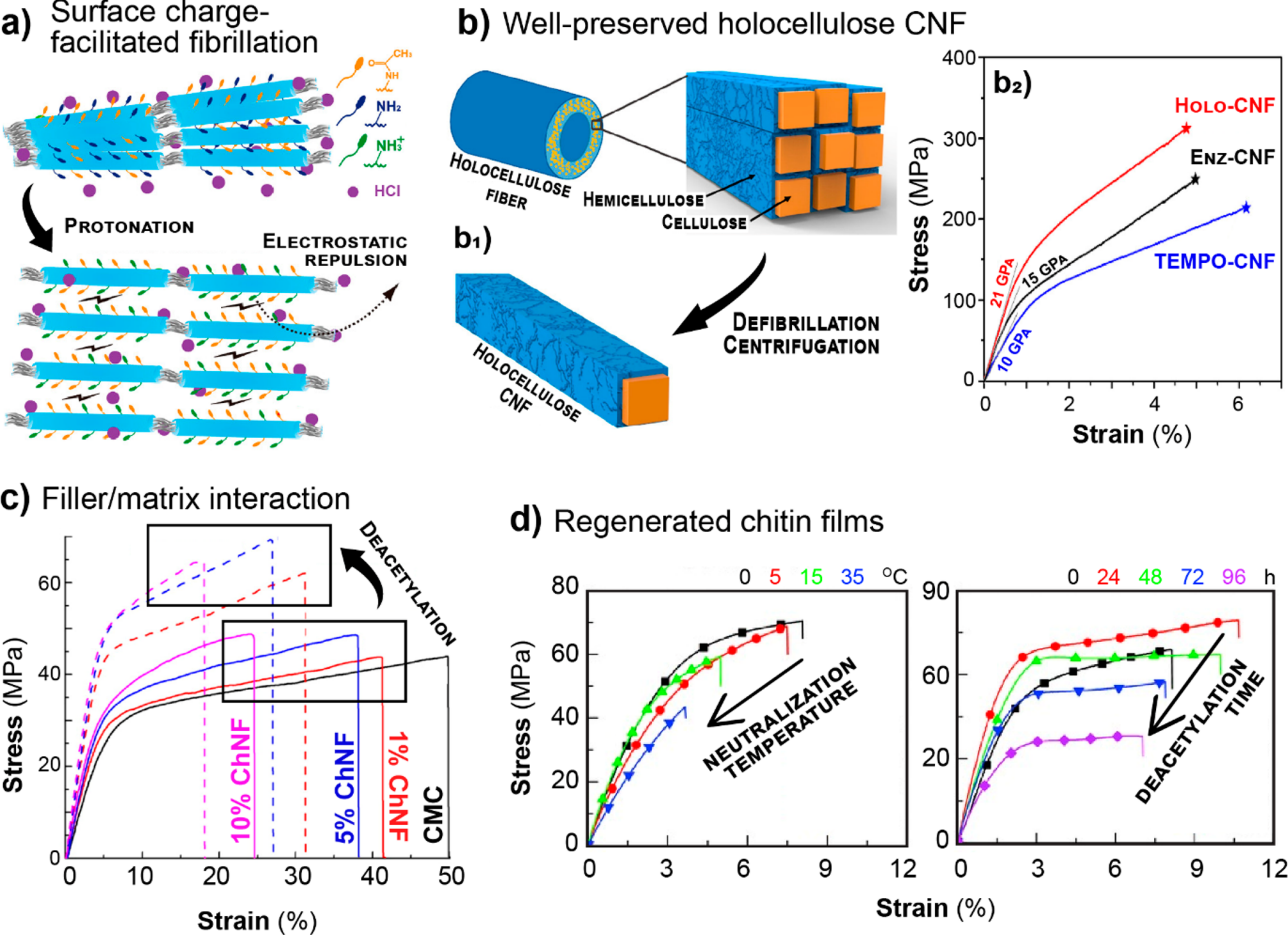
Effect of building block surface chemistry on the mechanical properties of hierarchically assembled materials. (a) Scheme (not to scale) of the fibrillation of partially deacetylated chitin, showing tightly (top) and loosely (bottom) bound chitin clusters. Adapted with permission from ref (360). Copyright 2020 American Chemical Society. (b) Scheme (b_1_, not to scale) of the isolation of well-preserved (unmodified holocellulose) CNF and representative stress–strain profiles (b_2_) of respective nanopapers or films compared to those of enzymatically pretreated and TO–CNF. Adapted with permission from ref (212). Copyright 2020 American Chemical Society. (c) Typical stress–strain profiles of CMC-based composite films (0–10 wt %) (solid curves; ζ-potential: ca. + 15 mV) or partially deacetylated (dashed curves; ζ-potential: ca. + 31 mV) ChNF. Adapted with permission from ref (841). Copyright 2016 American Chemical Society. (d) Tensile stress–strain profiles of films made from regenerated chitin submitted to different neutralization temperatures and deacetylation times. Adapted with permission from ref (842). Copyright 2017 John Wiley and Sons. Note: Further permissions related to the material utilized in (b) (pubs.acs.org/doi/10.1021/acsnano.9b07659) should be directed to the American Chemical Society.

The efficiency of intermolecular or interparticle cohesion transfer and therefore the robustness of ensuing constructs produced with biocolloids strongly depends on their surface chemical features. This applies to biopolymers, such as proteins, whose surface can be contoured. Building block size and surface chemistry go hand in hand. Mechanical fibrillation of chitin and cellulose, for instance, is expected to lead to thinner fibrils when they have a higher surface charge density due to the larger tendency to swell (Figure 32a), but some chemical modifications, such as periodate oxidation, may increase the chances of chain scission. Similarly, high methoxyl pectin is more prone to base-catalyzed depolymerization via β-elimination, as such chain-splitting reaction happens at glycosidic bonds next to esterified galacturonic acids.^840^ Similarly applies to the hydrodynamic radius of biopolymer coils in solution and the effective diameter of colloidal particles in suspension. Still, the weighed contributions of size and chemical structure, their interplay affecting both assembly strength and transfer of cohesion, is often dominated by the former. This section addresses the case when surface chemistry is a prime factor.

Compared to partially deacetylated chitin, unmodified chitin requires more energy for disintegration, but the native fibrillar colloid is often less degraded and stronger than the surface-modified ones, chitosan included, owing to the reduced crystallinity and MW upon derivatization or regeneration. Disintegration at mild conditions results in well-preserved ChNFs, featuring a low degree of deacetylation (DD), residual protein content, as well as high degree of crystallinity and MW, which altogether translate into high-strength materials.^727^ This is particularly relevant for ChNFs isolated from insect cuticles, as harsher treatments are needed compared to crustacean-derived chitin, which is mixed with calcium carbonate. Similar considerations apply to nanocelluloses: holocellulose CNF is extensively devoid of lignin but with cellulose and hemicellulose content similar to that in the native state, as well as MW and structuring. Holocellulose-CNFs lead to materials that perform better mechanically than those made from, for example, TEMPO-oxidized or enzymatically pretreated CNFs (Figure 32b_1_). Recyclability is another advantage in these materials.^843^ The mechanical properties of nanopapers based on well-preserved holocellulose CNFs, shown in Figure 32b_2_, with a Young’s modulus of 21 GPa, mirrors the more effective stress transfer compared to samples produced from typical CNFs. This feature originates from the lower defect density introduced by the milder mechanical treatment as well as to the improved interfibril bonding, given the presence of hemicelluloses evenly distributed on the CNF surfaces.^212^ Similar conclusions can be drawn for holocellulose papers compared to Kraft paper, the former being much stronger and stiffer given the stronger interfiber adhesion and thus the intrinsic mechanical properties of the native fibers, in the presence of hemicelluloses.^844^

The type and content of functional groups as well as their stereoregularity are known to play important roles in given properties, typical of hierarchical biopolymer constructs. One of the most exploited systems in this regard is chitin, which preserves a cationic character depending on the pH and ionic strength. In fact, although they have similar chemical natures, chitin (DD < 50%) and chitosan (DD > 50%) behave remarkably differently as far as the solubility, the polyelectrolyte-like colloidal behavior and the mechanical properties of the consolidated solid materials they produce, all of which depend on the DD. When used as an additive in papermaking, chitosan has been demonstrated to increase the tensile strength in both machine and cross directions, under a DD-dependent fashion, given the greater efficiency of higher-DD chitosan to render the cellulose network more cohesive, for example, by filling voids and providing extra sites for interactions.^845^ Depending on the processing conditions—markedly pH, ionic strength, and composition—electrostatic attraction among positively charged chitosan (charge density higher for higher DD) and anionic cellulosic fibers contribute to the network cohesion. This electrostatic driven mechanism is in line with that reported for ChNFs when acting as reinforcement agent in CMC films, in which the mechanical properties are improved for partially deacetylated ChNFs compared to the less deacetylated counterpart (Figure 32c). Such an outcome arises from the stronger association with anionic CMC at a pH intermediate compared to the p*K*_a_ of the complexing species.^841^ A representative effect of surface chemistry on films of regenerated and deacetylated chitin is depicted in Figure 32d. In the case of chitosan-based films, the deacetylation effect on the mechanical strength is still in debate: while higher DD leads to increased crystallinity and would therefore increase tensile strength at the expense of extensibility, the opposite is to be expected from the higher swelling enabled by higher DD.^846^ Also, because of the Coulombic arguments stated above, same-charge entities might impair packing, which would produce a more limited strength in the assembly. As already introduced (see Section 5.3.1), charge screening by electrolytes is effective in overcoming the repulsive barrier and triggers attractive colloidal interactions. In another approach, some groups induced both charges in the so-called zwitterionic nanochitins, wearing both amine and carboxylate groups, arising from partial surface deacetylation and TEMPO-mediated oxidation,^361,847^ either in this order or in the inverse sequence.^848^

Similar to the case of chitin, pectin naturally occurs as a polysaccharide whose surface chemistry, degree of methyl esterification (DM), specifically, has been extensively modulated to impart different functionality and performance. In an analogy to DD in chitin, DM affects most of the properties of pectin-based materials, all the way from gels to their solid-state counterparts. While high methoxyl pectin (DM > 50%) gels at acidic pH via hydrophobic interactions and hydrogen bonding, low methoxyl pectin (DM < 50%) gels over a wider pH range via the interaction between the carboxyl groups of pectin and added multivalent cations. The DM also affects the properties of dried films, which are stronger and tougher at high methoxyl pectin compared to those produced from low-methoxyl analogues, owing to the decreased cohesive strength of the latter, in a range of hydration degree and divalent calcium concentrations.^849^

## 7 Formation of Multiscale Architectures

In nature, multiscale architectures (spanning the molecular and macroscale sizes) enable a combination of properties that cannot be reached in monolithic or architecture-free systems. Multiscale architectures, ranging from the arrangement of macromolecules to macropores, allow tailorable density, strength, toughness, optical properties and directional response to strains. The combination of these properties can be enhanced simultaneously by given architectural considerations. For instance, an increase performance in both strength and toughness can be achieved, which is not typical in engineered materials. Using biopolymeric building blocks, including biocolloids, bottom-up nanomanufacturing enables a high versatility and scalability in material synthesis. We put forward the main approaches used to generate ordered nanostructures from biopolymers, including porous and consolidated constructs. The properties considered here, specifically developing from multiscale designs, are evaluated relative to those that are produced from random assemblies.

### 7.1.1 Nematic Order

Nematically ordered biopolymeric materials bring critical properties, such as controlled ionic flow,^850^ improved strength,^480^ improved toughness of porous structures,^851^ directional insulation,^851,852^ gas permeability control,^853^ and control over optical properties such as opacity,^854^ and light transmittance in filaments.^855^ Formation of materials with nematic ordering, that is, with aligned anisometric building blocks, enables optimization of the interactions, for instance to produce distinctively strong structures.

**Figure 33:**
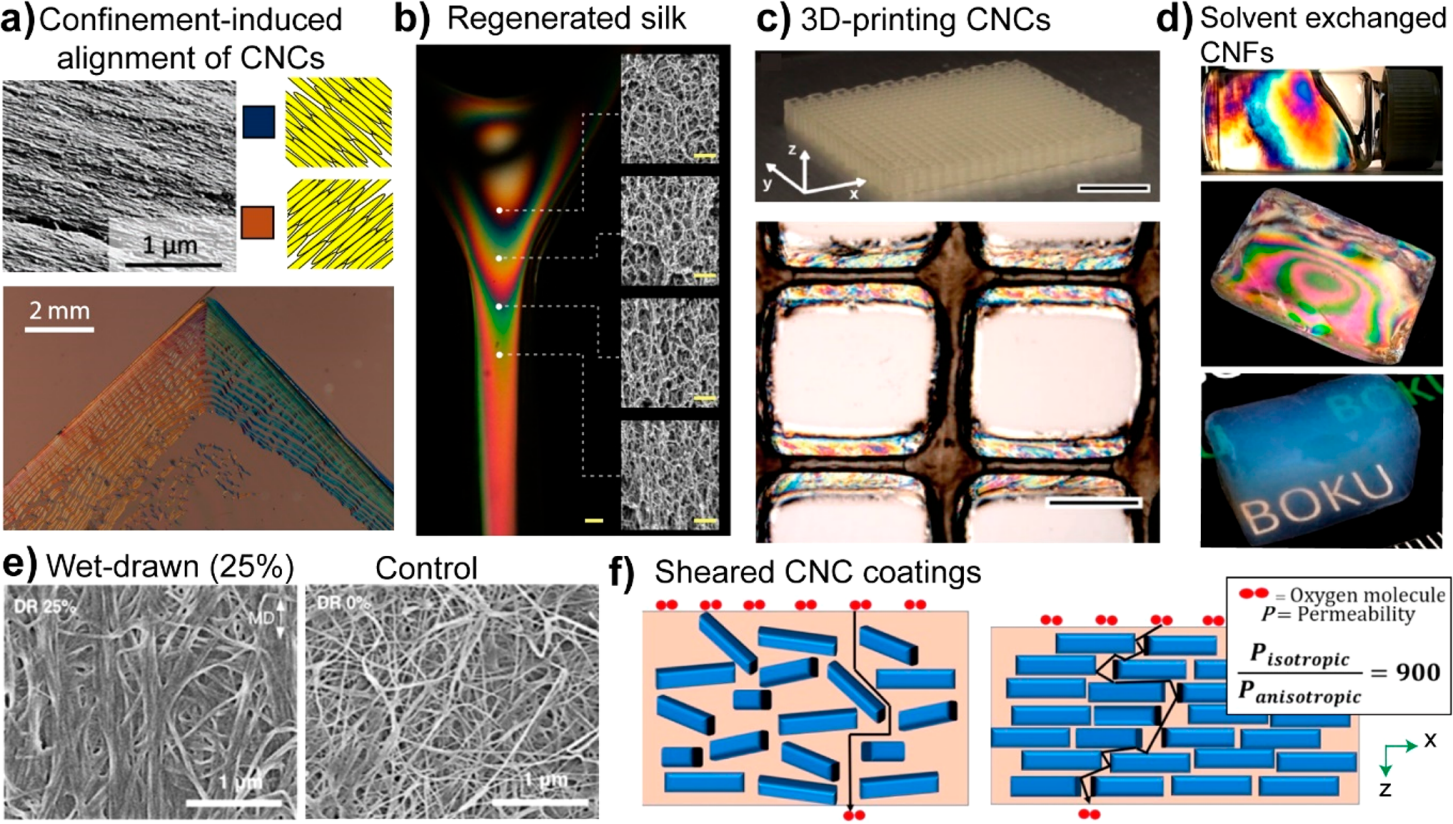
Nematic order (aligned nanofibrils) obtained in various consolidated materials. (a) Multiscale alignment of CNC within confined bonds as highlighted by a scanning electron micrograph (top-left), and cross-polarizer micrographs obtained in the presence of a retardation plate. Adapted with permission from ref (492). Copyright 2020 John Wiley and Sons. (b) Nanofibers of silk observed between cross polarizers. Reprinted with permission from ref (871). Copyright 2017 Nature Publishing Group. (c) Nematically aligned CNC as a result of extrusion observed between polarizers. Adapted with permission from ref (484). Copyright 2017 John Wiley and Sons. (d) Aerogel obtained from periodate-oxidized nanocellulose fibers observed between polarizers. (top) Precursor gel (middle and bottom), obtained aerogel with and without cross-polarizers. Adapted with permission from ref (323). Copyright 2017 American Chemical Society. (e) Alignment of BNC as a function of wet stretching. Adapted with permission from ref (860). Copyright 2017 American Chemical Society. (f) Schematic representation of the impact of shear alignment during CNC coatings on the relative orientation and resulting gas permeability. Adapted with permission from ref (853). Copyright 2019 American Chemical Society.

In filaments made of nanocelluloses, the strength increases linearly for Herman orientation parameters ranging from ∼0.52 to 0.62. In this range, toughness follows a logarithmic increase with alignment, while the elastic modulus increases following an exponential relationship. This suggests that a higher alignment initially result in strictly stronger assemblies, while at the upper boundaries of alignment, the materials become more brittle, with a higher modulus; thereafter the toughness is reduced proportionally.^481^ Similar results were obtained for CNC/CMC composites,^856^ although not for single-components, suggesting the importance of a “soft” phase.^722,837^ For nematically ordered, nanofibrillar constructs, an improvement of up to two folds in both strength and toughness (normalized by density) was achieved when comparing nematically oriented aerogels against their isotropically oriented counterparts.^851,857,858^ Regarding gas permeability in CNC films, for both CO_2_ and O_2_, it decreased by more than 10-fold when the orientation parameter increased from 0.28 to 0.85 (1 = perfect alignment) (Figure 33f).^853^ Lastly, directional thermal conductivity was shown to be doubled when under better alignment (orientation parameters increasing from 0.45 to 0.75).^859^ A linear scaling was observed for wet-drawn and aligned bacterial nanocellulose films.^860^

The building blocks were aligned by a combination of (1) physicochemical means where, phase transitions or the solvent properties were altered by mean of an external influence, such as solvent exchange or acidification, where a rapid unidirectional compression and gelation occurred; (2) shearing of the gelled dispersion, where unidirectional traction aligns the packed gels; (3) hydrodynamics of the dispersions, where shear control and drying phenomena are exploited, or (4) external stimuli such as electric or magnetic fields. The effect of (1) has been used to achieve alignment of cellulose nanofibrils by using antisolvents, such as acid or acetone. For instance, addition of acid to dispersions of TO–CNFs or periodate oxidized CNFs resulted in the formation of a nematically ordered hydrogel, which can be conserved into an aerogel structure by solvent exchanging the dispersion and supercritical drying. The porous nanofibrous architecture showed an improvement in resistance to compression^323,851^ as well as pore harmonization under compression, where the pore homogeneously and unidirectionally “shrank” instead of collapsing upon compression (Figure 33d).^323^ This latter pore compression toward nanopores is in fact associated with an increased capillary condensation as measured by nitrogen adsorption isotherms. Controlled aggregation of CNC dispersion was also shown to induce a transient nematic orientation, at high solid contents and saline conditions.^622^ In another solvent-induced ordering approach, a two-step procedure, via flow focusing, produced some of the highest strength for single component fibers.^480^ Using exclusively shear (2), as illustrated for instance in 3D printing efforts (Figure 33c),^484,861^ lower mechanical performance was observed when compared to shear combined with solvent exchange. Shear-orientation is reported in several studies dealing with extrusion and solvent exchange annealing of fibers^481,685^ as well as in dry spinning.^862,863^ Compositing nanofibrils with silk protein resulted in tailorable mechanical properties.^487^ Beyond mechanical performance, nematic ordering enables an anisotropic response to humidity, resulting in predictable folding of complex patterns.^864^ In the case of CNC films, an anisotropic film swelling, above 40-fold, was observed for films bearing highly aligned CNCs (orientation parameter of 0.8).^865^ In contrast, no anisotropy was observed for chiral-nematic systems (orientation parameter of 0.04). Films were obtained using sheared gels to control the optical properties of elastomeric composites.^483,854,866^ In other studies, stretching of a wet gel resulted in an improved alignment. For instance, compared to unaligned filaments obtained from bacterial nanocellulose (BNC), those obtained upon wet-drawing (30%) resulted in improved fibrils alignment, which increased the strength by up to 8-fold, achieving 1 GPa.^867^ This strategy was also employed to form direction-dependent thermal insulators (Figure 33e).^860^ In contrast, stretching wet gels of cellulose or chitin nanofibers enabled a somewhat limited compared to the previous results.^688,856,868^ Alternatively, mechanical constraints as imposed by the substrate or clamping improved the long-range order of the nanofibrils or regenerated cellulose.^755,762,869,870^ In strategy (3), more recently reported, the hydrodynamics of consolidation were exploited by controlled the interactions with a given substrate, upon assembly. For instance, infiltration onto and between substrates control the regeneration of silk fibroin building blocks (Figure 33b),^871^ or the long-range order of CNCs (Figure 33a).^491,492,767^ In the latter case, this was associated with multiscale ordering, where thin periodically distributed stripes were formed under confinement (Figure 33a). This resulted in adhesion strength between the confining substrates, up to 7 MPa, and an interesting adhesion anisotropy, where applying forces out of the plane of the bond easily fractured the interface.^492^ For 2D materials, that is, ultrathin films, doctor blading exploited localized convection to generate order at the single colloid level,^478^ which introduced as interesting opportunity to create model films, for example, to study the effect of alignment on the optical and mechanical poperties.^520^ For instance, friction force was observed to be 30% higher in the cross direction compared to that along the nematic director for films with an orientation parameter of 0.58. Regarding external stimuli to induce a nematic order (Strategy 4), magnetic and electric fields control the collective behavior of biopolymers and biocolloids, provided they possess a sufficiently high electric-dipole moment. The latter is particularly high for CNCs^490^ a reason why this approach is most commonly applied to CNC assemblies. However, collagen was one of the first nanofibrous element to be studied in this context, where a significant increase in birefringence was observed by assembly in the presence of a magnet.^872^ Other approaches used magnetic nanoparticles to couple the alignment of collagen,^873^ CNCs (in a PLA matrix),^874^ or amyloid fibrils^875^ under a magnetic field. The effect of electric fields on CNC assemblies has been thoroughly studied in dispersions.^876^ Complete unwinding of the chiral-nematic helix occurred at 2.2 kV/cm. Interestingly, the aligned assembly could be relaxed and would anneal into better-ordered, nearly fully cholesteric, assemblies.^601^ Noteworthy, the morphology of tactoids prior to annealing can be substantially altered by electric fields.^877^ In a similar manner, magnetic fields have been applied to control the orientation of CNC assemblies,^602^ leading to an increased alignment under relatively weak fields (≤1.2 T), albeit a biphasic initial concentration is required.^878^

It should be noted that upon unidirectional drying, as conventionally applied to cast-drying, nanofibers align in the plane perpendicular to the drying front. Although not completely nematically ordered, the system is not isotropically ordered and this can be maintained upon reswelling of the dried material.^879^ Overall, alignment of nanofibrils and biopolymers has been extensively studied, in efforts to enhance properties, such as mechanical strength. Many of these approaches affect the collective behavior of biopolymeric assemblies, while being scalable and therefore one can foresee applications where synthetic systems may not compete.

### 7.1.2 Chiral-Nematic Order

**Figure 34:**
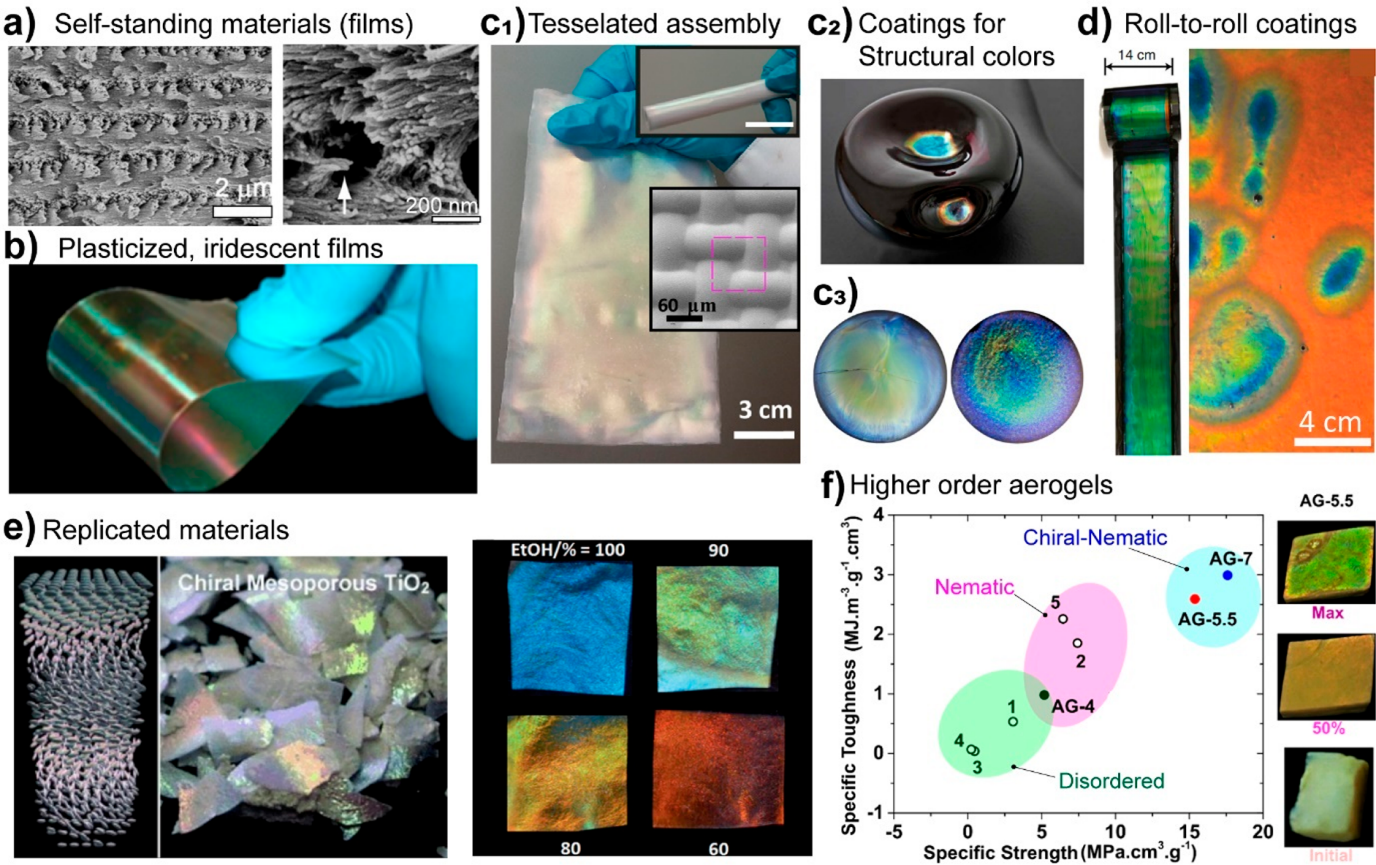
Chiral nematic structures obtained from cellulosic chiral-nematic phases. (a) Scanning electron microscopy images describing the rotation of the CNC mesogens along the chiral-nematic director. Adapted with permission from ref (910). Copyright 2012 Springer Nature. (b) An iridescent CNC film plasticized with PEG. Reprinted with permission from ref (893). Copyright 2017 John Wiley and Sons. (c1) A Nylon mesh coated with an iridescent CNC film showing improved flexibility (inset). Adapted with permission from ref (491). Copyright 2019 John Wiley and Sons. (c2,c3) Iridescent coatings on glass and PS surfaces. Adapted with permission from ref (477) and ref (579). Copyright 2019 Springer Nature and Copyright 2018 American Chemical Society. (d) Roll-to-roll coatings of HPC (left) and associated pressure response (handprint on the right). Adapted with permission from refs (904 and 905). Copyright 2018 Nature Publishing Group. (e) Templated titanium chiral nematic mesoporous structures (left) and cotemplated with a secondary phase (urea formaldehyde, right). The responsiveness to ethanol fraction in water is shown. Reprinted with permission from ref (899). Copyright 2012 John Wiley and Sons. (f) Impact on mechanical load (left) and pressure response (right) of chiral-nematic aerogels (photomechanical response). Adapted with permission from ref (482). Copyright 2019 The Royal Society of Chemistry. Note: Further permissions related to the material utilized in Figure 34c3 (pubs.acs.org/doi/10.1021/acs.biomac.8b00497) should be directed to the American Chemical Society.

Chiral-nematic liquids crystals, and associated materials with a cholesteric order, are abundant in nature as this architecture enables improved fracture toughness and selective light reflection (Figure 34f).^880^ Beyond top-down partial deconstruction of biomass, for example, where the cholesteric architecture typical of crustaceans is maintained,^881^ biobased materials with a chiral-nematic order are currently obtained principally via lyotropic liquid crystalline phase transitions.^882,883^ These architectures have been studied for several purposes; for instance, as precursors to form nematically organized materials,^854,884^ as is the case of certain dissolved biopolymers (e.g., HPC), to control the optical properties of the formed iridescent materials,^882^ as template to form inorganic or polymeric material bearing a chiral-nematic architecture,^885−887^ and for biomimetic fracture dissipation observed in strong and tough natural composites.^482,888^

Although liquid crystalline phase transitions of biocolloids were previously studied with fd-virus,^889^ recent efforts with chiral-nematic assemblies were shown for HPC and CNCs. This is associated with their commercial availability, combined with their well-documented behavior (Figure 34a).^572,890^ Furthermore, although their self-assembly is mostly limited to casting and, in certain cases, confined assembly, obtaining chiral-nematic assemblies from these building blocks is rather simple. The main reason for applying casting is associated with drying flux inhomogeneities and shear, which substantially disturb the assemblies, even in the presence of low forces. Minimal shear onto the gelled dispersion leads to alignment into a nematic order,^854,866^ while capillary confinement leads to the unwinding of the helices, that is, if the assembly kinetics is not proportionally slowed down. For instance, assembly within porous grids leads to nematic assemblies unless the assembly is performed at high humidity and low temperature (4 °C), while assembly in a glass capillary leads to chiral-nematic assemblies but with a better order onto the side showing slower assembly dynamics.^762,763^

The parameters that critically affect the development of chiral-nematic assemblies were already discussed, considering the effect of the mesogen morphology. Mainly three approaches have been shown to have a significant impact: (1) controlling the kinetics of the assembly using heat or extended equilibration processes; (2) applying additives to modulate the distances between the mesogens and (3) using external stimuli to enhance annealing. For (1), heat-induced evaporation leads to red-shifting of the reflection colors, which is associated with a stretching of the helices. This was used for patterning iridescence of chiral nematic films, shifting blue reflections to green or red color, locally.^891^ This effect is associated with increased disorder, the latter is highly reduced by annealing of tactoids through extended equilibration of the biphasic dispersions.^578−580^ The effect was shown to decrease the bandwidth of the cellulose-based reflectors. The use of additives to modulate chiral-nematic architectures from CNCs (2) is by far the more typically reported approach. Addition of a secondary phase, such as poly(vinyl alcohol) (PVA), poly(ethylene glycol) or poly(oligoethylene glycol methacrylate-*co*-hydroxyethyl methacrylate), red-shift the reflection, which can be used to tether a range of phenomena in CNC-based systems, for example, to respond to humidity or for controlled reflection of laser beams.^491,888,892−894^ Addition of up to 30% of PVA red-shifted reflections from ∼560 to 680 nm, accompanied by a significant increase in bandwidth, from ∼200 nm toward over 400 nm. Higher addition levels led to nearly complete disappearance of specific Bragg reflections. In parallel, addition of such polymers enhances the flexibility of the materials (Figure 34b). Addition of monovalent electrolytes induces similar effect, due to aggregation, although at small concentrations a blue shift is observed as a result of decreased electrostatic repulsion. Two external stimuli were shown to control annealing of tactoids, electrical and magnetic fields. However, only magnetic fields have been exploited to change the reflection properties in consolidated materials, which enabled complete control over the orientation of the cholesteric director and its homogeneity across the formed reflectors.^602^

The past decade has seen significant interest in the development of strong CNC-based films displaying selective iridescent reflections. These films generally presented a tensile strength between 10 and 50 MPa, while being relatively brittle, which is associated with their elastic moduli, in the GPa-range.^895^ Chiral-nematically ordered films from chitin were reported with tunable periodicity, although the mechanical properties of the constructs were not discussed.^592^ Interestingly, the pitch obtained was rather large and visible reflections were therefore not observed. Although it is reported that silk fibroin assembles into chiral-nematic order in nature,^896^ this is not reported for man-made materials. The films were also applied as coatings over a range of substrates including PS,^579^ nylon^491^ and glass (Figure 34c).^477,491^ They showed good adhesion on hydrophilic substrates but a reduced interaction when the contact angle tended toward 90°, although this could be compensated, at least to some extent, by using additives or compatibilizers.^491^ Templating of inorganic materials, using chiral-nematic biopolymeric assemblies, enabled one of the more advanced inorganic structures reported, using CNCs and HPC.^897^ This enabled carbon^898^ and inorganic materials such as titanium (Figure 34e),^899^ titania,^900^ alumina,^901^ silica,^887^ to be imbued with specific reflections, and thus to gain control on the periodicity of the mesoporous structures.

For the response to compression or shear forces, chiral-nematic 3D bulk materials have been prepared with mesoporous structures.^482,902,903^ These resulted in materials with pressure-responsiveness to photonic reflection, where a blue-shift of the reflections could be induced by compression.^482,885,903^ Interestingly, it was demonstrated that chiral-nematic structures formed more compression resilient architectures, therein aerogels, than nematic or isotropically ordered aerogels obtained from the same building blocks.^482^ The specific toughness and strength was improved by 60%, and 137%, respectively, when compared with the nematically ordered equivalent. While the fracture dissipation of cholesteric materials is an extremely appealing prospect, as used in natural architectures, no other examples are available exploiting biopolymeric assemblies to form bulk materials that resist out-of-plane fracture propagation, as induced by such cholesteric structures. The photomechanical response was also demonstrated for films of HPC, where the mechanical response was associated with the possibility to form compression sensors, for instance, to detect pressure distribution during stepping (Figure 34d).^904,905^ The reflection wavelength blue-shifted from ∼675 nm to 550 and 500 nm upon application of 1.4 and 3.1 kPa, respectively. This was accompanied by an increase in bandwidth, from ∼100 to 250 nm. Importantly, because the use of HPC involves a more mature manufacturing approach, roll-to-roll approaches enabled large scale implementation (producing meter-scaled sensors). Such technology was explored to control the opacity of films, albeit without the use of chiral-nematic structures.^906^

For the formation of photonic reflectors using biopolymeric constructs, an alternative to the chiral-nematic architectures has considered soft nanolithography, for example, to create reflective patterns, for instance using HPC.^907^ The technology was further combined with chiral-nematically oriented HPC to form shape memory, adaptable and mechano-responsive skin adhesives.^908^ Nano- and microtemplating of chiral-nematic films has also been proposed to couple a controlled orientation of the chiral-nematic director with secondary features imparted by the template.^491,909^ This enables convolution of reflection signals as imparted, for instance, by diffraction gratings in addition to helicoidal periodicity.^909^

### 7.1.3 Nanosize Networks

We next discuss the assembly of spherical particles, nanofibers, as well as dissolved biopolymers, into films and aerogels. We refer to nanonetworks with architectures that are microporous, in the pore size range <2 nm, mesoporous (pore size 2–50 nm) and macropores (pore size >50 nm), with length scales in the submicron range. Nanonetworks can display either low (particle lattices and films) or high (aerogels) specific surface area, depending on the pathway taken for the consolidation of the given biopolymeric precursor.

**Figure 35:**
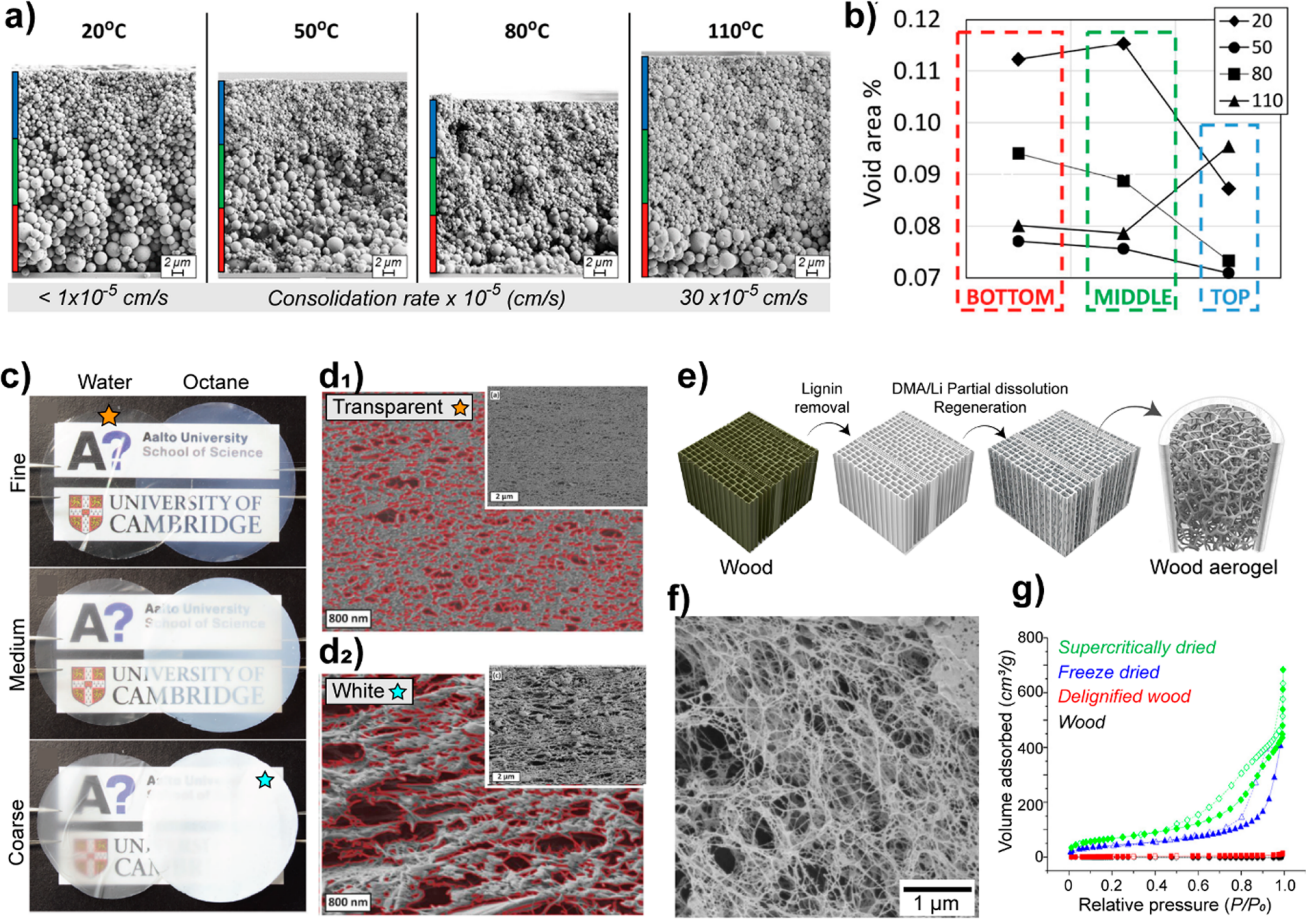
Effect of processing on the formation of nanonetworks of biopolymeric precursors. (a) Effect of the consolidation dynamics, controlled by the evaporation rate, on the final morphology of particulate films produced from lignin particles. (b) Distribution of the particle sizes, stratification, is a function of the drying temperature. Adapted with permission from ref (721). Copyright 2018 American Chemical Society. (c) Nanocelluloses of varying sizes, dried from water or octane, can form significantly different nanonetworks, (d1) tighter or (d2) more porous if dried from water or octane, respectively. Adapted with permission from ref (725). Copyright 2018 John Wiley and Sons. (e) Natural wood has been used to prepare hierarchically structure materials in which the (f) lumina of wood contain cellulose nanonetworks that are prepared by partially dissolution. (g) Such confined nanonetworks increase dramatically the surface area of the resulting wood structures. Adapted with permission from ref (934). Copyright 2020 American Chemical Society. Note: Further permissions related to the material utilized in (a,b) (pubs.acs.org/doi/10.1021/acs.langmuir.8b00650) and (e–g) (pubs.acs.org/doi/10.1021/acsnano.0c01888) should be directed to the American Chemical Society.

Controlling the consolidation kinetics of polydisperse systems offers a means to create random particle packing, with stratification of particle sizes across the normal direction. Particulate coatings from suspensions of polydisperse lignin particles, LP (from 50 to 2000 nm, with median at ca. 250 nm) have been prepared by evaporation induced self-assembly (EISA).^721^ Experimentally and numerically, the dynamics of LP packing and their stratification across the normal direction as a function of the consolidation rate were evaluated (Figure 35a).^721^ The final morphology of the nanonetwork correlated with the drying rate. At very slow drying rates (<1 × 10^–5^ cm/s), the LPs stratified with micron-sized particles sedimented at the bottom and particles <300 nm locating at the top layers of 50-μm thick coatings. Consolidation at faster rates (30 × 10^–5^ cm/s) led to a heterogeneity in particle size distribution across the film thickness, which is explained by a kinetic constraint. Packing, and therefore porosity, is modulated by the drying rate. Image analysis of the void area on the top, middle and bottom sections of the LP coatings showed that slower drying rates promoted tighter packing, while faster drying rates led to increased porosity, especially in the uppermost layers of the coating (Figure 35b).^721^ Additionally, given the advances on LP preparation,^451^ colloidal crystals (e.g., particle lattices) from biobased particles may become possible in the near future.

High-aspect ratio biocolloids, such as CNFs,^26^ ChNFs,^303^ and silk fibers,^113,310,911,912^ form extremely tight and entangled nanonetworks, in the form of films, or highly porous and low-density networks in aerogels. The consolidation route is the only factor separating such extremely diverse architectures, meaning that with the exact same initial precursor suspension one can obtain either very tight or loose architectures.^17,913,914^ A common aspect of tight nanonetworks is that processing occurs at room temperatures (20–25 °C) or higher, whereas loose, highly porous nanonetworks are commonly obtained by processing, at least one step, under temperature below 20–25 °C (e.g., freeze-drying).

CNF and ChNF films have shown remarkable mechanical strength, which related to the size of the building blocks and their ability to entangle in an interconnected, fully interlocked nanonetwork.^136,148,166,421,725^ More relevant to this section is the consolidation kinetics, which can be manipulated to yield fibrillar nanonetworks with given porosity.^725^ For instance, drying CNF suspensions from octane yields more porous networks than if dried from water (Figure 35b,c). Controlled drying kinetics coupled to CNF sizes led to materials with a wide variety of nanomorphologies, each with specific light scattering patterns, visually ranging from transparent to very opaque materials.^725^ Residual macromolecules (e.g., lignin,^136^ hemicellulose^915^ or proteins^916^ if isolated from algae) sometimes present in CNF, tend to create tighter nanonetworks, as they occupy the interfibrillar spaces across the material.

Highly porous aerogels, that is, loose nanonetworks, can be obtained from nanochitin,^632^ nanocelluloses (either crystals^917,918^ or fibrils^919,920^), alginate,^921,922^ lignin,^923^ tannin,^641^ proteins (e.g., silk,^924,925^ gelatin,^926^ collagen^927^) hemicellulose,^928^ to name a few. Aerogels, are here defined as low density materials that display a three-dimensional (3D) continuous network with length scales in the order of dozens of nanometers.^513^ Overall, the architecture and properties of the nanonetwork in such aerogels correlate with, and can be easily manipulated by the drying technique used for consolidation (e.g., supercritical drying, freeze-drying, freeze–thawing).^914,919^ For biocolloids, typically of high aspect-ratio (e.g., ChNFs, ChNCs, CNFs, and CNCs), the mass fraction of the precursor suspension must allow interconnectivity between the primary building blocks, for example, to form self-standing, robust aerogels.

Freeze–thawing and freeze-drying lead typically to networks in the macroscale, with porosity spanning few microns to over 100 μm. Supercritical drying (liquid CO_2_ drying) of biopolymeric suspensions, however, leads to nanonetworks with specific surface area (SSA) at least 1 order of magnitude higher than those obtained from traditional freeze-drying (up to 20 m^2^/g), reaching values from 200 to 400 m^2^/g. A typical biocolloid-based aerogel preparation involves an acid-induced gelation step of TO–CNF dispersion, followed by replacement of the acidic water by ethanol or acetone, to finally supercritically dry with liquid CO_2_. For instance, aerogels prepared from TO–CNF (3 nm wide) suspensions, with very low solid fraction (as low as 0.3 wt %) have been reported.^919^ The latter demonstrated the effects of processing on the final nanonetwork. The resulting aerogel comprised a nanoscale skeleton of well-individualized fibrils, with specific surface area of ∼350 m^2^/g. Other solvents, such as *tert-*butanol, have been tested for the formation of such aerogels (SSA of 160 m/g^2^) by freeze-drying TO–CNFs.^919^ In aqueous suspensions, the formation of solid water crystals is the main factor leading to macroporosity during freeze/freeze-drying; however, with eutectic mixtures, as is the case of *tert*-butanol/water system, the thermodynamics of crystallization change significantly, from the single solvent,^929^ improving both freezing and freezing drying processes. For instance, solvent-exchange/freeze-drying combination was used to achieve ChNF (partially deacetylated) networks with length scales below 100 nm and SSA over 300 m^2^/g.^930^ Many other examples in the literature corroborate such observations.^26,931−933^

Recently, a new approach to produce biopolymeric nanonetworks with SSA over 250 m^2^/g was introduced^934^ achieving superior mechanical performance when compared to the fibrillar, biopolymeric aerogels reported so far (Figure 35e). The latter involved aerogel-like nanonetworks within the capillaries of delignified wood. First, native wood (balsa) was delignified with NaClO_2_, followed by partial dissolution with DMAc/LiCl and further regeneration in acetone, followed by freeze-drying. The final wood aerogel resembled a typical delignified wood macrostructure; however, its lumina were filled by an entangled nanofibril nanonetwork (Figure 35f). DMAc/LiCl extracts components from the cell wall, by partially dissolving cellulose. Dissolved cellulose is trapped within the wood capillaries due to the slow flow kinetics within wood and probably due to the high viscosity of the solution. Upon the addition of the coagulant, the cellulose-cellulose hydrogen bonding is regenerated, leading to the coagulation of nanofibrils formed *in situ* in the lumina. A gradual increase in the SSA was observed, indicating the effects of the processing into the formation of the nanonetwork (Figure 35g).

Low-density nanonetworks formed from dissolved biopolymers share a great fraction of the research body on the subject. The process for their preparation is analogous of those used for biocolloidal suspensions. Biopolymers such as pectins, starch and chitosan have been the most exploited, given their high-water solubility and facile processability. However, biopolymers such as cellulose that demands special solvent systems for dissolution (e.g., DMAc/LiCl, ionic liquids) or conditions (below 0 °C), can be used for creating low density aerogels, as is the case of the high performance aero-cellulose system^935^ and others.^936,937^

Pectin from citrus and apple peels were converted into aerogels (named aeropectin), with length scales ranging from 20 to 50 nm, via dissolution–gelation-coagulation-supercritical drying. Dissolution of the pectin was carried at acidic pH due to a more favorable gelation taking place at such conditions, which was caused by a protonation of the carboxylate groups into carboxylic acids, which allowed intermolecular hydrogen bonding. Coagulation occurred in 1:1 H_2_O:EtOH, and the solvent was progressively exchanged to EtOH. The resulting pectin “alcogel” was then subjected to supercritical drying. A positive correlation of pectin concentration (2 to 6 wt %) with the density (0.05 to 0.17 g/cm^3^) and compressive moduli (5 to 20 MPa) was observed in the final aeropectin nanonetwork; however, the pectin content had no clear impact on the SSA of the materials, varying from 230 to 270 m^2^/g.^938^ Manipulating the processing conditions (adjusting pH, and therefore interactions between the regenerating biopolymers), aeropectin materials with 650 m^2^/g, displaying superinsulating properties, comparable to silica aerogels, were obtained.^939^ Many other biopolymeric low-density constructs have been prepared, using for instance starch^940^ and chitosan^941,942^ precursors, showing the universality of the formation process. The dissolution step and associated coagulant are the main parameters accordingly to the chosen biopolymer.

### 7.1.4 Macro-Sized Networks

Relevant to foams and sponges, the inner structuring of microporous materials (i.e., porosity, pore shape and size, pore wall thickness, and pore interconnectivity) is key for their design, fabrication, and application. Drying is critical to control ling these features, as the maintenance of the porous structure upon the removal of the solvent/dispersant may be challenging. Unlike supercritical fluids, a liquid/vapor interface is formed when liquid water is removed. With no meniscus and capillary pressure-induced stress, pore collapse is prevented under supercritical conditions.^513^ Porogen and sacrificial templating, in turn, can bring about tailor-made macropores.^943^

The most relevant methods to assemble biopolymers/biocolloids into macroporous networks, spanning dried foams and sponges, involve the postassembly removal of liquid (e.g., emulsion templating) or solid phases (hard templating by, e.g., sugar or salt crystals, polymeric particles, etc.), both with well-controlled geometries that lead to a tailored microstructure by acting as sacrificial agents. Notable examples include (1) CaCO_3_ microscale crystals serving as templates for chitosan/alginate layer-by-layer assembled hollow capsules, from which the core template is leached by dissolution with an EDTA Ca-chelating agent;^944^ (2) self-sacrificial templating by bacterial cellulose, which acts as both substrate and sacrificial microreactor;^945^ (3) poly[lactic-*co*-(glycolic acid)] microspheres coated by silk fibroin, the core being removed by dissolution in organic solvents;^946^ (4) paraffin wax and poly(methyl methacrylate) (PMMA) spheres serving as temporary templates for dual-porous regenerated cellulose scaffolds and being leached by organic solvents;^947^ and (5) PMMA particles that template chitosan hydrogels upon solubilization and washing by acetone, combined with cryogelation.^948^

**Figure 36:**
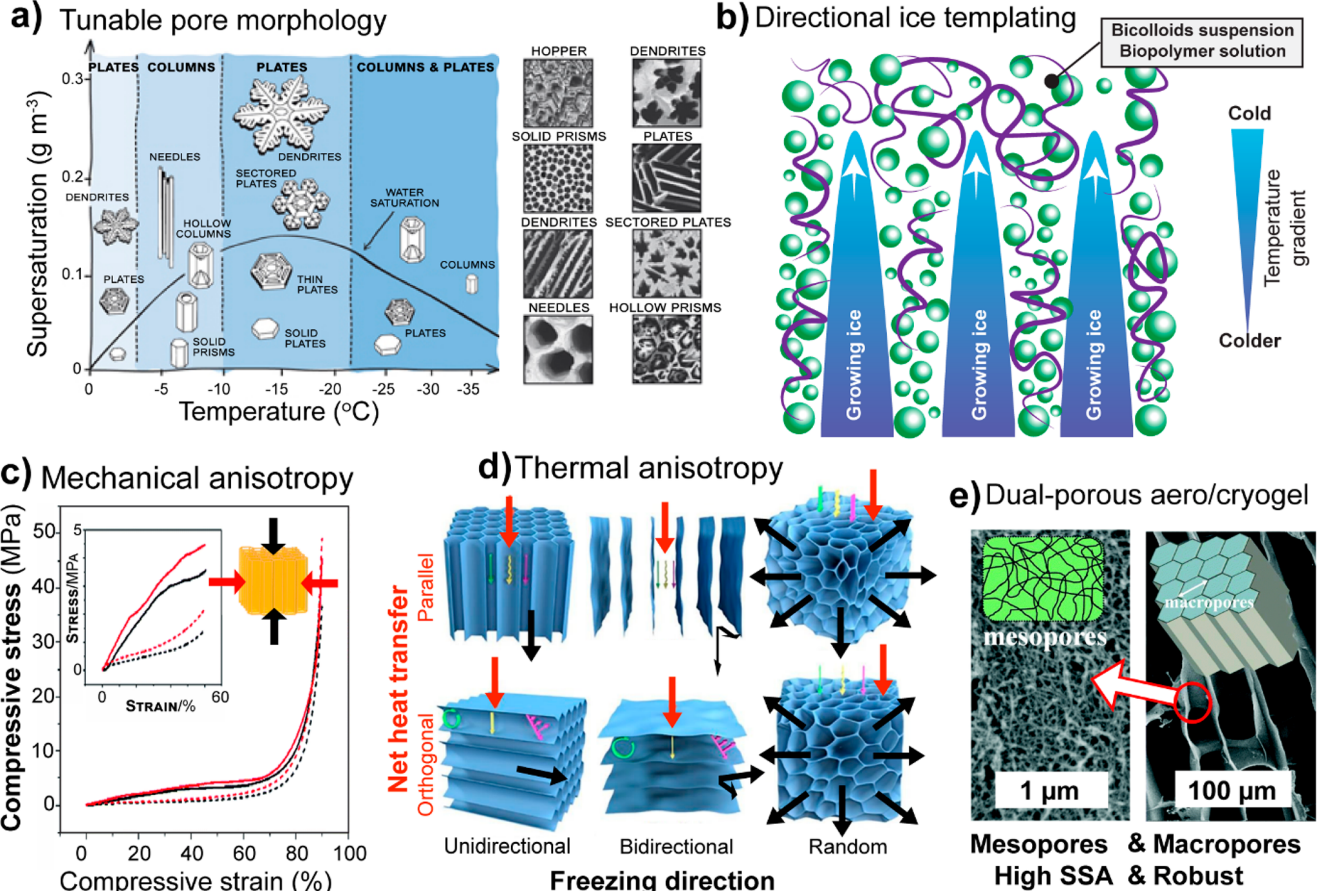
Biopolymer-based macro-networks. (a) Typical morphologies of snowflakes paralleled by ice-templated pores. Adapted with permission from ref (955). Copyright 2013 Materials Research Society. (b) Directional ice templating of a biopolymer solution or a biocolloid suspension into a lamellar or columnar microstructure due to solid ejection from the crystallization front. The white arrows indicate the direction of ice crystal growth along with the directional temperature gradient. (c) Differential mechanical behavior of CNF foams determined under compression, both parallel and orthogonal to the preferential freezing direction. Adapted with permission from ref (956). Copyright 2016 The Royal Society of Chemistry. (d) Differential heat transfer mechanisms. The orange arrows indicate the direction of the net energy transfer, and the contributions of convection, conduction, and radiation are represented by the smaller green, yellow, and pink arrows, respectively. They render (uni/bi) directionally frozen macro network-spanning foams anisotropic as far as thermal conductivity, which is different in the directions parallel and orthogonal to the preferential freezing direction (indicated by the black arrows). This property tends to be isotropic in homogeneously frozen foams. Adapted with permission from ref (957). Copyright 2019 Elsevier B.V. (e) SEM images of a non-cross-linked dual-porous CNF cryo/aerogel produced by both directional freezing under liquid nitrogen and supercritical CO_2_ drying, leading to macropores with mesoporous walls. Adapted with permission from ref (958). Copyright 2020 The Royal Society of Chemistry.

Ice crystals are by far the most widespread templating agent used after melting (freeze–thawing) or sublimation (freeze-casting). The pore morphology mirrors that of the sacrificial crystal if extensive shrinkage is prevented by proper structural integrity, as nicely exemplified for snowflake-inspired cryogels (Figure 36a). Upon sublimation, no liquid/vapor interface is developed, and therefore capillarity-induced pore collapse is minimized. However, sublimation requires high energy input, potentially representing a bottleneck for upscaled processes. An approach to eliminate the vacuum-drying step is the freeze–thawing-drying, wherein urea is used as additive and water is solvent-exchanged to ethanol to prevent pore collapse upon oven drying at ambient pressure.^949^ Otherwise, when needed, extensive shrinkage can be further prevented by covalent cross-linking. Freeze–thawed PD-ChNF hydrogels with solid contents as low as 0.05 wt % were freeze-cast into cryogels, whose solid contents (up to 0.6 wt %) were linearly correlated with density (directly) and porosity (inversely).^303^ Glutaraldehyde-cross-linking rendered foams shape-recoverable in wet conditions, a capacity that was found to remarkably depend on pH, enabled in a reversible manner in acidic media (pH 2; primary amines are protonated) but prevented in alkaline media (pH 11; deprotonated). Importantly, higher freezing rates led to weaker hydrogels due to insufficient cross-linking, as smaller ice crystals were less efficient in confining the solids in the intercrystalline lamellae.^303^ This freeze-linking process applies to cross-linker-free systems, as in the ice driven packing of sodium periodate-oxidized CNFs that were made wet-stable (then suitable for application or functionalization in liquid media, e.g., layer-by-layer assembly of multilayers)^950^ via hemiacetal cross-links between the introduced aldehyde groups and their native hydroxyls.^951,952^

Modulating the freezing rate represent a facile pathway to design the internal microstructure of solid foams, for instance by adjusting the heat conductance of the mold separating CNF^953^ or cationic CNF^954^ suspensions from the freezing medium. Adjusting the ice formation kinetics allowed tailoring the mechanical properties, owing to differential pore wall thicknesses and pore anisotropy^953^ and antibacterial efficiency, due to differential pore size and accessibility to microbial cells,^954^ of monolithic cryogels. The internal microstructure is also key when biopolymer foams serve as scaffolds for tissue engineering, as each tissue has its requirements as far as mechanical stiffness and pore size.^943^

The ice-templating approach offers the possibility of shaping the internal microstructure, not only in terms of dimensions but also architectural details. Stirred and static freezing processes, for instance, lead to CNF cryogels with remarkably different pore morphologies owing to the more heterogeneous distribution of ice crystals compared to that from quiescent conditions. Static freezing translates into a preferential crystallization direction, from outer surface to the core.^949^ This directional freezing (Figure 36b) was first applied to ceramic particle slurries that were freeze-dried and infiltrated by polymeric binders, leading to numerous organic–inorganic hybrids that were 300% tougher than their components individually.^959,960^ An artificial nacre was later obtained through the directional freezing of a chitosan solution into a lamellar 3D matrix that was then acetylated into β-chitin and infiltrated by a calcium bicarbonate solution for mineralization of aragonite nanocrystals and then by silk fibroin followed by hot pressing.^961^ In similar directions, an elegant approach was proposed, by inducing successive ice nucleation and preferential growth, providing water-contacting mold surfaces with wettability patterns.^962^ While hydrophilic and hydrophobic copper surfaces both led to short-range alignments, wettability gradients induced the formation of microstructures that were not easily obtained via conventional freeze-casting, including long-range lamellar, cross-aligned and circular lamellar patterns.

While homogeneous freezing results in isotropic foams, directional freezing creates anisotropic honeycomb-like columnar and lamellar morphologies.^963^ In anisotropic foams, the precursor building blocks, the pores and cell walls are oriented preferentially. For instance, CNCs and CNFs were highly aligned along the freezing direction, as demonstrated by the azimuthal integration of X-ray diffraction Debye–Scherrer rings.^963^ Mechanically, unidirectionally frozen monoliths are also anisotropic. The compressive modulus of mixed CMC/CNF foams has been shown to be about 1 order of magnitude higher when measured parallel to the freezing direction than the values obtained perpendicularly, meaning that the foams were stronger and stiffer along the void-column axis (Figure 36c).^956^ By contrast, virtually all flexural properties were slightly higher in the perpendicular direction. Thermal conductivity was remarkably higher in the freezing direction,^964^ as illustrated by heat transport through the channels (unidirectionally templated) or lamellae (bidirectionally templated), parallel to the ice-growth axis, Figure 36d. Heat transfer, that is, convection, conduction, and radiation, is prevented to a large extent in the orthogonal direction. The randomly frozen foam, in turn, is isotropic as far as thermal conduction, while the bidirectional anisotropic analogue performs better for directional thermal insulation.^957^

There are advantages and limitations of having mesoporosity (e.g., high SSA and low mechanical strength) or microporosity (e.g., mechanically robust but featuring low surface area, respectively). Micro and mesopores can be formed under directional freeze–thawing (leading to pores of 50–200 μm in diameter) and supercritical drying (leading to pores of 2–50 nm in diameter), respectively.^958^ The resulting dual-porous CNF foam (Figure 36e) benefit from high mechanical strength, SSA (250–450 m^2^/g, similar to supercritically dried CNF aerogels). In the process, CNFs were first cross-linked physically by HCl vapor, then directionally frozen in liquid nitrogen, thawed in acetone and ethanol (freeze–thawing was repeated cyclically) and finally dried in supercritical conditions. Mesoporous materials can be obtained from biopolymers not only via supercritical drying, but also through freeze-drying, in this case after solvent exchange to low-surface energy *tert*-butyl alcohol.^632^

### 7.1.5 Double Networks

Double networks (DN) are a class of soft and tough polymeric materials, usually in the form of hydrogels, that comprise interconnected, and hierarchically arranged networks each playing a specific role in the material’s structure. Typically, double networks comprise one rigid polymeric component as the first network, and a ductile polymer as the second network. Such configuration increases significantly the toughness, associated with the fracture energy of the materials due to the ability of one network to dissipate energy by deforming, while the other, form strong anchors preventing the fractures to initiate. Optimization of the formulation, such as molar concentration and cross-linking degree, enables the success of the double-network as well as tunability of its properties. Cross-linking can be done either by chemical, physical, or both routes.^965^ The colloidal interactions play a major role in the formation of DN from biopolymers, especially by allowing cross-linking between and within the networks, for instance by ionic or hydrogen bonding.

Double network films, synthesize by the consolidation of hydrogels, have been created by using cellulose nanofibrils as the first network and algal polysaccharides (alginate or carrageenan) as the second.^966^ CNFs were mixed (0, 10, or 30 wt %) with sodium alginate, κ or ι-carrageenan and vigorously homogenized to form a stable, translucent suspension/solution. The mixture was then filtered to form a thick wet gel that was dried under a Rapid Köthen sheet former. The dried materials were then swollen and immersed in salt solutions to promote ionic cross-linking of the algal polysaccharides with cations (Cu^2+^, Nd^3+^ and Ca^2+^). The Ca-alginate tight network (i.e., egg-box structure) was more efficient than the loosen Ca-carrageenan to suppress swelling of the CNF matrix. Double networking such biopolymers has been demonstrated to push the property boundaries beyond that of the single components, reaching values comparable to stiff rubber, *E*_*a*_ of 135 MPa, tensile strength of 17 MPa, elongation over 55% and work of fracture in the wet state of ∼5 MJ/m^3^. The introduction of cations (e.g., Ca^2+^) enabled such performance. Cross-linking, formed by ionic interactions, prevented CNFs to align under stress, leading to increased toughness by dissipating energy across the paired network.^966^ Combination of CNFs and alginate with multivalent cations has been used in other efforts, for instance to form 3D double-networks of hydrogels and aerogels.^879^ Additionally, similar materials have been prepared with the combination of bacterial cellulose (BC) and alginate, forming double hydrogels for osteochondral defect repair.^967^

Gelatin was combined with chitosan to prepare double networked hydrogels for biomedical applications.^968^ Acidic chitosan solution and gelatin powder were homogenized (45 °C), and then cooled (4 °C) until a gelled phase was observed. This resulted in helically arranged aggregates of gelatin, leading to a truncated network that was intermixed with the chitosan network. The gelled system was immersed in a sodium citrate solution at room temperature to induce ionic interactions that promote the cross-link of both biopolymer networks. Without the strong chitosan-gelatin binding, the networks would otherwise dissolve slowly in water; however, this was taken as an advantage to fabricate hollow materials suitable for biomedical application. By allowing a controlled diffusion of the citrate toward the inner parts of the gelled systems, either by controlling the concentration or reaction time, capsules and cylinders were synthesized among other hollow objects, which displayed shell thicknesses controlled by the extent of cross-linking.^968^

**Figure 37:**
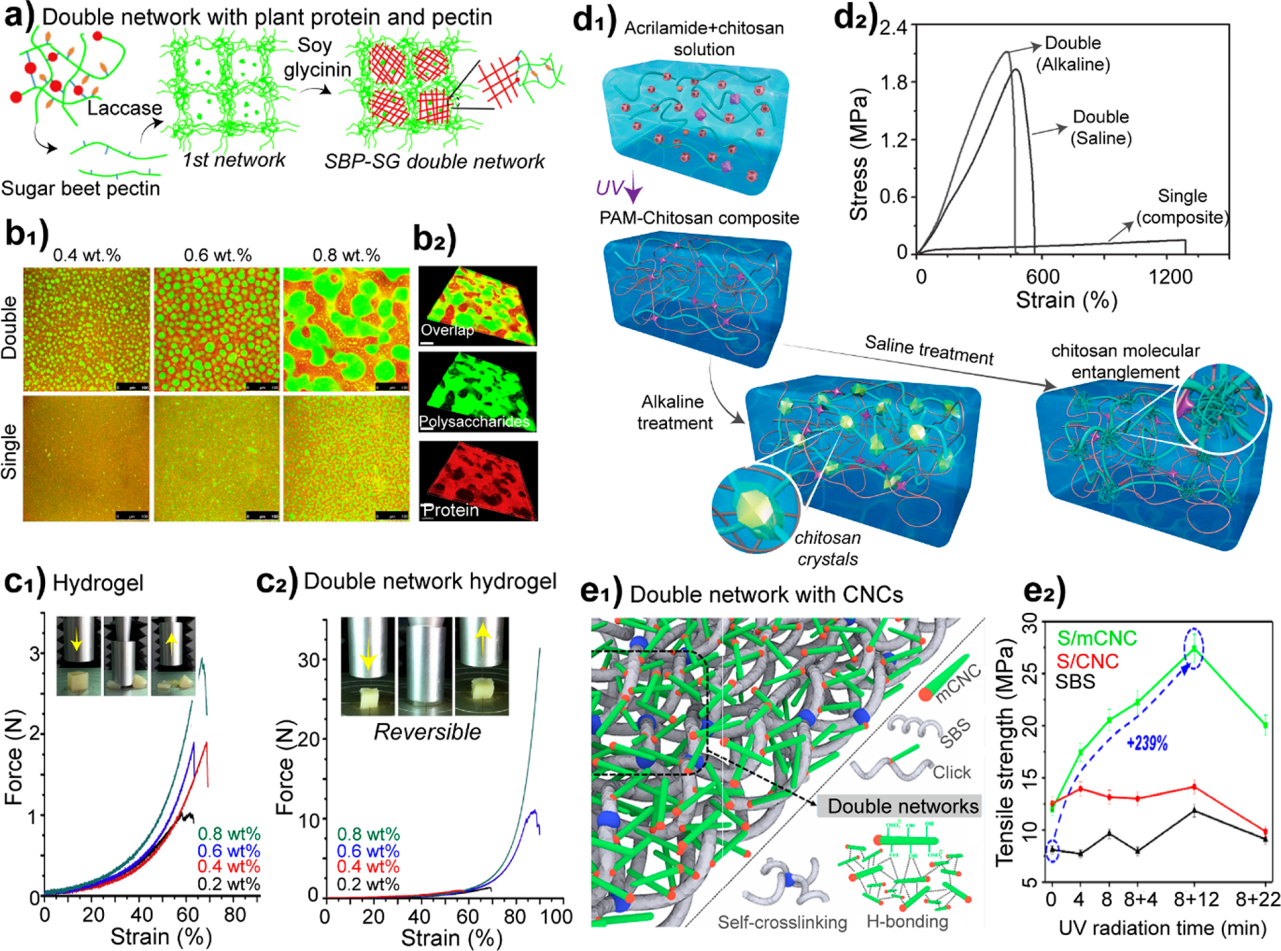
Formation of double-networked systems using biopolymer components. (a) Plant pectin (sugar beet pectin, SBP) and soy protein (soy glycinin, SG) produce a fully biobased double network by laccase-mediated reactions. (b1, b2) Morphology of both single or double networks can be tuned by the total concentration of the pectin and protein, with more pronounced changes observed for the double network. (c1) Single and (c2) double pectin/protein network differ in mechanical performance, by 1 order of magnitude, the latter displaying reversibility under compression. Adapted with permission from ref (973). Copyright 2015 Elsevier B.V. (d1) Acrylamide and chitosan form strong (d2) double networks upon treatment of the UV-cured composite under saline and alkaline conditions. Adapted with permission from ref (971). Copyright 2016 John Wiley and Sons. (e1) Modified cellulose nanocrystals (mCNC) combined with styrene–butadiene–styrene copolymer (SBS) form a double network hydrogel upon UV-activated click coupling. (e2) The modification of the nanocrystals (using thiol groups) enabled significant increase in the mechanical strength of the double networks. Adapted with permission from ref (417). Copyright 2019 American Chemical Society.

Edible double networks formed from glycinin (soy protein) and pectin from sugar beet and studied as far as the effect of formulation, associated microstructure, and properties (Figure 37a–c). Sugar beet pectin (SBP) was dispersed in acidic water to which laccase was added and incubated at given temperature and time. The first network promptly formed after cooling the solution. Laccase converted the ferulic acids in the SBP into ferulic acid dehydrodimers, which covalently cross-linked the SBP molecules. Soy glycinin (SG) was then infused in the first SBP network and the pH adjusted to neutral. Another enzyme, mTGase (microbial transglutaminase) was added to obtain the second network. mTGase modified specific protein moieties that could then strongly interact with other neighboring molecules. The SBP content played an important role in the formation of the DN (Figure 37b), allowing tailoring, by-design, the resulting microstructure. Most importantly, the mechanical performance of the DN was remarkably better (Figure 37c1) than that of the single networks (Figure 37c2), that is, those formed without enzyme modification. SBP-SG double networks sustained over 30 N load and were elastic. By contrast, the SBP-SG single networks only resisted 3 N before failing, after plastic deformation.

Synthetic-biobased hybrid double networks have been proposed. For instance, chitosan was utilized as the first network to host hydroxyethyl acrylate (HEA) as the second one.^969^ Chemically cross-linked chitosan, using glutaraldehyde, was infused with an aqueous HEA solution containing N,*N*′-methylenebis(acrylamide) (MBA) as cross-linker and α-ketoglutaric acid as photo initiator. The composite swollen system was then irradiated (ultraviolet light, 365 nm, 300 W, for 8 h at 100 mW/cm^2^) to form the double network. The compression strength of the DN varied from 20 to 80 MPa, depending on the cross-linking degree of the two networks. Overall, the lower the cross-linking degree the higher the mechanical strength, and toughness. Excessive cross-linking of the chitosan network blocked available sites for interaction with HEA, making the material more brittle and confirming that a good interface between the two networks were needed for maximizing stress transfer across the intertwined matrices.^969^

In another effort, modified gelatin was combined with tannic acid to prepare double networks that integrated high stiffness, super elasticity and self-healing properties.^970^ Gelatin was first chemically modified into a methacrylated form (by methacrylic anhydride), named thereafter GelMA. Aqueous solutions of GelMA were mixed with ammonium persulfate (APS) and tetramethylethylenediamine (TEMED) to induce gelation by oxidation and cross-linking. Then the gelled systems were immersed in tannic acid (TA) solution for given time to yield DN with given microstructures. The interactions between the two networks were purely driven by hydrogen bonding, which, given the polydentate character of tannic acid, formed multiple interactions and therefore high cohesion. The compression strength of GelMA-TA hydrogels (from 3.2 to 4.6 MPa, depending on the GelMA content) were considerably higher compared to the corresponding pristine GelMA (ca. 0.5 MPa). Tensile tests indicated stiffer DN with higher GelMA content. By decreasing the GelMA content (from 20 to 10 wt %), the elongation increased from 100 to 200%. The swelling capacity displayed a negative correlation with the TA content. Multiple hydrogen bonding between gelatin and TA rendered lower water absorption by the DN, due to the interactions between the networks being of the same nature as those with water. The short-range hydrogen bond interactions allowed self-healing properties; upon deformation (sliding of the polymeric chains upon stress) new hydrogen bond were rapidly formed with hydrogen acceptor and donors, which were present in both networks.^970^

The ability of chitosan to regenerate from solution into different polymeric arrangements has been harnessed to prepare hybrid double network hydrogels with tunable properties.^971^ Short-chain chitosan was covalently cross-linked with a poly(acrylamide) (PAM) network, followed by a simple soaking step, in either saline or alkaline media, to convert the composite system into a high-performance DN hydrogel by the formation of chitosan-microcrystalline structures and chain entanglement networks (Figure 37d1). The short-chain chitosan in the composite hydrogel moved freely and rearranged under the treatment of alkaline and saline solutions. When immersed in alkaline media (1 M NaOH), the chitosan amine groups became deprotonated, reducing ionic repulsion as well as hydrophobic interactions, which induced crystallization of the system into microdomains that physically cross-linked the networks. In the presence of salt (NaCl), a salting out effect occurred, shielding the electrostatic repulsions, thus increasing hydrophobic intrachain interactions, leading to intermolecular aggregates that served as connecting points. Both routes, alkali and salt addition, led to remarkably higher mechanical strength upon compression, when compared to the composite hydrogel (before post treatments) (Figure 37d1,d2). Compression stress was over 1.5 MPa for the double networks following alkaline post treatment, leading to a stiffer material, compared to values below 0.2 MPa for the composite chitosan-PAM hydrogel. The toughness was remarkably improved for the double network, with values at least 1 order of magnitude higher.

Biopolymers have enabled the formation of double networks even if used as minor components. For instance, lignin polymeric networks were used as a minor component (up to 2.5 wt %) to physically cross-link PU, forming tough and highly adherent double networks.^972^ Inter network hydrogen bonding took place upon removal of water when lignin was mixed with hydrophilic polyether-based PU followed by drying. After drying, the formed hydrogen bonds did not break in the presence of water, but the material swelled into a hydrogel state. The presence of 2.5 wt % lignin increased the fracture energy and Young’s modulus of the PU hydrogels, from 1540 to 2050 J/m^2^ and 1.3 to 2.6 MPa, respectively. Double networks of PU with lignin allowed high elasticity, with an immediate load recovery of 95%, and significantly increased the lap shear adhesiveness, from 3 to 8 kPa. Although not thoroughly investigated, lignin has a large potential for the formation of double networks due to its amphiphilic and polydentate character, its solubility in a variety of solvents, as well as its availability.

Double-networking with chemically modified CNCs improved remarkably the properties of styrene–butadiene–styrene copolymer (SBS), a thermoplastic elastomer.^417^ First, CNCs extracted from cotton linter were chemically modified in their reducing ends by the aldimine condensation, to introduce thiol groups (-SH). Then, the modified CNCs (mCNC), as well as the unmodified nanoparticles used as a reference, were added (10 wt %) to SBS to form double networks. In this process one of the networks was formed by the SBS self-cross-linking and the other by the CNCs self-interacting via hydrogen bonding as well as interacting with SBS via the end-modified group that underwent strong, specific coupling via click reaction (Figure 37e1). From a homogeneous mCNC/SBS system, the double network was obtained by exposure to an ultraviolet irradiation (365 nm) for few minutes, which induced the click reaction between mCNCs and SBS components. The systems were later dried to obtain films. The interconnectivity between the two networks could be controlled to a certain extent by the irradiation time, which affected the mechanical properties of the system. There was a remarkable increase (ca. 240%) in film’s tensile strength with irradiation time, up to 20 min, after which there the mechanical properties started to decline. No improvement in the strength was observed for unmodified CNCs and pure SBS (Figure 37e2). The strong interactions of the CNC network with the SBS matrix increased in line with the UV-induced CNC-SBS click reactions; however, excessive connectivity limited CNC–CNC interactions; thus, a balance between network intra- and interactions was key for maximizing the mechanical performance.

### 7.1.6 Blocky or Multidomain Systems

**Figure 38:**
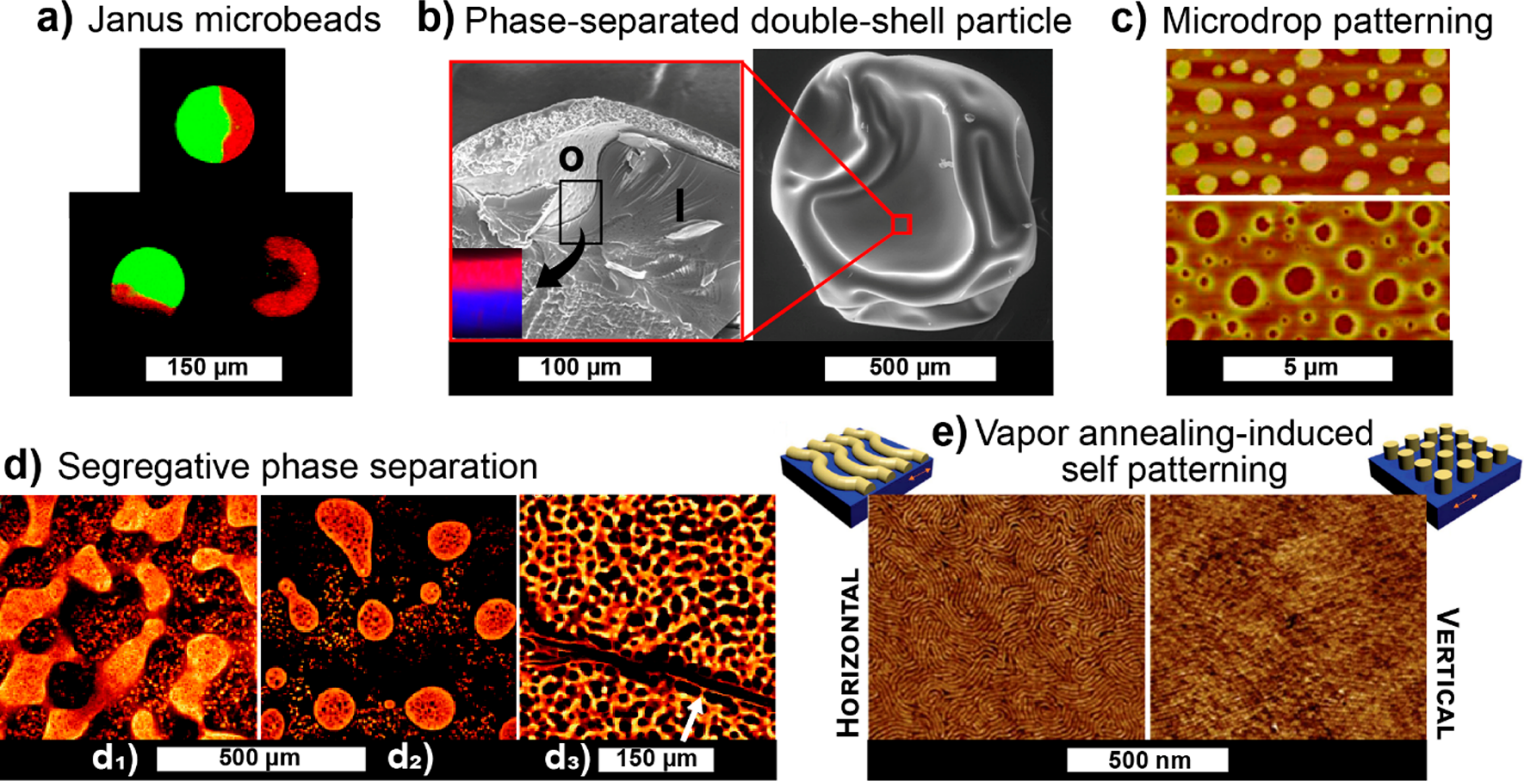
Engineered multidomain biopolymer systems. (a) Janus microbeads made from alginate (green) and pectin (red), before (top) and after (bottom) selective enzymatic hydrolysis with polygalacturonase type II (left) and alginate lyase (right). Adapted with permission from ref (978). Copyright 2012 American Chemical Society. (b) SEM of a segregative phase separated particles with outer (O) HPMC-rich phase (red layer in the confocal Raman micrograph) and inner (I) maltodextrin-rich phase (blue layer in the inset). Adapted with permission from ref (979). Copyright 2015 American Chemical Society. (c) AFM of microdrop-patterned films from SPS of PHB/cellulose, selectively degraded by PHB-depolymerase (top) and cellulase (bottom). Adapted with permission from ref (980). Copyright 2016 American Chemical Society. (d) Whey protein/gellan gum mixtures after SPS in the bulk (d_1_, note the isotropic bicontinuous microstructure) or confinement-patterned between cover glasses (d_2_, note the columnar microstructure perpendicular to the glass surface) or within a CNF network. Note the bright protein-rich wetting layer adjacent to the fiber in d_3_ followed by a dark phase rich in gum that is depleted from the former layer, which is connected to the bicontinuous bulk by fluid tubes, indicated by the arrow. Adapted with permission from ref (981). Copyright 2013 The Royal Society of Chemistry. (e) AFM of horizontally- and vertically oriented amphiphilic glycopolymer self-patterned over a TO–CNF substrate upon vapor annealing using different solvents. Adapted with permission from ref (982). Copyright 2020 The Authors.

Numerous natural systems benefit from the presence of blocky or multidomain architectures. For instance, a hierarchical protein assembly in multidomain hybrid fibers explains why spider prey-wrapping silk is the toughest natural polymer, with aciniform silk showing a high extensibility (>80% prior to failure)^974^ and ultimate strength (ca. 700 MPa).^975^ This performance arises from the interplay between α-helical (ca. 50%) and random-coil (ca. 35%) secondary structures decorated with crystalline β-sheet nanodomains (ca. 15%).^976^ This is an example of a single-component blocky system displaying different conformational states, which occur either naturally or can be induced. Single-component constructs are multidomain when the microstructure is the same, but a given functionality, within the sample, differs from one region to the other (such as in Janus interfaces). As far as biopolymers, hemispherical or spatial-dependent anisotropy may be achieved through site-specific functionalization of CNC films for example, through periodate oxidation and ozone treatment, which introduce, respectively, aldehyde and carboxyl functionalities at the different CNC ends.^977^ CNC stiffness and chemical polarity within cellulose chains aligned parallel in the native crystals represent an opportunity for asymmetric functionalization, which in turn enables the design of new materials, including Janus hairy CNC particles,^352^ thermoresponsive star-shaped particles,^408^ and others, as summarized recently.^410,411^ Further exploiting Janus assembly, single- and double-biopolymer Janus microbeads were produced by extrusion of aqueous solutions of pectin (into homo Janus beads) or of pectin and sodium alginate (into hetero Janus beads) using sunflower oil in a microfluidic flow-focusing device, following ionic cross-linking.^978^ The monodisperse microbeads were selectively hydrolyzed with polygalacturonase type II (pectin) and alginate lyase (alginate) (Figure 38a).

Associative phase separation was introduced earlier as a mean to form gels using electrostatic interactions (Section 5.3.1.1). The repulsive–not necessarily Coulombic–counterpart to this phenomenon is the segregative phase separation (SPS), which is driven by the thermodynamic incompatibility among two macromolecules dissolved in a common solvent, as predicted by the Flory–Huggins theory.^983^ The liquid–liquid phase separation leads to two polymer-rich phases with asymmetric composition, that is, the separating polymers are concentrated in the respective separating phases, where the other polymer being depleted.^984^ A classic example of SPS into aqueous two-phase system (ATPS) are the water-in-water (W/W) emulsions. Differently from conventional emulsions–microemulsions excluded–that display only kinetic stability, W/W emulsions are in thermodynamic equilibrium, are all-aqueous systems, feature low interfacial tensions, and find use in a range of emerging applications, for example, microreactors and templates for colloidosomes.^985,986^

SPS happens under conditions and can be induced during processing, with changes in composition as solvent is removed upon drying. Phase separation between corn starch and cellulose ether hydroxypropyl methylcellulose (HPMC) was observed when casting blended films, especially when the film-forming solution was subjected to high shear via microfluidization, leading to surface segregation, that is, with one HPMC-rich and another starch-rich phase.^987^ This approach to introduce, in a single layer, different functionalities and to develop surface-dependent behaviors. The drying-induced SPS that occurs with HPMC and maltodextrin result in films (casting), multiparticle powder (spray drying), and single particles (ultrasonic levitation; see Figure 38b).^979^ The effects of solid content, phase ratio and drying time on the phase segregation phenomenon, indicated that segregation is prevented at low solid contents but, otherwise, kinetic factors lead to thermodynamically driven SPS. The morphology of SPS systems can be tuned by the phase ratio, switching from lamellar-like to bicontinuous, wherein the two phases are interconnected. By adjusting the components and introducing selective enzymatic hydrolysis, patterned spin-cast films originated from SPS involving poly-3-hydroxybutyrate (PHB) and trimethylsilyl cellulose (THMC), which was later treated with HCl vapors.^980^ A range of PHB/THMC ratios led to SPS of different morphologies, and selective hydrolyses by cellulase from *Trichoderma viride* or PHB-depolymerase were used to remove one of the components into nonlithographically patterned films (Figure 38c). A similar approach was used to fabricate nanopatterned chitosan/bovine serum albumin films with tunable microstructure (i.e., salami, continuous, porous and droplet-matrix structures), achieved by adjusting the phase ratio and shear severity. In these cases, the sacrificial phase was removed by selective solvent etching.^988^ Humidity change has been introduced as a simple strategy to control the drying rate and patterning of SPS in biopolymer blends, leading to a range of phase morphologies.^989^

Confinement can be used to tailor the phase evolution and final morphology of SPS. Spinodal decomposition, the major mechanism driving SPS, has a characteristic wavelength that increases with time and over which confinement becomes increasingly important as this dimension approaches that of the confinement. Wetting layers have been formed by the so-called surface-directed spinodal decomposition, as demonstrated for whey protein/gellan gum mixtures.^981^ Meanwhile, bulk-phase SPS led to a bicontinuous morphology, under confinement between two cover glasses or within a CNF network, which led to columnar structures by alternating bicontinuous bulk and whey protein-rich phases, preferentially wetting the surfaces and separated from the bulk by a phase that was rich in gum (that was depleted from the wetting layer) (Figure 38d).

Finally, SPS-assisted patterning has been shown as a strategy that is suitable for copolymers, including carbohydrate-based block copolymers that self-assembled into periodic structures patterned with molecular-level precision into thin nano-organized films. In line with the CNF-confined assembly discussed previously, an amphiphilic diblock of glycopolymer polystyrene-*block*-maltoheptaose (PS-*b*-MH) was spin-cast onto different substrates, including CNF, prior to solvent vapor annealing.^982^ The interactions between the carbohydrate block (MH) and CNF guided copolymer orientation. By using a water-rich solvent (water:THF weight ratio 1:1) a random distribution of horizontally oriented chains was obtained. A solvent rich in THF (water:THF 1:15), a better solvent for the hydrophobic block, led to a hexagonally close-packed arrangement of vertically oriented chains (Figure 38e). Regardless of the annealing protocol, the topographical features of the CNF substrate were transferred to the blocky constructs, allowing multidomain thin films to be patterned with an interdomain spacing of 10–15 nm, far lower than the advanced lithography-based patterning techniques.

### 7.1.7 3D-Printing-Based Structures

**Figure 39:**
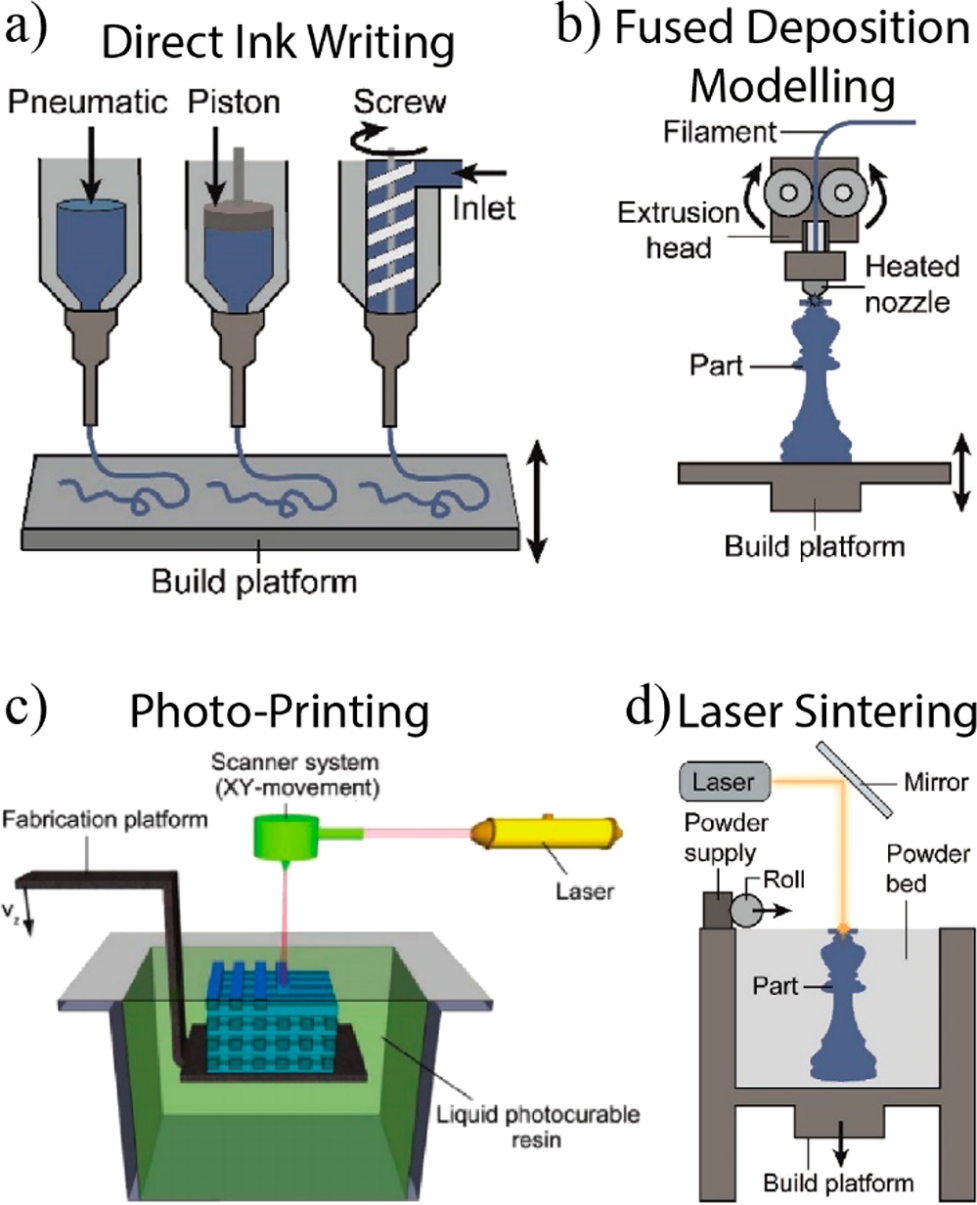
Main biopolymeric 3D-printing approaches. (a) Direct ink writing. (b) Fused deposition modeling. (c) Photoprinting. (d) Laser sintering.^1006,1012^ Adapted with permission from ref (1006) and ref (1012). Copyright 2020 John Wiley and Sons.

One of the principal techniques currently explored to study material formation with micro- and, particularly, macro-scaled textures is 3D-printing, as extensively discussed in recent reviews.^990−996^ Importantly, the vast majority of efforts are associated with plant-based biopolymers, given their availability at large scales to enable large-area fabrication. Emerging approaches using chitosan^997−1000^ and proteins^1001−1004^ are noted. 3D-printing biopolymers can combine efficiently multiscaled forces, as described in Section 4, with a phenomenology and associated consolidation methods (sol–gel and gels-to-solids) such as those described in Sections 5 and 6. In fact, in many instances, Sections 4 to 7 introduce developments associated with 3D- and 4D- printing. Currently, the vast majority of research efforts related to biopolymers involve the direct ink writing (DIW) method (Figure 39a), which relies on the direct extrusion of an ink solution or dispersion, usually exhibiting special rheological properties (see Section 5 for more information). Other techniques include fused deposition modeling (FDM) (Figure 39b), which is applied by melting thermopolymers and their composites.^1005^ Lastly, photoprinting technology and laser sintering are approaches that can use biopolymeric derivatives together with photoactivation, to cross-link the printed element, or with laser heating, to fuse the powder bed (Figure 39c,d).^1006^ As additive manufacturing progresses and becomes a more established form of manufacturing, one can expect biopolymeric materials to introduce unique opportunities in this field. For instance, the anisometry of the deconstructed biocolloids enables swelling and responsive morphing into predesigned shapes.^484,864^ Another example taking advantage of aqueous processing is direct “cryo-writing” of nanocellulose into 3D aerogels, wherein DIW is used on a cold plate to instantly freeze the dispersion into a solid, which can retain its shape after removal of water.^1007^ Other considerations in multidimensional printing of biopolymers may exploit specific interactions in the context of graded structures or multimaterial, multinozzle printing.^1008,1009^ The latter can be used in the context of microfluidic printing, which enables fine optimization of the resolution and a higher versatility in composition for high-end applications.^1010,1011^

## 8 Natural and Engineered Synergies

**Figure 40:**
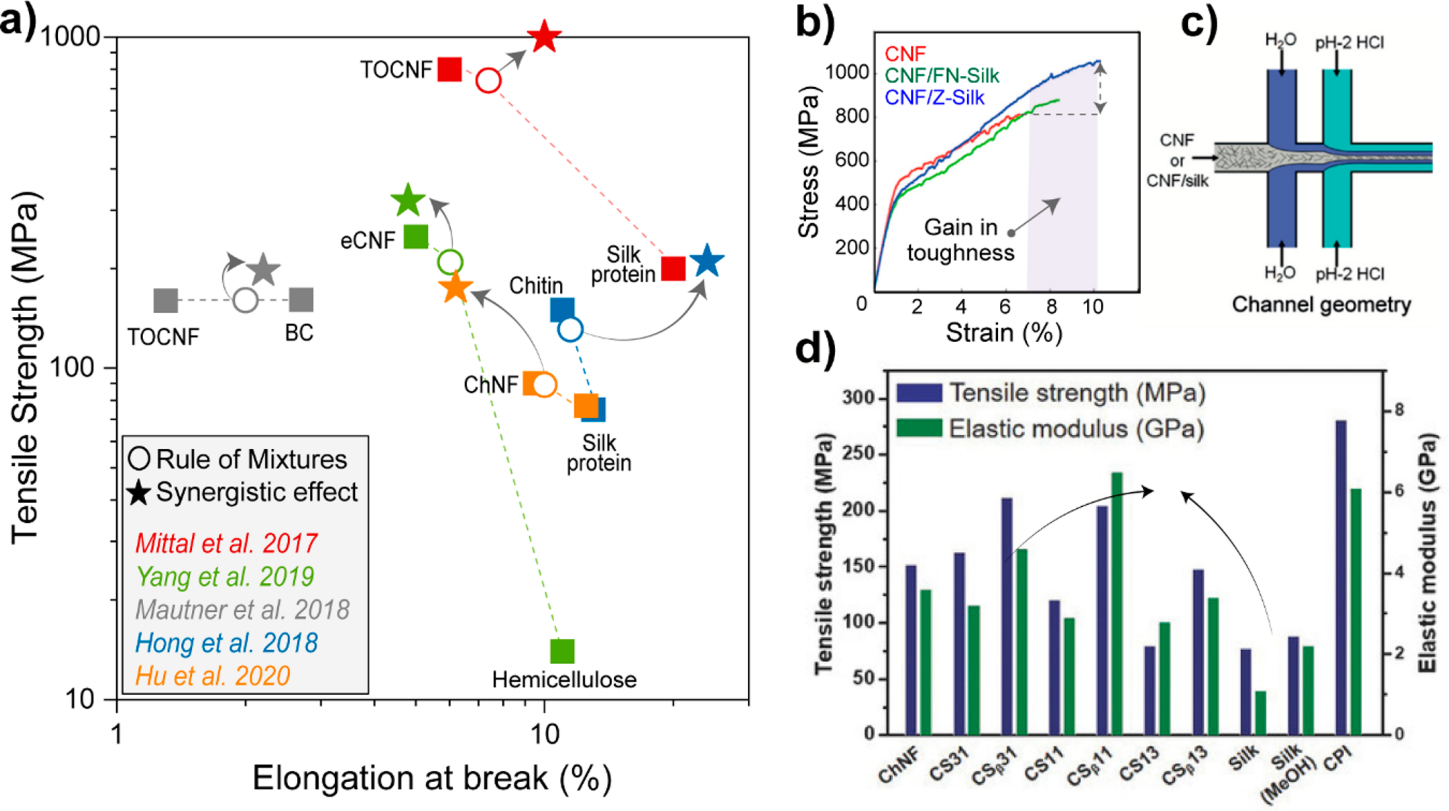
Synergistic effects observed in biopolymeric multicomponent materials. (a) Comparison of material strength according to a simple rule of mixture and results obtained from synergistic effects. The plot was constructed by using data from refs (212, 487, 1017, 1019−1021, and 1028). (b, c) Compared to pure TO–CNFs, the alignment of TO–CNFs in the presence of silk infusion proteins produced a clear gain in toughness and ultimate tensile strength. Adapted with permission from ref (487). Copyright 2017 American Chemical Society. (d) Chitin nanofibrils display synergistic interactions with silk proteins, especially in the presence of β-sheets arrangements. Reprinted with permission from ref (1019). Copyright 2018 John Wiley and Sons. Note: Further permissions related to the material utilized in (b, c) (pubs.acs.org/doi/10.1021/acsnano.7b02305) should be directed to the American Chemical Society.

In this section we discuss biopolymeric composite materials displaying cooperative interactions between the components, which push forward the upper boundaries of mechanical cohesion. Synergism is a nonlinear cumulative effect that cannot be predicted by the general rule of mixtures used to estimate the maximum value achievable for a property, such as mechanical strength, considering the volume fraction and strength of each component individually. The rule of mixtures does not account for interfacial interactions,^1013^ which in the case of biopolymeric composites can be as high as those for biopolymer self-interactions. Herein we use literature data to illustrate the synergies that exist in biopolymeric composites and compare their performance with those of individual components as well as from rule of mixture estimations (Figure 40a).

Interestingly, synergistic effects are not only linked to the building blocks, but strongly associated with the processing and conditions used for their consolidation into a composite. For instance, whereas several silk fibroin/cellulose nanostructured composite films display no synergies between the components,^1014−1016^ filaments produced from the same building blocks have shown strong synergism, leading to a remarkable mechanical performance.^487^ This is the case of TO–CNFs and silk fusion proteins combined at a 90/10 ratio and spun in a double flow-focusing microfluidic channel (Figure 40b,c).^480^ The TO–CNF neat filament performed already very well, with an ultimate tensile strength of ∼800 MPa; however, the addition of 10 wt % of silk proteins, which individually have a maximum strength of 200 MPa,^1017^ produced a tensile strength of 1000 MPa. The high elongation of the composite material, provided by the silk proteins, led to a high toughness ∼55 MJ m^–3^. The tight packing of the building blocks, and therefore high density of interfacial interactions, are major reasons for the synergistic interactions in such filaments, which otherwise were absent in films, whose nanoarchitecture was formed by randomly aligned layered elements. The observed effects are rather ubiquitous in natural composites but can be brought to a higher level in the case of engineered materials.

In addition to processing, the nature of the components (e.g., size or crystalline arrangement) favor their synergisms. Contrary to mechanically fibrillated cellulose nanofibrils,^1014−1016^ used for the production of composite films, regenerated cellulose showed a synergistic effect with silk fibroin.^1018^ For instance, cellulose and silk fibroin were dissolved in BmimCl ionic liquid, gelled in methanol, washed with water and dried at room temperature. The neat regenerated silk fibroin material was too brittle, thus preventing any measurement; however, previous reports showed silk fibroin films with an ultimate tensile strength of ∼75 MPa.^1019^ Meanwhile, the film of neat regenerated cellulose displayed a tensile strength of ∼120 MPa. However, it increase to 170 MPa was with the addition of silk proteins.^1018^ The regeneration into a tightly packed, nonporous, material favored interactions at the cellulose-silk interface, as investigated by NMR. Strong intermolecular hydrogen bonding was observed between -NH in silk and −OH at C2 and C3 position in cellulose, thus revealing that the synergies arose from the intimate mixing and specific regiochemistry of hydrogen bonds between silk and cellulose.^1018^

Silk fibroin and chitin colloids (ChNF) also showed synergistic effects in terms of the cohesion of composite materials.^1019,1020^ The ratio between each component, mostly in binary systems, was an additional feature affecting their synergism toward enhanced strength. Silk fibroin and β-chitin (from *squid pen*) at 3:1, 1:1 and 1:3 mass ratios were dissolved, cast, treated with alcohols, and hot-pressed to prepare films at varied content of each component, and also to induce a transition from random-coil and α-helix structures into β-sheet arrangement. Composites comprising chitin as the dominant matrix (ChNF:silk 3:1) displayed better mechanical performance than the one made mostly from silk (ChNF:silk 1:3); however, both were stronger than pure silk. The ChNF:silk 1:3 composite was not particularly strong when compared to the pure chitin film (Figure 40d). At an equal mass ratio or at higher chitin content, the synergistic effect resulted in a composite tensile strength of 200 MPa, significantly higher than that of neat chitin (150 MPa) or silk (75 MPa) **(**Figure 40a). Interestingly, when the dominant molecular structure of the silk was the β-sheet arrangement, the films became stronger than their counterparts. Although not completely understood, it appears that the synergy arises from additional hydrogen bonding and chitin-silk-chitin cross-linking as well as the minimization of defects in the randomly entangled ChNF network.^1019^ Addition of degummed silk nanowhiskers to deacetylated ChNFs (DE–ChNF), by up to 5 wt %, led to synergistic enhancement, from 90 MPa (pure DE–ChNF film) to 175 MPa (DE–ChNF-Silk composite film). Note, though, that the incorporation of 10 wt % silk colloids to the chitin matrix produced slightly weaker materials.^1020^ All in all, the synergies between silk and polysaccharide building blocks depend on the formulation, the nature of the building blocks, and the processing routes used.

Cellulose colloids with varied morphology and dimensionalities (e.g., bacterial cellulose and TO–CNF) were compounded to enhance the mechanical strength of all-cellulosic materials. Films formed from suspensions of pure bacterial cellulose (BC) (after shredding BC pellicles) presented a tensile strength of ∼160 MPa and elongation at break of ∼3%. TO–CNF displayed the same mechanical strength but produced stiffer films (elongation at break of ca. 1.3%). When composited at a 1:1 mass ratio, the resulting BC/TO–CNF material displayed a tensile strength at ∼200 MPa, with an intermediate elongation (2.2%). Therein, the effects cannot be explained solely by void filling, which decreases the density of defects of the materials. The composites containing nanofibers of different dimensionalities induced a double energy dissipating mechanism, where energy transfer was enhanced at both short and long relative deformations.^1021^ At shorter strains, energy dissipated through the pulling out of the infused TO–CNF network, whereas at higher deformations the BC fibrils slid pass each other, leading to bond breaking-reforming mechanisms that typically induce high cohesion in fibers. CNFs and CNCs did not exhibit such cooperative effect in spun composite filaments (CNF/CNC mass ratio of 4.4/1), albeit strong (up to 330 MPa), they were weaker than those from pure CNFs (ca. 443 MPa). In fact, the tensile strength of resulting CNF/CNC material was below the maximum value estimated by the rule of mixtures, 350 MPa.^1022^ Therefore, for cellulosic composites, the matching of component dimensions is an additional consideration to induce multiscale energy dissipation mechanisms.

Mixing low-MW carbohydrates, such as hemicellulose, with cellulose biocolloids significantly enhances the mechanical properties.^1023,1024^ This is in fact analogous to the role of hemicelluloses in the development of fiber cohesion in living plants, involving central cohesion-inducer components, that is, cellulose and lignin.^118,1025−1027^ Hemicelluloses usually form weak materials (tensile strength of ca. 15 MPa^1028^), but they effectively enhance the mechanical properties of cellulose-based matrices, even if added as a minor fraction of the material (up to 20%) (Figure 40).^212,1029^ In fact, when residual hemicelluloses were left intentionally on the surface of cellulose nanofibrils, the cohesion of the respective materials was significantly higher than that of pure cellulose counterpart.^212^ Cellulose nanofibrils containing 17 wt % of glucomannan and 9 wt % xylan displayed an increased tensile strength, up to 320 MPa, ∼28% higher than the pure CNF film.^212^ This result surpassed greatly the value estimated by a simple rule of mixtures, ∼200 MPa, that accounts for only physical reinforcing but not for interfacial effects. Hemicelluloses infused in cellulose colloids enhance the energy transfer between fibrils, by creating continuity among the fibril interstices; additionally, hemicelluloses are highly adhesive toward cellulosic surfaces^122^ therefore they act as bridges between the already strong colloids.

## 9 Outlook

We linked the interactions that occur in biopolymers, their constructs, and functions. In the process, it became clear that self-assembly is paramount to achieve the desired material performance. The most common biopolymers are synthesized following biological pathways in the presence of water. Evidently, such systems can also be disassembled using aqueous solvents. Accordingly, this review connects water-based processing, from fully dispersed to aggregated gels, to achieve functional biopolymeric architectures. We discussed the development of cohesion, induced by supramolecular and particle interactions, which depends on processing and is associated with assembly forces. Research during the past decade has provided insightful information about related phenomena, and hints at the possibility of rethinking processes currently used for manufacturing. Evidently, this is much needed in our efforts to scale up the synthesis of bioproducts from natural building blocks.

It took over 40 years to transfer the early findings related to synthetic polymers by Staundinger into industrial practice, when the prevalent competing polymeric materials were in fact biobased. At present, the reverse opportunity is accelerated after the realization of the negative environmental and health impacts associated with materials produced from fossil carbon. However, little change will occur unless regulation, policy making, public perception, government as well as entrepreneurship initiatives are in place. Moreover, societal involvement and cross-boundary efforts toward a growing biobased industry are needed to tackle the global challenges, including resource scarcity and climate change.

Although, there have been great advances in efforts to reengineer biopolymers into sustainable materials, processability and performance remain challenging compared to synthetic alternatives. The latter situation is best approached if one is armed with a full understanding of the nature of the interactions that exist in biopolymer systems. The potential is evident if one considers the properties of the building blocks. For instance, the mechanical properties of renewable nanomaterials surpass those of modern day thermopolymers. However, there is a need for a materials paradigm shift. This is best exemplified in the discussion of Sections 7 and 8, where innovative approaches were shown to enable new property spaces, opening new opportunities for biopolymeric constructs. As such, the replacement of synthetic systems might not be the main goal; instead, new classes of materials can be designed by taking advantage of the inherent, multiscale hierarchies already present in biopolymeric building blocks derived from plant and animal biomass.

Development and adoption of sustainable surface reactions will be important to improve or tailor interfacial properties of biobased materials. Understanding the fundamentals of the interactions that exist in supramolecular and superstructures is central to engineer new materials and to optimize their use, reuse and end of life. The latter aspects were not addressed in this review but should be measured very closely. In such efforts, conventional recycling streams will extend the lifespan of biopolymers, by reengineering the interactions. This has been a critical endeavor in the use of synthetic materials but is lagging behind for those that are biobased, although existing value chains, such as those of paper-products, may be used as a foundation.

As the materials aspect of biopolymers were put forward in this review, the availability of biomass, from residual streams or from the forest products industries, together with the emerging biorefineries, offer major opportunities in our efforts to build a sustainable society. In response to the current consumerism, biopolymeric materials should facilitate the rational use of local resources in material manufacture and in harmony with sustainable biomass supply, from well managed land, forest and agriculture and, especially, from waste or side streams. Full integration and use of every component in the available bioresources will make the use of biopolymers a truly sustainable option as building blocks to fulfill our materials needs.

New solutions to current and future material needs will benefit from the integration of biotechnology in the production, assembly, and use of renewable polymers.

## Supporting Information Available

Description of procedure and criteria used to prepare Figures 1 and 21 as well as references described in Figure 21 categorized by type of building block (PDF)

## Abbreviations

α: hydrogen bond activity
β: hydrogen bond basicity
β–α: net basicity
ASA: alkenyl succinic anhydride
AR: acrylic resin
BNC: bacterial nanocellulose
BSA: bovine serum albumin
CNT: carbon nanotube
ChNC: chitin nanocrystal
ChNF: chitin nanofibrils
CMF: cellulose microfibril
CNCs: cellulose nanocrystals
CNFs: cellulose nanofibrils
DE–ChNF: deacetylated chitin nanofibril
DIW: direct ink writing
DLVO: Derjaguin–Landau–Verwey–Overbeek
DMAc: dimethylacetamide
DMF: dimethylformamide
DMSO: dimethyl sulfoxide
DNA: deoxyribonucleic acid
DS: degree of substitution
DP: degree of polymerization
DD: degree of deacetylation
DM: degree of methyl esterification
FDM: fused deposition modeling
GA: gallic acid
G: guaiacyl
H: P-hydroxyphenyl
HDPE: high-density polyethylene
HPMC: hydroxypropyl methylcellulose
HPC: hydroxypropylcellulose
IL: ionic liquid
MW: molecular weight
MWCNT: multi-walled carbon nanotube
MAPP: maleic anhydride-modified polypropylene
NFC: natural fiber composite
NMMO: *N*-methylmorpholine *N*-oxide
NMR: nuclear magnetic resonance
NPM: *N*-methyl-2-pyrrolidone
PP: polypropylene
PLA: poly(lactic acid)
PE: polyethylene
PHB: poly(hydroxybutyrate)
PVA: poly(vinyl alcohol)
PA6: nylon-6
PD: partial deacetylation
PG: pyrogallol
PC: pyrocatechol
R&D: research and development
Rh: hydrodynamic radius
SPS: segregative phase separation
S: syringyl
SPI: soy protein isolate
SWCNT: single-walled carbon nanotube
TEMPO: (2,2,6,6-tetramethylpiperidin-1-yl)oxyl
TO–CNF: Tempo-oxidized cellulose nanofibrils
THMC: trimethylsilyl cellulose
vdW: van der Waals
WPI: whey protein isolate
WPC: wood polymer composite
