# Supplementary material for: Deconstruction and Reassembly of Renewable Polymers and Biocolloids into Next Generation Structured Materials
Source: Chem Rev. 2021 Aug 20;121(22):14088–188. doi: 10.1021/acs.chemrev.0c01333 (PMC8630709; doi:10.1021/acs.chemrev.0c01333)
Supplement: Supplementary file 1 — cr0c01333_si_001.pdf [file cr0c01333_si_001.pdf]

# Supporting Information

# Deconstruction and Reassembly of Renewable Polymers and Biocolloids into Next Generation Structured Materials

*Blaise L. Tardy,[1,*] Bruno D. Mattos,[1] Caio G. Otoni,[2,3] Marco Beaumont,[4,5] Johanna Majoinen,[1] Tero Kämäräinen,[1] Orlando J. Rojas[1,6,*]*

[1] Department of Bioproducts and Biosystems, School of Chemical Engineering, Aalto University, P. O. Box 16300, FI-00076 AALTO, Finland.

[2] Department of Physical Chemistry, Institute of Chemistry, University of Campinas, P. O. Box 6154, Campinas, SP 13083-970, Brazil.

[3] Department of Materials Engineering, Federal University of São Carlos, Rod. Washington Luís, km 235, São Carlos, SP 13565-905, Brazil.

[4] School of Chemistry and Physics, Queensland University of Technology, 2 George Street, Brisbane, QLD 4001 Australia

[5] Department of Chemistry, Institute of Chemistry of Renewable Resources, University of Natural Resources and Life Sciences, Vienna, A-3430 Tulln, Austria.

[6] Bioproducts Institute, Department of Chemical and Biological Engineering, Department of Chemistry and Department of Wood Science, University of British Columbia, 2360 East Mall, Vancouver, BC V6T 1Z4, Canada.

*Authors for Correspondence: Orlando J. Rojas, E-mail: orlando.rojas@ubc.ca, Tel: +1 604 822 3457 and Blaise L. Tardy, E-mail: blaise.tardy@aalto.fi Tel: +358 5059 79156

## Number of patents and publications

Patent search was performed with the Espacenet worldwide patent search engine. The number of annual publications on each material was carried out using Scopus with search limited to titles, abstracts and keywords limiting to the 'article' document type. The search terms used for different materials are listed in **Table S1**, plural forms were also used when applicable. The reference to global patent output was made through 1990–2018 based on the WIPO Patent Report: Statistics on Worldwide Patent Activity by the World Intellectual Property Organization (WIPO), while the total global scientific publication numbers are based on the Science and Engineering Indicators of the National Science Foundation between 2000–2018.

## Material property Ashby plot

Literature search was carried out to determine the bounds for the material property values displayed in Figure 21 in the main text with emphasis given to years 2010–2020. The outlined regions thereby depict most accurately the peak mechanical performance of the materials. Publications that reported both Young's modulus and ultimate tensile strength were only used. Composites and crosslinkers were excluded, while plasticizers such as glycerol were permitted for natural gums and protein-based materials due to their inherent brittleness.

The surface functionality of nanomaterials may vary. The references used to define the regions are listed in **Table S2**.

**Table S1**: Keywords used in the determination of annual patent and publication numbers when it consisted of multiple.

| **Material** | **Search terms** |
|---|---|
| Alginate | alginic acid, alginate |
| Arabic gum | acacia, acacia gum, gum acacia, arabic gum, gum arabic |
| Bacterial nanocellulose | bacterial cellulose, bacterial nanocellulose |
| Cellulose microfibril | microfibrillar cellulose, microfibrillated cellulose, cellulose microfibril, cellulose microfiber |
| Cellulose/chitin (*) nanocrystal | * nanocrystal, nanocrystalline *, * nanowhisker, * nanorod, * crystallite |
| Cellulose/chitin (*) nanofibril | nanofibrillar *, nanofibrillated *, * nanofibril, * nanofiber |
| Collagen | collagen, gelatin |
| Guar gum | guaran, guar gum |
| Hyaluronan | hyaluronic acid, hyaluronan |
| Polylactide | polylactic acid, polylactide |
| Silk protein | sericin, fibroin, silk protein |
| Soy protein | soy bean protein, soy protein |
| Xanthan gum | xanthan gum, xanthan |

**Table S2**: References used to define the material mechanical property regions in Figure 21 of the main text.

| Category | Material | References |
|---|---|---|
| Fibers | Amyloid: *lysozyme*, *β-lactoglobulin* | (*1–3*) |
| | Bacterial nanocellulose (BNC) | (*4–6*) |
| | Bovine serum albumin (BSA) | (*7–9*) |
| | Carbon nanotube (CNT) | (*10–17*) |
| | Cellulose (regenerated) | (*18–22*) |
| | Cellulose nanocrystal (CNC) | (*23*) |
| | Cellulose nanofibril (CNF) | (*24–36*) |
| | Chitin nanofibril (ChNF) | (*24*, *27*, *29*) |
| | Chitin/Chitosan | (*37–41*) |
| | Collagen | (*9*, *42–48*) |
| | Gelatin | (*49–51*) |
| | Natural silk fibers: *spider silk, silkworm cocoon, mussel byssus, lacewing egg stalk, glow-worm capture thread* | (*52–59*) |
| | Regenerated silk proteins | (*60–70*) |
| | Soy protein | (*71*) |
| Films | Bacterial nanocellulose (BNC) | (*4*, *72–75*) |
| | Carbon nanotube (CNT) | (*76–80*) |
| | Cellulose (regenerated) | (*81–85*) |
| | Cellulose nanocrystal (CNC) | (*72*, *73*, *86–90*) |
| | Cellulose nanofibril (CNF) | (*91–98*) |
| | Chitin nanocrystal (ChNC) | (*99*) |
| | Chitin nanofibril (ChNF) | (*99–106*) |
| | Chitin/Chitosan | (*107–111*) |
| | Filter paper | (*112*) |
| | Natural gums: *carrageenan, alginates, gum arabic, guar gum, locust bean gum, tara gum, babul gum* | (*113–131*) |
| | Pectin | (*132–138*) |
| | Proteins: *collagen, gelatin, keratin, whey proteins, soy protein* | (*110*, *139–159*) |
| | Starches: *corn, cassava, potato, wheat* | (*111*, *160–166*) |
| Individual | Amyloid fibril | (*167–170*) |
| | Cellulose nanocrystal | (*171–174*) |
| | Cellulose nanofibril | (*174*, *175*) |
| | Chitin nanofibril | (*176–179*) |
| | Cotton linter | (*180*) |
| | Multi-walled carbon nanotube (MWCNT) | (*181*) |
| | Plant fiber: *ramie, fir, kenaf, bamboo, pine* | (*182–184*) |
| | Single-walled carbon nanotube (SWCNT) | (*185*, *186*) |

## References


1. C. Meier, M. E. Welland, Wet-spinning of amyloid protein nanofibers into multifunctional high-performance biofibers. *Biomacromolecules*. **12**, 3453–3459 (2011).

2. A. Kamada, A. Levin, Z. Toprakcioglu, Y. Shen, V. Lutz-Bueno, K. N. Baumann, P. Mohammadi, M. B. Linder, R. Mezzenga, T. P. J. Knowles, Modulating the Mechanical Performance of Macroscale Fibers through Shear-Induced Alignment and Assembly of Protein Nanofibrils. *Small*. **16** (2020), doi:10.1002/smll.201904190.

3. A. Kamada, N. Mittal, L. D. Söderberg, T. Ingverud, W. Ohm, S. V. Roth, F. Lundell, C. Lendel, Flow-Assisted assembly of nanostructured protein microfibers. *Proc. Natl. Acad. Sci. U. S. A.* **114**, 1232–1237 (2017).

4. Z. Wu, S. Chen, R. Wu, N. Sheng, M. Zhang, P. Ji, H. Wang, Top-down peeling bacterial cellulose to high strength ultrathin films and multifunctional fibers. *Chem. Eng. J.* **391**, 123527 (2020).

5. S. Wang, F. Jiang, X. Xu, Y. Kuang, K. Fu, E. Hitz, L. Hu, Super-Strong, Super-Stiff Macrofibers with Aligned, Long Bacterial Cellulose Nanofibers. *Adv. Mater.* **29**, 1702498 (2017).

6. J. Yao, S. Chen, Y. Chen, B. Wang, Q. Pei, H. Wang, Macrofibers with High Mechanical Performance Based on Aligned Bacterial Cellulose Nanofibers. *ACS Appl. Mater. Interfaces*. **9**, 20330–20339 (2017).

7. Y. Dror, T. Ziv, V. Makarov, H. Wolf, A. Admon, E. Zussman, Nanofibers made of globular proteins. *Biomacromolecules*. **9**, 2749–2754 (2008).

8. H. He, C. Yang, F. Wang, Z. Wei, J. Shen, D. Chen, C. Fan, H. Zhang, K. Liu, Mechanically Strong Globular-Protein-Based Fibers Obtained Using a Microfluidic Spinning Technique. *Angew. Chemie - Int. Ed.* **59**, 4344–4348 (2020).

9. J. Zhang, J. Sun, B. Li, C. Yang, J. Shen, N. Wang, R. Gu, D. Wang, D. Chen, H. Hu, C. Fan, H. Zhang, K. Liu, Robust Biological Fibers Based on Widely Available Proteins: Facile Fabrication and Suturing Application. *Small*. **16**, 1907598 (2020).

10. K. Koziol, J. Vilatela, A. Moisala, M. Motta, P. Cunniff, M. Sennett, A. Windle, High-performance carbon nanotube fiber. *Science*. **318**, 1892–1895 (2007).

11. K. Liu, Y. Sun, R. Zhou, H. Zhu, J. Wang, L. Liu, S. Fan, K. Jiang, Carbon nanotube yarns with high tensile strength made by a twisting and shrinking method. *Nanotechnology*. **21**, 045708 (2010).

12. A. Ghemes, Y. Minami, J. Muramatsu, M. Okada, H. Mimura, Y. Inoue, Fabrication and mechanical properties of carbon nanotube yarns spun from ultra-long multi-walled carbon nanotube arrays. *Carbon N. Y.* **50**, 4579–4587 (2012).

13. N. Behabtu, C. C. Young, D. E. Tsentalovich, O. Kleinerman, X. Wang, A. W. K. Ma, E. A. Bengio, R. F. Ter Waarbeek, J. J. De Jong, R. E. Hoogerwerf, S. B. Fairchild, J. B. Ferguson, B. Maruyama, J. Kono, Y. Talmon, Y. Cohen, M. J. Otto, M. Pasquali, Strong, light, multifunctional fibers of carbon nanotubes with ultrahigh conductivity. *Science*. **339**, 182–186 (2013).

14. K. Sugano, M. Kurata, H. Kawada, Evaluation of mechanical properties of untwisted carbon nanotube yarn for application to composite materials. *Carbon N. Y.* **78**, 356–365 (2014).

15. C. Jiang, A. Saha, C. C. Young, D. P. Hashim, C. E. Ramirez, P. M. Ajayan, M. Pasquali, A. A. Martí, Macroscopic Nanotube Fibers Spun from Single-Walled Carbon Nanotube Polyelectrolytes. *ACS Nano*. **8**, 9107–9112 (2014).

16. J. N. Wang, X. G. Luo, T. Wu, Y. Chen, High-strength carbon nanotube fibre-like ribbon with high ductility and high electrical conductivity. *Nat. Commun.* **5**, 3848 (2014).

17. T. Q. Tran, Z. Fan, P. Liu, S. M. Myint, H. M. Duong, Super-strong and highly conductive carbon nanotube ribbons from post-treatment methods. *Carbon N. Y.* **99**, 407–415 (2016).

18. R. B. Adusumali, M. Reifferscheid, H. Weber, T. Roeder, H. Sixta, W. Gindl, in *Macromolecular Symposia* (2006), vol. 244, pp. 119–125.

19. L. K. J. Hauru, M. Hummel, A. Michud, H. Sixta, Dry jet-wet spinning of strong cellulose filaments from ionic liquid solution. *Cellulose*. **21**, 4471–4481 (2014).

20. H. Sixta, A. Michud, L. Hauru, S. Asaadi, Y. Ma, A. W. T. King, I. Kilpeläinen, M. Hummel, Ioncell-F: A high-strength regenerated cellulose fibre. *Nord. Pulp Pap. Res. J.* **30**, 43–57 (2015).

21. S. Asaadi, T. Kakko, A. W. T. King, I. Kilpeläinen, M. Hummel, H. Sixta, High-Performance Acetylated Ioncell-F Fibers with Low Degree of Substitution. *ACS Sustain. Chem. Eng.* **6**, 9418–9426 (2018).

22. J. Zhang, H. Kitayama, Y. Gotoh, High strength ultrafine cellulose fibers generated by solution blow spinning. *Eur. Polym. J.* **125**, 109513 (2020).

23. J. Araki, M. Miyayama, Wet spinning of cellulose nanowhiskers; fiber yarns obtained only from colloidal cellulose crystals. *Polymer (Guildf).* **188**, 122116 (2020).

24. A. Walther, J. V. I. Timonen, I. Díez, A. Laukkanen, O. Ikkala, Multifunctional high-performance biofibers based on wet-extrusion of renewable native cellulose nanofibrils. *Adv. Mater.* **23**, 2924–2928 (2011).

25. S. Iwamoto, A. Isogai, T. Iwata, Structure and mechanical properties of wet-spun fibers made from natural cellulose nanofibers. *Biomacromolecules*. **12**, 831–836 (2011).

26. N. Mittal, F. Ansari, V. Gowda Krishne, C. Brouzet, P. Chen, P. T. Larsson, S. V. Roth, F. Lundell, L. Wågberg, N. A. Kotov, L. D. Söderberg, Multiscale Control of Nanocellulose Assembly: Transferring Remarkable Nanoscale Fibril Mechanics to Macroscale Fibers. *ACS Nano*. **12**, 6378–6388 (2018).

27. H. C. Kim, D. Kim, J. Y. Lee, L. Zhai, J. Kim, Effect of Wet Spinning and Stretching to Enhance Mechanical Properties of Cellulose Nanofiber Filament. *Int. J. Precis. Eng. Manuf. - Green Technol.* **6**, 567–575 (2019).

28. H. G. Wise, H. Takana, F. Ohuchi, A. B. Dichiara, Field-Assisted Alignment of Cellulose Nanofibrils in a Continuous Flow-Focusing System. *ACS Appl. Mater. Interfaces*. **12**, 28568–28575 (2020).

29. J. G. Torres-Rendon, F. H. Schacher, S. Ifuku, A. Walther, Mechanical performance of macrofibers of cellulose and chitin nanofibrils aligned by wet-stretching: A critical comparison. *Biomacromolecules*. **15**, 2709–2717 (2014).

30. K. M. O. Håkansson, A. B. Fall, F. Lundell, S. Yu, C. Krywka, S. V. Roth, G. Santoro, M. Kvick, L. Prahl Wittberg, L. Wågberg, L. D. Söderberg, Hydrodynamic alignment and assembly of nanofibrils resulting in strong cellulose filaments. *Nat. Commun.* **5**, 4018 (2014).

31. S. Hooshmand, Y. Aitomäki, N. Norberg, A. P. Mathew, K. Oksman, Dry-Spun Single-Filament Fibers Comprising Solely Cellulose Nanofibers from Bioresidue. *ACS Appl. Mater. Interfaces*. **7**, 13022–13028 (2015).

32. M. J. Lundahl, A. G. Cunha, E. Rojo, A. C. Papageorgiou, L. Rautkari, J. C. Arboleda, O. J. Rojas, Strength and Water Interactions of Cellulose i Filaments Wet-Spun from Cellulose Nanofibril Hydrogels. *Sci. Rep.* **6**, 30695 (2016).

33. L. Geng, B. Chen, X. Peng, T. Kuang, Strength and modulus improvement of wet-spun cellulose I filaments by sequential physical and chemical cross-linking. *Mater. Des.* **136**, 45–53 (2017).

34. H. Mertaniemi, C. Escobedo-Lucea, A. Sanz-Garcia, C. Gandía, A. Mäkitie, J. Partanen, O. Ikkala, M. Yliperttula, Human stem cell decorated nanocellulose threads for biomedical applications.

*Biomaterials*. **82**, 208–220 (2016).

35. S. Hooshmand, Y. Aitomäki, L. Berglund, A. P. Mathew, K. Oksman, Enhanced alignment and mechanical properties through the use of hydroxyethyl cellulose in solvent-free native cellulose spun filaments. *Compos. Sci. Technol.* **150**, 79–86 (2017).

36. P. Mohammadi, M. S. Toivonen, O. Ikkala, W. Wagermaier, M. B. Linder, Aligning cellulose nanofibril dispersions for tougher fibers. *Sci. Rep.* **7**, 11860 (2017).

37. Y. Qin, X. Lu, N. Sun, R. D. Rogers, Dissolution or extraction of crustacean shells using ionic liquids to obtain high molecular weight purified chitin and direct production of chitin films and fibers. *Green Chem.* **12**, 968–97 (2010).

38. K. Zhu, H. Tu, P. Yang, C. Qiu, D. Zhang, A. Lu, L. Luo, F. Chen, X. Liu, L. Chen, Q. Fu, L. Zhang, Mechanically Strong Chitin Fibers with Nanofibril Structure, Biocompatibility, and Biodegradability. *Chem. Mater.* **31**, 2078–2087 (2019).

39. J. L. Shamshina, O. Zavgorodnya, P. Berton, P. K. Chhotaray, H. Choudhary, R. D. Rogers, Ionic Liquid Platform for Spinning Composite Chitin-Poly(lactic acid) Fibers. *ACS Sustain. Chem. Eng.* **6**, 10241–10251 (2018).

40. E. N. Dresvyanina, I. P. Dobrovol'skaya, P. V. Popryadukhin, V. E. Yudin, E. M. Ivan'kova, V. Y. Elokhovskii, A. Y. Khomenko, Influence of spinning conditions on properties of chitosan fibers. *Fibre Chem.* **44**, 280–283 (2013).

41. L. Notin, C. Viton, L. David, P. Alcouffe, C. Rochas, A. Domard, Morphology and mechanical properties of chitosan fibers obtained by gel-spinning: Influence of the dry-jet-stretching step and ageing. *Acta Biomater.* **2**, 387–402 (2006).

42. D. I. Zeugolis, R. G. Paul, G. Attenburrow, Engineering extruded collagen fibers for biomedical applications. *J. Appl. Polym. Sci.* **108**, 2886–2894 (2008).

43. R. Tonndorf, D. Aibibu, C. Cherif, Collagen multifilament spinning. *Mater. Sci. Eng. C*. **106** (2020), doi:10.1016/j.msec.2019.110105.

44. C. Haynl, E. Hofmann, K. Pawar, S. Förster, T. Scheibel, Microfluidics-Produced Collagen Fibers Show Extraordinary Mechanical Properties. *Nano Lett.* **16**, 5917–5922 (2016).

45. M. L. Siriwardane, K. Derosa, G. Collins, B. J. Pfister, Controlled formation of cross-linked collagen fibers for neural tissue engineering applications. *Biofabrication*. **6**, 015012 (2014).

46. J. M. Caves, V. A. Kumar, J. Wen, W. Cui, A. Martinez, R. Apkarian, J. E. Coats, K. Berland, E. L. Chaikof, Fibrillogenesis in continuously spun synthetic collagen fiber. *J. Biomed. Mater. Res. - Part B Appl. Biomater.* **93**, 24–38 (2010).

47. D. I. Zeugolis, R. G. Paul, G. Attenburrow, Post-self-assembly experimentation on extruded collagen fibres for tissue engineering applications. *Acta Biomater.* **4**, 1646–1656 (2008).

48. D. I. Zeugolis, R. G. Paul, G. Attenburrow, Factors influencing the properties of reconstituted collagen fibers prior to self-assembly: Animal species and collagen extraction method. *J. Biomed. Mater. Res. - Part A*. **86**, 892–904 (2008).

49. R. Fukae, A. Maekawa, O. Sangen, Gel-spinning and drawing of gelatin. *Polymer (Guildf).* **46**, 11193–11194 (2005).

50. P. R. Stoessel, R. A. Raso, T. Kaufmann, R. N. Grass, W. J. Stark, Fibers Mechanically Similar to Sheep Wool Obtained by Wet Spinning of Gelatin and Optional Plasticizers. *Macromol. Mater. Eng.* **300**, 234–241 (2015).

51. R. Fukae, T. Midorikawa, Preparation of gelatin fiber by gel spinning and its mechanical properties. *J. Appl. Polym. Sci.* **110**, 4011–4015 (2008).

52. J. E. Smeathers, J. F. V. Vincent, Mechanical Properties of Mussel Byssus Threads. *J. Molluscan Stud.* **45**, 219–230 (1979).

53. D. Piorkowski, T. A. Blackledge, C. P. Liao, N. E. Doran, C. L. Wu, S. J. Blamires, I. M. Tso, Humidity-dependent mechanical and adhesive properties of Arachnocampa tasmaniensis capture threads. *J. Zool.* **305**, 256–266 (2018).

54. F. Bauer, L. Bertinetti, A. Masic, T. Scheibel, Dependence of mechanical properties of lacewing egg stalks on relative humidity. *Biomacromolecules*. **13**, 3730–3735 (2012).

55. O. P. Troncoso, F. G. Torres, C. J. Grande, Characterization of the mechanical properties of tough biopolymer fibres from the mussel byssus of Aulacomya ater. *Acta Biomater.* **4**, 1114–1117 (2008).

56. J. Pérez-Rigueiro, C. Viney, J. Llorca, M. Elices, Mechanical properties of single-brin silkworm silk. *J. Appl. Polym. Sci.* **75**, 1270–1277 (2000).

57. F. Vollrath, B. Madsen, Z. Shao, The effect of spinning conditions on the mechanics of a spider's dragline silk. *Proc. R. Soc. B Biol. Sci.* **268**, 2339–2346 (2001).

58. G. V. Guinea, M. Elices, G. R. Plaza, G. B. Perea, R. Daza, C. Riekel, F. Agulló-Rueda, C. Hayashi, Y. Zhao, J. Pérez-Rigueiro, Minor ampullate silks from Nephila and Argiope spiders: Tensile properties and microstructural characterization. *Biomacromolecules*. **13**, 2087–2098 (2012).

59. C. Y. Hayashi, T. A. Blackledge, R. V. Lewis, Molecular and mechanical characterization of aciniform silk: Uniformity of iterated sequence modules in a novel member of the spider silk fibroin gene family. *Mol. Biol. Evol.* **21**, 1950–1959 (2004).

60. L. Lu, S. Fan, Q. Niu, Q. Peng, L. Geng, G. Yang, H. Shao, B. S. Hsiao, Y. Zhang, Strong Silk Fibers Containing Cellulose Nanofibers Generated by a Bioinspired Microfluidic Chip. *ACS Sustain. Chem. Eng.* **7**, 14765–14774 (2019).

61. R. Madurga, A. M. Gañán-Calvo, G. R. Plaza, G. V. Guinea, M. Elices, J. Pérez-Rigueiro, Straining flow spinning: Production of regenerated silk fibers under a wide range of mild coagulating chemistries. *Green Chem.* **19**, 3380–3389 (2017).

62. S. W. Ha, A. E. Tonelli, S. M. Hudson, Structural studies of Bombyx mori silk fibroin during regeneration from solutions and wet fiber spinning. *Biomacromolecules*. **6**, 1722–1731 (2005).

63. S. Ling, Z. Qin, C. Li, W. Huang, D. L. Kaplan, M. J. Buehler, Polymorphic regenerated silk fibers assembled through bioinspired spinning. *Nat. Commun.* **8**, 1387 (2017).

64. Z. Chen, H. Zhang, Z. Lin, Y. Lin, J. H. van Esch, X. Y. Liu, Programing Performance of Silk Fibroin Materials by Controlled Nucleation. *Adv. Funct. Mater.* **26**, 8978–8990 (2016).

65. G. Fang, Y. Huang, Y. Tang, Z. Qi, J. Yao, Z. Shao, X. Chen, Insights into Silk Formation Process: Correlation of Mechanical Properties and Structural Evolution during Artificial Spinning of Silk Fibers. *ACS Biomater. Sci. Eng.* **2**, 1992–2000 (2016).

66. A. Heidebrecht, L. Eisoldt, J. Diehl, A. Schmidt, M. Geffers, G. Lang, T. Scheibel, Biomimetic Fibers Made of Recombinant Spidroins with the Same Toughness as Natural Spider Silk. *Adv. Mater.* **27**, 2189–2194 (2015).

67. J. Luo, L. Zhang, Q. Peng, M. Sun, Y. Zhang, H. Shao, X. Hu, Tough silk fibers prepared in air using a biomimetic microfluidic chip. *Int. J. Biol. Macromol.* **66**, 319–324 (2014).

68. J. Yan, G. Zhou, D. P. Knight, Z. Shao, X. Chen, Wet-spinning of regenerated silk fiber from aqueous silk fibroin solution: Discussion of spinning parameters. *Biomacromolecules*. **11**, 1–5 (2010).

69. G. Zhou, Z. Shao, D. P. Knight, J. Yan, X. Chen, Silk fibers extruded artificially from aqueous solutions of regenerated bombyx mori silk fibroin are tougher than their natural counterparts. *Adv. Mater.* **21**, 366–370 (2009).

70. P. Corsini, J. Perez-Rigueiro, G. V. Guinea, G. R. Plaza, M. Elices, E. Marsano, M. M. Carnasciali, G. Freddi, Influence of the draw ratio on the tensile and fracture behavior of NMMO regenerated silk fibers. *J. Polym. Sci. Part B Polym. Phys.* **45**, 2568–2579 (2007).

71. N. Reddy, Y. Yang, Soyprotein fibers with high strength and water stability for potential medical applications. *Biotechnol. Prog.* **25**, 1796–1802 (2009).

72. Z. Li, X. Li, J. Ren, B. Wu, Q. Luo, X. Liu, C. Pei, Robust All-Cellulose Nanofiber Composite from Stack-Up Bacterial Cellulose Hydrogels via Self-Aggregation Forces. *J. Agric. Food Chem.* **68**, 2696–2701 (2020).

73. S. Wang, T. Li, C. Chen, W. Kong, S. Zhu, J. Dai, A. J. Diaz, E. Hitz, S. D. Solares, T. Li, L. Hu, Transparent, Anisotropic Biofilm with Aligned Bacterial Cellulose Nanofibers. *Adv. Funct. Mater.* **28**, 1707491 (2018).

74. L. Rozenberga, M. Skute, L. Belkova, I. Sable, L. Vikele, P. Semjonovs, M. Saka, M. Ruklisha, L. Paegle, Characterisation of films and nanopaper obtained from cellulose synthesised by acetic acid bacteria. *Carbohydr. Polym.* **144**, 33–40 (2016).

75. M. M. Rahman, A. N. Netravali, Aligned Bacterial Cellulose Arrays as "green" Nanofibers for Composite Materials. *ACS Macro Lett.* **5**, 1070–1074 (2016).

76. X. Yu, X. Zhang, J. Zou, Z. Lan, C. Jiang, J. Zhao, D. Zhang, M. Miao, Q. Li, Solvent-Tunable Microstructures of Aligned Carbon Nanotube Films. *Adv. Mater. Interfaces*. **3**, 1600352 (2016).

77. S. Qu, X. Jiang, Q. Li, L. Gao, G. Zhou, D. Zhang, W. Gong, W. Lu, Developing strong and tough carbon nanotube films by proper dispersing strategy and enhanced interfacial interactions. *Carbon N. Y.* **149**, 117–124 (2019).

78. Q. Liu, M. Li, Y. Gu, Y. Zhang, S. Wang, Q. Li, Z. Zhang, Highly aligned dense carbon nanotube sheets induced by multiple stretching and pressing. *Nanoscale*. **6**, 4338–4344 (2014).

79. Z. Shi, X. Chen, X. Wang, T. Zhang, J. Jin, Fabrication of superstrong ultrathin free-standing single-walled carbon nanotube films via a wet process. *Adv. Funct. Mater.* **21**, 4358–4363 (2011).

80. J. L. Rigueur, S. A. Hasan, S. V. Mahajan, J. H. Dickerson, Buckypaper fabrication by liberation of electrophoretically deposited carbon nanotubes. *Carbon N. Y.* **48**, 4090–4099 (2010).

81. Q. Yang, S. Fujisawa, T. Saito, A. Isogai, Improvement of mechanical and oxygen barrier properties of cellulose films by controlling drying conditions of regenerated cellulose hydrogels. *Cellulose*. **19**, 695–703 (2012).

82. N. Hameed, Q. Guo, Blend films of natural wool and cellulose prepared from an ionic liquid. *Cellulose*. **17**, 803–813 (2010).

83. S. Mahmoudian, M. U. Wahit, A. F. Ismail, A. A. Yussuf, Preparation of regenerated cellulose/montmorillonite nanocomposite films via ionic liquids. *Carbohydr. Polym.* **88**, 1251–1257 (2012).

84. R. De Silva, X. Wang, N. Byrne, Tri-component bio-composite materials prepared using an eco-friendly processing route. *Cellulose*. **20**, 2461–2468 (2013).

85. R. De Silva, K. Vongsanga, X. Wang, N. Byrne, Development of a novel regenerated cellulose composite material. *Carbohydr. Polym.* **121**, 382–387 (2015).

86. Z. Ling, K. Wang, W. Liu, W. Tang, Q. Yong, Tuning the cellulose nanocrystal alignments for supramolecular assembly of chiral nematic films with highly efficient UVB shielding capability. *J. Mater. Chem. C*. **8**, 8493–8501 (2020).

87. C. M. Walters, C. E. Boott, T. D. Nguyen, W. Y. Hamad, M. J. Maclachlan, Iridescent cellulose nanocrystal films modified with hydroxypropyl cellulose. *Biomacromolecules*. **21**, 1295–1302 (2020).

88. R. Bardet, N. Belgacem, J. Bras, Flexibility and color monitoring of cellulose nanocrystal iridescent solid films using anionic or neutral polymers. *ACS Appl. Mater. Interfaces*. **7**, 4010–4018 (2015).

89. J. M. Passantino, A. D. Haywood, J. Goswami, V. A. Davis, Effects of Polymer Additives and Dispersion State on the Mechanical Properties of Cellulose Nanocrystal Films. *Macromol. Mater. Eng.* **302**, 1600351 (2017).

90. A. B. Reising, R. J. Moon, J. P. Youngblood, Effect of particle alignment on mechanical properties of neat cellulose nanocrystal films. *J-for*. **2**, 32–41 (2012).

91. K. Kriechbaum, L. Bergström, Antioxidant and UV-Blocking Leather-Inspired Nanocellulose-Based Films with High Wet Strength. *Biomacromolecules*. **21**, 1720–1728 (2020).

92. F. Lossada, J. Guo, D. Jiao, S. Groeer, E. Bourgeat-Lami, D. Montarnal, A. Walther, Vitrimer Chemistry Meets Cellulose Nanofibrils: Bioinspired Nanopapers with High Water Resistance and Strong Adhesion. *Biomacromolecules*. **20**, 1045–1055 (2019).

93. P. R. Sharma, B. Zheng, S. K. Sharma, C. Zhan, R. Wang, S. R. Bhatia, B. S. Hsiao, High Aspect Ratio Carboxycellulose Nanofibers Prepared by Nitro-Oxidation Method and Their Nanopaper Properties. *ACS Appl. Nano Mater.* **1**, 3969–3980 (2018).

94. M. Österberg, J. Vartiainen, J. Lucenius, U. Hippi, J. Seppälä, R. Serimaa, J. Laine, A fast method to produce strong NFC films as a platform for barrier and functional materials. *ACS Appl. Mater. Interfaces*. **5**, 4640–4647 (2013).

95. N. Pahimanolis, A. Salminen, P. A. Penttilä, J. T. Korhonen, L. S. Johansson, J. Ruokolainen, R. Serimaa, J. Seppälä, Nanofibrillated cellulose/carboxymethyl cellulose composite with improved wet strength. *Cellulose*. **20**, 1459–1468 (2013).

96. A. J. Benítez, J. Torres-Rendon, M. Poutanen, A. Walther, Humidity and multiscale structure govern mechanical properties and deformation modes in films of native cellulose nanofibrils. *Biomacromolecules*. **14**, 4497–4506 (2013).

97. H. Sehaqui, N. Ezekiel Mushi, S. Morimune, M. Salajkova, T. Nishino, L. A. Berglund, Cellulose nanofiber orientation in nanopaper and nanocomposites by cold drawing. *ACS Appl. Mater. Interfaces*. **4**, 1043–1049 (2012).

98. H. Sehaqui, A. Liu, Q. Zhou, L. A. Berglund, Fast preparation procedure for large, flat cellulose and cellulose/inorganic nanopaper structures. *Biomacromolecules*. **11**, 2195–2198 (2010).

99. Y. Fan, H. Fukuzumi, T. Saito, A. Isogai, Comparative characterization of aqueous dispersions and cast films of different chitin nanowhiskers/nanofibers. *Int. J. Biol. Macromol.* **50**, 69–76 (2012).

100. P. Hassanzadeh, W. Sun, J. P. De Silva, J. Jin, K. Makhnejia, G. L. W. Cross, M. Rolandi, Mechanical properties of self-assembled chitin nanofiber networks. *J. Mater. Chem. B*. **2**, 2461–2466 (2014).

101. T. Naghdi, H. Golmohammadi, H. Yousefi, M. Hosseinifard, U. Kostiv, D. Horák, A. Merkoçi, Chitin Nanofiber Paper toward Optical (Bio)sensing Applications. *ACS Appl. Mater. Interfaces*. **12**, 15538–15552 (2020).

102. Q. Wu, N. E. Mushi, L. A. Berglund, High-Strength Nanostructured Films Based on Well-Preserved α-Chitin Nanofibrils Disintegrated from Insect Cuticles. *Biomacromolecules*. **21**, 604–612 (2020).

103. W. M. Fazli Wan Nawawi, K. Y. Lee, E. Kontturi, R. J. Murphy, A. Bismarck, Chitin Nanopaper from Mushroom Extract: Natural Composite of Nanofibers and Glucan from a Single Biobased Source. *ACS Sustain. Chem. Eng.* **7**, 6492–6496 (2019).

104. F. Riehle, D. Hoenders, J. Guo, A. Eckert, S. Ifuku, A. Walther, Sustainable Chitin Nanofibrils Provide Outstanding Flame-Retardant Nanopapers. *Biomacromolecules*. **20**, 1098–1108 (2019).

105. S. Ifuku, S. Morooka, A. N. Nakagaito, M. Morimoto, H. Saimoto, Preparation and characterization of

optically transparent chitin nanofiber/(Meth)acrylic resin composites. *Green Chem.* **13**, 1708–1711 (2011).

106. N. E. Mushi, T. Nishino, L. A. Berglund, Q. Zhou, Strong and tough chitin film from α-chitin nanofibers prepared by high pressure homogenization and chitosan addition. *ACS Sustain. Chem. Eng.* **7**, 1692–1697 (2019).

107. J. Huang, Y. Zhong, L. Zhang, J. Cai, Extremely Strong and Transparent Chitin Films: A High-Efficiency, Energy-Saving, and "Green" Route Using an Aqueous KOH/Urea Solution. *Adv. Funct. Mater.* **27**, 1701100 (2017).

108. B. Duan, C. Chang, B. Ding, J. Cai, M. Xu, S. Feng, J. Ren, X. Shi, Y. Du, L. Zhang, High strength films with gas-barrier fabricated from chitin solution dissolved at low temperature. *J. Mater. Chem. A.* **1**, 1867–1874 (2013).

109. K. Zhu, S. Shi, Y. Cao, A. Lu, J. Hu, L. Zhang, Robust chitin films with good biocompatibility and breathable properties. *Carbohydr. Polym.* **212**, 361–367 (2019).

110. S. Fakhreddin Hosseini, M. Rezaei, M. Zandi, F. F. Ghavi, Preparation and functional properties of fish gelatin-chitosan blend edible films. *Food Chem.* **136**, 1490–1495 (2013).

111. J. Bonilla, L. Atarés, M. Vargas, A. Chiralt, Properties of wheat starch film-forming dispersions and films as affected by chitosan addition. *J. Food Eng.* **114**, 303–312 (2013).

112. W. Zhang, Z. Jing, Y. Shan, X. Ge, X. Mu, Y. Jiang, H. Li, P. Wu, Paper reinforced with regenerated cellulose: A sustainable and fascinating material with good mechanical performance, barrier properties and shape retention in water. *J. Mater. Chem. A.* **4**, 17483–17490 (2016).

113. P. Kanmani, J. W. Rhim, Development and characterization of carrageenan/grapefruit seed extract composite films for active packaging. *Int. J. Biol. Macromol.* **68**, 258–266 (2014).

114. S. Shankar, J. P. Reddy, J. W. Rhim, H. Y. Kim, Preparation, characterization, and antimicrobial activity of chitin nanofibrils reinforced carrageenan nanocomposite films. *Carbohydr. Polym.* **117**, 468–475 (2015).

115. S. Gopi, A. Amalraj, N. Kalarikkal, J. Zhang, S. Thomas, Q. Guo, Preparation and characterization of nanocomposite films based on gum arabic, maltodextrin and polyethylene glycol reinforced with turmeric nanofiber isolated from turmeric spent. *Mater. Sci. Eng. C.* **97**, 723–729 (2019).

116. K. Łupina, D. Kowalczyk, E. Zięba, W. Kazimierczak, M. Mężyńska, M. Basiura-Cembala, A. E. Wiącek, Edible films made from blends of gelatin and polysaccharide-based emulsifiers - A comparative study. *Food Hydrocoll.* **96**, 555–567 (2019).

117. P. Zhang, Y. Zhao, Q. Shi, Characterization of a novel edible film based on gum ghatti: Effect of plasticizer type and concentration. *Carbohydr. Polym.* **153**, 345–355 (2016).

118. M. Rose Joseph, N. P.A, G. S., H. J. Maria, V. R, N. Kalarikkal, S. Thomas, V. K, A. E.V, Development and characterization of cellulose nanofibre reinforced Acacia nilotica gum nanocomposite. *Ind. Crops Prod.* **161**, 113180 (2021).

119. C. K. Saurabh, S. Gupta, J. Bahadur, S. Mazumder, P. S. Variyar, A. Sharma, Mechanical and barrier properties of guar gum based nano-composite films. *Carbohydr. Polym.* **124**, 77–84 (2015).

120. J. Tripathi, R. Ambolikar, S. Gupta, D. Jain, J. Bahadur, P. S. Variyar, Methylation of guar gum for improving mechanical and barrier properties of biodegradable packaging films. *Sci. Rep.* **9**, 1–9 (2019).

121. F. Liu, W. Chang, M. Chen, F. Xu, J. Ma, F. Zhong, Film-forming properties of guar gum, tara gum and locust bean gum. *Food Hydrocoll.* **98**, 105007 (2020).

122. A. Aydogdu, C. J. Radke, S. Bezci, E. Kirtil, Characterization of curcumin incorporated guar gum/orange oil antimicrobial emulsion films. *Int. J. Biol. Macromol.* **148**, 110–120 (2020).

123. A. Kurt, O. S. Toker, F. Tornuk, Effect of xanthan and locust bean gum synergistic interaction on characteristics of biodegradable edible film. *Int. J. Biol. Macromol.* **102**, 1035–1044 (2017).

124. G. A. Paula, N. M. B. Benevides, A. P. Cunha, A. V. de Oliveira, A. M. B. Pinto, J. P. S. Morais, H. M. C. Azeredo, Development and characterization of edible films from mixtures ofκ-carrageenan, I-carrageenan, and alginate. *Food Hydrocoll.* **47**, 140–145 (2015).

125. A. A. Oun, J. W. Rhim, Carrageenan-based hydrogels and films: Effect of ZnO and CuO nanoparticles on the physical, mechanical, and antimicrobial properties. *Food Hydrocoll.* **67**, 45–53 (2017).

126. Z. Kassab, F. Aziz, H. Hannache, H. Ben Youcef, M. El Achaby, Improved mechanical properties of k-carrageenan-based nanocomposite films reinforced with cellulose nanocrystals. *Int. J. Biol. Macromol.* **123**, 1248–1256 (2019).

127. T. R. Martiny, V. Raghavan, C. C. de Moraes, G. S. da Rosa, G. L. Dotto, Bio-Based Active Packaging: Carrageenan Film with Olive Leaf Extract for Lamb Meat Preservation. *Foods.* **9**, 1759 (2020).

128. M. Ionita, M. A. Pandele, H. Iovu, Sodium alginate/graphene oxide composite films with enhanced thermal and mechanical properties. *Carbohydr. Polym.* **94**, 339–344 (2013).

129. M. J. Costa, A. M. Marques, L. M. Pastrana, J. A. Teixeira, S. M. Sillankorva, M. A. Cerqueira, Physicochemical properties of alginate-based films: Effect of ionic crosslinking and mannuronic and guluronic acid ratio. *Food Hydrocoll.* **81**, 442–448 (2018).

130. A. S. Giz, M. Berberoglu, S. Bener, S. Aydelik-Ayazoglu, H. Bayraktar, B. E. Alaca, H. Catalgil-Giz, A detailed investigation of the effect of calcium crosslinking and glycerol plasticizing on the physical properties of alginate films. *Int. J. Biol. Macromol.* **148**, 49–55 (2020).

131. S. Roy, J. W. Rhim, Effect of CuS reinforcement on the mechanical, water vapor barrier, UV-light barrier, and antibacterial properties of alginate-based composite films. *Int. J. Biol. Macromol.* **164**, 37–44 (2020).

132. T. Giancone, E. Torrieri, P. Di Pierro, S. Cavella, C. V. L. Giosafatto, P. Masi, Effect of Surface Density on the Engineering Properties of High Methoxyl Pectin-Based Edible Films. *Food Bioprocess Technol.* **4**, 1228–1236 (2011).

133. V. Siracusa, S. Romani, M. Gigli, C. Mannozzi, J. P. Cecchini, U. Tylewicz, N. Lotti, Characterization of active edible films based on citral essential oil, alginate and pectin. *Materials (Basel).* **11** (2018), doi:10.3390/ma11101980.

134. H. G. R. Younis, H. R. S. Abdellatif, F. Ye, G. Zhao, Tuning the physicochemical properties of apple pectin films by incorporating chitosan/pectin fiber. *Int. J. Biol. Macromol.* **159**, 213–221 (2020).

135. J. Prachayawarakorn, S. Poomkaew, Property Improvement of Biodegradable High-Methoxy Pectin Film by Different Contents of Dialdehyde Starch. *J. Polym. Environ.* **28**, 2227–2235 (2020).

136. J. Meerasri, R. Sothornvit, Characterization of bioactive film from pectin incorporated with gamma-aminobutyric acid. *Int. J. Biol. Macromol.* **147**, 1285–1293 (2020).

137. P. Ezati, J. W. Rhim, pH-responsive pectin-based multifunctional films incorporated with curcumin and sulfur nanoparticles. *Carbohydr. Polym.* **230**, 115638 (2020).

138. L. B. Norcino, J. F. Mendes, C. V. L. Natarelli, A. Manrich, J. E. Oliveira, L. H. C. Mattoso, Pectin films loaded with copaiba oil nanoemulsions for potential use as bio-based active packaging. *Food Hydrocoll.* **106**, 105862 (2020).

139. R. A. Brown, M. Wiseman, C. B. Chuo, U. Cheema, S. N. Nazhat, Ultrarapid engineering of biomimetic materials and tissues: Fabrication of nano- and microstructures by plastic compression. *Adv. Funct. Mater.* **15**, 1762–1770 (2005).

140. T. Tanabe, N. Okitsu, A. Tachibana, K. Yamauchi, Preparation and characterization of keratin-chitosan

composite film. *Biomaterials*. **23**, 817–825 (2002).

141. N. Ramakrishnan, S. Sharma, A. Gupta, B. Y. Alashwal, Keratin based bioplastic film from chicken feathers and its characterization. *Int. J. Biol. Macromol.* **111**, 352–358 (2018).

142. T. Posati, A. Aluigi, A. Donnadio, G. Sotgiu, M. Mosconi, M. Muccini, G. Ruani, R. Zamboni, M. Seri, Keratin Film as Natural and Eco-Friendly Support for Organic Optoelectronic Devices. *Adv. Sustain. Syst.* **3**, 1900080 (2019).

143. M. Gambucci, A. Aluigi, M. Seri, G. Sotgiu, G. Zampini, A. Donnadio, A. Torreggiani, R. Zamboni, L. Latterini, T. Posati, Effect of Chemically Engineered Au/Ag Nanorods on the Optical and Mechanical Properties of Keratin Based Films. *Front. Chem.* **8**, 158 (2020).

144. C. Nowogrodski, I. Simon, S. Magdassi, O. Shoseyov, Fabrication of second skin from keratin and melanin. *Polymers (Basel).* **12**, 1–13 (2020).

145. Ó. L. Ramos, I. Reinas, S. I. Silva, J. C. Fernandes, M. A. Cerqueira, R. N. Pereira, A. A. Vicente, M. F. Poças, M. E. Pintado, F. X. Malcata, Effect of whey protein purity and glycerol content upon physical properties of edible films manufactured therefrom. *Food Hydrocoll.* **30**, 110–122 (2013).

146. M. Soazo, L. M. Pérez, A. C. Rubiolo, R. A. Verdini, Effect of freezing on physical properties of whey protein emulsion films. *Food Hydrocoll.* **31**, 256–263 (2013).

147. V. M. Azevedo, M. V. Dias, S. V. Borges, A. L. R. Costa, E. K. Silva, É. A. A. Medeiros, N. de F. F. Soares, Development of whey protein isolate bio-nanocomposites: Effect of montmorillonite and citric acid on structural, thermal, morphological and mechanical properties. *Food Hydrocoll.* **48**, 179–188 (2015).

148. S. Dash, S. K. Swain, Effect of nanoboron nitride on the physical and chemical properties of soy protein. *Compos. Sci. Technol.* **84**, 39–43 (2013).

149. Y. Li, H. Chen, Y. Dong, K. Li, L. Li, J. Li, Carbon nanoparticles/soy protein isolate bio-films with excellent mechanical and water barrier properties. *Ind. Crops Prod.* **82**, 133–140 (2016).

150. A. Sionkowska, M. Wisniewski, J. Skopinska, G. F. Poggi, E. Marsano, C. A. Maxwell, T. J. Wess, Thermal and mechanical properties of UV irradiated collagen/chitosan thin films. *Polym. Degrad. Stab.* **91**, 3026–3032 (2006).

151. S. Rani, A. K. Singh, R. R. Paswan, K. D. Kumar, R. Kumar, Preparation, Characterization and Antibacterial Evaluation of Soy Protein Isolate Biopolymeric Films Loaded with Nalidixic Acid. *J. Polym. Environ.* **28**, 1841–1850 (2020).

152. W. Gu, X. Liu, Q. Gao, S. Gong, J. Li, S. Q. Shi, Multiple Hydrogen Bonding Enables Strong, Tough, and Recyclable Soy Protein Films. *ACS Sustain. Chem. Eng.* **8**, 7680–7689 (2020).

153. V. A. Kumar, J. M. Caves, C. A. Haller, E. Dai, L. Liu, S. Grainger, E. L. Chaikof, Collagen-based substrates with tunable strength for soft tissue engineering. *Biomater. Sci.* **1**, 1193–1202 (2013).

154. S. Malladi, D. Miranda-Nieves, L. Leng, S. J. Grainger, C. Tarabanis, A. P. Nesmith, R. Kosaraju, C. A. Haller, K. K. Parker, E. L. Chaikof, A. Günther, Continuous Formation of Ultrathin, Strong Collagen Sheets with Tunable Anisotropy and Compaction. *ACS Biomater. Sci. Eng.* **6**, 4236–4246 (2020).

155. N. Cao, Y. Fu, J. He, Preparation and physical properties of soy protein isolate and gelatin composite films. *Food Hydrocoll.* **21**, 1153–1162 (2007).

156. N. Cao, X. Yang, Y. Fu, Effects of various plasticizers on mechanical and water vapor barrier properties of gelatin films. *Food Hydrocoll.* **23**, 729–735 (2009).

157. S. Farris, K. M. Schaich, L. S. Liu, P. H. Cooke, L. Piergiovanni, K. L. Yam, Gelatin-pectin composite films from polyion-complex hydrogels. *Food Hydrocoll.* **25**, 61–70 (2011).

158. Y. Li, C. Cao, Y. Pei, X. Liu, K. Tang, Preparation and properties of microfibrillated chitin/gelatin composites. *Int. J. Biol. Macromol.* **130**, 715–719 (2019).

159. C. G. Luciano, L. Tessaro, R. V. Lourenço, A. M. Q. B. Bittante, A. M. Fernandes, I. C. F. Moraes, P. J. do Amaral Sobral, Effects of nisin concentration on properties of gelatin film-forming solutions and their films. *Int. J. Food Sci. Technol.* **56**, 587–599 (2021).

160. I. Gonçalves, J. Lopes, A. Barra, D. Hernández, C. Nunes, K. Kapusniak, J. Kapusniak, D. V. Evtyugin, J. A. Lopes da Silva, P. Ferreira, M. A. Coimbra, Tailoring the surface properties and flexibility of starch-based films using oil and waxes recovered from potato chips byproducts. *Int. J. Biol. Macromol.* **163**, 251–259 (2020).

161. C. L. Luchese, J. C. Spada, I. C. Tessaro, Starch content affects physicochemical properties of corn and cassava starch-based films. *Ind. Crops Prod.* **109**, 619–626 (2017).

162. D. Domene-López, J. C. García-Quesada, I. Martin-Gullon, M. G. Montalbán, Influence of starch composition and molecular weight on physicochemical properties of biodegradable films. *Polymers (Basel).* **11**, 1084 (2019).

163. M. Cortés-Rodríguez, C. Villegas-Yépez, J. H. Gil González, P. E. Rodríguez, R. Ortega-Toro, Development and evaluation of edible films based on cassava starch, whey protein, and bees wax. *Heliyon*. **6**, e04884 (2020).

164. R. Andretta, C. L. Luchese, I. C. Tessaro, J. C. Spada, Development and characterization of pH-indicator films based on cassava starch and blueberry residue by thermocompression. *Food Hydrocoll.* **93**, 317–324 (2019).

165. C. Jiménez-Saelices, T. Trongsatitkul, D. Lourdin, I. Capron, Chitin Pickering Emulsion for Oil Inclusion in Composite Films. *Carbohydr. Polym.* **242**, 116366 (2020).

166. T. Phinainitisatra, N. Harnkarnsujarit, Development of starch-based peelable coating for edible packaging. *Int. J. Food Sci. Technol.* **56**, 321–329 (2021).

167. J. F. Smith, T. P. J. Knowles, C. M. Dobson, C. E. MacPhee, M. E. Welland, Characterization of the nanoscale properties of individual amyloid fibrils. *Proc. Natl. Acad. Sci. U. S. A.* **103**, 15806–15811 (2006).

168. Y. Y. Huang, T. P. J. Knowles, E. M. Terentjev, Strength of Nanotubes, Filaments, and Nanowires From Sonication-Induced Scission. *Adv. Mater.* **21**, 3945–3948 (2009).

169. F. S. Ruggeri, J. Adamcik, J. S. Jeong, H. A. Lashuel, R. Mezzenga, G. Dietler, Influence of the β-sheet content on the mechanical properties of aggregates during amyloid fibrillization. *Angew. Chemie - Int. Ed.* **54**, 2462–2466 (2015).

170. T. P. Knowles, A. W. Fitzpatrick, S. Meehan, H. R. Mott, M. Vendruscolo, C. M. Dobson, M. E. Welland, Role of intermolecular forces in defining material properties of protein nanofibrils. *Science*. **318**, 1900–1903 (2007).

171. S. Iwamoto, W. Kai, A. Isogai, T. Iwata, Elastic modulus of single cellulose microfibrils from tunicate measured by atomic force microscopy. *Biomacromolecules*. **10**, 2571–2576 (2009).

172. R. R. Lahiji, X. Xu, R. Reifenberger, A. Raman, A. Rudie, R. J. Moon, Atomic force microscopy characterization of cellulose nanocrystals. *Langmuir*. **26**, 4480–4488 (2010).

173. K. Tashiro, M. Kobayashi, Theoretical evaluation of three-dimensional elastic constants of native and regenerated celluloses: role of hydrogen bonds. *Polymer (Guildf).* **32**, 1516–1526 (1991).

174. T. Saito, R. Kuramae, J. Wohlert, L. A. Berglund, A. Isogai, An ultrastrong nanofibrillar biomaterial: The strength of single cellulose nanofibrils revealed via sonication-induced fragmentation. *Biomacromolecules*. **14**, 248–253 (2013).

175. I. Diddens, B. Murphy, M. Krisch, M. Müller, Anisotropic elastic properties of cellulose measured using inelastic X-ray scattering. *Macromolecules*. **41**, 9755–9759 (2008).

176. Y. Bamba, Y. Ogawa, T. Saito, L. A. Berglund, A. Isogai, Estimating the Strength of Single Chitin Nanofibrils via Sonication-Induced Fragmentation. *Biomacromolecules*. **18**, 4405–4410 (2017).

177. H. Moshe-Drezner, D. Shilo, A. Dorogoy, E. Zolotoyabko, Nanometer-scale mapping of elastic modules in biogenic composites: The nacre of mollusk shells. *Adv. Funct. Mater.* **20**, 2723–2728 (2010).

178. T. Nishino, R. Matsui, K. Nakamae, Elastic modulus of the crystalline regions of chitin and chitosan. *J. Polym. Sci. Part B Polym. Phys.* **37**, 1191–1196 (1999).

179. Y. Ogawa, R. Hori, U. J. Kim, M. Wada, Elastic modulus in the crystalline region and the thermal expansion coefficients of α-chitin determined using synchrotron radiated X-ray diffraction. *Carbohydr. Polym.* **83**, 1213–1217 (2011).

180. R. Farag, Y. Elmogahzy, in *Handbook of Tensile Properties of Textile and Technical Fibres* (2009), pp. 51–72.

181. M. F. Yu, O. Lourie, M. J. Dyer, K. Moloni, T. F. Kelly, R. S. Ruoff, Strength and breaking mechanism of multiwalled carbon nanotubes under tensile load. *Science*. **287**, 637–640 (2000).

182. L. Mott, L. Groom, S. Shaler, Mechanical properties of individual southern pine fibers: Part II. Comparison of earlywood and latewood fibers with respect to tree height and juvenility. *Wood Fiber Sci.* **34**, 221–237 (2002).

183. G. Wang, S. Q. Shi, J. Wang, Y. Yu, S. Cao, H. Cheng, Tensile properties of four types of individual cellulosic fibers. *Wood Fiber Sci.* **43**, 353–364 (2011).

184. A. Ishikawa, T. Okano, J. Sugiyama, Fine structure and tensile properties of ramie fibres in the crystalline form of cellulose I, II, IIII and IVI. *Polymer (Guildf).* **38**, 463–468 (1997).

185. M. S. Wang, D. Golberg, Y. Bando, Tensile tests on individual single-walled carbon nanotubes: Linking nanotube strength with its defects. *Adv. Mater.* **22**, 4071–4075 (2010).

186. A. Krishnan, E. Dujardin, T. Ebbesen, Young's modulus of single-walled nanotubes. *Phys. Rev. B - Condens. Matter Mater. Phys.* **58**, 14013–14019 (1998).
